# Stereoselective Diels–Alder Reactions of *gem*-Diborylalkenes: Toward the Synthesis of *gem-*Diboron-Based Polymers

**DOI:** 10.1021/jacs.1c01471

**Published:** 2021-04-14

**Authors:** Nadim Eghbarieh, Nicole Hanania, Alon Zamir, Molhm Nassir, Tamar Stein, Ahmad Masarwa

**Affiliations:** † Institute of Chemistry, The Hebrew University of Jerusalem, Jerusalem 9190401, Israel; ‡ Fritz Haber Center for Molecular Dynamics Research, Institute of Chemistry, The Hebrew University of Jerusalem, Jerusalem 9190401, Israel

## Abstract

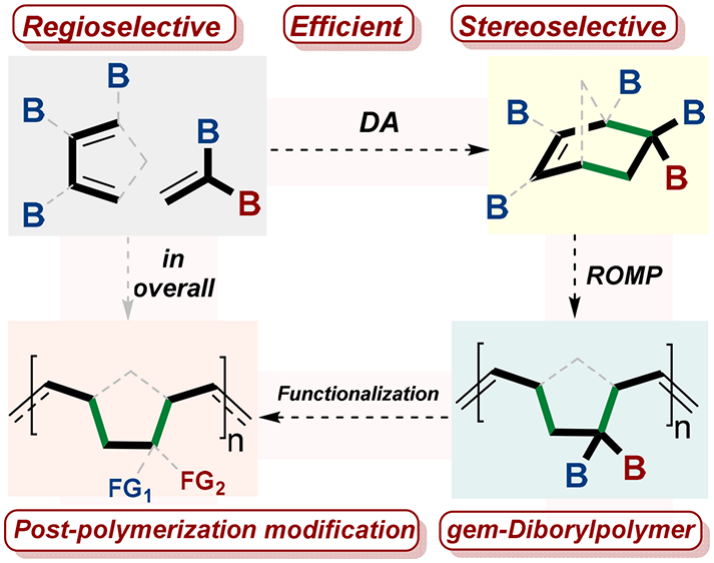

Although *gem*-diborylalkenes are known to be among the most valuable reagents in modern organic synthesis, providing a rapid access to a wide array of transformations, including the construction of C–C and C-heteroatom bonds, their use as dienophile-reactive groups has been rare. Herein we report the Diels–Alder (DA) reaction of (unsymmetrical) *gem*-diborylalkenes. These reactions provide a general and efficient method for the stereoselective conversion of *gem*-diborylalkenes to rapidly access 1,1-bisborylcyclohexenes. Using the same DA reaction manifold with borylated-dienes and *gem-*diborylalkenes, we also developed a concise, highly regioselective synthesis of 1,1,2-tris- and 1,1,3,4-tetrakis(boronates)cyclohexenes, a family of compounds that currently lack efficient synthetic access. Furthermore, DFT calculations provided insight into the underlying factors that control the chemo-, regio-, and stereoselectivity of these DA reactions. This method also provides stereodivergent syntheses of *gem-*diborylnorbornenes. The utility of the *gem-*diborylnorbornene building blocks was demonstrated by ring-opening metathesis polymerization (ROMP), providing a highly modular approach to the first synthesis of the *gem-*diboron-based polymers. Additionally, these polymers have been successfully submitted to postpolymerization modification reactions. Given its simplicity and versatility, we believe that this novel DA and ROMP approach holds great promise for organoboron synthesis as well as organoboron-based polymers and that it will result in more novel transformations in both academic and industrial research.

## Introduction

Organoboron reagents have had an enormous impact on the development of new chemical reactions^1^ and have extended the scope of accessible complex molecular scaffolds.^2−4^ Organoboronate compounds are particularly attractive owing to their wide availability and air stability,^5^ making them versatile reagents in organic synthesis.^6,7^

Although many synthetic methods utilize transformations of C–B bonds,^2^ the development of polyborylated reagents would enable greater structural diversity, an important objective.^8^ Therefore, over the past decade, much effort has been expended to synthesize new functionalized classes of polyboronates, which have been shown to be excellent building blocks for the modular construction of new compounds.^9−12^

**Figure 1:**
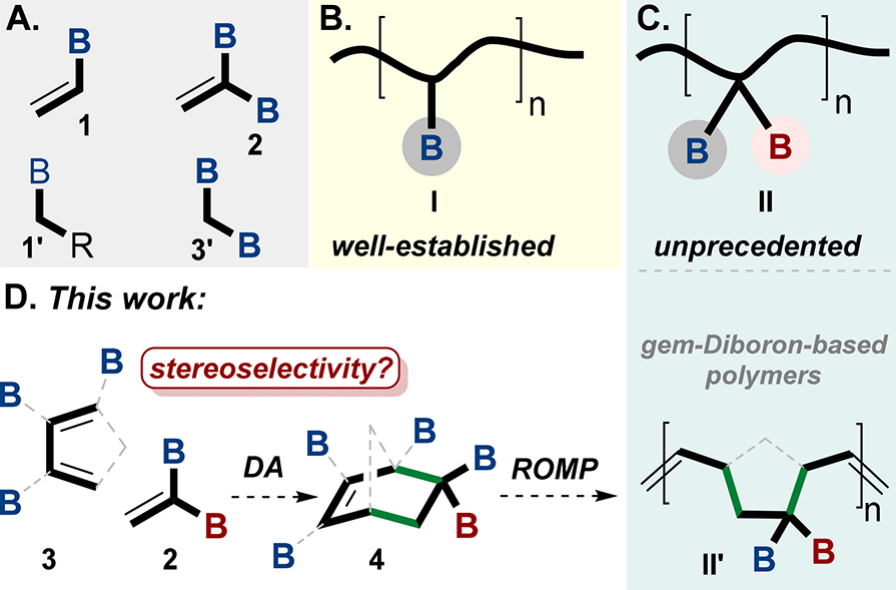
General scheme of the work. (A) Classifying of organoboron compounds. (B) The well-known boron-based polymer **I**. (C) The unprecedented *gem-*diboron based polymer **II**. (D) General scheme of the Diels–Alder reaction of polyboronated compounds **3** with **2** and the application of their cycloaddition product **4** in the ROMP reaction to generate the *gem*-diborylpolymer **II′**. B = boron group.

Among polyboron-containing structural motifs, (unsymmetrical)^13^*gem-*diboron derivatives (**2**, **3′**) are a well-known emerging class with good potential for novel synthetic applications (Figure 1A).^14−17^ The special properties and structures of bisnucleophile *gem*-diboryl compounds **2** and **3′** (termed geminated organodimetallics)^18,19^ have attracted increasing attention from synthetic chemists, particularly in constructing C–C/C–heteroatom bonds. In recent years, *gem*-diboryl compounds **2** and **3′** have been widely adopted as coupling partners in synthetic chemistry.^11,20^ For example, *gem*-diborylalkenes **2** have served as building blocks for Suzuki–Miyaura cross-coupling, nucleophilic partners, reduction approaches, Michael additions,^21^ radical chemistry,^22^ and other reactions.^13,23^

Despite the fact that organoborons **1** and **1′** (Figure 1A) have been applied in many fields including materials, polymer^24−29^**I** (Figure 1B), drugs,^30^ and, in industry, *gem-*diboryl units **2** and **3′** have seldom been employed in these fields, e.g., polymer **II** (Figure 1C).^30^ To this end, we contend that a new paradigm of research is needed to complementarily propel this *gem-*diboryl class of compounds to reach the same application level as their monoboron analogues.

As a part of a general program to investigate the reactivity and selectivity of *gem*-diboryl compounds in new synthetic applications, we sought to prepare variants bearing the *gem-*diboryl-norbornene group (**4**) because these strained compounds (∼27 kcal/mol of inherent strain) might offer new opportunities toward the ring-opening metathesis polymerization (ROMP) reaction^31^ and lead to unprecedented *gem*-diboryl-based polymer **II′** (Figure 1D).

## Results and Discussion

**Figure 2:**
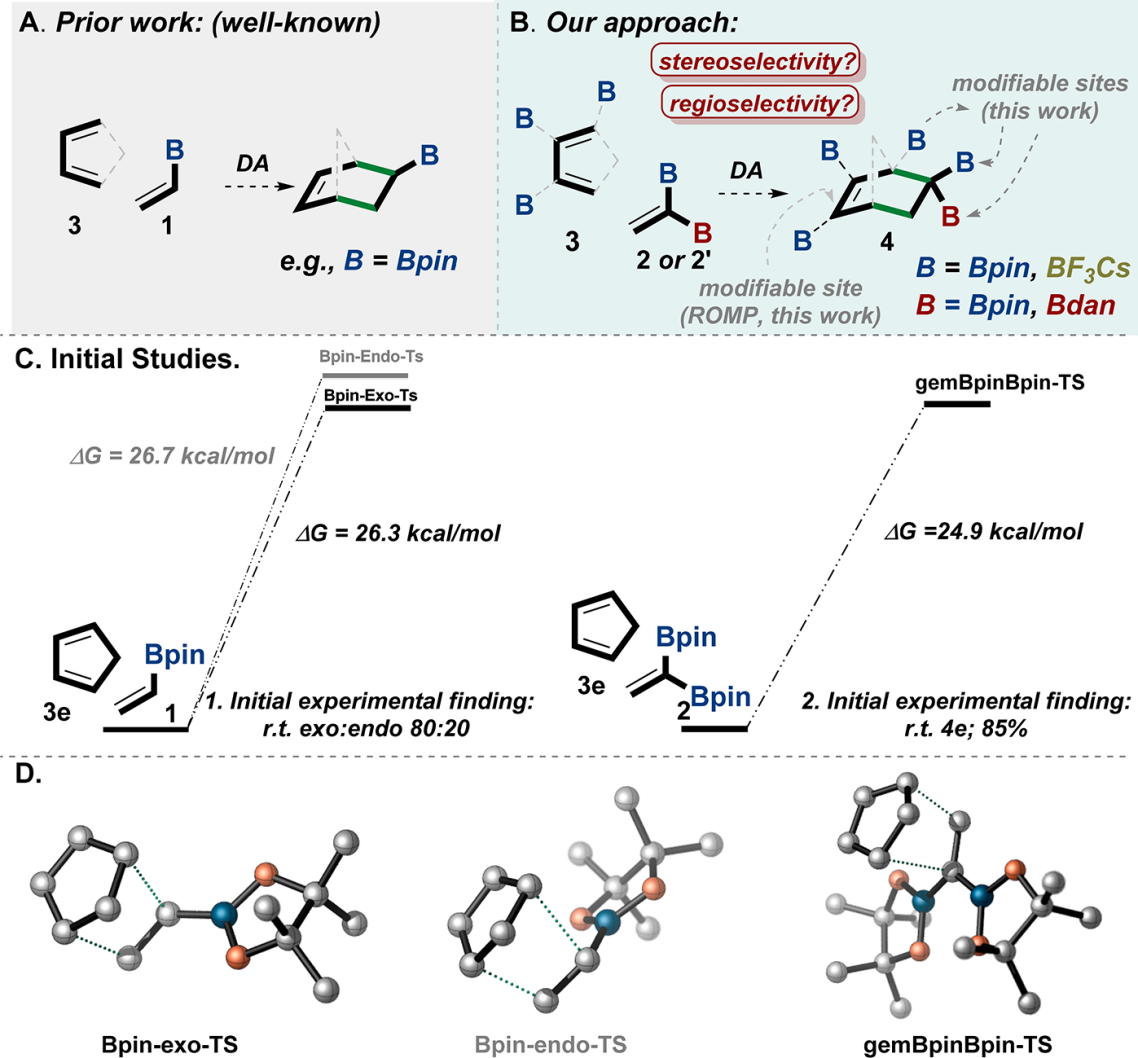
Initial study and work plan. (A) The well-known DA reaction using the vinylborantes **1** with diene **3**. (B) Description of our DA reaction of polyboronated compounds. (C) DFT calculations indicate that *gem*-diborylalkene **2** can undergo the DA reaction with diene **3e**. (D) CYLview structures of the TSs of **Bpin-exo-TS**, **Bpin-endo-TS**, and **gemBpinBpin-TS**; the calculations were performed with Gaussian 16 software using the M06-2X.^41^ TS structures were visualized with CYLview.1.0b.^42^ B = boron group, Bpin = pinacolato-boron, Bdan = B-1,8-diaminonaphthalene.

We posited that an efficient way to prepare *gem-*diboryl-norbornene structural motifs **4** would be through a [4 + 2] cycloaddition reaction of *gem-*diborylalkenes **2** with cyclopentadiene (CP) **3**. Although the [4 + 2] cycloaddition reactions of vinylboranates,^32^ e.g., **1**, are well documented in the literature (Figure 2A),^33−36^ to the best of our knowledge, the use of *gem-*diborylkenes **2** in these types of reactions is rare,^23^ despite the potential to provide new and efficient strategies to efficiently construct complex molecules (Figure 2B).^34,36^

Recently, we reported a photoredox-mediated reaction of *gem-*diborylalkenes^22^ and showed that *gem*-diborylalkenes **2** have similar electron deficiency as vinylboron **1**;^22^ hence, **2** should serve as a suitable dienophile for this type of cycloaddition reaction. However, key challenges include (1) the steric repulsion introduced by the two groups of the bulky Bpin units in the TS of the cycloaddition reaction;^37^ (2) whether the regio- and stereoselectivity of the cycloaddition can be controlled when two unsymmetrical boron groups are placed on the geminated carbon of dienophile **2′**;^13^ and (3) whether the reaction can proceed in a regioselective manner when borylated dienes react with **2** (Figure 2B).^34^

To answer these questions, we first conducted computational studies on the Diels–Alder (DA) reaction to predict whether *gem*-diborylalkenes **2** could be used as dienophile reactive partners for the DA reaction.^35,38^ According to our computational studies of the energy profiles of the cycloaddition reactions of diene-Cp **3e** with dienophiles **1** and **2** at room temperature (rt), the transition state of **gemBpinBpin-TS** is likely to be more energetically stabilized compared to vinyboronate TS **Bpin-exo-TS** by 1.4 kcal/mol (Figure 2C,D). Considerations for the relative stabilities of the different transition states are discussed in the SI (pp S65, S66).^36,38,39^ Moreover, the CHelpG population analysis^40^ on the carbons of the reactive double bond of the dienophiles (**1** and **2**) shows that the double bond of **2** has less electron concentration than the double bond in **1** (full details are given in the SI (pp S65, S66). These observations support the fact that **2** is slightly more “dienophilic” toward the DA reaction than **1**, albeit more bulky (Figure 2C,D).

**Figure 3:**
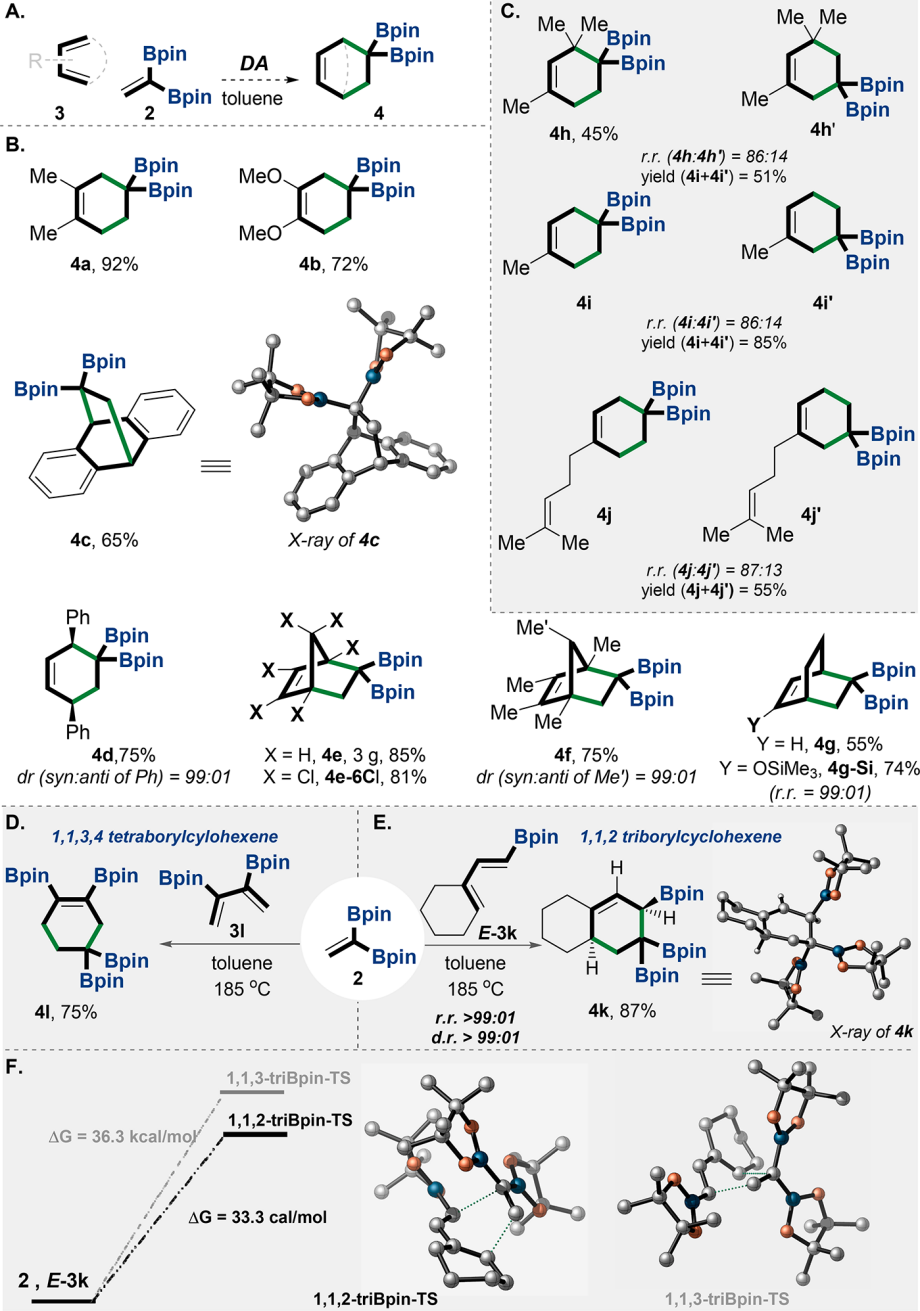
Diels–Alder reaction with **2**. (A) General reaction conditions of the DA with **2**. (B) examples of the DA products as a result of reaction of **2** with symmetrical dienes. (C) Examples of the DA products as a result of a regioselective reaction of **2** with unsymmetrical dienes. (D) Preparation of the 1,1,3,4-tetraBpincyclohexene **4l** by the DA reaction of **2** with diene **3l**. (E) Regioselective preparation of the 1,1,2-triBpincyclohexene adduct **4k** by the stereospecific DA reaction of **2** with diene *E***-3k**. (F) DFT calculations for the regioselective rationale by the TSs of **1,1,2-triBpin-TS** and **1,1,3-triBpin-TS**; the calculations were performed with Gaussian 16 software using M06-2X.^41^ The relative structures of **4f** and **4g-Si** have been confirmed by 2D NMR NOESY. X-ray and TS structures were visualized with CYLview 1.0b.^42^ rr = regioisomeric ratio, dr = diastereomeric ratio, yields are isolated. Bpin = pinacolato-boron.

To investigate our proposed reaction, *gem*-diborylalkene **2**, along with **3e**, was subjected to DA reaction conditions (Figures 2C-2, 3A,B). We obtained the desired *gem*-diborylated cycloaddition product **4e** in good yield at rt with toluene as solvent. Next, we investigated the scope of the DA reaction using readily available *gem*-diborylalkene **2** and various diene substrates (**3**) bearing aliphatic, aromatic, and heteroatom substituents (Figure 3B). Generally, the products (**4a**–**g**) were isolated in good yields under the established optimal conditions. The reaction also proceeded in very good yield on a gram scale, e.g., **4e**. The reaction works well with anthracene derivative **3c**, affording the *gem*-diborylcyclic adduct **4c**, which was confirmed by X-ray crystallographic analysis (see the CYLview structure of **4c**, Figure 3B). In addition, the reaction works smoothly with hexachlorocyclopentadiene **3e-6Cl**, forging the hexachloro cycloaddition product **4e-Cl**.

Using bulky dienes (i.e., pentamethylcyclopentadiene **3f**), along with adjusting the reaction temperature, played a critical role in controlling diastereoselectivity (i.e., the *syn* vs *anti* outcomes on **4f**; Figure 3B). Whereas at a high temperature (185 °C) the reaction proceeds with moderate diastereoselectivity (dr = 83:17), at room temperature the reaction afforded the bis-borylated compound **4f** as the exclusive diastereoisomer (dr > 99:01). Interestingly, the product *gem*-diboryl-bicyclo[2.2.2]octane **4g-Si** was obtained in a high regioselective mode, i.e., rr > 99:01 (Figure 3B).

The reaction also exhibits moderate regioselectivity when unsymmetrical dienes are used, giving the *para* adducts (**4h**–**j**, Figure 3C). Notably, the constitutional isomers in **4** (**h**, **h′**) and **4** (**i**, **i′**) can be easily separated by column chromatography (Figure 3C; see the SI).

Remarkably, the reaction of **2** with 3,4-diboryl diene **3l** can create the exceptional 1,1,3,4-tetraborylcyclohexene skeleton **4l** (Figure 3D). Moreover, we examined the DA reaction of 1-boron-diene^34,43^*E***-3k** with *gem-*diborylalkene **2**, and surprisingly, the reaction generated the rare 1,1,2-triboryl cyclic adduct **4k** as the exclusive isomer with complete stereospecificity (Figure 3E).^12,43,44^ We have obtained unambiguous support for the structures of **4k** using X-ray crystallographic analysis (see CYLviews in Figure 3E). DFT calculations indicate that the TS of the 1,1,2-triboryl constitutional isomer is favored by 3 kcal/mol over the TS of the 1,1,3-triboryl isomer (**1,1,2-triBpin-TS** vs **1,1,3-triBpin-TS**; Figure 3F).

**Figure 4:**
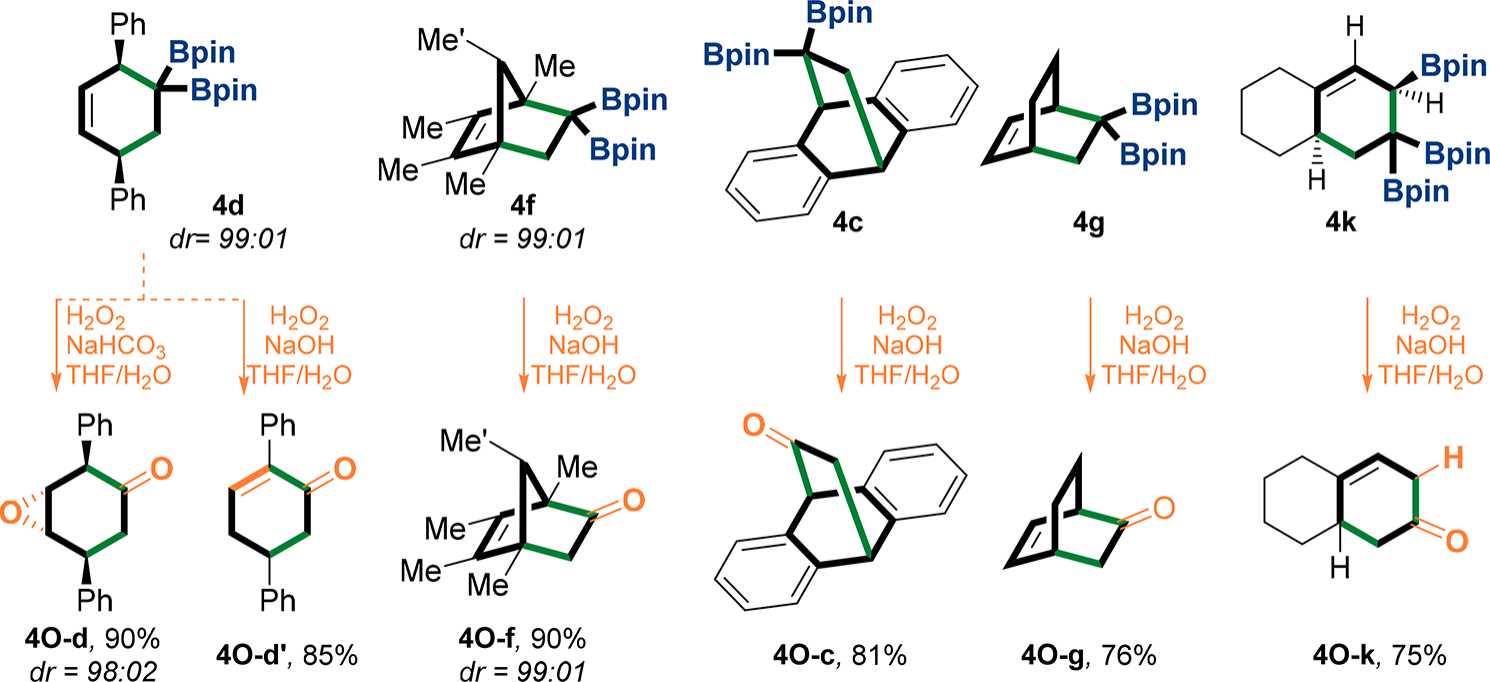
Oxidation reaction of **4**. Examples of the utility of *gem*-diborylcyclohexenes **4** in the oxidation reaction that yields the ketone products **4O**. The relative structure **4O-d** has been confirmed by 2D NMR NOESY. rr = regioisomeric ratio, dr = diastereomeric ratio, yields are isolated. Bpin = pinacolato-boron.

Next, the polyborylated cycloadducts **4** were subjected to oxidation reactions using H_2_O_2_ (Figure 4).^45^ Oxygenated compounds **4O** were obtained in a chemoselective manner (Figure 4). Thus, we have demonstrated that *gem*-diborylalkenes serve as ketene equivalents in [4 + 2] cycloadditions.^46^ Of note, **4d** has been subjected to two oxidation reactions that lead chemoselectively to two different products, **4O-d** and **4O-d′**. In **4O-d**, first the double bond was stereoselectively epoxidized and then the *gem*-di-Bpin unit underwent oxidation. However, in **4O-d′** the geminal-Bpin position first underwent oxidation and then the double bond isomerizes due to the deprotonation of the acidic benzylic and allylic proton that is located α to the generated carbonyl (Figure 4; see proposed pathways in the SI). When triboryl compound **4k** was subjected to the oxidation conditions, ketone **4O-k** was selectively obtained, most likely resulting from oxidation of the *gem*-diboryl moiety to give a boron-enolate,^47^ which hydrolyzed in situ to finally give **4O-k** (Figure 4; see proposed pathways in the SI).

**Figure 5:**
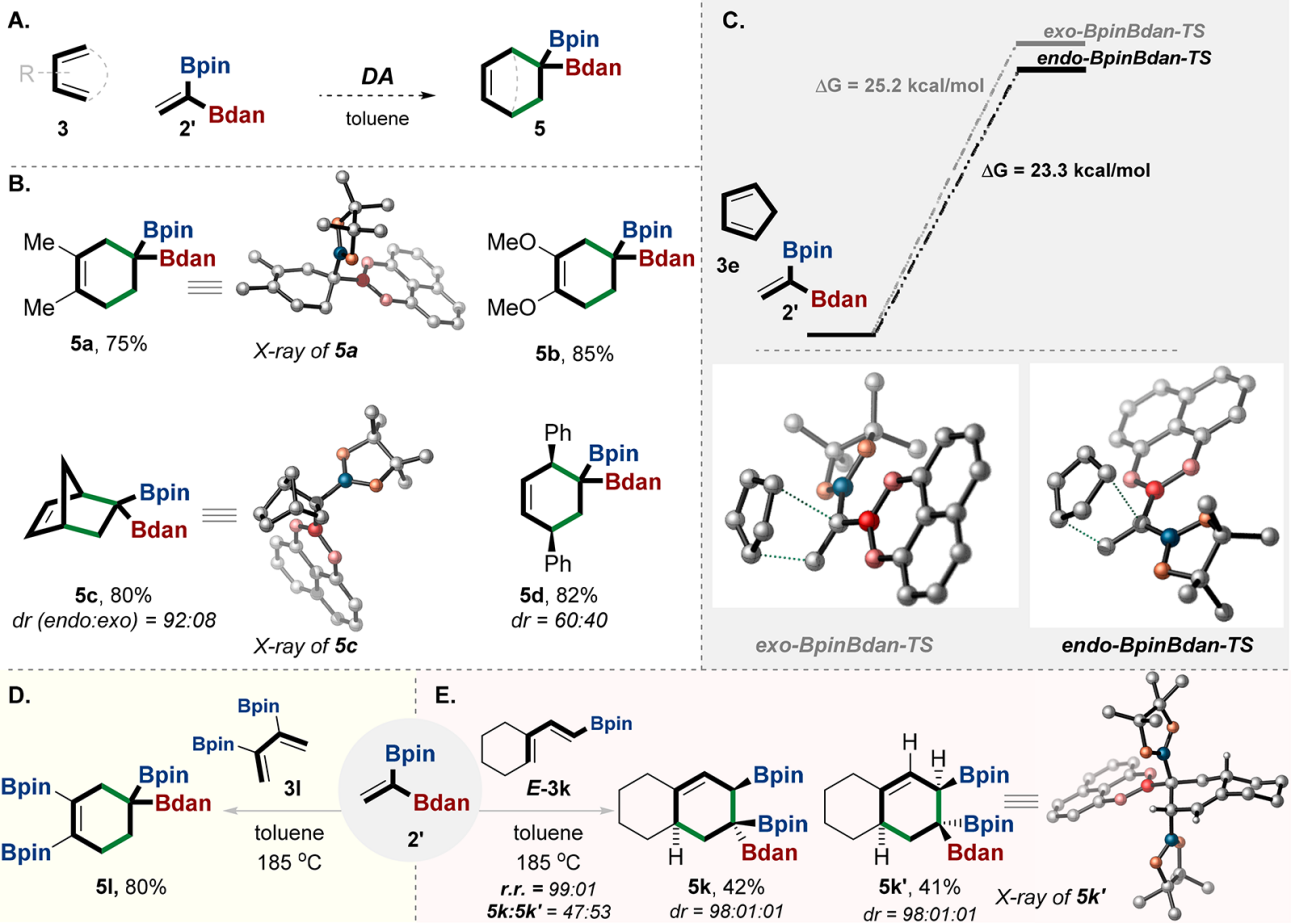
Diels–Alder reaction with **2′**. (A) General reaction conditions of the DA with unsymmetrical *gem*-diborylalkene **2′**. (B) Examples of the DA products as a result of reaction of **2** with different dienes. (C) DFT calculations of the TSs of **exo-BpinBdan-TS** and **endo-BpinBdan-TS**; the calculations were performed with Gaussian 16 software using M06-2X.^41^ (D) DA reaction of **2′** with diene **3l** leads to 1-Bdan-1,3,4-triBpin-cyclohexene **5l**. (E) Stereospecific DA reaction of **2′** with diene *E***-3K** leads regioselectively to diastereomers **5k** and **5k′**; the two 1,1,2-triboryl products were easily separated by column chromatography. The 1,2-regioselectivity manner has been determined by the X-ray structure of **5k′**. The relative configuration of **5k** has been confirmed by 2D NMR NOESY. X-ray and TS structures were visualized with CYLview 1.0b.^42^ rr = regioisomeric ratio, dr = diastereomeric ratio, yields are isolated. Bpin = pinacolato-boron, Bdan = B-1,8-diaminonaphthalene.

Furthermore, we investigated the DA reaction using unsymmetrical 1,1-bisdiborylalkenes (**2′** and **2-F**). It was anticipated that the cycloaddition might proceed with good stereoselectivity (Figure 5).^13^ We were happy to discover that the reaction of 1,1-BpinBdan-ethene (**2′**) with dienes **3a**–**d** afforded the unsymmetrical *gem-*diborylalkane **5a**–**d** (confirmed by X-ray crystallographic analyses of **5a** and **5c**) in good yield (Figure 5A,B). High diastereoselectivity was observed in the reaction of **2′** with CP, affording cycloaddition product **5c** (*endo*:*exo* = 92:8). Our calculations indicate that the reaction favors the *endo* product **5c** with the TS energy of **endo-BpinBdan-TS**, which is 2 kcal/mol less than that of **exo-BpinBdan-TS** (Figure 5C).^24,35,38^ This is in good agreement with the fact that the aromatic-planar Bdan group is less bulky than the Bpin group.^13,33,37,48^ Therefore, it would appear that the diastereoselectivity in this case is driven primarily by sterics (additional considerations for the relative stabilities of the different transition states are discussed in the SI (pp S65, S66)).

Moreover, the reaction of **2′** with 3,4-diboryl diene **3l** yielded 1,1,3,4-tetraborylcyclohexene adduct **5l** (Figure 5D). Additionally, the DA reaction of 1-boron-diene^34,43^ (*E***-3k**) with *gem-*diborylalkene **2′** generated regioselectively the separable diastereomers of the 1,1,2-triboryl cyclic adduct with complete stereospecificity (**5k**, **5k′**; Figure 5E).^43,44^ We have obtained unambiguous support for the structures of **5k′** using X-ray crystallographic analysis (see CYLviews in Figure 5E). These two different boron groups can provide the basis for selective C–B sequential functionalization, which in turn reacts differently.^13,49^

**Figure 6:**
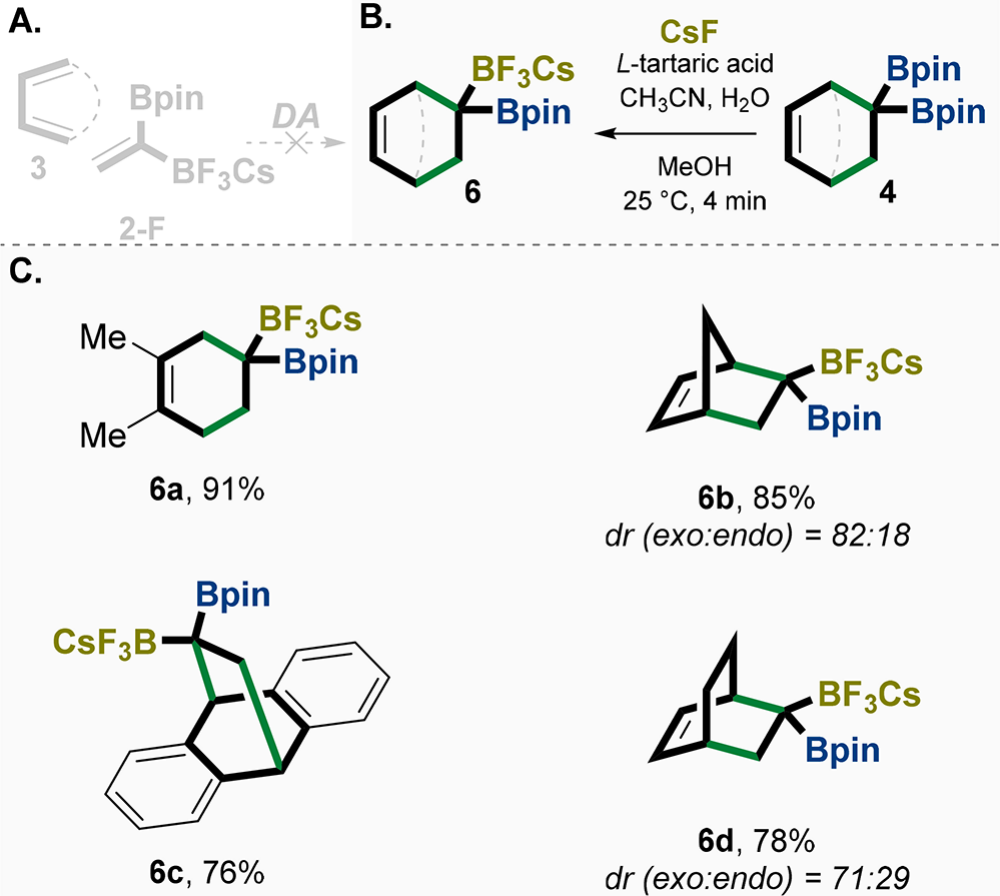
(A) Unsuccessful Diels–Alder reaction with **2-F**. (B) General reaction conditions of the chemoselective trifluorination with *gem*-diborylalkanes **4** as an alternative method for the unsuccessful direct DA of **2-F** with diene **3**. (C) Examples of the trifluorination products as a result of reaction of **2** with different dienes. The relative configurations of **6b** and **6d** have been confirmed by 2D NMR NOESY. dr = diastereomeric ratio, yields are isolated. Bpin = pinacolato-boron.

Unlike boronic esters, for example, the Bpin and Bdan groups, monoalkyl-trifluoroborate salts are known to be easily activated and to undergo rapid transmetalation with transition-metal complexes. In general, owing to their air, moisture, shelf, and thermal stability, as well as their occurrence as free-flowing crystalline solids, monotrifluoroborate salts have now become extremely popular reagents in synthesis. Thus, we attempted to use the *gem-*BpinBF_3_Cs alkene **2-F** as a dienophile for the DA reaction (Figure 6A). Unfortunately, the reaction did not afford the desired product (*gem-*diborylcyclohexene BF_3_Cs containing **6**) and instead led to decomposition (Figure 6A). Alternatively, we sought to obtain product **6** using our recently reported conditions for late-stage trifluoroboration of *gem-*diborylalkenes by employing CsF (Figure 6B,C).^50^ We were pleased to observe the desired mono-BF_3_Cs products **6a**–**d** in good yield (Figure 6C). Moreover, the reaction shows moderate diastereoselectivity in **6b** and **6d** when **4e** and **4g**, respectively, are used. The rationale of the diastereoselectivity is in good agreement with our reported mechanism that follows the likelihood that fluorination occurs from the less sterically encumbered face of norbornenes **4e**–**g**, affording the *exo*-disposed BF_3_Cs group (**6b** and **6d**, respectively).^50^

**Figure 7:**
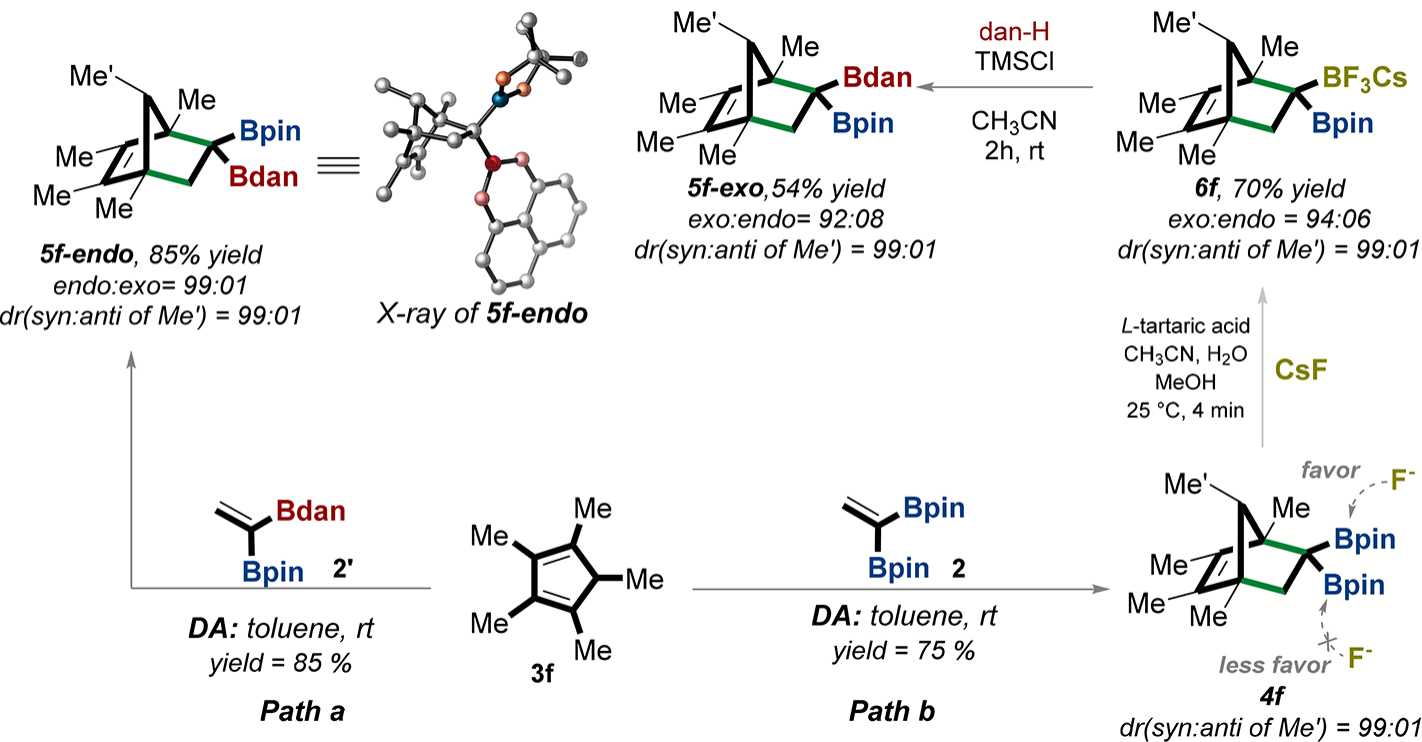
Stereodivergent synthesis of norbornene **5f**. Path a: Norbornenediastereomer **5f-endo** was stereoselectivity synthesized in one step from DA reaction of cyclopentadiene **3f** with the unsymmetrical *gem*-diborylalkene **2′**. The relative configuration of **5f-endo** was determined using X-ray crystallography (see the CYLview structure of **5f-endo**). Path b: **5f-exo** was synthesized in three steps. Step 1: diastereoselective DA reaction of **3f** with *gem*-diborolalkene **2**, which leads to norbornene **4f**. Step 2: stereoselective trifluorination of norbornene **4f** from the less hindered *si-*face, leading to the exo product **6f**. Step 3: the *gem*-diborylnorbornene-BF_3_Cs **6f** was directly converted to unsymmetrical *gem*-Bpin Bdan-norbornene **5f-exo** in a stereospecific manner. X-ray structures were visualized with CYLview 1.0b.^42^ dr = diastereomeric ratio, yields are isolated. Bpin = pinacolato-boron, Bdan = B-1,8-diaminonaphthalene.

Next, we envisioned a stereodivergent synthesis of norbornene **5f** involving controlling *exo* and *endo* norbornene structural motifs as illustrated in Figure 7. Toward this goal, cyclopentadienyl **3f** was reacted in two different scenarios. In path a (Figure 7), **3f** reacted with the unsymmetrical *gem*-diborylalkene **2′** to form only the cycloaddition product **5f-***endo* (confirmed by X-ray) in 85% yield. In path b (Figure 7), **3f** was first reacted with the symmetrical *gem*-BpinBpin-alkene **2** to yield **4f** as a single diastereomer, which was then subjected to a diastereoselective trifluoroboration favoring the less sterically hindered face of the norbornene (denoted by arrows in **4f**) to afford **6f** in high diastereoselectivity (94:06; Figure 7).^50^ Finally, the BF_3_Cs group was converted to the Bdan group to afford the **5f-exo** product (Figure 7).^50^ Overall, our method serves as a powerful tool for the diastereocontrolled synthesis of norbornene motifs.

**Figure 8:**
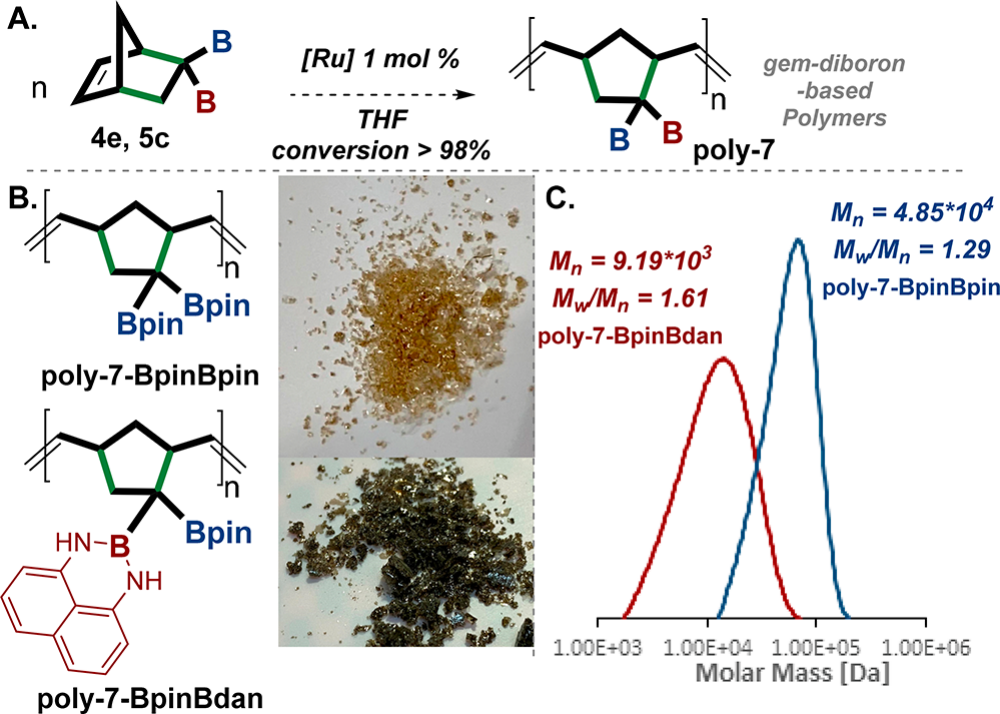
Synthesis of *gem-*diboron-based polymers. (A) General scheme of the catalyzed Ru ROMP of norbornenes **4e** and **5c** to yield polymers **poly-7-BpinBpin** and **poly-7-BpinBdan** with >98% conversion. (B) The obtained polymers. (C) Polydispersity indexes (PDI) and gel permeation chromatography (GPC) of both polymers. Thermogravimetric analysis (TGA) for both polymers **poly-7-BpinBpin** and **poly-7-BpinBdan** is provided in the SI (p S51). [Ru] = Grubbs second-generation catalyst. Bpin = pinacolato-boron, Bdan = B-1,8-diaminonaphthalene.

With these valuable *gem*-diborylcyclohexenes in hand, e.g., *gem-*diborylnorbornene, we sought to demonstrate their synthetic utility in selective transformations of the double bond through the ROMP reaction, as depicted in Figure 8A, to generate the novel *gem-*diborylalkene-based polymers.

Organoboron polymers^29^ have attracted widespread attention, since they provide unique properties for catalysis, sensory materials, luminescent materials, and biomedical applications.^26,27,29,30^ Among them, polymers with pendant boronic acids/esters account for the majority, since boronic acids/esters could serve as responsive sites of sensitive materials or dynamic cross-linking points of self-assembled polymers and self-healing materials.^26,27,30^

Moreover, the presence of the boron moiety on the polymer offers a great opportunity for postpolymerization modifications^28,29^ to achieve new functional groups that are difficult to achieve in regular polymerizations.^24,25,29^ Although polymers that contain monoboron units have been widely used,^27^ their *gem-*diboryl analogues have not been investigated; thus, they can provide a new array of polymer properties to forge a wider diversity of *gem-*diboron-based polymers. Thus, *gem-*diborylnorbornenes hold great promise to serve as a monomer for ROMP^31^ reactions that form polymers containing *gem*-diboryl units (Figure 8A). It is noteworthy that the ROMP reactions for the mono-boron-containing monomers are rarely reported, and there are only a few examples of very special side-chain boron groups.^51^

In this regard, we chose the [Ru] Grubbs second-generation catalyst as a catalyst in the ROMP reactions, because its known to tolerate broad functional groups, air, and moisture, and this includes the fact that the Bpin group is expected to be intact under the Ru catalysis conditions for other types of reactions, i.e., olefin metathesis catalysts.^52^

We subjected norbornenes **4e** and **5c** to ROMP^31^ polymerization reaction conditions using 1 mol % of the [Ru] Grubbs second-generation catalyst and tetrahydrofuran (THF) as a solvent.^53^ We were gratified to observe that *gem*-diborylnorbornene **4e** and **5c** selectively polymerized to afford, for the first time, *gem-*diboron-based polymers **poly-7-BpinBpin** and **poly-7-BpinBdan**, respectively, in high conversions (Figure 8B). This transformation was followed by ^1^H NMR spectroscopy of both monomers **4e** and **5c** and their corresponding polymers **poly-7-BpinBpin** and **poly-7-BpinBdan**, respectively, as depicted in the SI (pp S45–S49, S64).^25^ The resulting polymer, **poly-7-BpinBpin**, was observed to have a light yellowish color; it has a high molecular weight (MW = 4.85 × 10^4^) and polydispersity indexes (PDI) of *M*_w_/*M*_n_ = 1.29, based on gel permeation chromatography (GPC) (Figure 8B,C). Furthermore, the polymer **poly-7-BpinBdan** was dark green and has a lower molecular weight (MW = 9.19 × 10^3^) and a higher PDI of *M*_w_/*M*_n_ = 1.61 (Figure 8B,C).^24,25,31,39^ Although these pristine polymers are expected to be colorless, the dark color of the **poly-7-BpinBdan** might be a result of the conjugated nature of the aromatic Bdan group. While both polymers have different MW values, the **poly-7-BpinBpin** is closer to the one expected at a 1 mol % catalyst compared with literature.^51^

**Figure 9:**
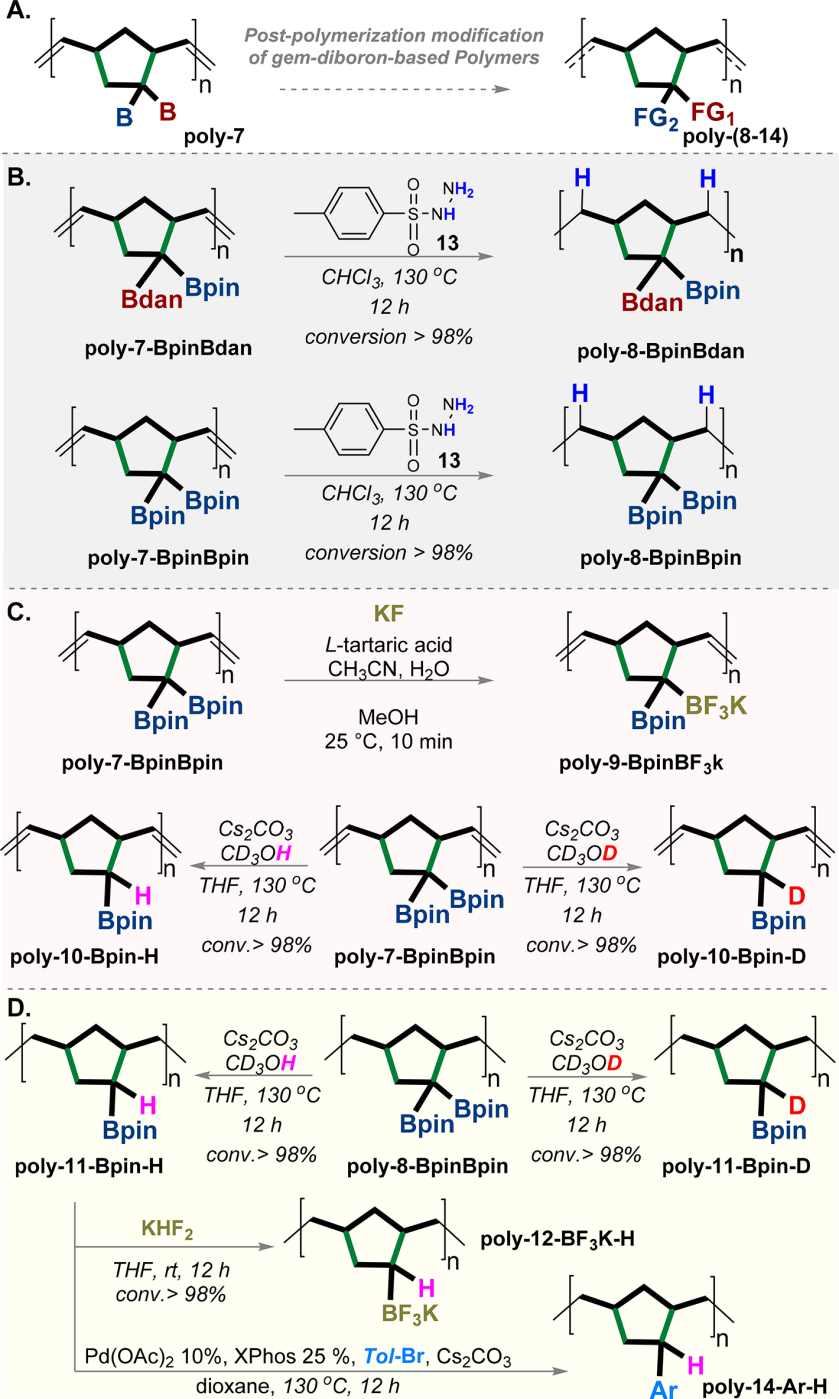
Postpolymerization modifications of *gem-*diboron-based polymers. (A) General scheme of the postpolymerization modifications of **poly-7**. (B) Hydrogenation of polymers **poly-7-BpinBpin** and **poly-7-BpinBdan**. (C) Trifluorination, protodeborylation, and deuterodeborylation reactions of **poly-7-BpinBpin**. (D) Examples of postpolymerization modifications of **poly-8-BpinBpin** and **poly-11-Bpin-H** which include trifluorination, protodeborylation, deuterodeborylation, and arylation. Bpin = pinacolato-boron, Bdan = B-1,8-diaminonaphthalene.

Subsequently, we examined the potential of postpolymerization modifications of these *gem*-diboron-based polymers by the functionalization of the double bond and the replacements of *gem-*diboryl units (Figure 9A). These modifications include the hydrogenation of the ethylene moieties using *p*-toluenesulfonyl hydrazide **13** as a source for hydrogen (Figure 9B).^54,55^ The hydrogenation reactions proceed very efficiently for both *gem*-diboron-based polymers **poly-7-BpinBpin** and **poly-7-BpinBdan** to obtain the new hydrogenated polymers **poly-8-BpinBpin** and **poly-8-BpinBdan**, respectively, in a complete conversion (Figure 9B). The hydrogenated polymers **poly-8-BpinBpin** and **poly-8-BpinBdan** were distinctly confirmed by ^1^H and ^13^C NMR spectroscopy (for full details see the SI).^55^

Our efforts were also directed to the postpolymerization transformation of the boron groups through the selective replacement of one of the boron groups by trifluorination,^50^ protodeborylation,^56^ and deuterodeborylation^56^ as described in Figure 9C. Remarkably, the trifluorination^50^ reaction of polymer **poly-7-BpinBpin** represents an alternative method to the unsuccessful direct ROMP reaction of monomer Bpin-BF_3_Cs-norbornene **6b**. Of note, these new polymers were confirmed by ^1^H, D, ^19^F, and ^13^C NMR spectroscopy (for full details see the SI).

Moreover, the hydrogenated polymer **poly-8-BpinBpin** underwent selective protodeborylation,^56^ and deuterodeborylation,^56^ which were then followed by a trifluorination reaction^22,50^ of **poly-11-Bpin-H** (Figure 9D). Overall, **poly-7-BpinBpin** underwent three chemoselective sequential postpolymerization modifications, i.e., hydrogenation, protodeborylation, and trifluorination, which eventually gave **poly-7-BF**_**3**_**K-H** (Figure 9B and D). Finally, **poly-11-Bpin-H** was subjected to the Pd-catalyzed Suzuki–Miyaura cross-coupling reaction, to replace the C–B bond with the new C–C bond, which forms the arylated polymer **poly-14-Ar-H** (Figure 9D).

These preliminary postpolymerization transformations have demonstrated the utility of a replaceable-main-chain-based strategy using boron and the double bond as the key elements for opening new opportunities for the synthesis of a variety of new polymers.

## Summary

In conclusion, we have developed a method that addresses the long-standing challenge of regio- and stereoselective Diels–Alder cycloadditions with poly-alkenylboranes. This was achieved by introducing a new method that enables the use of (unsymmetrical) *gem-*diborylalkenes as a reasonable reactive dienophile for the DA reaction. The products of these reactions enable the formal synthesis of polyborylated cycloadducts, particularly the 1,1,2-tri- and 1,1,3,4-tetraborylcyclic adduct, which would be difficult to accomplish with the existing strategies. In addition, the reaction offers the stereodivergent synthesis of norbornenes by using a diastereoselective trifluorination reaction. We demonstrate the use of the *gem*-diborylalkenes as ketene equivalents in [4 + 2] cycloadditions. Moreover, we utilized *gem-*diborylnorbornene in the synthesis, for the first time, of *gem-*diboryl-based polymers through ROMP. These polymers underwent successful postpolymerization modifications to access new polymers, which also demonstrates the potential diversity of the main chain replacement. Studies to achieve an enantioselective DA transformation using chiral *gem-*diborylalkenes^33^ as well as new postpolymerization transformations of *gem*-diborylcycloalkene-based polymers are currently under investigation and will be reported in due course.

## Supporting Information Available

Supporting information and chemical compound information (PDF)

## Accession Codes

CCDC 2058307–2058312 contain the supplementary crystallographic data for this paper. These data can be obtained free of charge via www.ccdc.cam.ac.uk/data_request/cif, or by emailing data_request@ccdc.cam.ac.uk, or by contacting The Cambridge Crystallographic Data Centre, 12 Union Road, Cambridge CB2 1EZ, UK; fax: +44 1223 336033.

## References

[ref1] Blakemore D. C.; Castro L.; Churcher I.; Rees D. C.; Thomas A. W.; Wilson D. M.; Wood A. Organic synthesis provides opportunities to transform drug discovery. Nat. Chem. 2018, 10, 383–394. 10.1038/s41557-018-0021-z. 29568051

[ref2] Sandford C.; Aggarwal V. K. Stereospecific functionalizations and transformations of secondary and tertiary boronic esters. Chem. Commun. 2017, 53, 5481–5494. 10.1039/C7CC01254C. 28397919

[ref3] Hall D. Boronic Acids Preparation and Applications in Organic Synthesis and Medicine Preface. In Boronic Acids: Preparation and Applications in Organic Synthesis and Medicine; Hall D., Ed.; Wiley-VCH Verlag GmbH& Co. KGaA: Weinheim, 2005.

[ref4] Fyfe J. W. B.; Watson A. J. B. Recent Developments in Organoboron Chemistry: Old Dogs, New Tricks. Chem. 2017, 3, 31–55. 10.1016/j.chempr.2017.05.008.

[ref5] Knochel P.; Ila H.; Korn T.; Baron O. Functionalized Organoborane Derivatives in Organic Synthesis. In Handbook of Functionalized Organometallics; Wiley-VCH, 2005; pp 45–108 10.1002/9783527619467.ch3.

[ref6] Fernández E.; Whiting A. Synthesis and Application of Organoboron Compounds. In Topics in Organometallic Chemistry; Springer International Publishing, 2015; pp 1–331 10.1007/978-3-319-13054-5.

[ref7] Crudden C. M.; Glasspoole B. W.; Lata C. J. Expanding the scope of transformations of organoboron species: carbon-carbon bond formation with retention of configuration. Chem. Commun. 2009, 6704–6716. 10.1039/b911537d. 19885455

[ref8] Miura T.; Nakahashi J.; Sasatsu T.; Murakami M. Synthesis of gamma-Boryl-Substituted Homoallylic Alcohols with anti Stereochemistry Based on a Double-Bond Transposition. Angew. Chem., Int. Ed. 2019, 58, 1138–1142. 10.1002/anie.201811205. 30474905

[ref9] Gao S.; Duan M.; Shao Q.; Houk K. N.; Chen M. Development of alpha, alpha-Disubstituted Crotylboronate Reagents and Stereoselective Crotylation via Bronsted or Lewis Acid Catalysis. J. Am. Chem. Soc. 2020, 142, 18355–18368. 10.1021/jacs.0c04107. 33052047

[ref10] Hu J. F.; Zhao Y.; Shi Z. Z. Highly tunable multi-borylation of gem-difluoroalkenes via copper catalysis. Nat. Catal. 2018, 1, 860–869. 10.1038/s41929-018-0147-9.

[ref11] Teo W. J.; Yang X. X.; Poon Y. Y.; Ge S. Z. Cobalt-catalyzed deoxygenative triborylation of allylic ethers to access 1,1,3-triborylalkanes. Nat. Commun. 2020, 11, 5193 10.1038/s41467-020-19039-7. 33060600 PMC7562742

[ref12] Coombs J. R.; Zhang L.; Morken J. P. Enantiomerically Enriched Tris(boronates): Readily Accessible Conjunctive Reagents for Asymmetric Synthesis. J. Am. Chem. Soc. 2014, 136, 16140–16143. 10.1021/ja510081r. 25387002 PMC5539535

[ref13] Babu K. N.; Massarwe F.; Reddy R. R.; Eghbarieh N.; Jakob M.; Masarwa A. Unsymmetrical 1,1-Bisboryl Species: Valuable Building Blocks in Synthesis. Molecules 2020, 25, 959 10.3390/molecules25040959. PMC7070756 32093409

[ref14] Zuo Z.; Huang Z. Synthesis of 1,1-diboronate esters by cobalt-catalyzed sequential hydroboration of terminal alkynes. Org. Chem. Front. 2016, 3, 434–438. 10.1039/C5QO00426H.

[ref15] Zhao H.; Tong M.; Wang H.; Xu S. Transition-metal-free synthesis of 1,1-diboronate esters with a fully substituted benzylic center via diborylation of lithiated carbamates. Org. Biomol. Chem. 2017, 15, 3418–3422. 10.1039/C7OB00654C. 28387416

[ref16] Li L.; Gong T.; Lu X.; Xiao B.; Fu Y. Nickel-catalyzed synthesis of 1,1-diborylalkanes from terminal alkenes. Nat. Commun. 2017, 8, 345 10.1038/s41467-017-00363-4. 28839152 PMC5571201

[ref17] Nallagonda R.; Padala K.; Masarwa A. gem-Diborylalkanes: recent advances in their preparation, transformation and application. Org. Biomol. Chem. 2018, 16, 1050–1064. 10.1039/C7OB02978K. 29379940

[ref18] Marek I.; Normant J. F. Synthesis and reactivity of sp(3)-geminated organodimetallics. Chem. Rev. 1996, 96, 3241–3267. 10.1021/cr9600161. 11848859

[ref19] Marek I. Synthesis and reactivity of sp(2) geminated organobismetallic derivatives. Chem. Rev. 2000, 100, 2887–2900. 10.1021/cr990288e. 11749308

[ref20] Hu M.; Ge S. Z. Versatile cobalt-catalyzed regioselective chain-walking double hydroboration of 1, n-dienes to access gem-bis(boryl)alkanes. Nat. Commun. 2020, 11, 10.1038/s41467-020-14543-2. PMC7005816 32034153

[ref21] Liang M. Z.; Meek S. J. Catalytic Enantioselective Synthesis of 1,4-Keto-Alkenylboronate Esters and 1,4-Dicarbonyls. Angew. Chem., Int. Ed. 2019, 58, 14234–14239. 10.1002/anie.201907757. PMC6764896 31353794

[ref22] Kumar N.; Eghbarieh N.; Stein T.; Shames A. I.; Masarwa A. Photoredox-Mediated Reaction of gem-Diborylalkenes: Reactivity Toward Diverse 1,1-Bisborylalkanes. Chem. - Eur. J. 2020, 26, 5360–5364. 10.1002/chem.202000603. 32141638

[ref23] Royes J.; Cuenca A. B.; Fernandez E. Access to 1,1-Diborylalkenes and Concomitant Stereoselective Reactivity. Eur. J. Org. Chem. 2018, 2018, 2728–2739. 10.1002/ejoc.201701786.

[ref24] Nishikawa T.; Ouchi M. An Alkenyl Boronate as a Monomer for Radical Polymerizations: Boron as a Guide for Chain Growth and as a Replaceable Side Chain for Post-Polymerization Transformation. Angew. Chem., Int. Ed. 2019, 58, 12435–12439. 10.1002/anie.201905135. 31283869

[ref25] He C. Z.; Pan X. C. MIDA Boronate Stabilized Polymers as a Versatile Platform for Organoboron and Functionalized Polymers. Macromolecules 2020, 53, 3700–3708. 10.1021/acs.macromol.0c00665.

[ref26] Chauhan N. P. S.; Hosmane N. S.; Mozafari M., Boron-based polymers: opportunities and challenges. Mater. Today. Chem. 2019, 14, 100184 10.1016/j.mtchem.2019.08.003.

[ref27] Cheng F.; Jakle F. Boron-containing polymers as versatile building blocks for functional nanostructured materials. Polym. Chem. 2011, 2, 2122–2132. 10.1039/c1py00123j.

[ref28] Jacobs B. P.; Brantley J. N. Exploring Combinatorial Approaches to Polymer Diversification. Macromolecules 2020, 53, 9287–9293. 10.1021/acs.macromol.0c01538.

[ref29] Jakle F. Advances in the Synthesis of Organoborane Polymers for Optical, Electronic, and Sensory Applications. Chem. Rev. 2010, 110, 3985–4022. 10.1021/cr100026f. 20536123

[ref30] Brooks W. L. A.; Sumerlin B. S. Synthesis and Applications of Boronic Acid-Containing Polymers: From Materials to Medicine. Chem. Rev. 2016, 116, 1375–1397. 10.1021/acs.chemrev.5b00300. 26367140

[ref31] Sutthasupa S.; Shiotsuki M.; Sanda F. Recent advances in ring-opening metathesis polymerization, and application to synthesis of functional materials. Polym. J. 2010, 42, 905–915. 10.1038/pj.2010.94.

[ref32] Hall D. G.; Rybak T.; Verdelet T. Multicomponent Hetero-[4 + 2] Cycloaddition/Allylboration Reaction: From Natural Product Synthesis to Drug Discovery. Acc. Chem. Res. 2016, 49, 2489–2500. 10.1021/acs.accounts.6b00403. 27753496

[ref33] Ni D. S.; Witherspoon B. P.; Zhang H.; Zhou C.; Houk K. N.; Brown M. K. Stereoselective [4 + 2]-Cycloaddition with Chiral Alkenylboranes. Angew. Chem., Int. Ed. 2020, 59, 11432–11439. 10.1002/anie.202000652. 32390259

[ref34] Pyziak J.; Walkowiak J.; Marciniec B. Recent Advances in Boron-Substituted 1,3-Dienes Chemistry: Synthesis and Application. Chem. - Eur. J. 2017, 23, 3502–3541. 10.1002/chem.201602124. 28297134

[ref35] Silva M. A.; Pellegrinet S. C.; Goodman J. M. Diels-Alder reactions of vinylboranes: A computational study on the boron substituent effects. ARKIVOC 2003, 2003, 556–565. 10.3998/ark.5550190.0004.a51.

[ref36] Sarotti A. M.; Pisano P. L.; Pellegrinet S. C. A facile microwave-assisted Diels-Alder reaction of vinylboronates. Org. Biomol. Chem. 2010, 8, 5069–5073. 10.1039/c0ob00020e. 20835449

[ref37] Fasano V.; McFord A. W.; Butts C. P.; Collins B. S. L.; Fey N.; Alder R. W.; Aggarwal V. K. How Big is the Pinacol Boronic Ester as a Substituent?. Angew. Chem., Int. Ed. 2020, 59, 22403–22407. 10.1002/anie.202007776. 32866342

[ref38] Vallejos M. M.; Grimblat N.; Pellegrinet S. C. Reactivity and Selectivity of Boron-Substituted Alkenes in the Diels-Alder Reaction with Cyclopentadiene. A Study of the Electron Charge Density and Its Laplacian. J. Phys. Chem. A 2014, 118, 5559–5570. 10.1021/jp504972r. 24983836

[ref39] Belov D. S.; Mathivathanan L.; Beazley M. J.; Martin W. B.; Bukhryakov K. V. Stereospecific Ring-Opening Metathesis Polymerization of Norbornene Catalyzed by Iron Complexes. Angew. Chem., Int. Ed. 2021, 60, 2934–2938. 10.1002/anie.202011150. 33125813

[ref40] Chirlian L. E.; Francl M. M. Atomic Charges Derived from Electrostatic Potentials - a Detailed Study. J. Comput. Chem. 1987, 8, 894–905. 10.1002/jcc.540080616.

[ref41] Zhao Y.; Truhlar D. G. The M06 suite of density functionals for main group thermochemistry, thermochemical kinetics, noncovalent interactions, excited states, and transition elements: two new functionals and systematic testing of four M06-class functionals and 12 other functionals. Theor. Chem. Acc. 2008, 120, 215–241. 10.1007/s00214-007-0310-x.

[ref42] Legault C. Y. C. CYLview, 1.0b; Université de Sherbrooke, 2018; available online: http://www.cylview.org (accessed on Feb 11 2021). Most of the hydrogen atoms are removed for clarity.

[ref43] Francois B.; Eberlin L.; Berree F.; Whiting A.; Carboni B. Generating Skeletal Diversity and Complexity from Boron-Substituted 1,3-Dienes and Enophiles. Eur. J. Org. Chem. 2020, 2020, 3282–3293. 10.1002/ejoc.202000330.

[ref44] Li J. B.; Wang H. N.; Qiu Z. H.; Huang C. Y.; Li C. J. Metal-Free Direct Deoxygenative Borylation of Aldehydes and Ketones. J. Am. Chem. Soc. 2020, 142, 13011–13020. 10.1021/jacs.0c03813. 32597177

[ref45] Lee H.; Lee Y.; Cho S. H. Palladium-Catalyzed Chemoselective Negishi Cross-Coupling of Bis[(pinacolato)boryl]methylzinc Halides with Aryl (Pseudo)Halides. Org. Lett. 2019, 21, 5912–5916. 10.1021/acs.orglett.9b02050. 31329446

[ref46] Aggarwal V. K.; Ali A.; Coogan M. P. The development and use of ketene equivalents in [4 + 2] cycloadditions for organic synthesis. Tetrahedron 1999, 55, 293–312. 10.1016/S0040-4020(98)00924-7.

[ref47] Ng E. W. H.; Low K. H.; Chiu P. Synthesis and Applications of Unquaternized C-Bound Boron Enolates. J. Am. Chem. Soc. 2018, 140, 3537–3541. 10.1021/jacs.8b00614. 29481069

[ref48] Cuenca A. B.; Cid J.; Garcia-Lopez D.; Carbo J. J.; Fernandez E. Unsymmetrical 1,1-diborated multisubstituted sp(3)-carbons formed via a metal-free concerted-asynchronous mechanism (vol 13, pg 9659, 2015). Org. Biomol. Chem. 2015, 13, 11772–11772. 10.1039/C5OB90195B. 26264986

[ref49] Lee J. C. H.; McDonald R.; Hall D. G. Enantioselective preparation and chemoselective cross-coupling of 1,1-diboron compounds. Nat. Chem. 2011, 3, 894–899. 10.1038/nchem.1150. 22024887

[ref50] Kumar N.; Reddy R. R.; Masarwa A. Stereoselective Desymmetrization of gem-Diborylalkanes by “Trifluorination. Chem. - Eur. J. 2019, 25, 8008–8012. 10.1002/chem.201901267. 30964216

[ref51] Novoa S.; Paquette J. A.; Barbon S. M.; Maar R. R.; Gilroy J. B. Side-chain boron difluoride formazanate polymers via ring-opening metathesis polymerization. J. Mater. Chem. C 2016, 4, 3987–3994. 10.1039/C5TC03287C.

[ref52] Morrill C.; Grubbs R. H. Synthesis of functionalized vinyl boronates via ruthenium-catalyzed olefin cross-metathesis and subsequent conversion to vinyl halides. J. Org. Chem. 2003, 68, 6031–6034. 10.1021/jo0345345. 12868943

[ref53] Vidal F.; McQuade J.; Lalancette R.; Jakle F. ROMP-Boranes as Moisture-Tolerant and Recyclable Lewis Acid Organocatalysts. J. Am. Chem. Soc. 2020, 142, 14427–14431. 10.1021/jacs.0c05454. 32787237

[ref54] Delaude L.; Demonceau A.; Noels A. F. Probing the stereoselectivity of the ruthenium-catalyzed ring-opening metathesis polymerization of norbornene and norbornadiene diesters. Macromolecules 2003, 36, 1446–1456. 10.1021/ma021315x.

[ref55] AlSamak B.; AmirEbrahimi V.; Carvill A. G.; Hamilton J. G.; Rooney J. J. Determination of the tacticity of ring-opened metathesis polymers of norbornene and norbornadiene by C-13 NMR spectroscopy of their hydrogenated derivatives. Polym. Int. 1996, 41, 85–92. 10.1002/(SICI)1097-0126(199609)41:1<85::AID-PI582>3.0.CO;2-Y.

[ref56] Li X. Y.; Hall D. G. Stereodivergent Asymmetric Synthesis of alpha, beta-Disubstituted beta-Aminoalkylboronic Acid Derivatives via Group-Selective Protodeboronation Enabling Access to the Elusive Anti Isomer. J. Am. Chem. Soc. 2020, 142, 9063–9069. 10.1021/jacs.0c03207. 32320234

