# Supplementary material for: Stereoselective Diels–Alder Reactions of gem-Diborylalkenes: Toward the Synthesis of gem-Diboron-Based Polymers
Source: J Am Chem Soc. 2021 Apr 14;143(16):6211–20. doi: 10.1021/jacs.1c01471 (PMC8488944; doi:10.1021/jacs.1c01471)

Supplementary information

Stereoselective Diels-Alder Reactions of *gem*-Diborylalkenes: Toward the Synthesis of *gem*-Diboron-Based Polymers

Nadim Eghbarieh,[1#] Nicole Hanania,[1#] Alon Zamir,[1,2] Molhm Nassir,[1] Tamar Stein,[1,2]* and Ahmad Masarwa[1]*

[1]Institute of Chemistry, The Hebrew University of Jerusalem, Jerusalem 9190401 (Israel)

[2]Fritz Haber Center for Molecular Dynamics Research, Institute of Chemistry, The Hebrew University of Jerusalem, Jerusalem 9190401 (Israel).

[#]These authors contributed equally to the work.

*Corresponding authors: E-mail: tamar.stein@mail.huji.ac.il ; Ahmad.Masarwa1@mail.huji.ac.il.

Table of Contents

1. Material, Methods, and General Remarks. S3

2. Procedure and characterization for starting materials **2** and **3**. S4

3. Procedure and characterization data of D.A. reaction products **4** and **5**. S7

4. Procedure and characterization data for oxidation reaction products **4O**. S33

5. Procedure and characterization data for triflourination reaction products **6**. S38

6. Procedure and characterization data for the synthesis of **5f-*exo***. S43

7. Procedures and data for polymers **poly-7** to **poly-14**. S45

8. Computational Details. S65

9. NMR spectra Reprint. S91

10. X-Ray Crystallography Data. S165

11. Diastereomeric and regioisomeric ratios determination by NMR. S171

12. NOESY NMR analysis. S177

1. Material, Methods, and General Remarks

Unless otherwise stated, reactions were performed in oven-dried glassware fitted with rubber septa under inert atmosphere and were stirred with teflon-coated magnetic stirring bars. Liquid reagents and solvents were transferred *via* syringe using standard schlenk techniques. Solvents Toluene, methanol and acetonitrile were used as commercial grade and used as received with no drying. tetrahydrofuran (THF), diethyl ether (Et_2O), and dichloromethane (CH_2Cl_2) were used from a solvent purification system. All other reagents were used as received unless otherwise noted. Dienes **3** were all purchased except **3l** and ***E*-3k** that were synthesized according to the literature.[1-2] Thin layer chromatography (TLC) was performed using silica gel 60 F-254 precoated plates (0.25 mm) and visualized by UV irradiation λ=232 nm, CAM stain, $KMnO_4$ stain, and other stains. Silica gel of particle size 230-400 mesh was used for flash chromatography, Flash chromatography (FC) was performed using CombiFlash, with SiO2 columns. ^{1}H and ^{13}C NMR spectra were recorded on 400, 500 MHz spectrometers with ^{13}C operating frequencies of 101, 126 MHz, ^{11}B operating frequencies of 128, 160 MHz, ^{19}F operating frequencies of 376, 471 MHz respectively. ^{1}H and ^{13}C NMR spectra were referenced to TMS as an internal standard with a deuterated solvent unless otherwise stated. X-Ray structures were visualized with CYLview[3], most of the hydrogen atoms are removed for clarity. Chemical shifts (δ) are reported in ppm relative to the residual solvents ($CDCl_3$) signal (δ = 7.26 for ^{1}H NMR and δ = 77.0 for ^{13}C NMR) and (DMSO-d_6) signal (δ = 2.50 (ppm) for ^{1}H NMR and δ = 39.5 (septet) for ^{13}C NMR). Data for ^{1}H NMR spectra are reported as follows: chemical shift (multiplicity, coupling constants, and number of hydrogen). Abbreviations are as follows: s (singlet), d (doublet), t (triplet), q (quartet), p (pentate), m (multiplet), brs (broad singlet). Melting point were determined on a melting point apparatus. High-Resolution Mass Spectrometry (HRMS) using methanol or acetonitrile as solvent. The molecular weight of the polymer was determined by gel permeation chromatography (GPC) using polystyrene as a standard and THF as eluent. Thermogravimetric analysis (TGA) was performed on Mettler Toledo TG 50 analyzer. Measurements were carried out at temperature range that extended from 25–950 ˚C at a heating rate of 10 ˚C min^{-1} under N_2.

2. Procedures and characterizations for starting materials 2 and 3.

Notes: (1) all dienes **3a-j** were purchased as commercially available reagents except for **3I** and ***E*-3k** that were prepared according to a literature reported procedure. (2) Dienophile **2** was prepared according to a literature reported procedure.

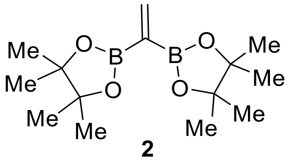

2

***2,2'-(ethene-1,1-diyl)bis(4,4,5,5-tetramethyl-1,3,2-dioxaborolane)*(2):**[4]

A solution of Bis(pinacolato)diboron-(B_2pin_2) (2.5 g, 5.00 mmol) was dissolved in mixture of dry Ether and pentane (30 :7.5) ml under inert conditions, followed by addition of vinyl bromide (1.0 M in THF) (15 mL, 15 mmol), the reaction mixture was cooled to -110 °C and serried for 10 min. next freshly prepared LiTMP solution was added dropwise. The reaction mixture was kept at -110 °C for a further 30 minutes. After 30 min the residue was heated gradually to rt and stirred for 1 h. then the reaction mixture was quenched via solution of 10 % $NaHSO_4$ (25 mL) and H_2O (25 mL) and stirred for 5 min. the residue extracted with Et_2O (3 × 80 mL). then the organic layer washed with brine solution followed by addition of $MgSO_4$, finally filtered and concentrated in vacuo. The crude product was purified by silica gel column chromatography using hexane: ethyl acetate as an eluent. isolated **2** in 55% yield as light yellowish solid.

R_f = 0.34 (10% EtOAc in hexane);

^{1}H NMR (400 MHz, $CDCl_3$) δ: 6.58 (s, 2H), 1.26 (s, 24H).

^{11}B NMR (128 MHz, $CDCl_3$) δ: 30.6.

The spectral data matched those reported in the literature.[4]

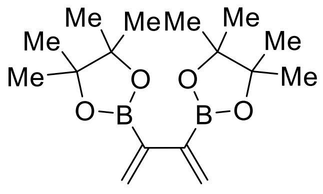

3l

***2,2'-(buta-1,3-diene-2,3-diyl)bis(4,4,5,5-tetramethyl-1,3,2-dioxaborolane)* (3l):**[1]

Freshly prepared LiTMP solution was added to a solution of vinyl bromide (1.00 M, 2.5 mmol) in THF at −110 °C, followed by slowly addition of Bis(pinacolato)diboron-(B_2pin_2), 1.25 mmol) in THF (12 mL). The resulting mixture was allowed to warm gradually to room temperature and stirred for 12 h. The reaction was quenched with 1ml of saturated aqueous NH_4Cl, and the mixture was diluted with diethyl ether (10 mL) and water (10 mL). The organic layer was separated, dried over anhydrous magnesium sulfate, filtered, and concentrated in vacuo to form a colorless solid, which was purified by column chromatography to give **3l** (60 %) as a white solid and **2** (30 %) yields as a light yellowish solid.

R_f = 0.47 (10% EtOAc in hexane);

^{1}H NMR (400 MHz, $CDCl_3$) δ: 5.93 (d, *J* = 3.40 Hz, 2H), 5.85 (d, *J* = 3.68 Hz, 2H), 1.27 (s, 24H).

^{13}C NMR (101 MHz, $CDCl_3$) δ: 130.73, 83.62, 24.93 (C-B) Carbon signal not observed due to quadrupolar relaxation.

^{11}B NMR (128 MHz, $CDCl_3$) δ: 30.1.

The spectral data matched those reported in the literature.[1]

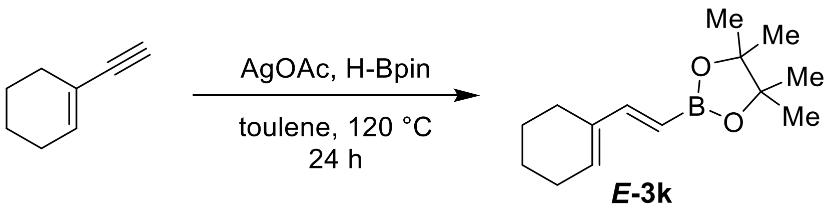

***E*-3k**

***(E)-2-(2-(Cyclohex-1-en-1-yl)vinyl)-4,4,5,5-tetramethyl-1,3,2-dioxaborolane* (*E*-3k):**[2]

Prepared according to a literature reported procedure with slight modification.

In a glove box, a flame dried pressure tube (30 mL) with a magnetic stirrer bar, was charged with AgOAc (0.2 mmol), toluene (4 mL) and Ethynylcyclohexene (2 mmol). Then H-Bpin (3 mmol) was added drop wise over one minute. The resulting mixture was stirred at 120 °C for 24 h. After cooling to room temperature, the reaction mixture was diluted with 5 mL of Et_2O and filtered through a plug of celite, followed by washing with 10–20 mL of Et_2O. The combined residue was concentrated under reduced pressure, and then the resulting crude product was purified by column chromatography on silica gel, the product ***E*-3k**, was obtained as a colorless oil with (70 %) yield.

Note: compound becomes crystal when kept in -30 °C.

R_f = 0.57 (10% EtOAc in hexane).

^{1}H NMR (400 MHz, $CDCl_3$) δ: 6.93 (d, *J* = 18.23 Hz, 1H), 5.86 (s, 1H), 5.33 (dd, *J* = 18.22, 0.42 Hz, 1H), 2.06-2.04 (m, 4H), 1.60-1.47 (m, 4H), 1.17 (s, 12H).

^{13}C NMR (101 MHz, $CDCl_3$) δ: 153.12, 137.06, 133.99, 82.82, 26.09, 24.69, 23.70, 22.37, 22.29. (C-B) Carbon signal not observed due to quadrupolar relaxation.

^{11}B NMR (128 MHz, $CDCl_3$) δ: 30.0.

The spectral data matched those reported in the literature.[2]

3. Procedure and characterization data for the D.A. reaction products 4 and 5.

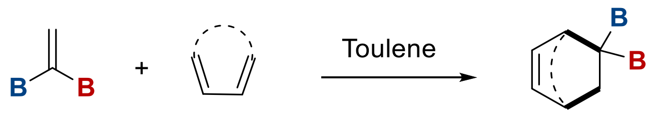

2 **3** **4/5**

B = Bpin
B = Bpin, Bdan

Vinyl gem-diboronic ester (**2**) (1 eq, 0.3 mmol) was inserted into pressure tube and dissolved with toluene (3 ml), under inert conditions (glovebox), followed by addition of the Diene (**3**) (3 eq, 0.9 mmol). the tube was sealed and heated to the stated time and temperature, monitored by TLC, after completion of the reaction the residue was concentrated under reduced pressure. The crude material was purified by short silica gel chromatography with EtOAc/Hexane affording the cycloaddition product (**4**,**5**).

Table S1- Optimization of time and temperature:

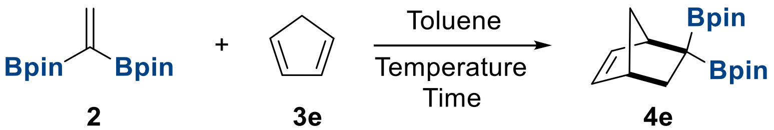

2 **3e** **4e**

Entry	Temperature °C	Time (h)	% Yield of 3[a,b]
1.	180	24	80[c]
2.	180	12	85[c]
3.	100	12	70
4.	180	3	92
5.	rt	24	90
6.	rt	24 Opened to Air	77[c]

[a] Reactions were carried out with 0.20 mmol of **2** and 0.60 mmol of Cyclopentadiene **3e** along with 2 ml of Toluene under inert conditions. [b] Isolated yield. [c] not a clean reaction.

Table S2- Optimization of solvent:

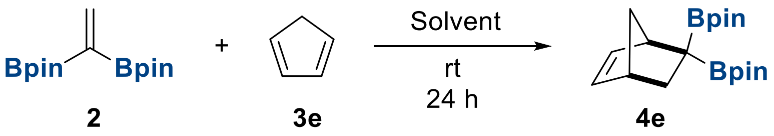

Entry	Solvent	Time (h)	% Yield of 3[a,b]
1.	Toluene	24	90
2.	Ethanol	24	80
3.	Dichloromethane	24	50
4.	Water	24	80[c]
5.	Ethyl acetate	24	65
6.	Cyclohexane	24	50
7.	CH_3CN	24	traces
8.	Solvent Free	24	80[c]

[a] Reactions were carried out with 0.20 mmol of **2** and 0.60 mmol of Cyclopentadiene **3e** along with 2 ml of Toluene under inert conditions. [b] Isolated yield. [c] not a clean reaction.

Table S3- scope of the D.A. reaction of 2 with 3:

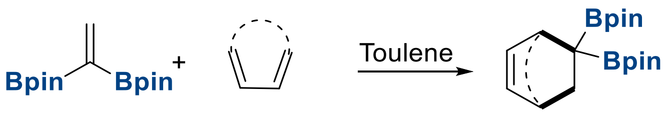

2 3 4

Entry	Diene	Time	Temperature °C	Yield (%)^a	Isomeric ratio	Products
1.	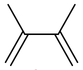 3a	12 h	180	92	--	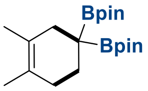 4a
2.	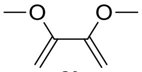 3b	12 h	180	72	--	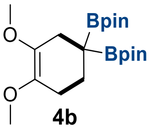 4b
3.	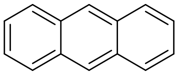 3c	24 h	185	65	--	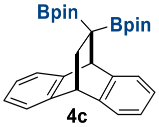 4c
4.	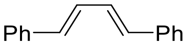 3d	12 h	180	75	99:01^b	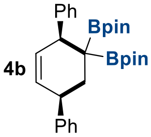 4b
5.	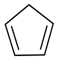 3e	24 h	rt	90	--	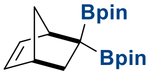 4e
6.	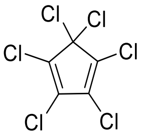 3e-6Cl	12 h	185	81	--	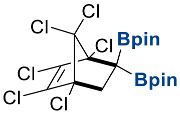 4e-6Cl
7.	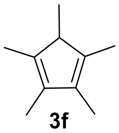 3f	30 h	rt	75	99:01^b	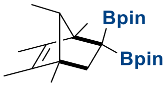 4f
8.	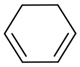 3g	12 h	180	55	--	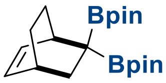 4e

**9.**	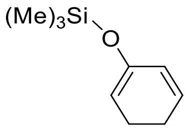 **3g**	**12 h**	**150**	**74**	**99:01**[c]	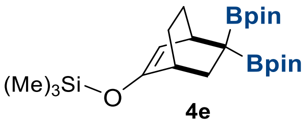 **4e**
**10.**	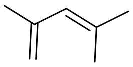 **3h**	**24 h**	**180**	**51**	**86:14**[c]	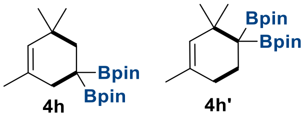 **4h** **4h'**
**11.**	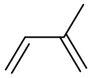 **3i**	**24 h**	**180**	**85**	**86:14**[c]	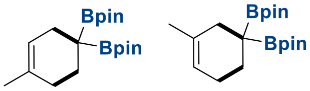 **4i** **4i'**
**12.**	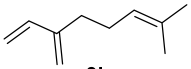 **3j**	**24 h**	**180**	**55**	**87:13**[c]	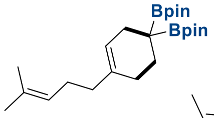 **4j** 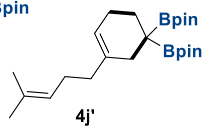 **4j'**
**13.**	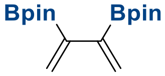 **3l**	**12 h**	**180**	**75**	**--**	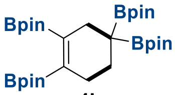 **4b**
**14.**	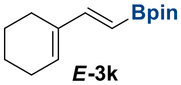 ***E*-3k**	**24 h**	**180**	**87**	**99:01**[b] **99:01**[c]	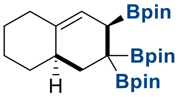

[a] Isolated yield. [b] Ratio of *dr* = *diastereomeric ratio*. [c] Ratio of *r.r.* = *regioisomeric ratio*.

Table S4- scope of the D.A. reaction of 2' with 3:

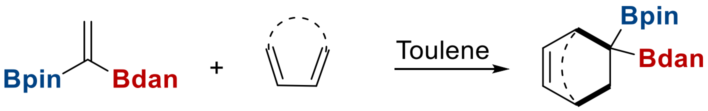

2' 3 4

Entry	Diene	Time	Temperature °C	Yield (%)[a]	Isomeric ratio	Products
1.	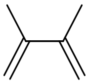 3a	12 h	180	75	--	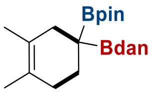 5a
2.	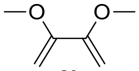 3b	12 h	180	85	--	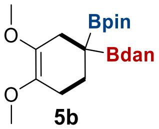 5b
3.	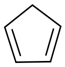 3e	24 h	rt	80	92:08[b]	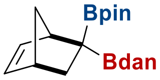 5c
4.	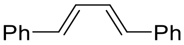 3d	12 h	180	82	60:40[b]	5d 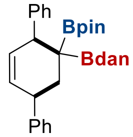
5.	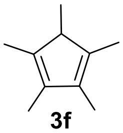 3f	24 h	rt	85	99:01:01[b]	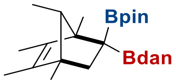 5f
6.	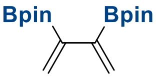 3l	12 h	180	92	--	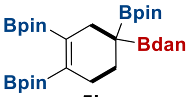 5l
7.	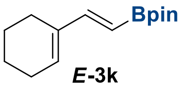 *E*-3k	24 h	180	75	98:01:01[b]<br>99:01[c]	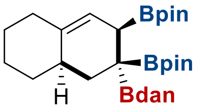 5k 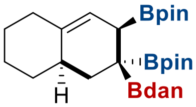 5k'

[a] Isolated yield. [b] Ratio of *dr* = *diastereomeric ratio*. [c] Ratio of *r.r.* = *regioisomeric ratio*.

Unsuccessful example of D.A. reaction with dienophiles 2 and 2’:

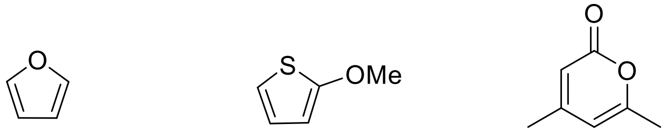

Unsuccessful example of D.A. reaction with 3a and 3e as dienes:

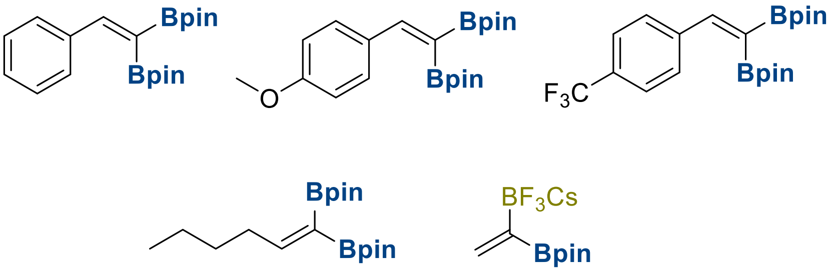

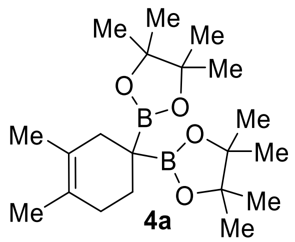

4a

2,2'-(3,4-dimethylcyclohex-3-ene-1,1-diyl)bis(4,4,5,5-tetramethyl-1,3,2-dioxaborolane) **(4a):** General procedure (A) followed, isolated **4a** in (92 %) yield as a white solid, mp 44-49 °C, R_f = 0.42 (10% EtOAc in hexane).

^{1}H NMR (400 MHz, $CDCl_3$) δ: 1.98 (s, 2H), 1.89 (m, 2H), 1.65 (t, *J* = 6.3 Hz, 2H), 1.50 (s, 3H), 1.43 (s, 3H), 1.075 (d, *J* = 4.7 Hz, 24H).

^{13}C NMR (126 MHz, $CDCl_3$) δ: 127.63, 124.90, 82.93, 34.88, 30.99, 26.39, 24.71, 24.63, 19.49, 19.05. (C-B) Carbon signal not observed due to quadrupolar relaxation.

^{11}B NMR (128 MHz, $CDCl_3$) δ: 34.7.

HRMS (ESI) Calcd for $[C_{20}H_{36}B_2O_4+[H]]^+$ $[M+H]^+$: m/z 363.2882, found 363.2886.

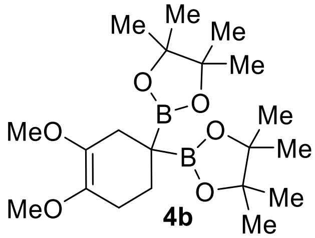

4b

2,2'-(3,4-dimethoxycyclohex-3-ene-1,1-diyl)bis(4,4,5,5-tetramethyl-1,3,2-dioxaborolane) **(4b):**General procedure (A) followed, isolated **4b** in (72 %) yield as white solid, mp 146-148 °C, R_f = 0.323 (20% EtOAc in hexane).

^{1}H NMR (400 MHz, CD_3CN) δ: 3.50 (s, 3H), 3.47 (s, 3H), 2.17 (s, 2H), 2.10 (m, 2H), 1.68 (t, *J* = 6.1 Hz, 2H), 1.19 (d, *J* = 3.1 Hz, 24H).

^{13}C NMR (126 MHz, CD_3CN) δ: 140.87, 139.81, 84.15, 57.51, 29.36, 26.51, 25.95, 24.97. (C-B) Carbon signal not observed due to quadrupolar relaxation.

^{11}B NMR (128 MHz, CD_3CN) δ: 34.7.

HRMS (ESI) Calcd for $[C_{20}H_{36}B_2O_6+H]^+$ $[M+H]^+$: m/z 395.2778, found 395.2744 .

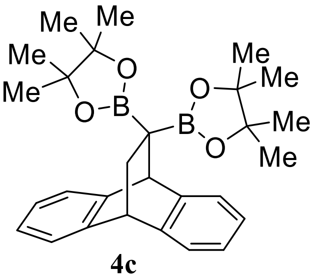

4c

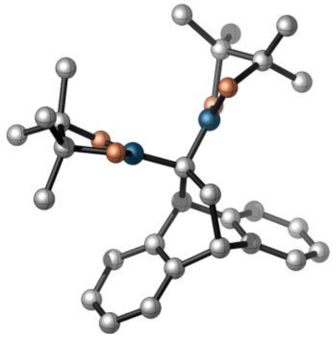

X-ray structure of 4c

2,2'-(9,10-dihydro-9,10-ethanoanthracene-11,11-diyl)bis(4,4,5,5-tetramethyl-1,3,2-dioxaborolane) **(4c):** General procedure (A) followed, isolated **4c** in (65 %) yield as white solid, mp 166-170 °C, R_f = 0.34 (10% EtOAc in hexane).

^{1}H NMR (400 MHz, $CDCl_3$) δ: 7.23-7.21 (m, 2H), 7.16-7.14 (m, 2H), 6.94-6.92 (m, 4H), 4.52 (s, 1H), 4.23 (t, J = 2.6 Hz, 1H), 2.04 (d, J = 2.7 Hz, 2H), 0.99 (s, 12H), 0.88 (s, 12H).

^{13}C NMR (101 MHz, $CDCl_3$) δ: 144.41, 144.00, 125.35, 124.83, 124.21, 123.21, 83.49, 47.28, 45.16, 33.27, 31.07, 25.06, 24.88, 24.67, 24.47. (C-B) Carbon signal not observed due to quadrupolar relaxation.

^{11}B NMR (128 MHz, $CDCl_3$) δ: 34.1.

HRMS (ESI) Calcd for $[C_{28}H_{36}B_2O_4+H]^+$ $[M+H]^+$: m/z 459.2882, found 459.2901.

Note: the structure of **4c** was also confirmed by X-ray crystallographic analysis (see page S165)

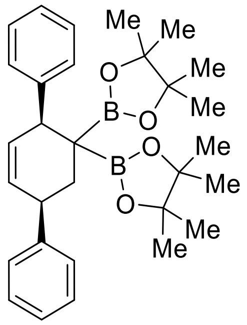

4d

2,2'-(2,5-diphenylcyclohex-3-ene-1,1-diyl)bis(4,4,5,5-tetramethyl-1,3,2-dioxaborolane) **(4d):** General procedure (A) followed, isolated **4d** in (75 %) yield as white solid, mp 190-193 °C, R_f = 0.36 (10% EtOAc in hexane).

^{1}H NMR (400 MHz, $CDCl_3$) δ: 7.47-7.45 (m, 2H), 7.35-7.30 (m, 4H), 7.25-7.13 (m, 4H), 5.95 (dq, *J* = 10.1, 2.3 Hz, 1H), 5.93 (dq, *J* = 10.1, 1.3 Hz, 1H), 3.93 (t, *J*= 2.2 Hz, 1H), 3.51-3.46 (m, 1H), 2.21 (dd, *J* =13.8, 5.8 Hz, 1H), 1.93 (dd, *J* = 13.8, 11.4 Hz, 1H), 1.33 (s, 6H), 1.29 (s, 6H), 1.00 (s, 6H), 0.82 (s, 6H).

^{13}C NMR (101 MHz, $CDCl_3$) δ: 146.97, 144.61, 132.52, 130.29, 130.11, 128.48, 127.82, 127.73, 126.29, 126.08, 83.61, 83.07, 42.60, 41.42, 31.67, 25.13, 24.89, 24.38. (C-B) Carbon signal not observed due to quadrupolar relaxation.

^{11}B NMR (128 MHz, $CDCl_3$) δ: 34.1.

HRMS (ESI) Calcd for $[C_{30}H_{40}B_2O_4+H]^+$ $[M+H]^+$: m/z 487.3196, found 487.3221.

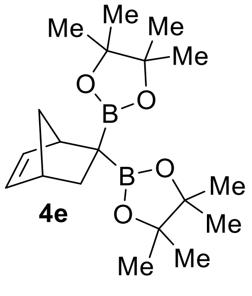

4e

2,2'-((1S,4S)-bicyclo[2.2.1]hept-5-ene-2,2-diyl)bis(4,4,5,5-tetramethyl-1,3,2-dioxaborolane) **(4e):** General procedure (A) followed, isolated **4e** in (90 %) yield as white solid, mp 62-65 °C, R_f = 0.4 (10% EtOAc in hexane).

^{1}**H NMR** (400 MHz, $CDCl_3$) δ: 6.14 (dd, *J* = 5.6, 2.7 Hz, 1H), 5.95 (dd, *J* = 5.6, 2.4 Hz, 1H), 3.13 (s, 1H), 2.86 (s, 1H), 2.00 (dd, *J* = 11.0, 3.7 Hz, 1H), 1.39 (dd, *J* = 10.5, 1.5 Hz, 1H), 1.26-1.24 (m, 1H), 1.18 (dd, *J* = 23.0, 3.6 Hz, 24H), 1.10 (d, *J* = 8.0 Hz, 1H).

^{13}**C NMR** (126 MHz, $CDCl_3$) δ: 136.93, 134.90, 83.05, 49.83, 46.56, 42.90, 31.67, 24.74, 24.72, 24.70. (C-B) Carbon signal not observed due to quadrupolar relaxation.

^{11}**B NMR** (128 MHz, $CDCl_3$) δ: 34.5.

HRMS (ESI) Calcd for $[C_{19}H_{32}B_2O_4+H]^+$ $[M+H]^+$: m/z 347.2556, found 347.2574.

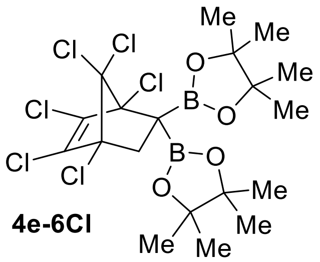

4e-6Cl

2,2'-((1R,4R)-1,4,5,6,7,7-hexachlorobicyclo[2.2.1]hept-5-ene-2,2-diyl)bis(4,4,5,5-tetramethyl-1,3,2-dioxaborolane) **(4e-6Cl):** General procedure (A) followed, isolated **4e-6Cl** in (81 %) yield as brownish oil, R_f = 0.46 (10% EtOAc in hexane).

^{1}**H NMR** (400 MHz, $CDCl_3$) δ: 2.90 (d, *J* = 12.1 Hz, 1H), 2.56 (d, *J* = 12.1 Hz, 1H), 1.25 (s, 6H), 1.24 (s, 6H), 1.23 (s, 6H), 1.21 (s, 6H).

^{13}**C NMR** (101 MHz, $CDCl_3$) δ: 134.36, 130.95, 104.38, 84.89, 84.29, 83.20, 79.33, 40.91, 25.40, 25.03, 24.66, 24.63. (C-B) Carbon signal not observed due to quadrupolar relaxation.

^{11}**B NMR** (128 MHz, $CDCl_3$) δ: 32.4.

HRMS (ESI) Calcd for $[C_{19}H_{26}B_2Cl_6O_4+H]^+$ $[M+H]^+$: m/z 553.0293, found 553.0214.

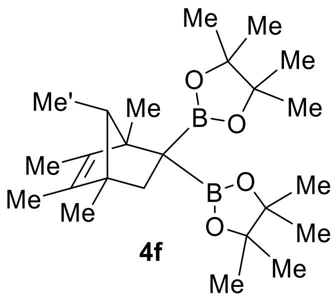

4f

***2,2'-(1,4,5,6,7-pentamethylbicyclo[2.2.1]hept-5-ene-2,2-diyl)bis(4,4,5,5-tetramethyl-1,3,2-dioxaborolane)* (4f):**

General procedure (A) followed, with dr *(syn:anti of Me') = 99:01*, isolated **4f** in (75 %) yield as white solid as a single diastereomer, mp 120-121 °C (decomposed), R_f = 0.5 (10% EtOAc in hexane).

^{1}H NMR (400 MHz, $CDCl_3$) δ: 1.77(d, *J* = 10.7 Hz, 1H), 1.61 (d, *J* = 0.8 Hz, 3H), 1.52 (d, *J* = 10.9 Hz, 1H), 1.49 (q, *J* = 6.4 Hz, 1H), 1.44 (d, *J* = 1.1, 3H), 1.21 (s, 3H), 1.20 (d, *J* = 0.9 Hz, 12H), 1.18 (s, 6H), 1.12 (s, 6H), 1.01 (s, 3H), 0.46 (d, *J* = 6.3 Hz, 3H).

^{13}C NMR (101 MHz, $CDCl_3$) δ: 136.87, 133.83, 82.66, 82.49, 71.96, 60.92, 58.11, 53.03, 42.12, 25.50, 24.97, 24.87, 24.22, 15.95, 14.59, 11.85, 9.62, 8.59. (C-B) Carbon signal not observed due to quadrupolar relaxation.

^{11}B NMR (128 MHz, $CDCl_3$) δ: 33.9.

HRMS (ESI) Calcd for $[C_{24}H_{42}B_2O_4+H]^+$ $[M+H]^+$: m/z 417.3350, found 417.3381.

Notes: (1) Upon heating to 185 °C the diastereomeric ratio dr *(syn:anti of Me') = 5:1.* (2) the relative configuration of **4f** was determined by NOESY NMR (see page S177)**.**

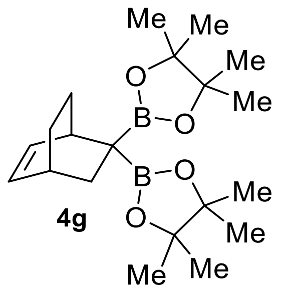

***2,2'-((1S,4R)-bicyclo[2.2.2]oct-5-ene-2,2-diyl)bis(4,4,5,5-tetramethyl-1,3,2-dioxaborolane)* (4g):** General procedure (A) followed, isolated **4g** in (55 %) yield as white solid, mp 104-105 °C, R_f = 0.45 (10% EtOAc in hexane).

^{1}H NMR (400 MHz, $CDCl_3$) δ: 6.38 (t, *J* = 7.1 Hz, 1H), 6.16 (t, *J* = 7.5 Hz, 1H), 2.83-2.80 (m, 1H), 2.51-2.48 (m, 1H), 1.92 (dd, *J* = 11.8, 2.2 Hz, 1H), 1.69-1.61 (m, 3H), 1.51-1.45 (m, 1H), 1.28 (d, *J* = 2.1 Hz, 1H), 1.23 (s, 12H), 1.17 (d, *J* = 2.2 Hz, 12H).

^{13}C NMR (101 MHz, $CDCl_3$) δ: 136.79, 133.63, 83.20, 83.05, 33.27, 31.75, 30.53, 25.78, 24.95, 24.78, 24.76, 24.60. (C-B) Carbon signal not observed due to quadrupolar relaxation.

^{11}B NMR (128 MHz, $CDCl_3$) δ: 34.0.

HRMS (ESI) Calcd for $[C_{20}H_{34}B_2O_4+H]^+$ $[M+H]^+$: m/z 361.2723, found 361.2727.

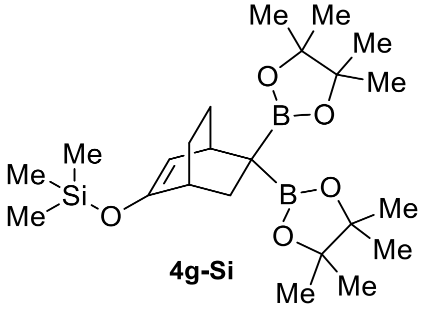

***(((1R,4S)-5,5-bis(4,4,5,5-tetramethyl-1,3,2-dioxaborolan-2-yl)bicyclo[2.2.2]oct-2-en-2-yl)oxy)trimethylsilane* (4g-Si):**

General procedure (A) followed, isolated **4g-Si** in (74 %) yield (*r.r.= 99:01*) as colorless oil, R_f= 0.62 (10% EtOAc in hexane).

^{1}H NMR (400 MHz, $CDCl_3$) δ: 5.23 (dd, *J* = 7.1, 2.3 Hz, 1H), 2.83 (dt, *J* = 7.1, 2.8 Hz, 1H), 2.31 (sext, *J* = 2.6 Hz, 1H), 1.94-1.84 (m, 2H), 1.67-1.60 (m, 2H), 1.49-1.37 (m, 2H), 1.20 (d, *J*

= 1.7 Hz, 12H), 1.17 (d, *J* = 3.1 Hz, 12H), 0.18 (s, 9H).

^{13}C NMR (101 MHz, $CDCl_3$) δ: 156.29, 108.17, 83.21, 83.02, 36.66, 34.15, 32.12, 27.55, 25.64, 25.02, 24.94, 24.73, 24.63, 0.43. (C-B) Carbon signal not observed due to quadrupolar relaxation.

^{11}B NMR (128 MHZ, $CDCl_3$) δ: 34.7

HRMS (ESI) calcd for $[C_{23}H_{42}B_2O_5Si+H]^+$ $[M+H]^+$: m/z 449.3069, found 449.3062.

Note: the relative configuration of **4g-Si** was determined by NOESY NMR (see page S178)**.**

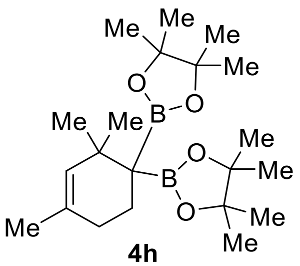

4h

Note: 4h and **4h'** were obtained in regioisomeric ratio of (*r.r. = 86:14)* using **3h** as diene, both isomers were separated by column.

2,2'-(2,2,4-trimethylcyclohex-3-ene-1,1-diyl)bis(4,4,5,5-tetramethyl-1,3,2-dioxaborolane) **(4h):** General procedure (A) followed, isolated **4h** as a single regioisomer in (45 %) yield as colorless oil, R_f = 0.54 (10% EtOAc in hexane).

^{1}H NMR (400 MHz, $CDCl_3$) δ: 5.18-5.16 (m, 1H), 1.94 (t, *J* = 6.2 Hz, 2H), 1.83 (t, *J* = 6.2 Hz, 2H), 1.58 (s, 3H), 1.20 (d, *J* = 2.8 Hz, 24H), 1.13 (s, 6H).

^{13}C NMR (101 MHz, $CDCl_3$) δ: 134.65, 130.65, 82.65, 34.47, 29.29, 28.76, 24.97, 24.77, 24.56, 23.81. (C-B) Carbon signal not observed due to quadrupolar relaxation.

^{11}B NMR (128 MHz, $CDCl_3$) δ: 34.8.

HRMS (ESI) Calcd for $[C_{21}H_{38}B_2O_4+H]^+$ $[M+H]^+$: m/z 377.3037, found 377.3047.

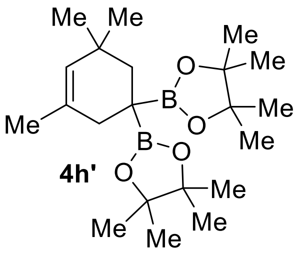

Note: **4h** and **4h'** were obtained in regioisomeric ratio of (*r.r. = 86:14)* using **3h** as diene, both isomers were separated by column.

2,2'-(3,5,5-trimethylcyclohex-3-ene-1,1-diyl)bis(4,4,5,5-tetramethyl-1,3,2-dioxaborolane) **(4h'):** General procedure (A) followed, isolated **4h'** as a single regioisomer in (6 %) yield as colorless oil, R_f = 0.51 (10% EtOAc in hexane).

^{1}H NMR (400 MHz, $CDCl_3$) δ: 5.06 (m, 1H), 2.05 (s, 2H), 1.7 (s, 2H), 1.64 (s, 3H), 1.22 (d, *J* = 3.1 Hz, 24H), 0.94 (s, 6H).

^{13}C NMR (101 MHz, $CDCl_3$) δ: 132.71, 131.54, 83.13, 39.48, 32.20, 31.92, 31.36, 25.02, 24.80, 24.20. (C-B) Carbon signal not observed due to quadrupolar relaxation.

^{11}B NMR (128 MHz, $CDCl_3$) δ: 34.9.

HRMS (ESI) Calcd for $[C_{21}H_{38}B_2O_4+H]^+$ $[M+H]^+$: m/z 377.3037, found 377.3047.

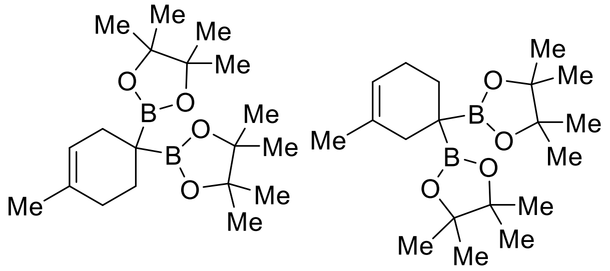

4i **4i'**

Note: **4i** and **4i'** were obtained in regioisomeric ratio of (*r.r. = 86:14)* **3i** as diene, **4i** was separated by column as a single regioisomer.

2,2'-(3-methylcyclohex-3-ene-1,1-diyl)bis(4,4,5,5-tetramethyl-1,3,2-dioxaborolane) **(4i'):**

2,2'-(4-methylcyclohex-3-ene-1,1-diyl)bis(4,4,5,5-tetramethyl-1,3,2-dioxaborolane) **(4i):**

General procedure (A) followed, isolated **4i** and **4i'** as both regioisomer in (85 %) yield as white solid, mp 122-124 °C, R_f = 0.48 (10% EtOAc in hexane).

^{1}H NMR (400 MHz, $CDCl_3$, for **minor regioisomer-4i'**) δ: 5.24-5.22 (m, 1H), 2.10-2.03 (m, 2H), 1.98- 1.87 (m, 2H), 1.65 (t, *J* = 6.1 Hz, 2H), 1.57 (s, 3H), 1.13 (s, 24H).

^{13}C NMR (126 MHz, $CDCl_3$, for **both regioisomer**) δ: 135.60, 133.55, 122.59, 120.56, 82.95, 77.48, 77.16, 76.84, 29.39, 28.45, 25.98, 24.71, 24.66, 23.91. (C-B) Carbon signal not observed due to quadrupolar relaxation.

^{11}B NMR (128 MHz, $CDCl_3$, for **both regioisomer**) δ: 34.8.

HRMS (ESI) Calcd for $[C_{19}H_{34}B_2O_4+H]^+$ $[M+H]^+$: m/z 349.2723, found 349.2725.

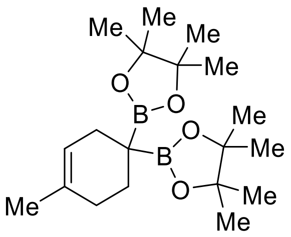

4i

Note: 4i and **4i'** were obtained in regioisomeric ratio of (*r.r. = 86:14)* **3i** as diene, **4i** was separated by column as a single regioisomer.

2,2'-(4-methylcyclohex-3-ene-1,1-diyl)bis(4,4,5,5-tetramethyl-1,3,2-dioxaborolane) **(4i):** General procedure (A) followed, isolated **4i** as a single regioisomer in (63 %) yield as white solid, mp 123-125 °C, R_f = 0.49 (10% EtOAc in hexane).

^{1}H NMR (400 MHz, $CDCl_3$, for **major regioisomer**) δ: 5.50-5.47 (m, 1H), 2.16 (m, 2H), 1.95 (m, 2H), 1.80 (t, *J* = 6.1 Hz, 2H), 1.58 (s, 3H), 1.20 (s, 24H).

^{13}C NMR (126 MHz, $CDCl_3$) δ: 133.65, 122.63, 83.02, 77.41, 29.44, 28.50, 26.04, 24.71, 23.96. (C-B) Carbon signal not observed due to quadrupolar relaxation.

^{11}B NMR (128 MHz, $CDCl_3$) δ: 34.4.

HRMS (ESI) Calcd for $[C_{19}H_{34}B_2O_4+H]^+$ $[M+H]^+$: m/z 349.2723, found 349.2725.

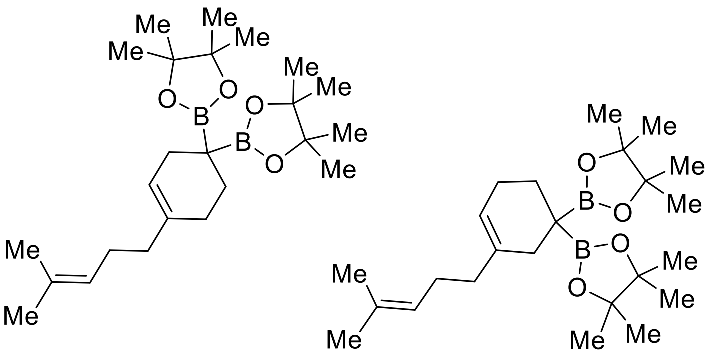

4j **4j'**

Note: 4j and **4j'** were obtained in regioisomeric ratio of (*r.r. = 87:13)* using **3j** as diene.

***2,2'-(4-(4-methylpent-3-en-1-yl)cyclohex-3-ene-1,1-diyl)bis(4,4,5,5-tetramethyl-1,3,2-dioxaborolane)* (4j):**

***2,2'-(3-(4-methylpent-3-en-1-yl)cyclohex-3-ene-1,1-diyl)bis(4,4,5,5-tetramethyl-1,3,2-dioxaborolane)* (4j'):**

General procedure (B) followed, isolated **4j** and **4j'** in (55 %) yield (*r.r. = 85:15)* as colorless oil for both regioisomer, R_f= 0.382 (10% EtOAc in hexane).

^{1}H NMR (400 MHz, $CDCl_3$, for **major regioisomer**) δ: 5.51-5.49 (m, 1H), 5.14-5.07 (m, 1H), 2.20-2.17 (m, 2H), 2.12-1.88 (m, 6H), 1.79 (t, *J* = 6. Hz, 2H), 1.68 (s, 3H), 1.66 (s, 3H), 1.20 (s, 24H).

^{1}H NMR (400 MHz, $CDCl_3$, for **minor regioisomer**) δ: 5.32-5.31 (m, 1H), 5.14-5.07 (m, 1H), 2.12 (m, 2H), 2.10-1.88 (m, 6H), 1.75 (t, *J* = 6.2 Hz, 2H), 1.69 (s, 3H), 1.68 (s, 3H), 1.26 (s, 24H).

^{13}C NMR (126 MHz, $CDCl_3$, for **both regioisomer**) δ: 139.46, 137.30, 131.22, 130.95, 125.13, 124.84, 122.29, 120.60, 83.02, 38.44, 31.41, 28.41, 27.64, 26.80, 25.98, 25.82, 24.77, 24.74, 24.72, 17.79. (C-B) Carbon signal not observed due to quadrupolar relaxation.

^{11}B NMR (128 MHz, $CDCl_3$, for **both regioisomer**) δ: 34.4.

HRMS (ESI) Calcd for $[C_{24}H_{42}B_2O_4+H]^+$ $[M+H]^+$: m/z 417.3350, found 417.3310.

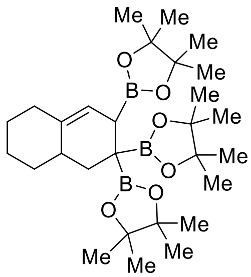

4k

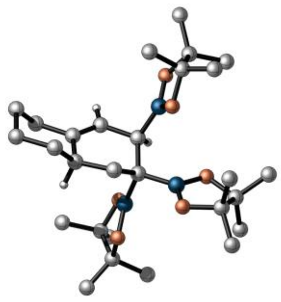

X-ray structure of 4k

***2,2',2''-(1,2,3,5,6,7,8,8a-octahydronaphthalene-2,2,3-triyl)tris(4,4,5,5-tetramethyl-1,3,2-dioxaborolane)*(4k):**

General procedure (A) followed, isolated **4k** in (87 %) yield (*r.r.= 99:01*) as white solid, mp 194-196 °C, R_f = 0.285 (10% EtOAc in hexane).

^{1}H NMR (400 MHz, $CDCl_3$) δ: 5.53 (d, *J* = 6.4 Hz, 1H), 2.19-2.09 (m, 4H), 1.79 (t, *J* = 11.1 Hz, 2H), 1.65 (d, *J* = 11.4 Hz, 1H), 1.53 (q, *J* = 3.3, 12.0 Hz, 1H), 1.29-1.26 (m, 1H), 1.25 (s, 1H), 1.21 (s, 12H), 1.19 (s, 6H), 1.16 (d, *J* = 2.0 Hz, 12H), 1.14 (s, 6H), 0.79 (dq, *J* = 13.1, 3.2 Hz, 1H).

^{13}C NMR (126 MHz, $CDCl_3$) δ: 139.34, 122.30, 82.98, 82.93, 82.84, 36.75, 36.15, 35.86, 32.89, 28.47, 26.76, 25.32, 25.11, 24.91, 24.65, 24.00. (C-B) Carbon signal not observed due to quadrupolar relaxation.

^{11}B NMR (128 MHZ, $CDCl_3$) δ: 34.0.

HRMS (ESI) calcd for $[C_{28}H_{49}B_3O_6+H]^+$ $[M+H]^+$: m/z 515.3895, found 515.3880.

Notes: (1) the structure of **4k** was also confirmed by X-ray crystallographic analysis (see page S166). (2) the relative configuration of **4k** was determined by NOESY NMR (see page S181)**.**

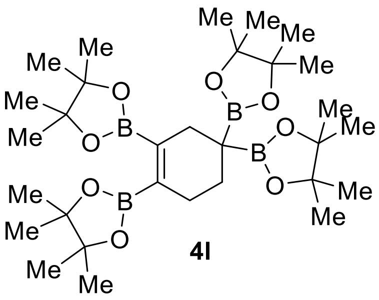

***2,2',2'',2'''-(cyclohex-3-ene-1,1,3,4-tetrayl)tetrakis(4,4,5,5-tetramethyl-1,3,2-dioxaborolane)* (4l):**

General procedure (A) followed, isolated **4l** in (75 %) yield as white solid, mp 175-177 °C, R_f = 0.14 (10% EtOAc in hexane).

^{1}H NMR (500 MHz, $CDCl_3$) δ: 2.35 (t, *J* = 2.3 Hz, 2H), 2.18-2.15 (m, 2H), 1.74 (t, *J* = 6.1 Hz, 2H), 1.26 (s, 12H), 1.24 (s, 12H), 1.18 (s, 24H).

^{13}C NMR (126 MHz, $CDCl_3$) δ: 83.00, 31.08, 27.34, 25.04, 24.94, 24.79, 24.72, 24.39.

(C-B) Carbon signal not observed due to quadrupolar relaxation.

^{11}B NMR (128 MHz, $CDCl_3$) δ: 34.3, 30.3.

HRMS (ESI) Calcd for $[C_{30}H_{54}B_4O_8+H]^+$ $[M+H]^+$: m/z 587.4284, found 587.4284.

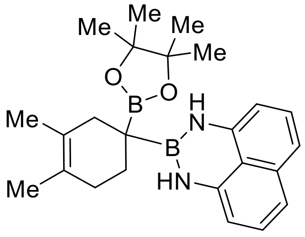

5a

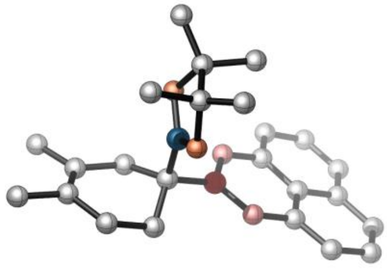

X-ray structure of 5a

***2-(3,4-dimethyl-1-(4,4,5,5-tetramethyl-1,3,2-dioxaborolan-2-yl)cyclohex-3-en-1-yl)-2,3-dihydro-1H-naphtho[1,8-de][1,3,2]diazaborinine* (5a):**

General procedure (A) followed, isolated **5a** in (75 %) yield as white solid, mp 205-208 °C, R_f = 0.42 (10% EtOAc in hexane).

^{1}H NMR (400 MHZ, $CDCl_3$) δ: 7.11 (t, *J* = 7.7 Hz, 2H), 7.01 (d, *J* = 8.8 Hz, 2H), 6.31 (dd, *J* = 7.3, 0.9 Hz, 2H), 5.85 (s, 2H), 2.25 (d, *J* = 16.5 Hz, 1H), 2.14 (d, *J* = 10.2 Hz, 1H), 2.08 (d, *J* = 16.4 Hz, 1H), 2.01-1.94 (m, 2H), 1.72 (s, 3H), 1.69-1.63 (m, 1H), 1.62 (s, 3H), 1.23 (d, *J* = 6.7 Hz, 12H).

^{13}C NMR (101 MHz, $CDCl_3$) δ: 141.53, 136.42, 127.66, 126.7, 125.74, 119.68, 117.29, 105.58, 83.25, 35.58, 31.06, 27.12, 24.77, 24.61, 19.56, 19.12. (C-B) Carbon signal not observed due to quadrupolar relaxation.

^{11}B NMR (128 MHZ, $CDCl_3$) δ: 34.5, 30.9.

HRMS (ESI) Calcd for $[C_{24}H_{32}B_2N_2O_2+H]^+$ $[M+H]^+$: m/z 403.2731, found 403.2721.

Note: the structure of **5a** was also confirmed by X-ray crystallographic analysis (see page S167).

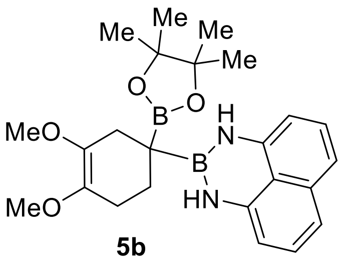

5b

2-(3,4-dimethoxy-1-(4,4,5,5-tetramethyl-1,3,2-dioxaborolan-2-yl)cyclohex-3-en-1-yl)-2,3-dihydro-1H-naphtho[1,8-de][1,3,2]diazaborinine **(5b):**

General procedure (A) followed, isolated **5b** in (85 %) yield as white solid, mp 162-164 °C (decomposed) R_f = 0.31 (20% EtOAc in hexane).

^{1}H NMR (400 MHz, $CDCl_3$) δ: 7.10 (t, *J* = 7.7 Hz, 2H), 7.00 (d, *J* = 8.8 Hz, 2H), 6.30 (dd, *J* = 7.4, 0.9 Hz, 2H), 5.82 (s, 2H), 3.65 (s, 3H), 3.59 (s, 3H), 2.49 (dq, *J* = 15.7, 1.3 Hz, 1H), 2.45-2.36 (m, 1H), 2.25 -2.18 (m, 2H), 2.14- 1.99 (m, 1H), 1.68-1.62 (m, 1H), 1.24 (d, *J* = 2.8 Hz, 12H).

^{13}C NMR (126 MHz, CD_3CN) δ: 142.63, 140.83, 139.69, 137.29, 128.67, 106.54, 84.37, 57.66, 30.11, 26.99, 26.11, 24.98, 24.89. (C-B) Carbon signal not observed due to quadrupolar relaxation.

^{11}B NMR (128 MHz, $CDCl_3$) δ: 31.1, 28.5.

HRMS (ESI) Calcd for $[C_{24}H_{32}B_2N_2O_4+H]^+$ $[M+H]^+$: m/z 435.2629, found 435.2634.

Note: compound is not stable in $CDCl_3$ over time.

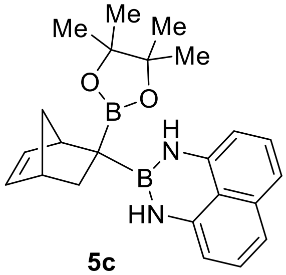

5c

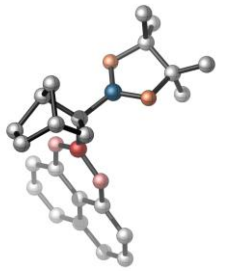

X-ray structure of 5c

***2-(4,4,5,5-tetramethyl-1,3,2-dioxaborolan-2-yl)bicyclo[2.2.1]hept-5-en-2-yl)-2,3-dihydro-1H-naphtho[1,8-de][1,3,2]diazaborinine* (5c):**

General procedure (A) followed, isolated **5c** gave *(dr= 91:9),* as a single diastereomer in (80 %) yield*)* as white solid, mp 150-154 °C, R_f = 0.382 (10% EtOAc in hexane).

^{1}H NMR (400 MHz, $CDCl_3$) δ: 7.07 (t, *J* = 7.7 Hz, 2H), 6.96 (d, *J* = 8.3 Hz, 2H), 6.27 (dd, *J* = 7.3, 0.9 Hz, 2H), 6.08-6.03 (m, 2H), 5.71 (s, 2H), 3.22 (s, 1H), 2.96 (s, 1H), 2.16 (dd, *J* = 11.3, 3.8 Hz, 1H), 1.36 (dq, *J* = 7.9, 1.7 Hz, 1H), 1.23 (d, *J* = 4.1 Hz, 12H), 1.13-1.09 (m, 2H).

^{13}C NMR (126 MHz, $CDCl_3$) δ: 141.56, 136.40, 135.76, 135.45, 127.67, 119.49, 117.20, 105.49, 83.50, 50.43, 48.18, 43.06, 31.13, 24.96, 24.66. (C-B) Carbon signal not observed due to quadrupolar relaxation.

^{11}B NMR (128 MHz, $CDCl_3$) δ: 34.2, 31.8.

HRMS (ESI) Calcd for $[C_{23}H_{28}B_2N_2O_2+H]^+$ $[M+H]^+$: m/z 387.2418, found 387.2417.

Note: the structure of **5c** was also confirmed by X-ray crystallographic analysis (see page S168).

5d

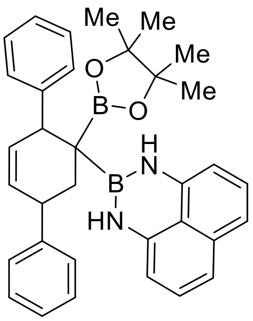

***2-(2'-(4,4,5,5-tetramethyl-1,3,2-dioxaborolan-2-yl)-1',2',3',4'-tetrahydro-[1,1':4',1''-terphenyl]-2'-yl)-2,3-dihydro-1H-naphtho[1,8-de][1,3,2]diazaborinine* (5d):**

General procedure (A) followed, isolated **5d** gave *(dr= 60:40),* in (82 %) yield as white solid for both diastereomers, mp 250-253 °C, R_f = 0.382 (10% EtOAc in hexane).

^{1}H NMR (400 MHz, $CDCl_3$) δ: 7.46-7.30 (m, 7H), 7.28-7.21 (m, 2H), 7.19-7.10 (m, 1H), 7.07 (t, *J* = 7.2 Hz, 3H), 7.01-6.94 (m, 4H), 6.90 (d, *J* = 0.9 Hz, 1H), 6.86 (d, *J* = 0.9 Hz, 1H), 6.35 (dd, *J* = 7.3, 1.0 Hz, 1H), 6.10-5.94 (m, 5H), 5.86 (s, 1H), 5.48 (s, 2H), 3.91 (d, *J* = 4.9 Hz, 1H), 3.84-3.78 (m, 1H), 3.70 (brs, 1H), 3.44-3.39 (m, 1H), 2.16-2.03 (m, 2H), 1.65 (dd, *J* = 13.5, 11.0 Hz, 1H), 1.28 (s, 6H), 1.25 (s, 6H), 0.91 (s, 3H), 0.82 (s, 3H).

^{13}C NMR (126 MHz, $CDCl_3$) δ: 146.89, 146.19, 143.98, 142.99, 141.30, 141.07, 136.42, 136.28, 132.46, 132.37, 131.13, 130.66, 130.40, 129.39, 128.77, 128.65, 127.98, 127.83, 127.76, 127.72, 127.56, 126.54, 126.50, 126.44, 126.38, 119.72, 119.50, 117.64, 117.26, 105.90, 105.41, 83.84, 83.35, 44.19, 42.25, 41.53, 41.00, 32.64, 31.74, 30.31, 25.27, 24.69, 24.53, 24.50, 22.80, 14.27. (C-B) Carbon signal not observed due to quadrupolar relaxation.

^{11}B NMR (128 MHz, $CDCl_3$) δ: 34.5.

HRMS (ESI) Calcd for $[C_{34}H_{36}B_2N_2O_2+H]^+$ $[M+H]^+$: m/z 527.3047, found 527.3034.

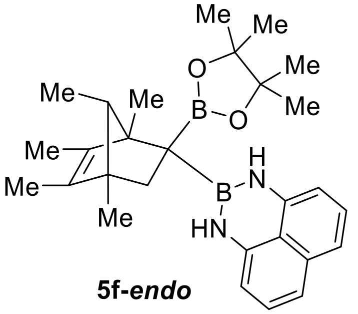

5f-*endo*

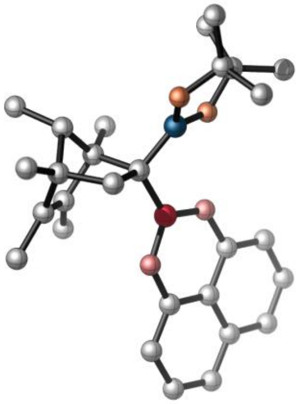

X-ray structure of 5f-*endo*

***2-1,4,5,6,7-pentamethyl-2-(4,4,5,5-tetramethyl-1,3,2-dioxaborolan-2-yl)bicyclo[2.2.1]hept-5-en-2-yl)-2,3-dihydro-1H-naphtho[1,8-de][1,3,2]diazaborinine* (5f-*endo*):**

General procedure (A) followed, isolated **5f-*endo*** gave (*endo:exo=99:01*) and *dr* (*syn:anti of Me'*) = *99:01*, in (85 %) yield as white solid for single diastereomer, mp 140-144 °C, R_f = 0.57 (10% EtOAc in hexane).

^{1}H NMR (500 MHz, $CDCl_3$) δ: 7.08 (t, *J*= 7.7 Hz, 2H), 6.96 (d, *J*= 8.3 Hz, 2H), 6.23 (dd, *J*=7.3, 0.9 Hz, 2H), 5.92 (s, 2H), 2.05 (d, *J*= 11.9 Hz, 1H), 1.63 (s, 3H), 1.42 (s, 3H), 1.40 (s, 1H), 1.31 (s, 12H), 1.24 (s, 3H), 1.17 (s, 1H), 1.14 (s, 3H), 0.55 (d, *J*= 3.3 Hz, 3H).

^{13}C NMR (126 MHz, $CDCl_3$) δ: 141.92, 136.53, 136.05, 133.44, 127.71, 119.46, 116.80, 105.19, 83.28, 61.41, 61.26, 53.27, 40.99, 25.33, 24.79, 15.82, 15.22, 11.60, 9.58, 8.81. (C-B) Carbon signal not observed due to quadrupolar relaxation.

^{11}B NMR (128 MHz, $CDCl_3$) δ: 30.8, 28.2.

HRMS (ESI) Calcd for $[C_{28}H_{38}B_2N_2O_2+H]^+$ $[M+H]^+$: m/z 457.3202, found 457.3206.

Note: the structure of **5f-endo** was also confirmed by X-ray crystallographic analysis (see page S170).

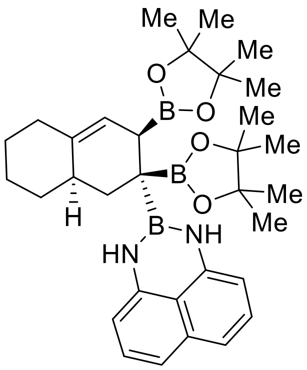

5k

Note: **5k** and **5k'** *(r.r. = 99:01)* and *(d.r. = 47:53)* using ***E*-3k** as 1,3-diene.

(±)2-((2S,3S)-2,3-bis(4,4,5,5-tetramethyl-1,3,2-dioxaborolan-2-yl)-1,2,3,5,6,7,8,8a-octahydronaphthalen-2-yl)-2,3-dihydro-1H-naphtho[1,8-de][1,3,2]diazaborinine **(5k):**

General procedure (A) followed, isolated **5k** in (42 %) yield, as white solid for a single diastereomer, mp 133-135 °C (decomposed) R_f = 0.37 (20% EtOAc in hexane).

^{1}H NMR (400 MHz, $CDCl_3$) δ: 7.08 (t, *J* = 7.7 Hz, 2H), 6.97 (d, *J* = 8.8 Hz, 2H), 6.30 (dd, *J* = 7.7, 0.9 Hz, 2H), 5.90 (s, 2H), 5.31 (s,1H), 2.35 (d, *J* = 12.2 Hz, 1H), 2.16 (d, *J* = 12.4 Hz, 1H), 2.09-1.99 (m, 2H), 1.88 (dd, *J* = 12.4, 5.7 Hz, 1H) 1.79-1.75 (m, 3H), 1.65 (t, *J* = 11.8 Hz, 1H), 1.45 (tt, *J* =12.7, 3.7 Hz, 1H), 1.33 (td, *J* =12.3, 3.5 Hz, 2H), 1.27 (d, *J* = 2.0 Hz, 12H), 1.21 (s, 6H), 1.18 (s, 6H).

^{13}C NMR (126 MHz, $CDCl_3$) δ: 143.49, 141.48, 136.46, 127.71, 119.66, 117.24, 105.55, 83.25, 83.17, 42.21, 37.90, 33.40, 30.10, 27.54, 25.21, 25.04, 24.96, 24.91, 24.74, 24.46, 21.53.

(C-B) Carbon signal not observed due to quadrupolar relaxation.

^{11}B NMR (128 MHz, $CDCl_3$) δ: 34.5.

HRMS (ESI) Calcd for $[C_{32}H_{45}B_3N_2O_4+H]^+$ $[M+H]^+$: m/z 555.3747, found 555.3753.

Note: the relative configuration of **5k** was determined by NOESY NMR (see page S183).

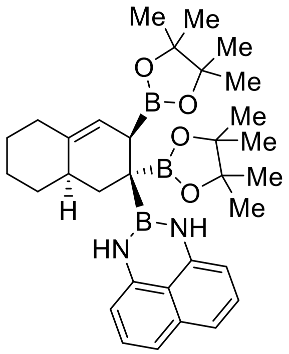

5k'

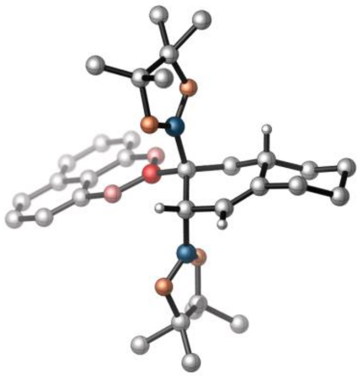

X-ray structure of 5k'

Note: **5k** and **5k'** *(r.r. = 99:01)* and *(d.r. = 47:53)* using ***E*-3k** as 1,3-diene.

***(±)2-((2R,3S)-2,3-bis(4,4,5,5-tetramethyl-1,3,2-dioxaborolan-2-yl)-1,2,3,5,6,7,8,8a-octahydronaphthalen-2-yl)-2,3-dihydro-1H-naphtho[1,8-de][1,3,2]diazaborinine* (5k'):**
General procedure (A) followed, isolated **5k'** in (41 %) yield, as white solid for a single diastereomer, mp 132-134 °C (decomposed) R_f = 0.428 (20% EtOAc in hexane).

^{1}H NMR (400 MHz, $CDCl_3$) δ: 7.08 (t, *J* = 7.7 Hz, 2H), 6.95 (d, *J* = 8.3 Hz, 2H), 6.27 (dd, *J* = 7.7, 0.9 Hz, 2H), 5.87 (s, 2H), 5.31 (d, *J* = 6.0 Hz, 1H), 2.25-2.18 (m, 3H), 2.02 (dd, *J* = 9.4, 6.4 Hz, 1H), 1.90-1.86 (m, 2H), 1.75-1.70 (m, 3H), 1.41-1.33(m, 1H), 1.32-1.26 (m, 1H), 1.17 (d, *J* =3.1 Hz, 12H), 1.09 (s, 6H), 1.05 (s, 6H), 0.96-0.93 (m, 1H).

^{13}C NMR (126 MHz, $CDCl_3$) δ: 141.77, 140.12, 136.41, 127.64, 121.19, 119.60, 116.88, 105.37, 83.19, 82.90, 36.65, 35.94, 35.93, 32.23, 28.38, 26.72, 24.91, 24.81, 24.57, 24.45.

(C-B) Carbon signal not observed due to quadrupolar relaxation.

^{11}B NMR (128 MHz, $CDCl_3$) δ: 32.4.

HRMS (ESI) Calcd for $[C_{32}H_{45}B_3N_2O_4+H]^+$ $[M+H]^+$: m/z 555.3747, found 555.3753.

Notes: (1)the structure of **5k'** was also confirmed by X-ray crystallographic analysis (see page S169). (2) the relative configuration of **5k'** was determined by NOESY NMR (see page S182)**.**

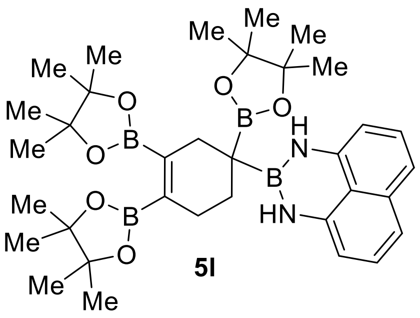

5l

***2-(1,3,4-tris(4,4,5,5-tetramethyl-1,3,2-dioxaborolan-2-yl)cyclohex-3-en-1-yl)-2,3-dihydro-1H-naphtho[1,8-de][1,3,2]diazaborinine* (5l):**

General procedure (A) followed, isolated **5l** in (80 %) yield, as white solid, mp 202-204 °C, R_f = 0.2 (10% EtOAc in hexane).

^{1}H NMR (500 MHz, $CDCl_3$) δ: 7.06 (t, *J* = 7.7 Hz, 2H), 6.96 (d, *J* = 8.3 Hz, 2H), 6.27 (dd, *J* =7.3, 0.9 Hz, 2H), 5.89 (s, 2H), 2.46 (d, *J* = 18.0 Hz, 1H), 2.30-2.13 (m, 3H), 1.91-1.86 (m, 1H), 1.91-1.86 (m, 1H), 1.75-1.70 (m, 1H), 1.31 (d, *J* = 1.1 Hz, 12H), 1.25 (s, 12H), 1.20 (s, 12H).

^{13}C NMR (126 MHz, $CDCl_3$) δ: 141.77, 136.43, 127.66, 119.76, 117.10, 105.57, 83.48, 83.33, 83.32, 31.54, 27.60, 26.26, 25.11, 25.06, 25.02, 24.96, 24.84, 24.79. (C-B) Carbon signal not observed due to quadrupolar relaxation.

^{11}B NMR (128 MHZ, $CDCl_3$) δ: 32.7.

HRMS (ESI) Calcd for $[C_{34}H_{50}B_4N_2O_6+H]^+$ $[M+H]^+$: m/z 627.4136, found 627.4150.

4. Procedure and characterization data for oxidation reaction of products 4O.

General procedure B -Oxidation & epoxidation of the *gem*-diborylcyclohexene:[5-6]

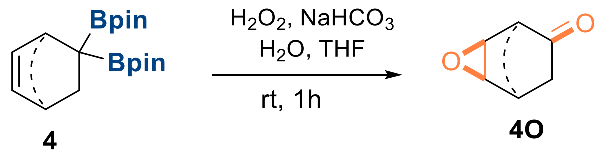

Compound (**4**) (50 mg, 0.1 mmol) was dissolved in a mixture of THF/ H_2O (1.5 mL, v/v = 2:1) at rt. Followed by addition of H_2O_2 (37 wt%, 1.5 mL), and $NaHCO_3$ (84 mg, 1.0 mmol) were slowly added at 25 °C. After stirring for 1 h at room temperature, the reaction mixture was partitioned between a saturated $NaHCO_3$ solution (10 mL) and CH_2Cl_2 (15 mL). The aqueous phase was extracted with ethyl acetate (15 mL x 3) and the combined organic layers were dried over Na_2SO_4, filtered, and concentrated under reduced pressure. The crude reaction mixture was purified by column chromatography on silica gel (EtOAc: Hexane = 1:10) to give a **4O** as pure product.

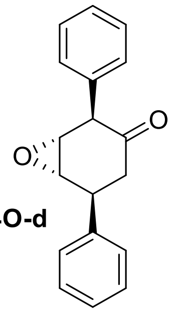

(±)2,5-diphenyl-7-oxabicyclo[4.1.0]heptan-3-one **(4O-d):**

General procedure (B) followed, isolated **4O-d** in (90 %) yield (*dr =98:02*) as white solid for single diastereomer, mp 105-107 °C, R_f = 0.54 (10% EtOAc in hexane).

^{1}H NMR (400 MHz, $CDCl_3$) δ: 7.31-7.27 (m, 7H), 7.21-7.16 (m, 3H), 3.51 (dd, J = 3.0 ,0.9 Hz, 1H), 3.41-3.34 (m, 1H), 2.81 (ddd, J = 17.3, 4.3, 1.4 Hz, 1H), 2.59-2.54 (m, 1H), 2.56 (dd, J =17.3, 12.2 Hz, 1H), 2.25 (ddd, J = 15.0, 11.4, 0.9 Hz, 1H).

^{13}C NMR (101 MHz, $CDCl_3$) δ: 205.05, 143.02, 134.04, 129.04, 128.56, 128.37, 127.22, 127.13, 126.99, 63.64, 61.80, 45.77, 34.11, 31.77.

HRMS (ESI) Calcd for $[C_{18}H_{16}O_2+H]^+$ $[M+H]^+$: m/z 265.1223, found 265.1235.

Note: the relative configuration of **4O-d** was determined by NOESY NMR (see page S184)**.**

Proposed pathway for preparation of **4O-d**:

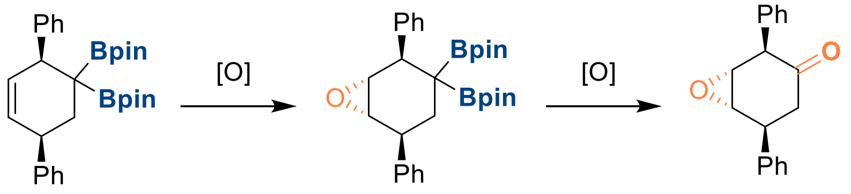

General procedure (C)- Oxidation of the *gem*-diborylcyclohexene:

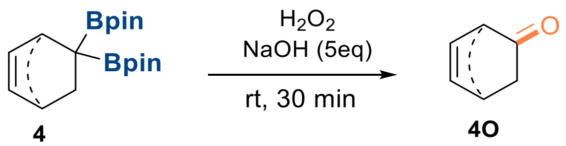

To a 10 mL dram vial, compound (**4**) (0.1 mmol) was dissolved in a mixture of of THF/H_2O (4 mL, 1:1 v/v). Followed by addition of H_2O_2 (37 wt%, 1.5 mL), and NaOH (3M) solution (5eq), then was left to stir for 30 min at room temperature. The reaction mixture was partitioned between a saturated brine solution (10 mL) and CH_2Cl_2 (15 mL). The aqueous phase was extracted with CH_2Cl_2 (15 mL x 3) and the combined organic layers were dried over $MgSO_4$, filtered, and concentrated under reduced pressure. The crude mixture was purified by column chromatography on silica gel (EtOAc: Hexane = 1:10) to give the pure products (**4O**).

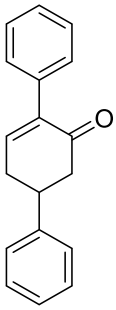

4O-d'

2,5-diphenylcyclohex-2-en-1-one **(4O-d'):**

General procedure (C) followed, isolated **4O-d'** in (85 %) yield as white solid, mp 116-118 °C, R_f = 0.48 (10% EtOAc in hexane).

^{1}H NMR (400 MHz, $CDCl_3$) δ: 7.31-7.26 (m, 5H), 7.25-7.24 (m, 1H), 7.23-7.21 (m, 3H), 7.19 (s, 1H), 7.02 (dd, J = 3.2 Hz, 1H), 3.45-3.38 (m, 1H), 2.85-2.73 (m, 3H), 2.65 (dd, J =10.8, 2.7 Hz, 1H).

^{13}C NMR (101 MHz, $CDCl_3$) δ: 197.42, 146.93, 146.91, 143.31, 140.49, 136.31, 128.96, 128.81, 128.24, 127.91, 127.18, 126.83, 45.88, 41.12, 34.67.

HRMS (ESI) Calcd for $[C_{18}H_{16}O+H]^+$ $[M+H]^+$: m/z 249.1274, found 249.1274.

Proposed pathway for preparation of **4O-d'**:

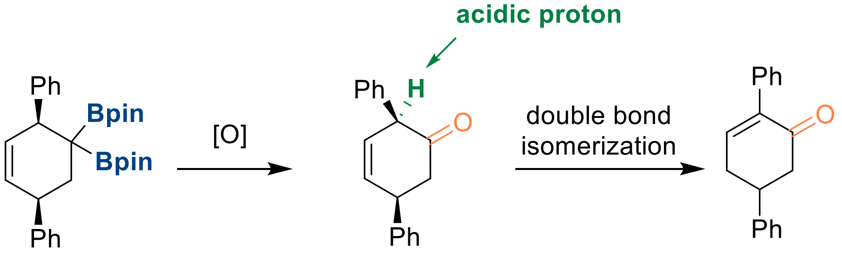

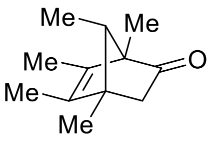

4O-f

***1,4,5,6,7-pentamethylbicyclo[2.2.1]hept-5-en-2-one* (4O-f):**

General procedure (C) followed, isolated **4O-f** in (90 %) yield as colorless oil, $R_f = 0.37$ (10% EtOAc in hexane).

^{1}H NMR (400 MHz, $CDCl_3$) δ: 2.50 (d, *J* = 17.5 Hz, 1H), 2.36 (d, *J* = 17.5 1H), 2.31 (q, *J* = 7.1 Hz, 1H), 1.63 (s, 6H), 1.31 (s, 3H), 1.07 (s, 3H), 0.94 (d, *J* = 7.2 Hz, 3H).

^{13}C NMR (101 MHz, $CDCl_3$) δ: 176.47, 139.43, 132.27, 100.02, 51.42, 48.08, 46.78, 20.31, 17.59, 15.16, 12.96, 9.61.

HRMS (ESI) Calcd for $[C_{12}H_{18}O+H]^+$ $[M]^+$: m/z 178.1352, found 178.1262.

Note: the relative configuration of **4O-f** was determined by NOESY NMR (see page S179)**.**

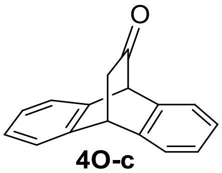

4O-c

***9,10-dihydro-9,10-ethanoanthracen-11-one* (4O-c):**[7]

General procedure (C) followed, isolated **4O-c** in (90 %) yield as white solid, mp 144-146 °C, $R_f = 0.61$ (10% EtOAc in hexane).

^{1}H NMR (400 MHz, $CDCl_3$) δ: 7.38 (td, *J* = 7.3, 0.6 Hz, 4H), 7.26-7.15 (m, 4H), 7.19 (s, 1H), 4.82 (s, 1H), 4.57 (t, *J* = 2.6 Hz, 1H), 2.35 (d, *J* = 2.6 Hz, 2H).

^{13}C NMR (101 MHz, $CDCl_3$) δ: 205.68, 142.65, 137.14, 127.52, 126.78, 125.69, 124.08, 63.03, 44.83, 38.93.

HRMS (ESI) Calcd for $[C_{16}H_{12}O+H]^+$ $[M+H]^+$: m/z 221.0961, found 221.0983.

The spectral data matched those reported in the literature.[7]

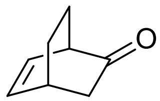

4O-g

***Bicyclo[2.2.2]oct-5-en-2-one* (4O-g):**

General procedure (C) followed, isolated **4O-g** in (76 %) yield as colorless oil, R_f = 0.43 (10% EtOAc in hexane).

^{1}H NMR (400 MHZ, $CDCl_3$) δ: 6.48 (t, *J* = 7.6 Hz, 1H), 6.20 (t, *J* = 6.5 Hz, 1H), 3.14-3.13 (m, 1H), 3.00-2.97 (m, 1H), 1.89-1.82 (m, 1H), 1.73-1.67 (m, 1H), 1.63-1.48 (m, 4H). The spectral data matched those reported in the literature.[8]

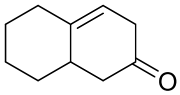

4O-k

***3,5,6,7,8,8a-hexahydronaphthalen-2(1H)-one* (4O-k):**[9]

General procedure (C) followed, isolated **4O-k** in (75 %) yield as colorless oil, R_f = 0.78 (10% EtOAc in hexane).

^{1}H NMR (400 MHz, $CDCl_3$) δ: 5.34 (m, 1H), 2.93 (dq, *J* = 20.4, 3.2 Hz, 1H), 2.78-2.74 (m, 1H), 2.59 (ddd, *J* = 13.4, 6.6, 1.7 Hz, 1H), 2.48 (brs, 1H), 2.34-2.31 (m, 1H), 2.26 (dd, *J* = 13.9, 8.5 Hz, 1H), 2.00 (t, *J* = 12.8 Hz, 1H), 1.90-1.87 (m,1H), 1.82-1.77 (m, 2H), 1.43-1.33 (m, 3H). The spectral data matched those reported in the literature.[9]

Proposed pathway for the preparation of **4O-k**:

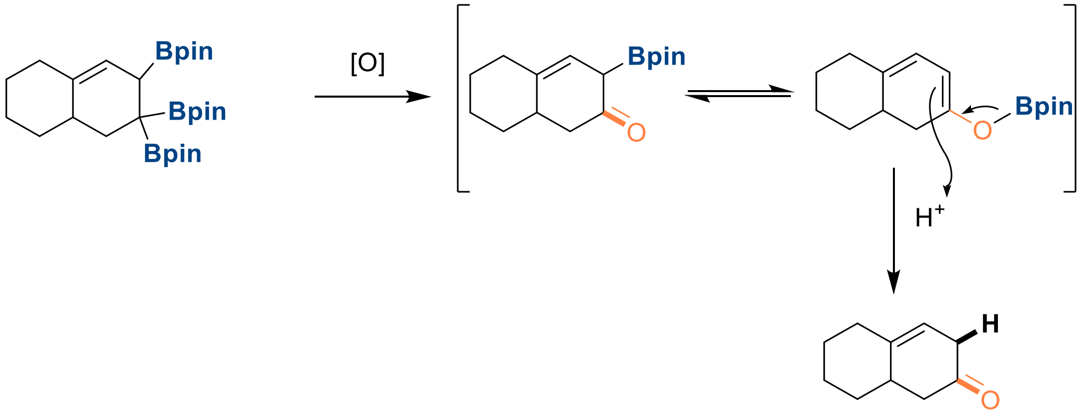

5. Procedure and characterization data for triflourination reaction of products 6.

General procedure (D) for the preparation of *gem*-diborylcyclohexene:

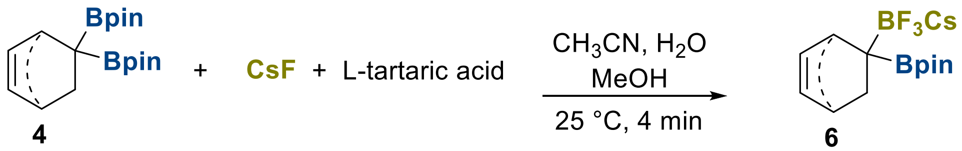

Similar to a reported Procedure,[10] *gem*-diborylcyclohexene (**4**) (0.50 mmol) was dissolved in a mixture of acetonitrile and methanol (2:2) ml. To the above mixture, a solution of potassium fluoride (2.0 mmol, in H_2O 0.2 mL) was added dropwise, and the mixture was stirred at room temperature for 2-4 minutes. *L*-(+)-tartaric acid (1.02 mmol, in 1 mL THF) was added dropwise to the rapidly stirring clear solution, during that time white precipitate crashed out. The reaction mixture was filtered to remove the white precipitate and washed thoroughly with excess acetonitrile (5 mL), then the filtrate was concentrated and recrystallized with hexane and diethyl ether furnished the corresponding potassium organotrifluoroborate (**6**) as amorphous solid, that was further dried under high vacuum for overnight.

Note: All mp experiments for *gem*-organotrifluoroborate salts obtained a decomposition at a range of 200-250 °C.

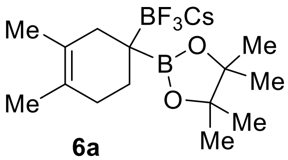

6a

***2-(3,4-dimethyl-1-(trifluoro-l4-boraneyl)cyclohex-3-en-1-yl)-4,4,5,5-tetramethyl-1,3,2-dioxaborolane, cesium salt* (6a):**

General procedure (D) followed, isolated **6a** in (91 %) yield as white solid.

^{1}H NMR (400 MHZ, DMSO-*d$_6$*) δ: 2.01 (brs, 1H),1.80 (d, *J* =16.4 Hz, 1H), 1.68-1.63 (m, 2H), 1.53 (brs, 1H), 1.49 (s, 3H), 1.44 (s, 3H), 1.12 (td, *J* = 12.4, 5.4 Hz, 1H), 1.02 (d, *J* = 9.1 Hz, 12H).

^{13}C NMR (101 MHz, DMSO-*d$_6$*) δ: 129.02, 123.08, 80.48, 36.49, 31.84, 27.06, 24.45, 24.42, 19.71, 19.11. (C-B) Carbon signal not observed due to quadrupolar relaxation.

^{11}B NMR (128 MHz, DMSO-*d$_6$*) δ: 35.4, 4.6.

^{19}F NMR (376 MHz, DMSO-*d$_6$*) δ: -142.2 (s).

HRMS (ESI) Calcd for $[C_{14}H_{24}B_2O_2F_3]^-$ $[M]^-$: m/z 302.1847, found 302.1963.

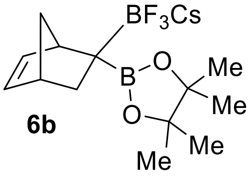

6b

***4,4,5,5-tetramethyl-2-((1S,2S,4S)-2-(trifluoro-l4-boraneyl)bicyclo[2.2.1]hept-5-en-2-yl)-1,3,2-dioxaborolane, cesium salt* (6b):**

General procedure (D) followed, isolated **6b** in (85 %) yield (*dr= 82:18)* as white solid for both diastereomers.

^{1}H NMR (400 MHz, DMSO-*d$_6$,* for **major diastereomer**) δ: 6.04 (dd, *J* = 5.4, 2.7Hz, 1H), 5.68 (dd, *J* = 5.4, 2.8 Hz, 1H), 2.65 (s, 1H), 1.61 (dd, *J* = 10.1, 3.5 Hz, 1H), 1.47 (d, *J* = 6.7 Hz, 1H), 1.24 (brs, 1H), 1.08 (d, *J* = 3.1 Hz, 12H), 0.96 (dd, *J* = 10.2, 1.5 Hz, 1H), 0.75-0.70 (m, 1H).

^{1}H NMR (400 MHz, DMSO-*d₆*, for **minor diastereomer**) δ: 5.83-5.79 (m, 1H), 5.63 (dd, *J* = 5.4, 2.6 Hz ,1H), 2.76 (s, 1H), 1.68 (dd, *J* = 10.0, 3.8 Hz, 1H), 1.24 (brs, 1H), 1.16 (d, *J* = 4.7 Hz, 1H), 1.09 (d, *J* = 8.1Hz, 12H), 0.93 (d, *J* = 9.8 Hz, 1H), 0.75-0.70 (m, 1H).

^{13}C NMR (101 MHz, DMSO-d_6, for **both diastereomers)** δ: 139.23, 132.38, 80.73, 80.57, 46.82, 45.72, 42.22, 31.84, 24.74, 24.70, 24.45, 24.33. (C-B) Carbon signal not observed due to quadrupolar relaxation.

^{11}B NMR (128 MHz, DMSO-d_6, for **both diastereomers**) δ: 35.3, 4.3.

^{19}F NMR (376 MHz, DMSO-d_6, for **major diastereomer**) δ: -135.4 (br).

^{19}F NMR (376 MHz, DMSO-d_6, for **minor diastereomer**) δ: -133.0 (br).

HRMS (ESI) Calcd for $[C_{13}H_{20}B_2O_2F_3]^+$ $[M]^+$: m/z 287.1601, found 287.1594.

Note: the relative configuration of **6b** was determined by NOESY NMR (see page S179)

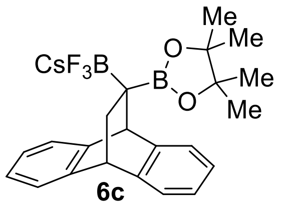

6c

(((11-(4,4,5,5-tetramethyl-1,3,2-dioxaborolan-2-yl)-9,10-dihydro-9,10-ethanoanthracen-11-yl)boraneylidene)-l3-fluoraneyl)cesium(III) fluoride **(6c):**

General procedure (D) followed, isolated **6c** in (76 %) yield as white solid.

^{1}H NMR (400 MHz, DMSO-d_6) δ: 7.10-7.00 (m, 4H), 6.84 (brs, 4H), 4.2 (s, 1H), 4.12 (s, 1H), 1.80 (d, *J* = 9.2 Hz, 1H), 1.58 (d, *J* =10.5 Hz, 1H), 0.9 (s, 6H), 0.74 (brs, 6H).

^{13}C NMR (101 MHz, DMSO-d_6) δ: 147.75, 145.98, 145.20, 144.71, 124.18, 123.69, 123.42, 123.35, 123.09, 122.60, 121.52, 80.87, 73.52, 47.71, 47.69, 45.28, 32.94, 24.96, 24.66, 24.35.

(C-B) Carbon signal not observed due to quadrupolar relaxation.

^{11}B NMR (128 MHz, DMSO-d_6) δ: 37.1, 4.3.

^{19}F NMR (376 MHz, DMSO-d_6) δ: -136.4 (s).

HRMS (ESI) Calcd for $[C_{22}H_{26}B_2O_2F_3]^-$ $[M]^-$: m/z 400.2006, found 400.1988.

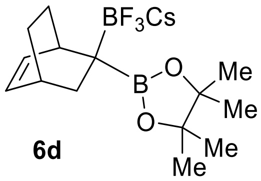

6d

***4,4,5,5-tetramethyl-2-((1S,2S,4R)-2-(trifluoro-l4-boraneyl)bicyclo[2.2.2]oct-5-en-2-yl)-1,3,2-dioxaborolane, cesium salt* (6d):**

General procedure (D) followed, isolated **6d** in (78 %) yield (*dr= 71:29)* as white solid for both diastereomers.

^{1}H NMR (400 MHz, DMSO-d_6, for **major diastereomer**) δ: 6.30 (t, J = 7.3 Hz, 1H), 5.92 (t, J = 7.3 Hz, 1H), 2.50-2.41 (m, 1H), 2.24 (m, 1H), 2.01-1.90 (m, 1H), 1.66-1.58 (m, 1H), 1.38-1.34 (m, 2H), 1.23 (m, 1H), 1.01 (d, J = 7.2 Hz, 12H), 0.7-0.67 (m, 1H).

^{1}H NMR (400 MHz, DMSO-d_6, for **minor diastereomer**) δ: 6.15 (t, J = 7.5 Hz, 1H), 5.80 (t, J = 7.2 Hz ,1H), 2.50-2.41 (m, 1H), 2.24 (m, 1H), 2.01-1.90 (m, 1H), 1.66-1.58 (m, 1H), 1.46-1.43 (m, 1H), 1.38-1.34 (m, 2H), 1.09 (s, 12H), 0.75-0.71 (m, 1H).

^{13}C NMR (101 MHz, DMSO-d_6, for **both diastereomers**) δ: 139.31, 138.01, 131.28, 128.93, 80.57, 33.39, 33.14, 32.80, 32.24, 30.96, 30.56, 27.69, 26.66, 24.80, 24.62, 24.56, 24.48, 24.20, 23.47. (C-B) Carbon signal not observed due to quadrupolar relaxation.

^{11}B NMR (128 MHz, DMSO-d_6, for **both diastereomers**) δ: 37.1, 4.4.

^{19}F NMR (376 MHz, DMSO-d_6, *for* **both diastereomers**) δ: -142.2 (s).

HRMS (ESI) Calcd for $[C_{14}H_{22}B_2O_2F_3]^+$ $[M]^+$:m/z 301.1758, found 301.1750.

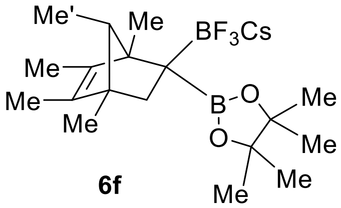

6f

***4,4,5,5-tetramethyl-2-((1R,2S,4R,7S)-1,4,5,6,7-pentamethyl-2-(trifluoro-l4-boraneyl) bicyclo[2.2.1] hept-5-en-2-yl)-1,3,2-dioxaborolane, cesium salt* (6f):**

General procedure (D) followed, isolated **6f** in (70 %) yield (*dr= 94:06)* as white solid for both diastereomers.

^{1}H NMR (400 MHz, DMSO-*d$_6$*, for **major diastereomers**) δ: 1.77 (q, *J* = 6.6 Hz, 1H), 1.54 (s, 3H), 1.38 (d, *J* = 9.8 Hz, 1H), 1.34 (s, 3H), 1.28 (d, *J* = 10.4 Hz, 1H), 1.04 (s, 6H), 0.99 (s, 3H), 0.95 (s, 6H), 0.88 (s, 3H), 0.31 (d, *J* = 6.3 Hz, 3H).

^{1}H NMR (400 MHz, DMSO-*d$_6$*, for **minor diastereomers**) δ: 1.78 (q, *J* = 6.6 Hz, 1H), 1.54 (s, 3H), 1.39 (d, *J* = 9.8 Hz, 1H), 1.34 (s, 3H), 1.29 (d, *J* = 10.4 Hz, 1H), 1.07 (d, *J* = 6.1 Hz, 12H), 0.99 (s, 3H), 0.88 (s, 3H), 0.36 (d, *J* = 6.5 Hz, 3H).

^{13}C NMR (101 MHz, DMSO-*d$_6$*, for **both diastereomers**) δ: 138.70, 130.36, 80.18, 79.19, 57.43, 56.19, 54.91, 51.23, 43.07, 25.47, 24.88, 24.43, 23.78, 16.69, 14.31, 12.14, 9.65, 8.72. (C-B) Carbon signal not observed due to quadrupolar relaxation.

^{11}B NMR (128 MHz, DMSO-*d$_6$, for* **both diastereomers**) δ: 38.6, 4.1.

^{19}F NMR (376 MHz, DMSO-*d$_6$*, for **both diastereomers**) δ: -131.7 (br).

HRMS (ESI) Calcd for $[C_{18}H_{30}B_2O_2F_3]^-$ $[M]^-$:m/z 357.2396, found 357.2375

6. Procedure and characterization data for the synthesis of 5f-exo.

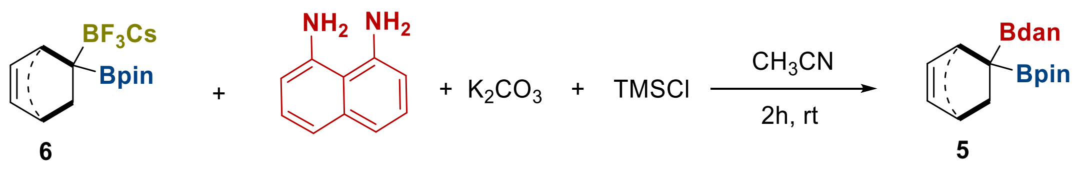

Prepared according to a literature reported procedure with slight modification.[10] into an oven-dried 50 ml Schlenk tube cooled under N_2, was charged with cesium salts of alkyl trifluoroborates (**6**) (0.2mmol), potassium carbonate (3equiv, 0.6mmol) and dissolved in CH_3CN (2 ml), to the reaction mixture, 1,8-diaminoaphthalene (1.3 equiv) was added, and then Trimethylsilylchloride (3equiv, 0.6mmol) was added dropwise. The reaction was stirred for 2-3 hours and was monitored by TLC, when completed the reaction was diluted with ethyl acetate (5ml) and filtered on filter paper, the remain filtrate were evaporated under reduced pressure to obtain a crude reddish product, in which was purified throw a short column on silica gel, yielding **5f-exo** as a white solid.

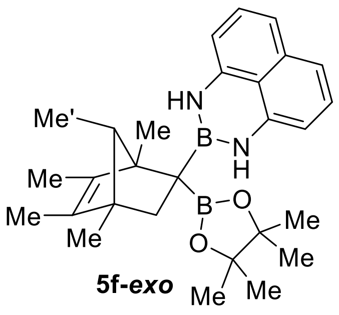

5f-*exo*

***2-1,4,5,6,7-pentamethyl-2-(4,4,5,5-tetramethyl-1,3,2-dioxaborolan-2-yl)bicyclo[2.2.1]hept-5-en-2-yl)-2,3-dihydro-1H-naphtho[1,8-de][1,3,2]diazaborinine* (5f-*exo*):**

General procedure (E) followed with (*dr= 92:08*), isolated **5f-*exo*** in (54 %) yield as white solid as a single diastereomer, mp 139-141 °C, R_f = 0.61 (10% EtOAc in hexane).

^{1}H NMR (500 MHZ, $CDCl_3$) δ: 7.09 (t, J = 7.8 Hz, 2H), 6.97 (d, J = 8.3 Hz, 2H), 6.29 (dd, J = 7.3, 0.9 Hz, 2H), 6.05 (s, 2H), 1.71 (d, J = 1.1 Hz, 1H), 1.66 (s, 3H), 1.54 (brs, 1H), 1.50 (d, J

=1.2 Hz, 3H), 1.36 (q, J = 6.3 Hz, 1H), 1.23 (s, 6H), 1.20 (s, 3H), 1.18 (s, 6H), 1.12 (s, 3H), 0.48 (d, J = 6.3 Hz, 3H).

^{13}C NMR (126 MHz, $CDCl_3$) δ: 141.68, 136.84, 136.49, 134.42, 127.74, 119.57, 117.12, 105.39, 83.12, 61.29, 59.62, 52.99, 41.29, 29.86, 26.24, 24.12, 16.20, 15.63, 11.82, 9.78, 8.60, 1.17. (C-B) Carbon signal not observed due to quadrupolar relaxation.

^{11}B NMR (161 MHZ, $CDCl_3$) δ: 31.1, 28.5.

HRMS (ESI) Calcd for $[C_{28}H_{38}B_2N_2O_2+H]^+$ $[M+H]^+$: m/z 457.3202, found 457.3206

7. Procedures and data for polymers poly-7 to poly-14.

General procedure for preparation of poly-7-BpinBpin polymer:

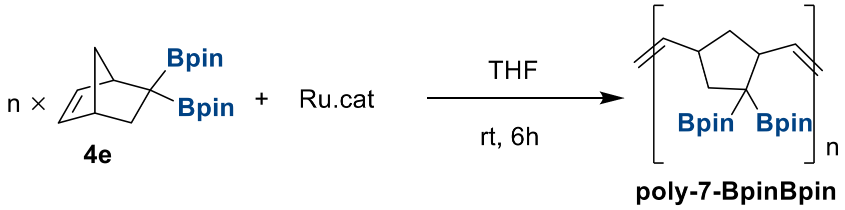

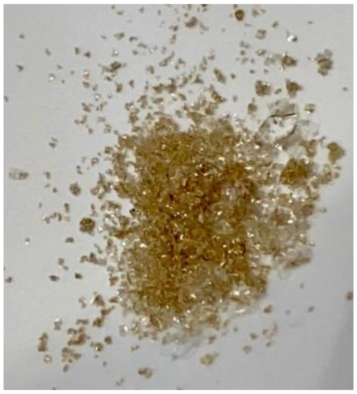

To a thick wall pressure tube contains a stir bar, *gem*-diboryl-norbornene (**4e**) (1 equiv) and ruthenium catalyst (0.01 equiv) were added under inert conditions (glovebox), along with THF. The tube was sealed, and the reaction was monitored by TLC, upon completion of the starting material 6h, solvent was evaporated and the crude mixture was treated with MeOH, to yield the solid **poly-7-BpinBpin** polymer by filtration.

^{1}H NMR (400 MHz, $CDCl_3$) δ: 5.51-5.05 (m), 3.20 (br), 2.87-2.71 (m), 2.30-1.91 (m), 1.19 (br).

^{13}C NMR (126 MHz, $CDCl_3$) δ: 133.33, 133.29, 132.26, 82.73, 46.07, 44.06, 43.95, 43.05, 41.81, 41.30, 40.99, 38.54, 37.94, 37.73, 37.39, 25.26, 25.18, 25.15, 25.07, 24.89, 24.85, 24.80, 24.70, 24.65, 24.52. (C-B) Carbon signal not observed due to quadrupolar relaxation.

^{11}B NMR (128 MHz, $CDCl_3$) δ: 31.1.

Table S6- Optimization of Solvent for polymerization reaction:

Entry	Solvent	Time (h)	% Conversion[a]
1.	Dichloromethane	24	100
2.	THF	6	100
3.	Toulene	24	65
4.	Water	24	0
5.	Ethanol	24	75
6.	Cyclohexane	24	50
7.	CH_3CN	24	0

[a] Reactions were carried out with 0.20 mmol of **4** and 1% of Ru.cat along with 2 ml of Solvent under inert conditions monitored by TLC.

<u>Comparison of *gem*-diboryl-norbornene (4e) and poly-7-BpinBpin polymer</u>

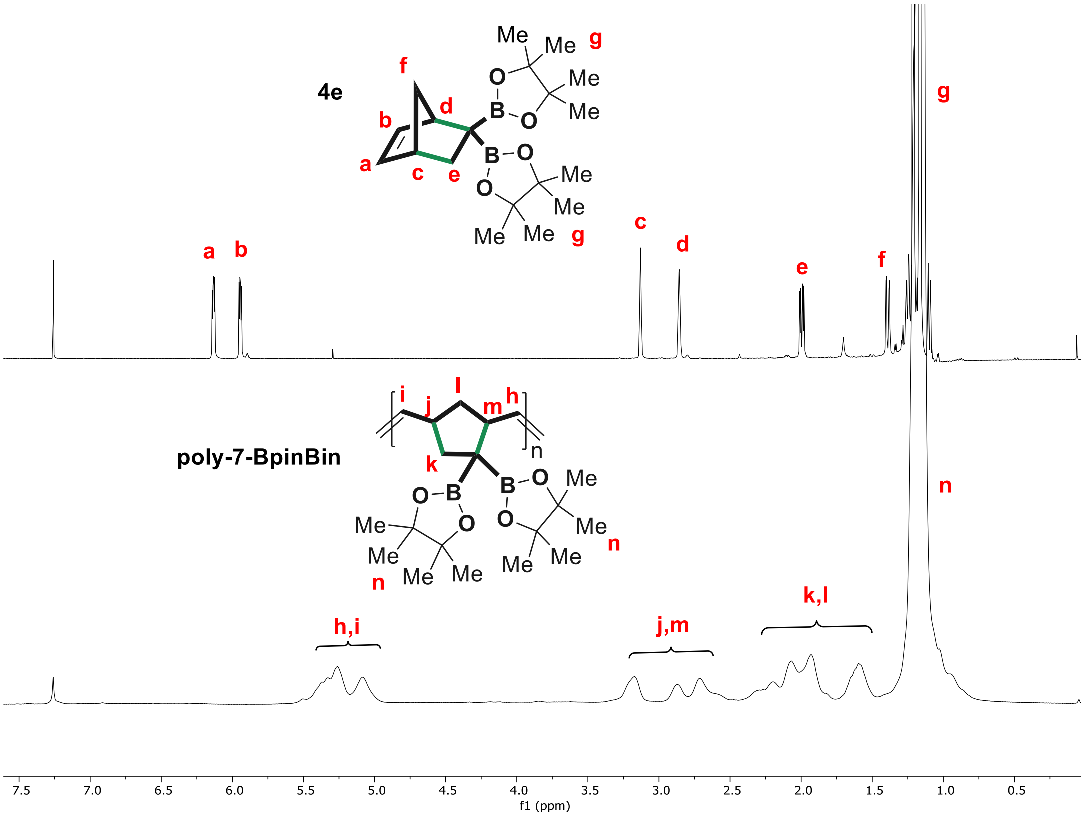

Polydispersity indexes (PDI) gel permeation chromatography (GPC) for poly-7-BpinBdan

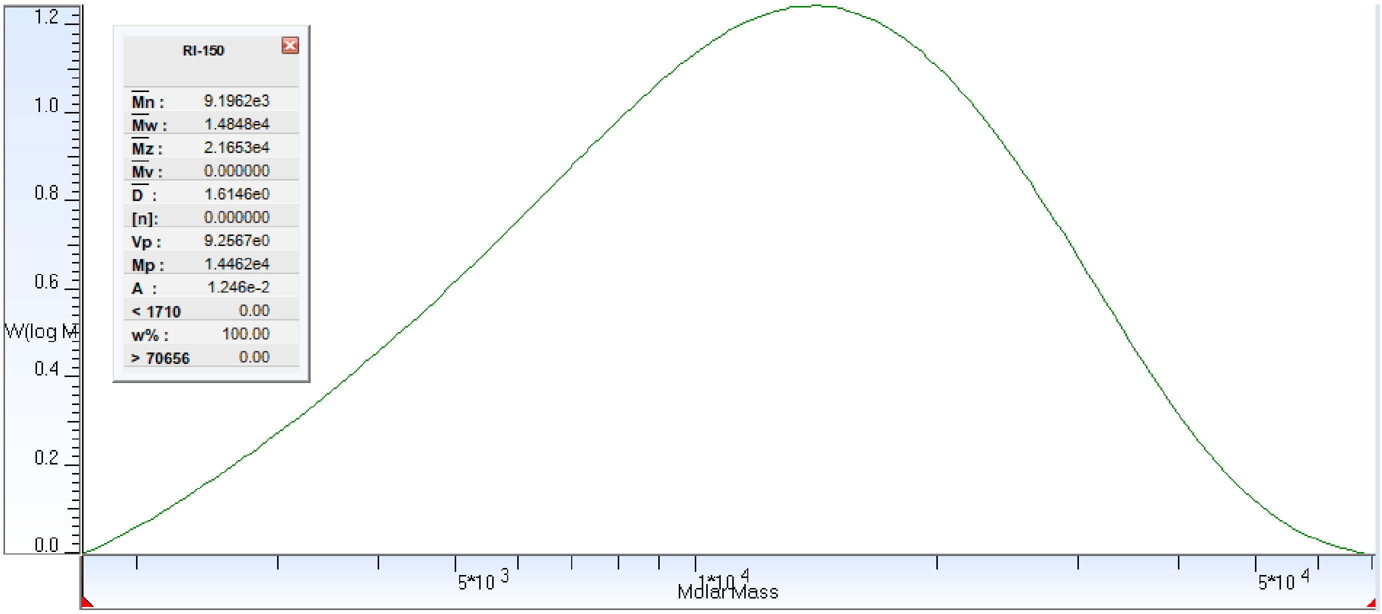

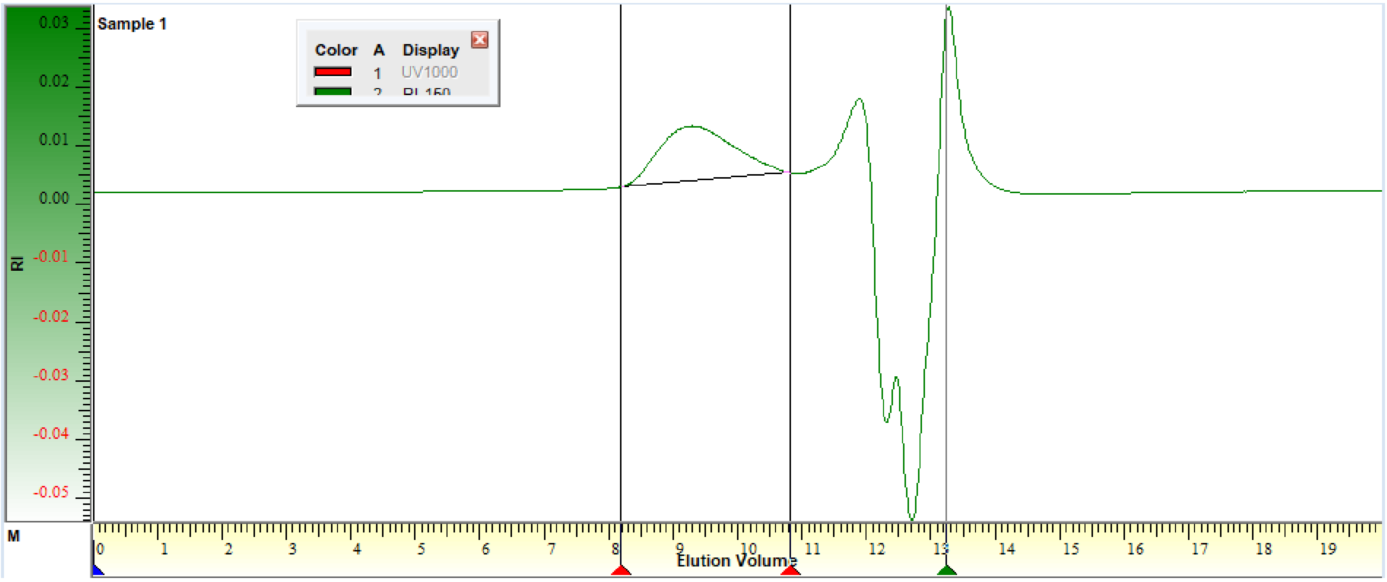

General procedure for preparation of poly-7-BpinBdan polymer:

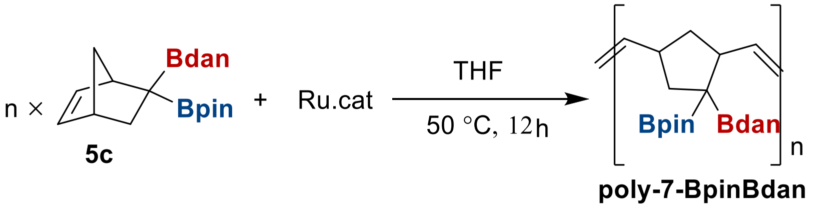

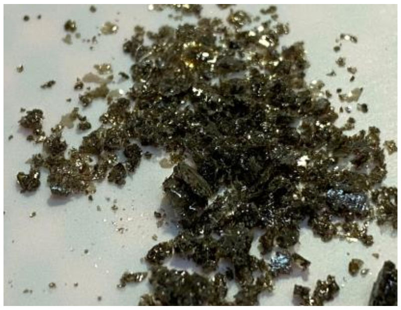

To a thick wall pressure tube contains a stir bar, *gem*-diboryl- norobornene **(5c)** (1 equiv) and ruthenium catalyst (0.01 equiv) were added under inert conditions (glovebox), along with THF. The tube was sealed, taken out of glovebox and heated to 50°C in a pre-heated oil bath, and the reaction was monitored by TLC. Upon completion of the starting material, solvent was evaporated and the crude mixture was treated with MeOH, to yield a black-green solid **poly-7-BpinBdan** polymer by filtration.

^{1}H NMR (400 MHz, $CDCl_3$) δ: 7.14-6.93 (m), 6.44-6.24 (m), 5.84-5.21 (m), 3.48 (br), 3.01 (br), 2.52-1.75 (m), 1.37-1.24 (m).

^{13}C NMR (126 MHz, $CDCl_3$) δ: 141.65, 141.44, 136.36, 134.35, 133.43, 127.63, 119.62, 119.43, 117.77, 117.08, 105.93, 105.66, 105.33, 83.42, 46.89, 43.28, 41.28, 38.08, 36.62, 35.56, 35.08, 29.74, 25.41, 24.94, 24.92, 24.85, 24.78, 24.63, 24.51, 24.40. (C-B) Carbon signal not observed due to quadrupolar relaxation.

^{11}B NMR (128 MHz, $CDCl_3$) δ: 33.5.

Comparison of *gem*-diboryl- norbornene (5c) and poly-7-BpinBdan polymer

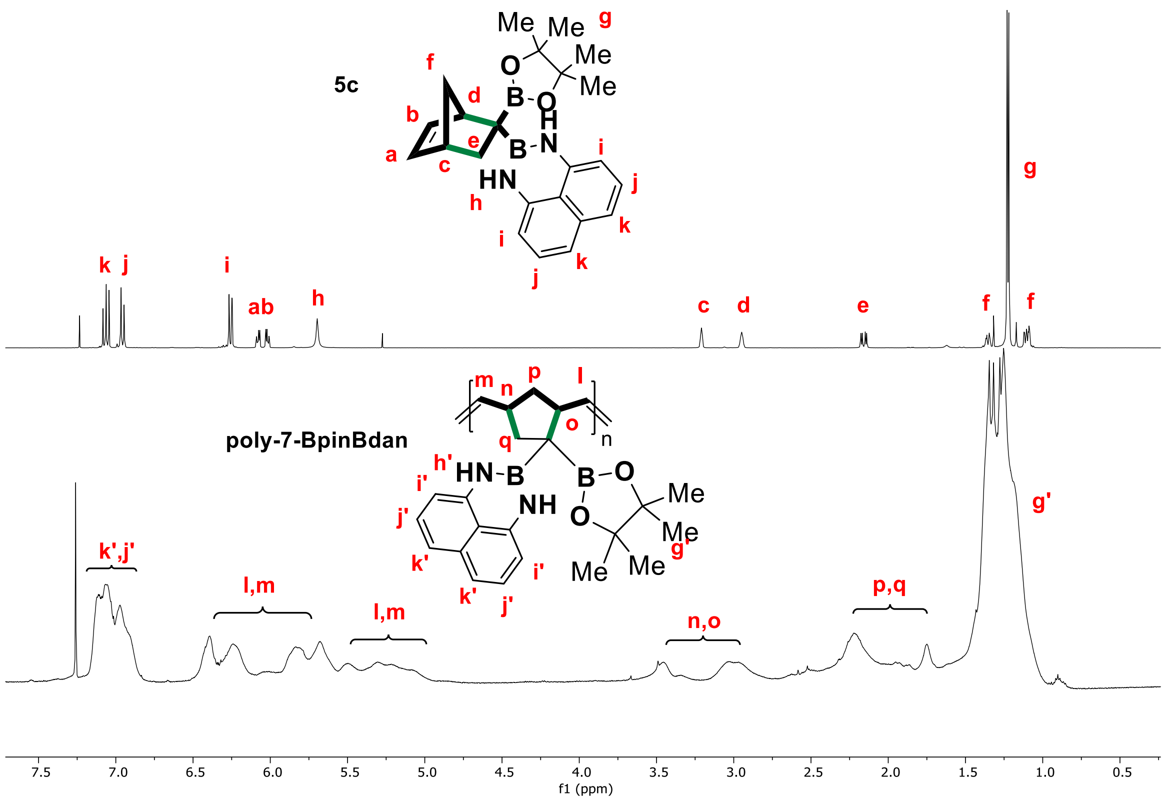

Polydispersity indexes (PDI) gel permeation chromatography (GPC) for poly-7-BpinBdan

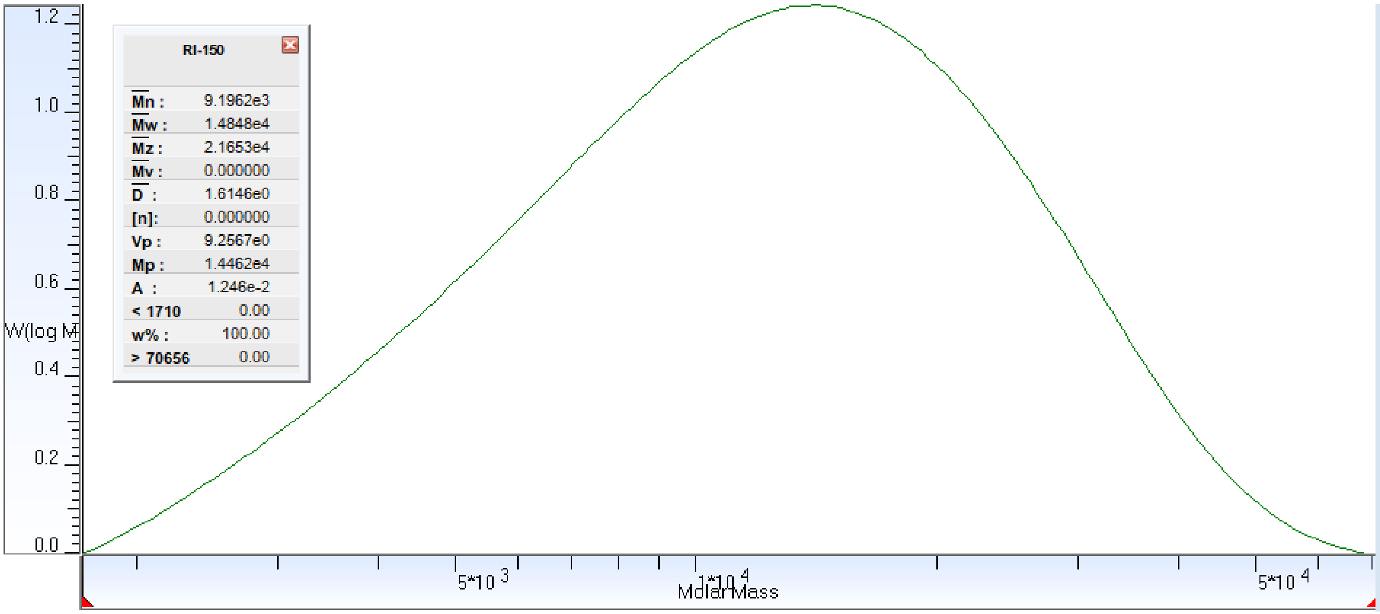

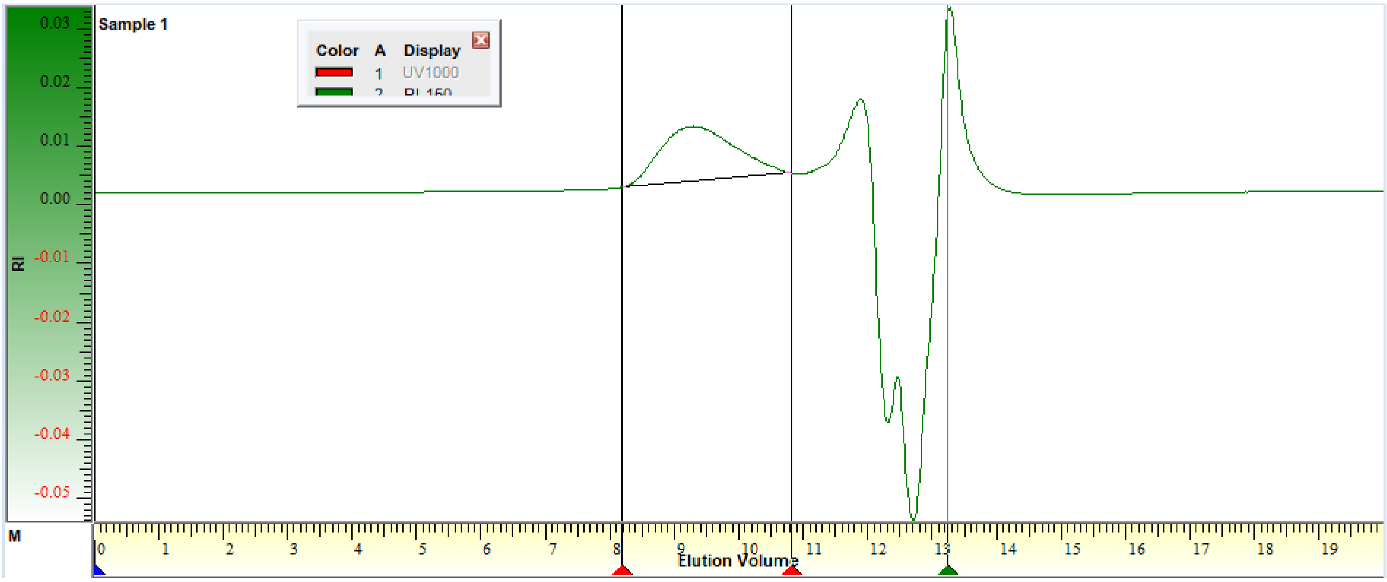

Thermogravimetric Analysis (TGA) for poly-7-BpinBpin and poly-7-BpinBdan

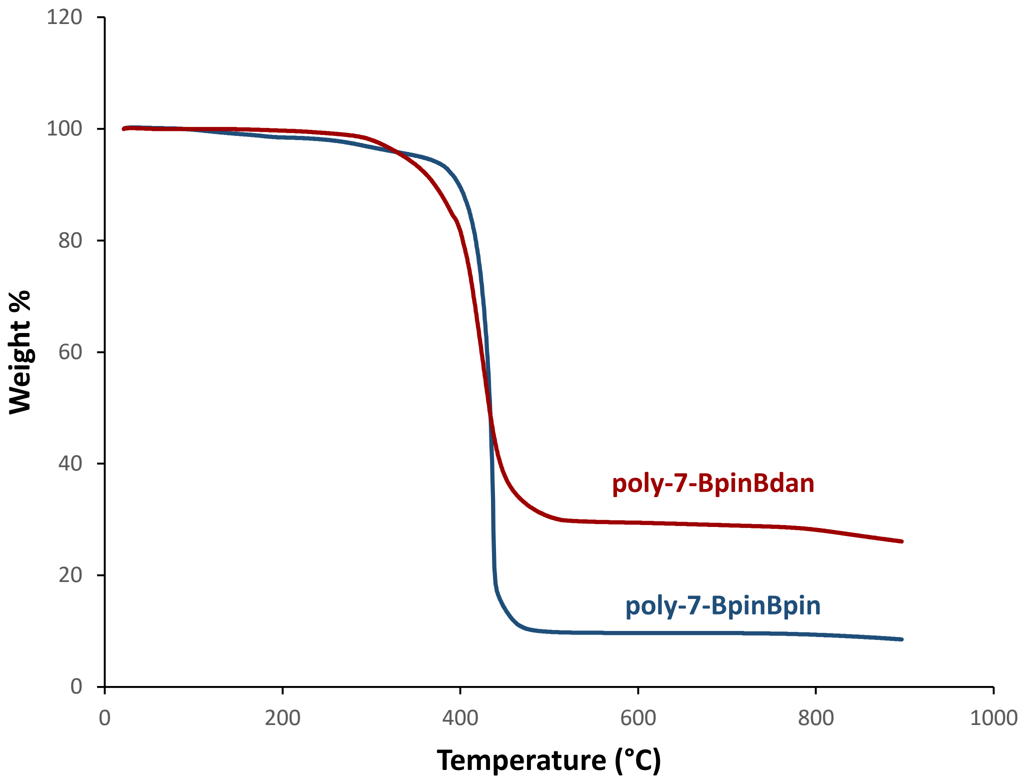

General procedure for the preparation of polymer poly-8-BpinBdan:

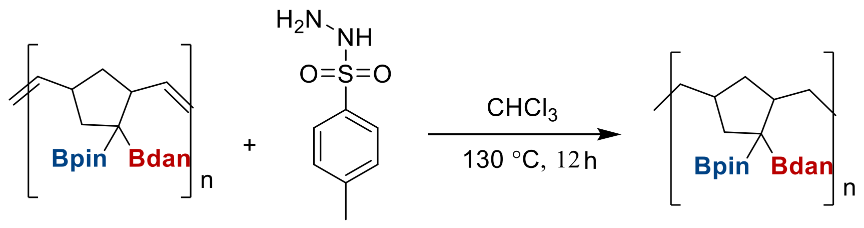

poly-7-BpinBdan 13 poly-8-BpinBdan

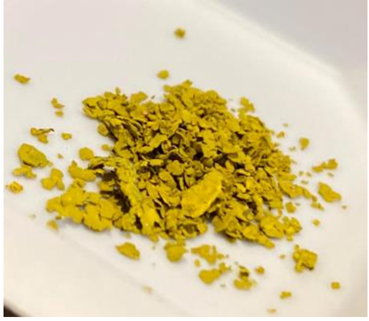

Prepared similar to a literature reported procedure.[11] In a thick wall pressure tube contains a stir bar, *gem*-diboryl polymer (**poly-7-BpinBdan**) (1 equiv) and *p*-toluenesulfonyl hydrazide (**13**) (4 equiv) were added, along with Chloroform, the tube was purged with Nitrogen and sealed, then heated to 130 °C in a pre-heated oil bath for 12 h. Upon completion, the crude mixture was added to an excess of MeOH, and filtered to obtain a yellow-green solid **poly-8-BpinBdan** polymer, that was further cleaned repeatedly by MeOH and dry in high vacuum for overnight.

^{1}H NMR (500 MHz, $CDCl_3$) δ: 7.10-6.99 (m), 6.32-6.26 (br), 5.94-5.79 (m), 2.40-1.53 (m), 1.21 (brs), 0.90-0.87 (m).

^{13}C NMR (126 MHz, $CDCl_3$) δ:141.61, 136.45, 130.05, 129.85, 128.89, 127.77, 126.87, 119.66, 117.15, 105.61, 83.31, 43.68, 43.05, 42.24, 41.02, 40.54, 40.00, 39.10, 36.20, 34.57, 33.68, 29.81, 29.46, 25.05, 24.46. (C-B) Carbon signal not observed due to quadrupolar relaxation.

^{11}B NMR (161 MHz, $CDCl_3$) δ: 35.1.

Comparison of ^{1}H-NMR for poly-7-BpinBdan and poly-8-BpinBdan

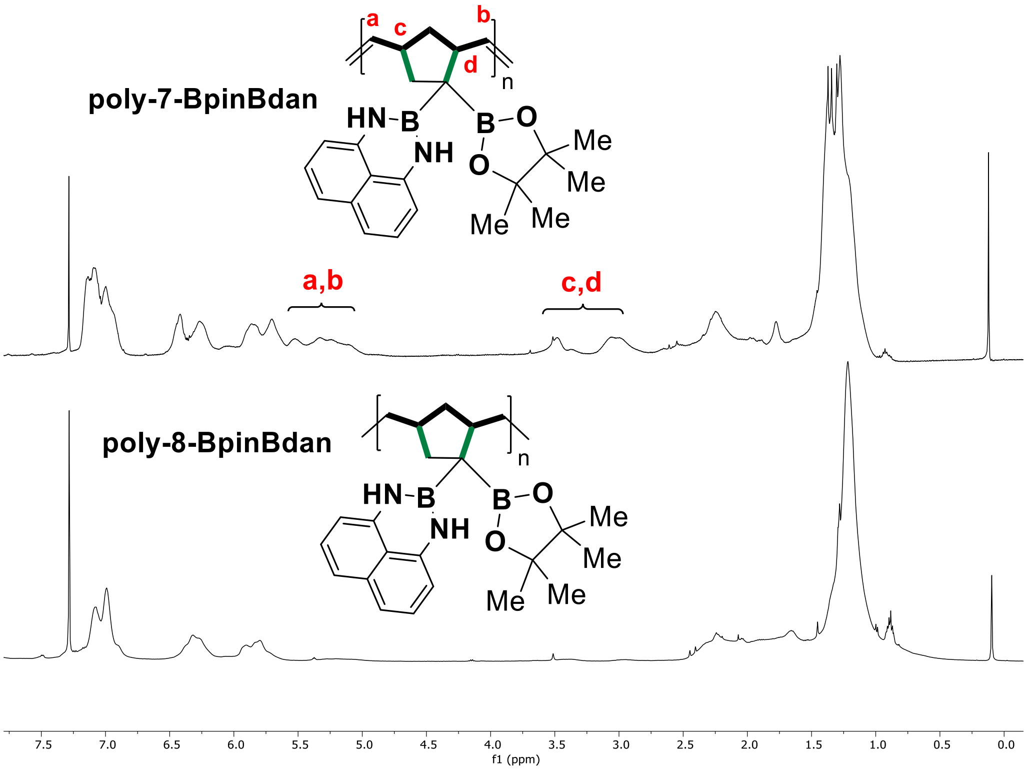

Comparison of ^{13}C-NMR for poly-7-BpinBdan and poly-8-BpinBdan

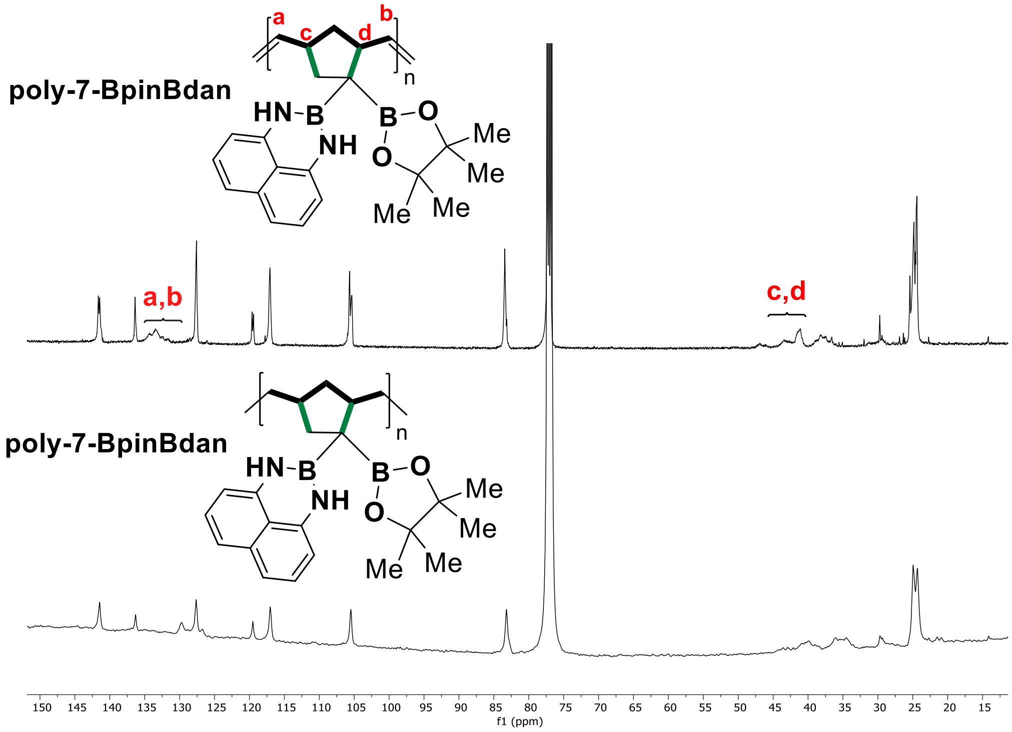

General procedure for the preparation of polymer poly-8-BpinBpin:

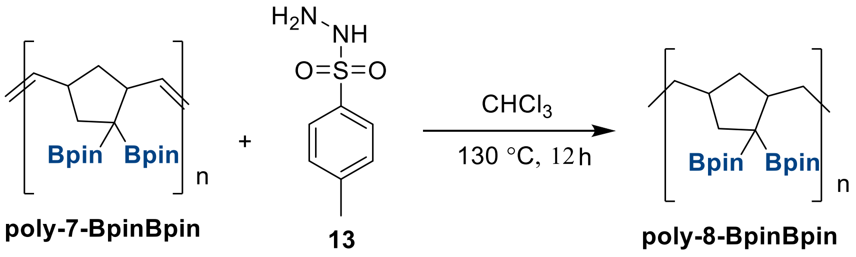

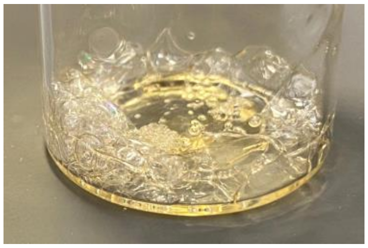

In a thick wall pressure tube that contains a stir bar, *gem*-diboryl polymer (**poly-7-BpinBpin**) (1 equiv) and *p*-toluenesulfonyl hydrazide (**13**) (4 equiv) were added, along with $CHCl_3$, the tube was purged with nitrogen and sealed, then heated to 130°C in a pre-heated oil bath for 12 h. Upon completion, the crude mixture was added to an excess of MeOH, and filtered to yield a slight yellow crystals **poly-8-BpinBpin** polymer by filtration, that was further cleaned repeatedly by MeOH and dry in high vacuum for overnight.

^{1}H NMR (500 MHz, $CDCl_3$) δ: 2.22-2.18 (br), 1.94-1.89 (br), 1.65-1.49 (m), 1.21-1.17(m), 1.08 (br), 0.71(br).

^{13}C NMR (126 MHz, $CDCl_3$) δ: 82.77, 47.71, 43.48, 43.34, 43.16, 43.06, 42.50, 42.24, 42.07, 40.88, 40.54, 40.28, 37.64, 37.51, 36.17, 35.33, 35.13, 34.59, 31.97, 31.07, 29.85, 29.58, 29.46, 29.17, 25.17, 25.11, 25.08, 24.99, 24.94, 24.90, 24.87, 24.65, 24.62.

19.05. (C-B) Carbon signal not observed due to quadrupolar relaxation.

^{11}B NMR (161 MHz, $CDCl_3$) δ: 37.2

Comparison of ^{1}H-NMR for poly-7-BpinBpin and poly-8-Bpin-Bpin

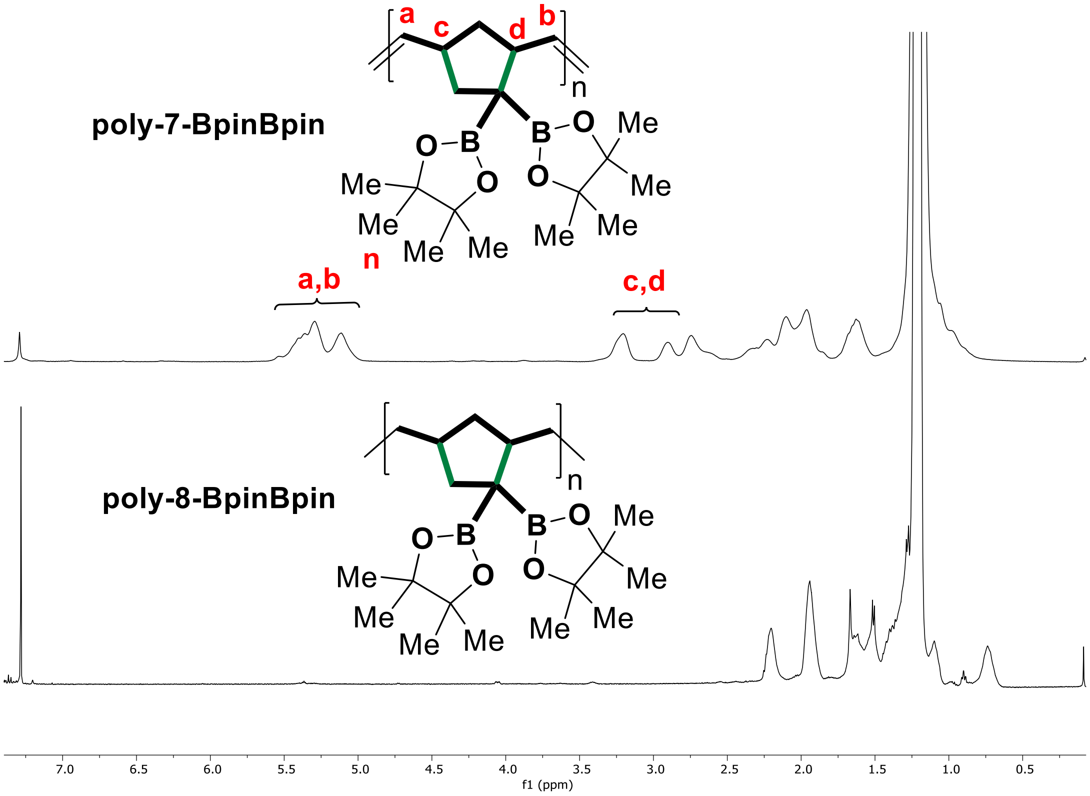

Comparison of ^{13}C-NMR for poly-7-BpinBpin and poly-8-Bpin-Bpin

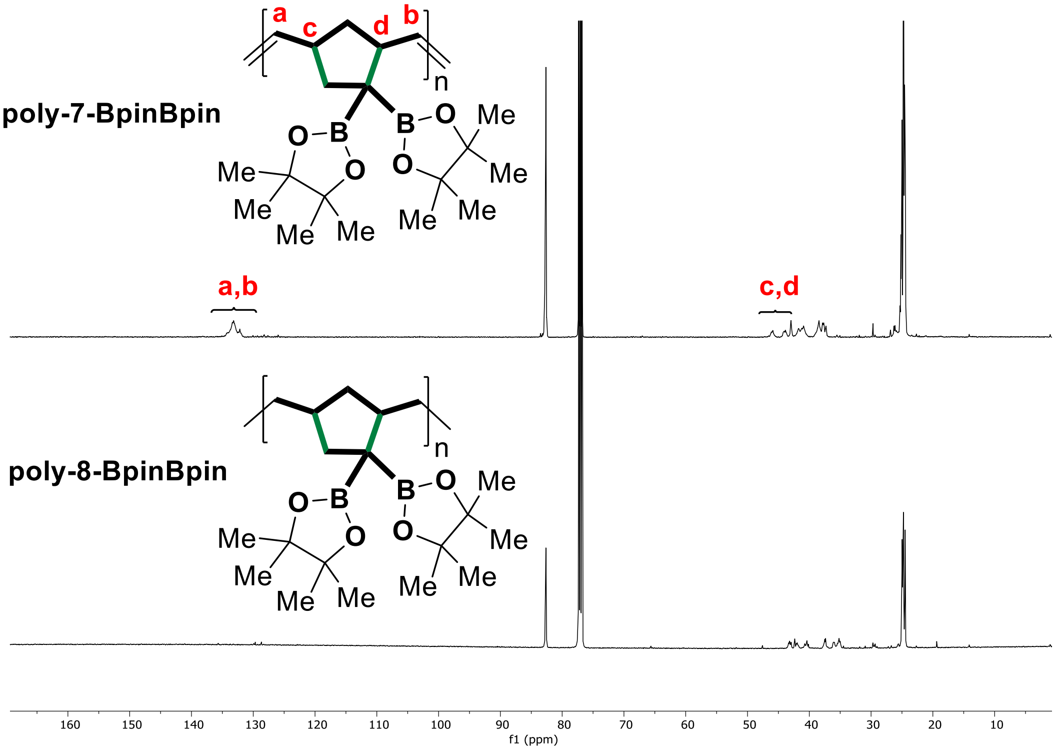

General procedure for the preparation of polymer poly-9-BpinBF$_3$K:

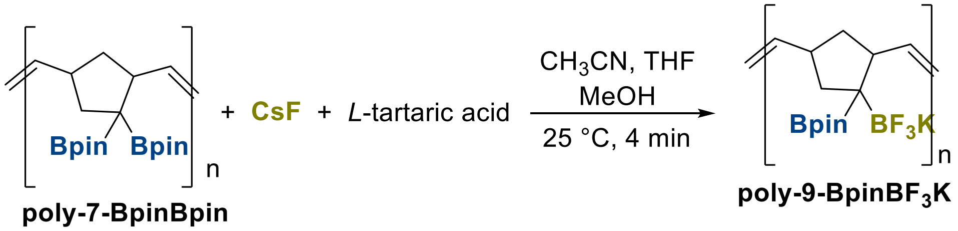

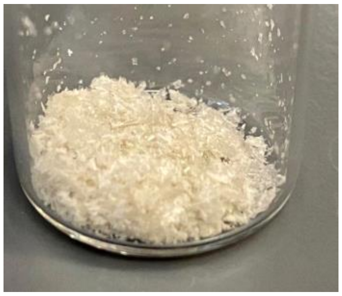

gem-diboryl alkane polymer (**poly-7-BpinBpin**) (1 equiv) was dissolved in a mixture of CH_3CN, THF and MeOH (1:1:1). To the above mixture, a solution of potassium fluoride (4 equiv, in H_2O) was added dropwise, and the mixture was stirred at room temperature for 2-4 minutes. *L*-(+)-tartaric acid (2.05 equiv, in THF) was added dropwise to the rapidly stirring clear solution, during that time white precipitate crashed out. The reaction mixture was steered for 10 more min, followed by filtration to remove the white precipitate and washed thoroughly with excess of CH_3CN and THF, the filtrate was concentrated and recrystallized with Et_2O furnished the corresponding potassium organotrifluoroborate (**poly-9-BpinBF$_3$K**) as a white solid, that was further dried under high vacuum for overnight.

^{1}H NMR (500 MHz, DMSO-*d$_6$*) δ: 5.33-4.98 (m), 3.13-2.76 (m), 2.03-1.44 (m), 1.36-1.12 (m).

^{11}B NMR (161 MHz, DMSO-*d$_6$*) δ: 31.0, 2.3.

^{19}F NMR (471 MHz, DMSO-*d$_6$*) δ: -133.5 to -134.9 (m), -139.8 to -140.9 (m).

Comparision of ^{11}B NMR for poly-7-BpinBpin and poly-9-BpinBF3K

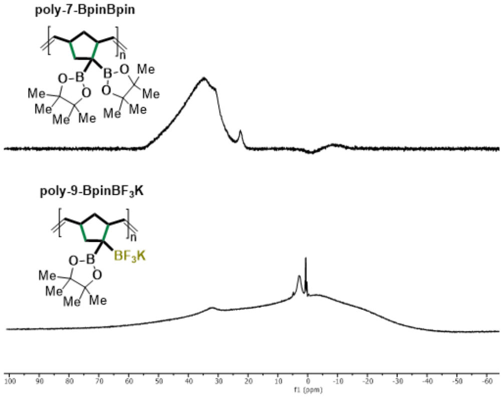

General procedure for the preparation of polymer poly-10-Bpin-H and poly-10-Bpin-D:

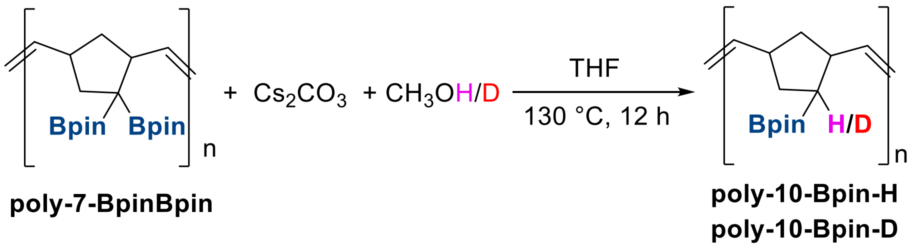

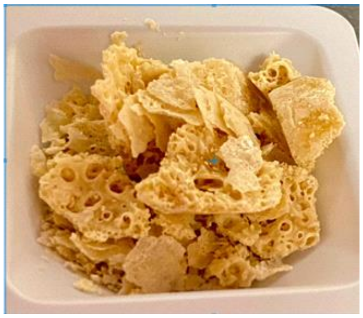

A thick wall pressure tube contains a stir bar, was taken inside the glovebox, *gem*-diboryl alkane polymer (**poly-7-BpinBpin**) (1 equiv) and Cs_2CO_3 (2 equiv) were dissolved in dry THF, along with CH_3OH/CD_3OD (5 equiv). The tube was sealed, taken out of glovebox and heated to 130°C in a pre-heated oil bath. After 12 h the reaction mixture was cooled to rt, and solvent was taken carefully without the precipitated base and added to excess of CH_3OH/CD_3OD, to yield a yellowish crystals of **poly-10-Bpin-H/D** polymer by filtration and dry in high vacuum for overnight.

For **poly-10-Bpin-H:**

^{1}H NMR (500 MHz, $CDCl_3$) δ: 5.37-5.15 (m), 3.27-2.60 (m), 2.34 (br), 1.95-1.86 (m), 1.70-1.51 (m), 1.20 (br).

^{13}C NMR (126 MHz, $CDCl_3$) δ: 133.18, 82.94, 46.18, 44.81, 42.57, 41.77, 39.91, 39.33, 38.72, 36.42, 35.64, 29.84, 27.89, 25.17, 24.86. (C-B) Carbon signal not observed due to quadrupolar relaxation.

^{11}B NMR (161 MHz, $CDCl_3$) δ: 35.1.

For **poly-10-Bpin-D:**

^{1}H NMR (500 MHz, $CDCl_3$) δ: 5.37-5.13 (m), 3.21-2.72 (m), 2.09-1.76 (m), 1.63-1.61 (m), 1.18 (br).

^{13}C NMR (126 MHz, $CDCl_3$) δ: 133.38, 132.99, 132.71, 82.90, 48.73, 48.03, 47.73, 46.62, 46.42, 45.88, 44.78, 43.05, 42.60, 41.80, 41.54, 41.34, 41.11, 39.91, 39.58, 39.29, 38.62, 38.11, 37.95, 37.67, 36.31, 36.07, 35.51, 34.61, 34.00, 33.67, 33.01, 32.69, 32.35, 31.86, 31.65, 31.29, 30.63, 29.94, 29.86, 29.77, 29.64, 29.49, 28.23, 27.37, 24.88. (C-B) Carbon signal not observed due to quadrupolar relaxation.

^{11}B NMR (161 MHz, $CDCl_3$) δ: 33.9.

D NMR (77 MHz, $CDCl_3$) δ: 1.42.

Comparison of ^{1}H-NMR for poly-7-BpinBpin, poly-10-Bpin-H and poly-10-Bpin-D

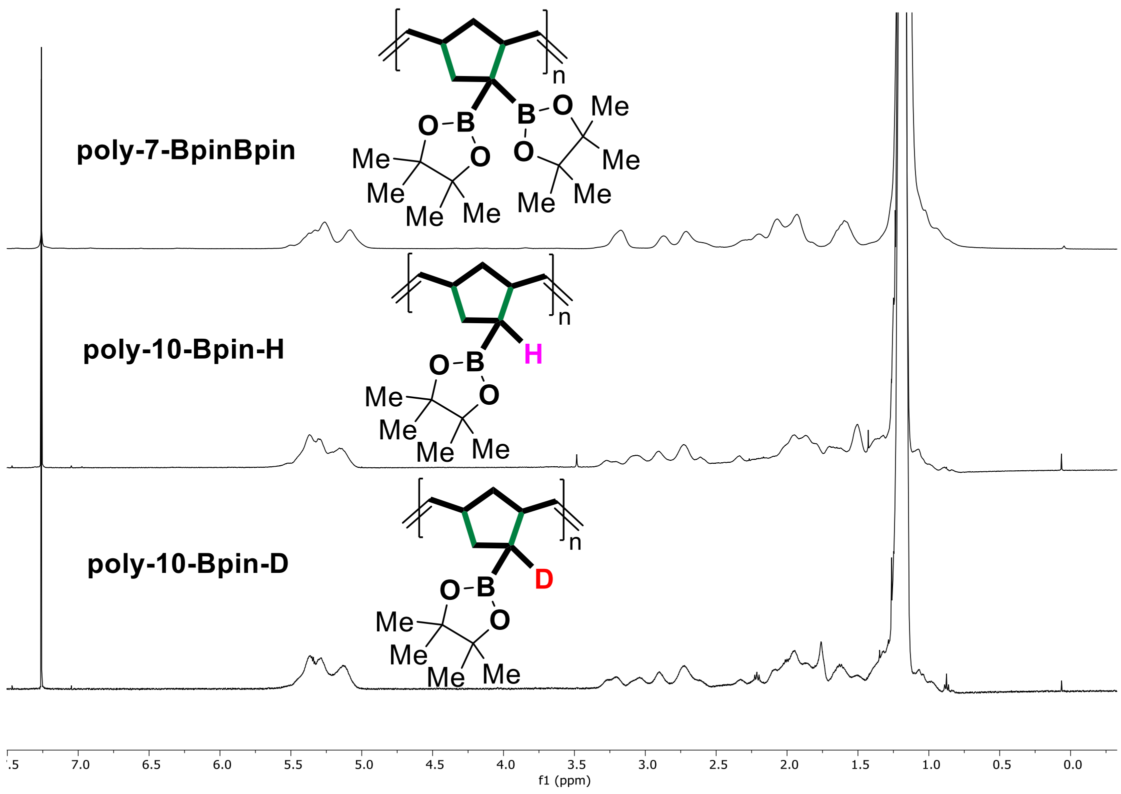

<u>**Comparison of ^{1}H-NMR for poly-7-BpinBpin and poly-10-Bpin-H**</u>

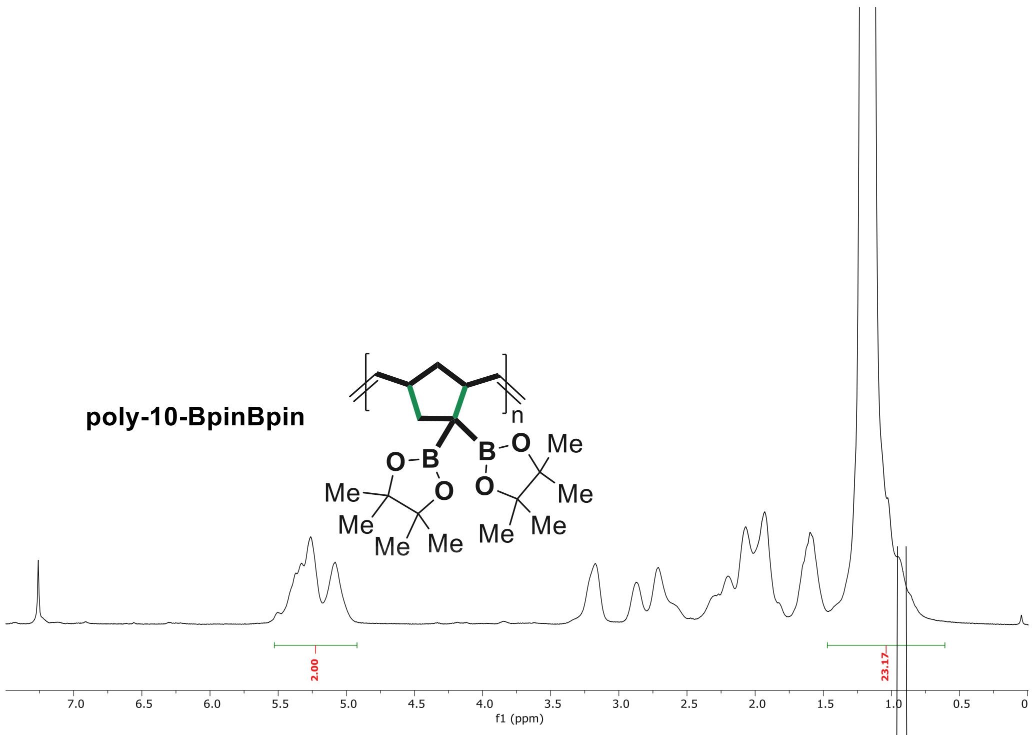

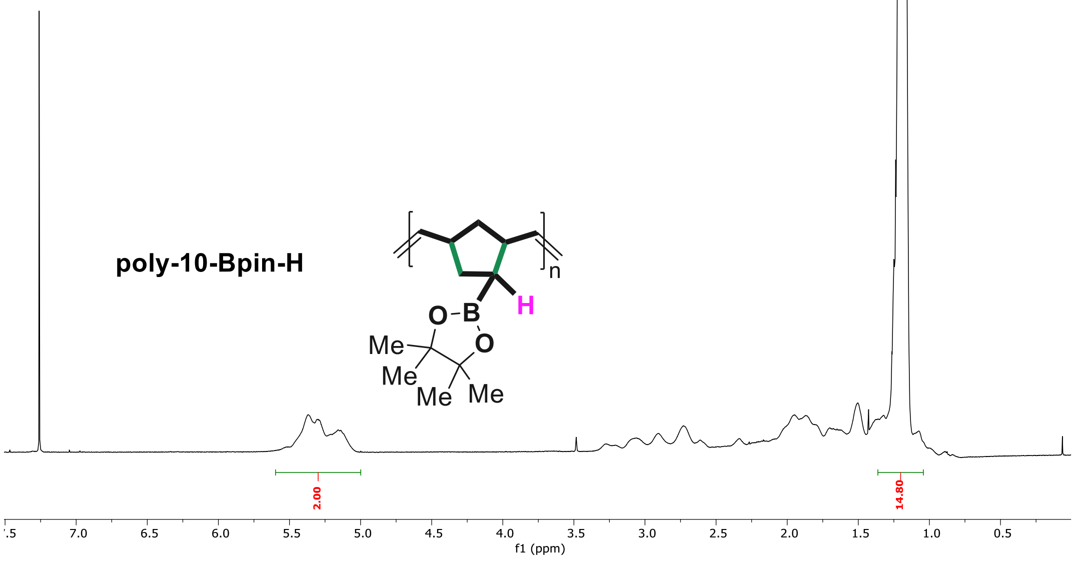

General procedure for the preparation of polymer poly-11-Bpin-H and poly-11-Bpin-D:

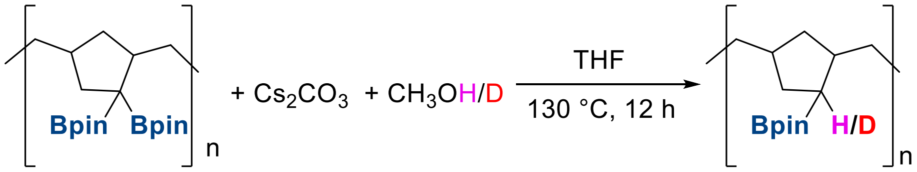

poly-8-BpinBpin **poly-11-Bpin-H** **poly-11-Bpin-D**

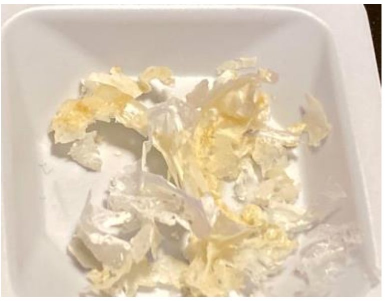

A thick wall pressure tube contains a stir bar, was taken inside the glovebox, *gem*-diboryl alkane polymer (**poly-8-BpinBpin**) (1 equiv) and Cs_2CO_3 (2 equiv) were dissolved in dry THF, along with CH_3OH/CD_3OD (5 equiv). The tube was sealed, taken out of glovebox and heated to 130°C in a pre-heated oil bath. After 12 h the reaction mixture was cooled to rt, and solvent was taken carefully without the precipitated base and added to excess of CH_3OH/CD_3OD, to yield a yellowish crystals of **poly-11-Bpin-H/D** polymer by filtration and dry in high vacuum for overnight.

For **poly-11-Bpin-H:**

^{1}H NMR (500 MHz, $CDCl_3$) δ: 2.23-2.17(m), 2.02-1.79 (m), 1.67-1.58 (m), 1.20 (br), 0.78-0.62 (m).

^{13}C NMR (126 MHz, $CDCl_3$) δ: 82.83, 77.41, 77.16, 76.91, 43.34, 41.88, 40.58, 37.44, 36.07, 35.39, 34.98, 31.08, 29.88, 29.71, 29.47, 27.36, 26.52, 25.07, 24.88. (C-B) Carbon signal not observed due to quadrupolar relaxation.

^{11}B NMR (161 MHz, $CDCl_3$) δ: 33.8.

For **poly-11-Bpin-D:**

^{1}H NMR (500 MHz, $CDCl_3$) δ: 2.20 (br), 1.93 (br), 1.78-1.64 (m), 1.21 (br), 0.74 (br).

^{13}C NMR (126 MHz, $CDCl_3$) δ: 82.82, 43.34, 41.96, 40.56, 39.06, 37.43, 36.05, 35.41, 34.97, 34.64, 31.06, 29.87, 29.71, 29.45, 28.19, 27.35, 26.51, 25.17, 25.05, 24.88, 24.61. (C-B) Carbon signal not observed due to quadrupolar relaxation.

^{11}B NMR (161 MHz, $CDCl_3$) δ: 33.9.

D NMR (77 MHz, $CDCl_3$) δ: 1.37.

General procedure for the preparation of polymer poly-12-BF3K-H:

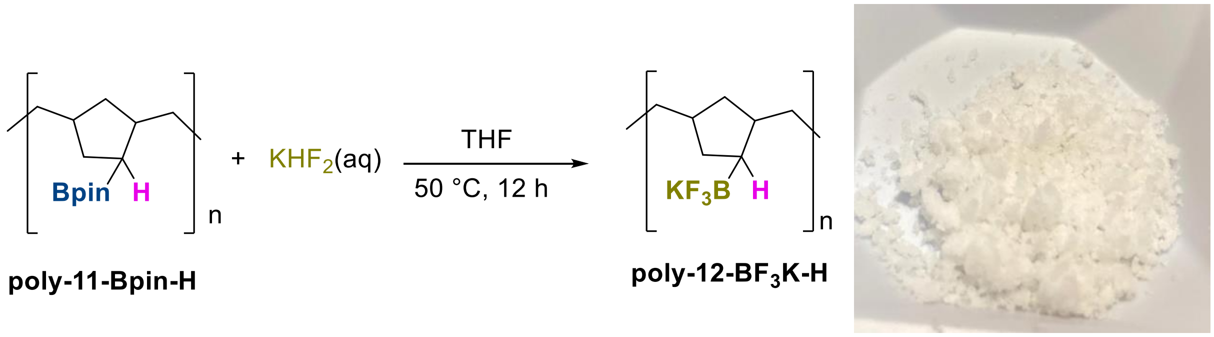

KHF_2 (aqueous 4 Molar) was added to a steered solution of (**poly-11-Bpin-H**) in THF. heated to 50°C in a water bath for 12 h. upon completion solvent was evaporated fully to yield a white solid, that was treated with Et_2O repeatedly to yield a white solid of **poly-12-BF$_3$K** that was further dried in high vacuum at 50 °C over night.

Comparision of ^{11}B NMR for poly-11-Bpin-H and poly-12-BF3K-H

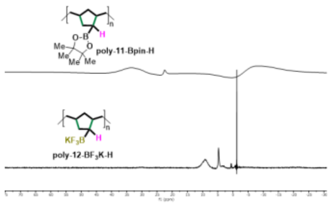

^{1}H NMR (500 MHz, DMSO-d_6) δ: 2.03-1.96 (m), 1.24 (br), 0.86 (s).

^{11}B NMR (128 MHz, DMSO-d_6) δ: 4.7.

^{19}F NMR (471 MHz, DMSO-d_6) δ: -139.2 to -139.4 (m).

General procedure for the preparation of polymer poly-14-Ar-H:

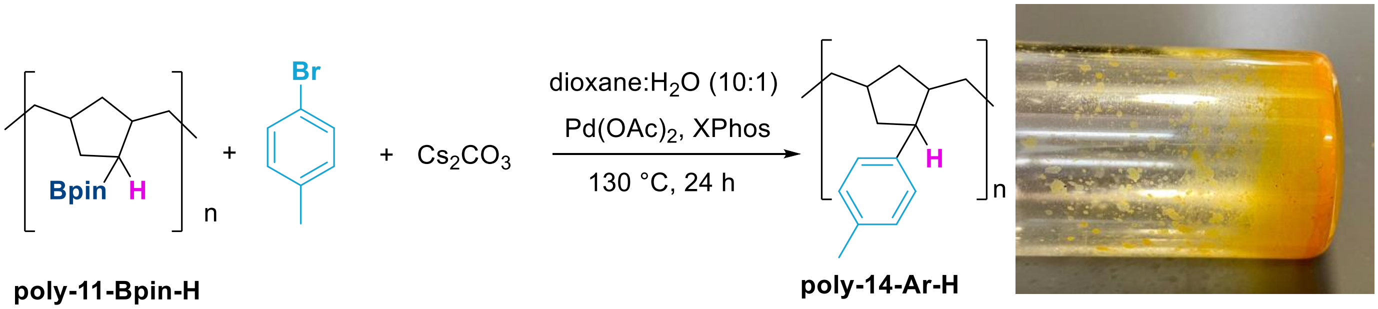

A thick wall pressure tube contains a stir bar, was taken inside the glovebox, $Pd(OAc)_2$ (10 mol%), XPhos (25 mol%) and Cs_2CO_3 (2.5 equiv) were added along with dioxane, polymer (**poly-11-Bpin-H**) (1 equiv) was added, along with H_2O, the tube was sealed, heated to 130 °C in a pre-heated oil bath. After 12 h the reaction mixture was cooled to rt, and solvent was taken carefully without the precipitated materials and added to excess of CH_3OH, to yield an orange solids of **poly-14-Ar-H** polymer by filtration and dry in high vacuum for overnight.

^{1}H NMR (500 MHz, CDCl$_3$) δ: 7.32 (br), 6.77 (br), 2.19 (br), 2.04-1.54 (m), 1.23 (br), 0.89-0.76 (m).

^{13}C NMR (126 MHz, CDCl$_3$) δ: 135.74, 134.70, 128.42, 25.09, 24.89, 24.61, 23.11, 21.31.

<u>Comparison of ^{1}H-NMR for crude poly-14-Ar-H, poly-14-Ar-H and poly-11-Bpin-H</u>

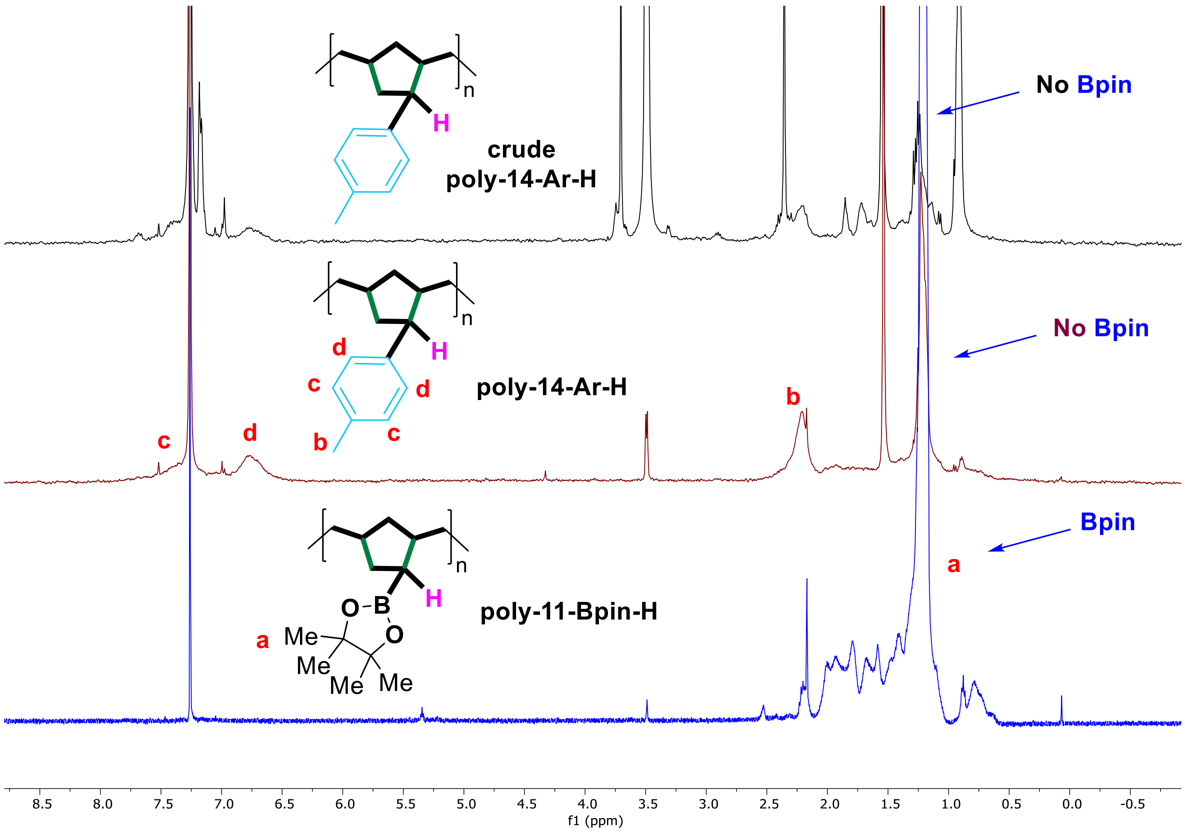

Comparison of ^{1}H-NMR between poly-7-BpinBpin, poly-10-Bpin-H, poly-10-Bpin-D, poly-8-BpinBpin, poly-11-Bpin-H, poly-11-Bpin-D and poly-14-Ar-H

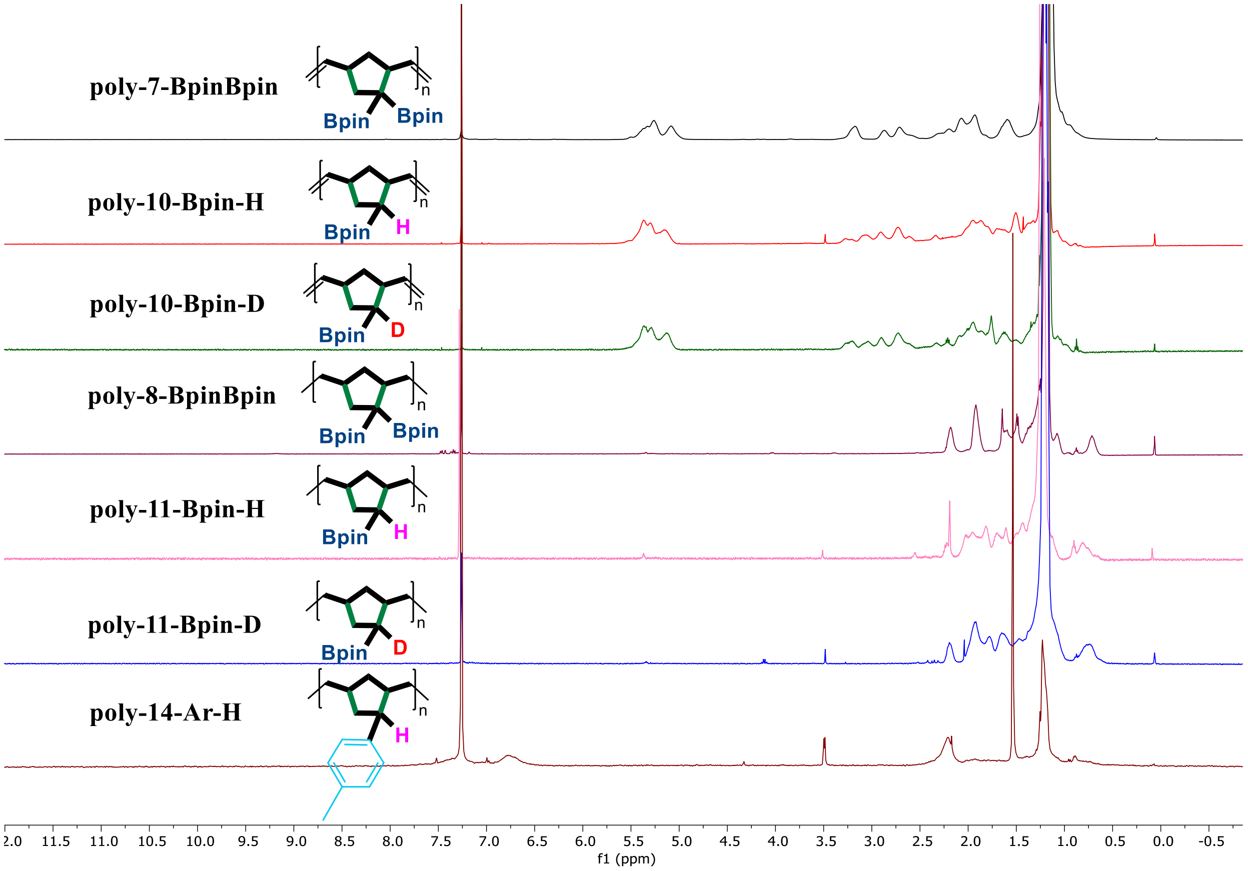

8. Computational Details.

All calculations in this manuscript were performed with Gaussian 16 software package.[12] Structures presented in this manuscript were optimized in the gas phase using the M06-2X[13] functional with the 6-311(d) basis set.[14] Each structure was identified as a minimum or a saddle point on the potential energy surface by performing vibrational frequency calculations at the same level of theory. In order to calculate the free energy in solution, we performed single point calculation with Toluene solvent using the SMD method,[15] which is considered suitable for the solvation free energy calculations.[16]

In some cases, low frequencies were observed. We have used frequency cutoff according to Grimme's correction[17] as implemented in the GoodVibes software package[18] to check their effect on the final results. The impact of the low frequencies on the relative free energies has been demonstrated as minor and thus not reported.

GHelpG analysis:

We have performed CHelpG[19] population analysis on *gem*-diborylalkene (**2**) and vinylboron (**1**) dienophiles. The partial charge difference on C2 hints that additional substituents i.e. Bpin result in less electron concentration between the carbon-carbon double-bond, prompting the reaction. Thus, *gem*-diborylalkene (**2**) is more dienophilic than the vinylboron (**1**).

Table S7- Chelpg population analysis:

	vinylboron (**1**)	*gem*-diborylalkene (**2**)
C1	-0.32	-0.32
C2	-0.34	-0.29
Sum	-0.65	-0.60

* Chelpg population analysis on the dienophile's reactive carbons of the double-bonds. C1 is the carbon that is bonded to the Bpin, C2 is not bonded to Bpin.

Consideration of the stabilization for the different transition states:

The proximity between hydrogens on the Cyclopentadiene (**3e**) and oxygens on the Bpin substituent can stabilize the transition state. The relative stability of Bpin-Exo-TS (Figure S1a) with respect to Bpin-Endo-TS (Figure S1b), can be explained by the fact that in the Bpin-Exo-TS hydrogen bond is formed between the acidic allylic hydrogen and oxygen on the Bpin group while in the Bpin Endo no such bond is formed. Hydrogen bonds might also form between the vynilic hydrogens and the oxygen on the Bpin group. Such hydrogen bonds are expected to be weaker than hydrogen bond of the acidic allylic hydrogens, [20] but can give further stabilization and may be responsible for the relative stability of *gem*-diborylalkene (**2**) TS (Figure S1c). Similarly, in BpinBdan-Endo-TS (Figure S1d), the distance between hydrogen and oxygen is 2.35 Å. The structure is more stable than BpinBdan-Exo-TS (Figure S1e), where the acidic allylic hydrogen interacts with nitrogen, which is less electronegative than oxygen. We cannot compare BpinBpin-TS with BpinBdan-TS as the steric effect comes into play.

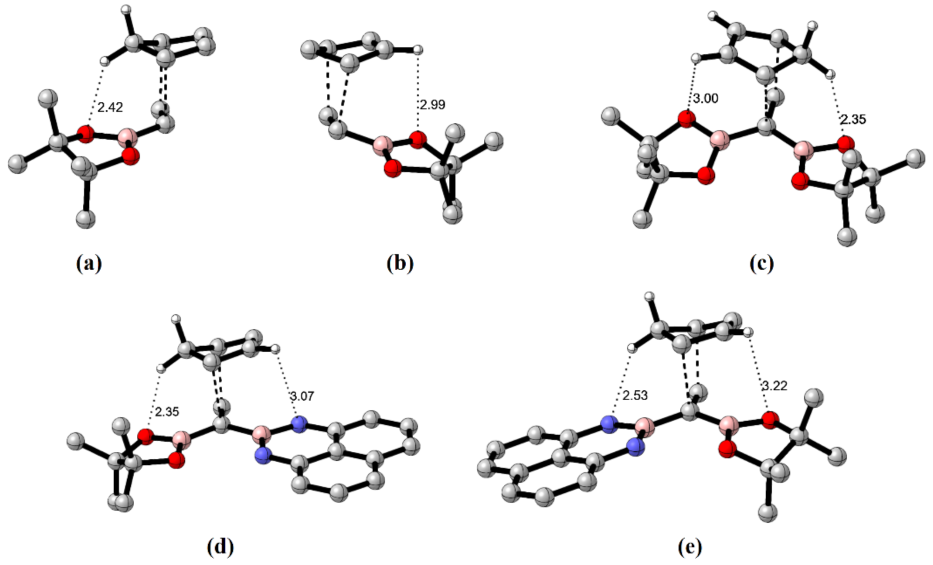

Figure S1: Different hydrogen/oxygen and hydrogen/nitrogen distances given in Å for different transition states.

Cartesian coordinates of the structures reported in the manuscript:

For reactants:

XYZ-Coordinates for the Cyclopentadiene (3e)

C	0.000000000	0.733800000	-0.986618000
C	0.000000000	-0.733800000	-0.986618000
C	0.000000000	-1.175194000	0.280070000
C	0.000000000	0.000000000	1.213060000
C	0.000000000	1.175194000	0.280070000
H	0.000000000	1.349328000	-1.878236000
H	0.000000000	-1.349328000	-1.878236000
H	0.000000000	-2.207238000	0.606323000
H	-0.877892000	0.000000000	1.872022000
H	0.877892000	0.000000000	1.872022000
H	0.000000000	2.207238000	0.606323000

XYZ-Coordinates for the 1-boron-diene (*E*-3k)

C	2.813764000	0.083277000	0.088951000
C	3.626133000	1.336258000	-0.160938000
C	5.105821000	1.149637000	0.171614000
C	5.618376000	-0.164607000	-0.414965000
C	4.877578000	-1.344444000	0.214234000
C	3.394790000	-1.105434000	0.289744000
H	3.203609000	2.157083000	0.428474000
H	3.509756000	1.633054000	-1.210811000
H	5.686050000	1.996627000	-0.202591000

H	5.232281000	1.129612000	1.259933000
H	6.695259000	-0.267998000	-0.261113000
H	5.447098000	-0.164473000	-1.497165000
H	5.067181000	-2.262008000	-0.352303000
H	5.264033000	-1.536011000	1.223815000
H	2.767119000	-1.957662000	0.539328000
C	1.350395000	0.246504000	0.128419000
C	0.439843000	-0.647377000	-0.282278000
H	0.990278000	1.201343000	0.513814000
H	0.780693000	-1.583356000	-0.720528000
C	-3.717028000	1.487762000	1.261410000
H	-4.799889000	1.435769000	1.119077000
H	-3.430577000	2.539553000	1.313982000
H	-3.465249000	1.020933000	2.212898000
O	-1.576369000	0.797871000	0.390082000
B	-1.071145000	-0.359039000	-0.155120000
O	-2.057918000	-1.215667000	-0.572607000
H	-2.829317000	-1.188497000	1.977004000
C	-3.601677000	-1.407978000	1.236732000
H	-3.605777000	-2.484790000	1.061320000
H	-4.571211000	-1.118090000	1.647463000
C	-3.312434000	-0.696937000	-0.082725000
H	-4.134641000	-0.636173000	-2.088444000
C	-2.990714000	0.820498000	0.106658000
C	-3.175480000	1.627423000	-1.175978000
H	-2.738905000	2.617146000	-1.033657000
H	-4.230917000	1.745590000	-1.430697000
H	-2.665392000	1.150400000	-2.015666000
H	-5.347261000	-0.535908000	-0.796525000

H	-4.555077000	-2.076401000	-1.158950000
C	-4.402554000	-0.997611000	-1.096355000

XYZ-Coordinates for the Vinylboron (1)

B	1.230788000	-0.347310000	-0.077341000
O	0.707340000	0.859163000	0.311865000
O	0.266053000	-1.264555000	-0.398868000
C	-1.007434000	-0.708488000	-0.007997000
C	-0.705253000	0.829805000	0.020598000
C	-1.446886000	1.606193000	1.095922000
H	-1.202314000	1.243856000	2.094354000
H	-1.171214000	2.661688000	1.044572000
H	-2.528719000	1.530832000	0.952000000
C	-1.335805000	-1.280918000	1.369642000
H	-2.320618000	-0.959287000	1.717271000
H	-1.330655000	-2.371082000	1.309725000
H	-0.591898000	-0.983126000	2.112195000
C	2.751252000	-0.639141000	-0.147478000
H	3.085833000	-1.636753000	-0.427134000
C	3.665403000	0.291178000	0.126416000
H	3.373879000	1.299465000	0.408812000
H	4.733274000	0.091519000	0.082593000
C	-2.061796000	-1.134070000	-1.016124000
H	-1.771350000	-0.877507000	-2.034847000
H	-2.200973000	-2.216219000	-0.969067000
H	-1.948610000	1.579681000	-1.609396000
C	-0.892911000	1.503115000	-1.337755000
H	-0.370516000	0.958543000	-2.127736000

H	-3.022193000	-0.658547000	-0.796405000
H	-0.474425000	2.510591000	-1.293562000

XYZ-Coordinates for the *gem*-diborylalkene (2)

B	-0.182109000	1.353441000	0.688478000
O	0.000000000	2.573921000	1.292868000
O	-0.576229000	1.470453000	-0.617412000
C	-0.448195000	2.856616000	-0.989073000
C	-0.515549000	3.580783000	0.399101000
C	-1.944036000	3.884710000	0.847272000
H	-2.581077000	3.000410000	0.770871000
H	-1.927841000	4.196001000	1.893804000
H	-2.392487000	4.686963000	0.256008000
C	-1.568079000	3.214484000	-1.951922000
H	-1.550438000	4.282533000	-2.188315000
H	-1.443327000	2.658603000	-2.883633000
H	-2.546339000	2.964810000	-1.540908000
C	0.000000000	0.000000000	1.431503000
C	0.000000000	0.000000000	2.772488000
H	0.103585000	-0.918488000	3.346855000
C	0.344755000	4.828354000	0.511279000
H	0.241194000	5.259619000	1.509370000
H	1.399257000	4.604463000	0.350294000
H	0.032226000	5.581569000	-0.218036000
C	0.906507000	3.005352000	-1.679283000
H	1.726936000	2.760889000	-1.000708000
H	0.951361000	2.308998000	-2.518820000
H	1.059383000	4.018746000	-2.059028000
B	0.182109000	-1.353441000	0.688478000

O	0.576229000	-1.470453000	-0.617412000
C	0.448195000	-2.856616000	-0.989073000
C	-0.906507000	-3.005352000	-1.679283000
H	-0.951361000	-2.308998000	-2.518820000
H	1.443327000	-2.658603000	-2.883633000
H	1.550438000	-4.282533000	-2.188315000
H	2.546339000	-2.964810000	-1.540908000
C	1.568079000	-3.214484000	-1.951922000
H	-1.059383000	-4.018746000	-2.059028000
H	-1.726936000	-2.760889000	-1.000708000
C	0.515549000	-3.580783000	0.399101000
H	-0.032226000	-5.581569000	-0.218036000
C	-0.344755000	-4.828354000	0.511279000
H	-1.399257000	-4.604463000	0.350294000
H	-0.241194000	-5.259619000	1.509370000
C	1.944036000	-3.884710000	0.847272000
H	2.392487000	-4.686963000	0.256008000
H	2.581077000	-3.000410000	0.770871000
H	1.927841000	-4.196001000	1.893804000
O	0.000000000	-2.573921000	1.292868000
H	-0.103585000	0.918488000	3.346855000

XYZ-Coordinates for the 1,1 BpinBdan-ethene (2’)

C	1.063747000	-3.004023000	-0.428398000
H	2.055007000	-3.447350000	-0.491742000
C	0.884208000	-1.696330000	-0.195311000
B	-0.550058000	-1.059394000	-0.123199000
N	-0.738174000	0.342713000	-0.234300000
N	-1.732878000	-1.830830000	0.056752000

H	-1.688896000	-2.827034000	0.194321000
H	0.065216000	0.946050000	-0.329522000
H	-5.510747000	2.599060000	-0.059652000
C	-4.528001000	2.140196000	-0.096012000
C	-3.404929000	2.906031000	-0.260692000
C	-2.126071000	2.324692000	-0.309821000
C	-1.981797000	0.958137000	-0.191676000
C	-3.134508000	0.135854000	-0.019959000
C	-4.421549000	0.732180000	0.030191000
C	-5.555650000	-0.100395000	0.206873000
C	-5.405338000	-1.455408000	0.329122000
C	-4.134579000	-2.055343000	0.281519000
C	-3.008350000	-1.278940000	0.108674000
H	-3.495436000	3.983629000	-0.356475000
H	-1.247307000	2.948670000	-0.442463000
H	-4.038180000	-3.132843000	0.379628000
H	-6.278261000	-2.086186000	0.465325000
H	-6.540819000	0.353076000	0.244844000
B	2.162865000	-0.820117000	-0.058175000
O	2.144694000	0.555961000	-0.108003000
C	3.461658000	1.027234000	0.257078000
C	4.353715000	-0.218845000	-0.070617000
O	3.429172000	-1.313830000	0.104110000
C	5.535053000	-0.418584000	0.863435000
H	5.213312000	-0.556135000	1.895614000
H	6.094474000	-1.307446000	0.564532000
H	6.212686000	0.438998000	0.819727000
H	4.809423000	2.598402000	-0.367527000
H	3.114157000	3.086594000	-0.257746000
H	3.656377000	2.102995000	-1.620187000

C	3.781457000	2.273069000	-0.550895000
C	4.817817000	-0.257362000	-1.525151000
H	5.572635000	0.505881000	-1.729056000
H	5.254535000	-1.236537000	-1.731026000
H	3.982874000	-0.111863000	-2.214601000
C	3.414418000	1.359049000	1.746658000
H	3.188612000	0.473737000	2.345339000
H	4.359851000	1.781224000	2.095419000
H	2.624891000	2.092319000	1.923280000
H	0.237935000	-3.698263000	-0.585189000

For transition States:

XYZ-Coordinates for the Bpin-Exo-TS

B	-0.131172000	-0.469691000	-0.593524000
O	-0.670486000	0.796278000	-0.718862000
O	-1.012139000	-1.346827000	-0.002469000
C	-2.288010000	-0.687600000	0.092854000
C	-1.885882000	0.826458000	0.050816000
C	-1.532615000	1.388983000	1.427595000
H	-0.811326000	0.752223000	1.945711000
H	-1.079023000	2.374758000	1.303041000
H	-2.415810000	1.496104000	2.062022000
C	-2.976285000	-1.129149000	1.374155000
H	-3.911544000	-0.581157000	1.522427000
H	-3.212798000	-2.193817000	1.316921000
H	-2.340178000	-0.974095000	2.245867000
C	1.289763000	-0.831456000	-1.034100000
H	1.574241000	-1.878527000	-0.999271000
C	2.110000000	0.041318000	-1.740263000

H	1.709575000	1.002041000	-2.051190000
H	2.888529000	-0.347383000	-2.386929000
C	4.108863000	-0.244088000	0.125925000
C	3.391054000	0.882407000	-0.282739000
H	3.835557000	1.717756000	-0.811850000
H	5.070900000	-0.547952000	-0.269080000
C	2.268717000	1.056368000	0.707598000
H	2.659894000	1.633375000	1.559344000
H	1.375917000	1.554921000	0.336498000
C	2.089174000	-0.364089000	1.153186000
C	3.303020000	-1.012200000	0.973761000
H	3.538618000	-2.006307000	1.333820000
H	1.303822000	-0.697006000	1.820577000
C	-2.887870000	1.735533000	-0.641305000
H	-3.036763000	1.448384000	-1.682116000
H	-3.854556000	1.709776000	-0.129648000
H	-2.523965000	2.765316000	-0.626095000
C	-3.109733000	-1.120313000	-1.120355000
H	-2.635457000	-0.806015000	-2.052949000
H	-3.176649000	-2.210090000	-1.130298000
H	-4.123311000	-0.712495000	-1.091301000

XYZ-Coordinates for the Bpin-Endo-TS

C	1.327844000	-0.088376000	-1.439641000
C	2.197723000	-1.164556000	-1.299549000
H	2.975321000	-1.325679000	-2.041329000
H	1.843520000	-2.071444000	-0.822975000
C	2.784478000	-0.634703000	1.322603000

C	3.570564000	-0.567236000	0.169430000
H	4.417827000	-1.218872000	-0.012320000
H	2.718537000	-1.490057000	1.983537000
C	3.550016000	0.875261000	-0.270220000
H	4.333972000	1.407683000	0.289590000
H	3.711111000	1.054794000	-1.331614000
C	2.218885000	1.299080000	0.280546000
C	1.954313000	0.489227000	1.375020000
H	1.149205000	0.631127000	2.084335000
H	1.767487000	2.265275000	0.093602000
B	-0.068576000	-0.059355000	-0.815863000
O	-0.512627000	-0.979941000	0.108842000
H	-2.334408000	-2.769087000	-0.311377000
H	-2.430095000	-1.601417000	-1.630931000
C	-2.659337000	-1.760382000	-0.574756000
O	-1.027767000	0.884818000	-1.110322000
C	-1.919055000	-0.757411000	0.308795000
C	-2.098743000	0.728362000	-0.163814000
H	-3.742438000	-1.700553000	-0.440969000
H	-3.545776000	0.412621000	-1.753117000
C	-3.419249000	1.021579000	-0.858052000
H	-3.451424000	2.070925000	-1.159386000
H	-4.262513000	0.835128000	-0.186316000
H	-1.813473000	2.745404000	0.511846000
C	-1.860938000	1.746671000	0.950846000
C	-2.253200000	-1.005524000	1.771079000
H	-2.089066000	-2.058073000	2.012244000
H	-0.912900000	1.561975000	1.460682000
H	-1.625196000	-0.408769000	2.433084000
H	-3.301162000	-0.769145000	1.978056000

H	-2.662102000	1.734082000	1.694013000
H	1.559793000	0.659886000	-2.193218000

XYZ-Coordinates for the gemBpinBpin-TS

C	-0.016244000	1.796355000	-1.550948000
H	-0.928528000	2.030896000	-2.089711000
C	0.028370000	0.639775000	-0.755123000
B	1.387642000	-0.004925000	-0.446780000
O	1.592460000	-1.129927000	0.318649000
C	2.999234000	-1.228810000	0.587756000
C	3.240623000	-0.565492000	1.944246000
H	2.980149000	0.496026000	1.918531000
H	2.605095000	-1.048661000	2.689366000
H	4.281205000	-0.657189000	2.265628000
C	3.625845000	-0.426383000	-0.601176000
O	2.586742000	0.519463000	-0.905028000
H	4.737868000	1.060305000	0.528820000
C	4.901023000	0.328841000	-0.263231000
H	5.687940000	-0.360502000	0.056973000
H	5.258367000	0.863578000	-1.146154000
H	-2.048806000	1.997510000	1.903393000
C	-1.199508000	2.371038000	1.346778000
C	0.097239000	1.902023000	1.445406000
H	0.452403000	1.176438000	2.165846000
H	1.884747000	2.412268000	0.251415000

C	0.979055000	2.837202000	0.677446000
H	1.269146000	3.655908000	1.353521000
H	0.241810000	4.201937000	-0.990398000
C	-0.016502000	3.396726000	-0.311345000
C	-1.269474000	3.305807000	0.307948000
H	-2.180969000	3.761566000	-0.057495000
C	3.836242000	-1.279336000	-1.851201000
H	4.658027000	-1.988910000	-1.725367000
H	2.931970000	-1.836796000	-2.105787000
H	4.072632000	-0.623026000	-2.691372000
C	3.390728000	-2.696047000	0.647944000
H	2.905276000	-3.171385000	1.503046000
H	3.080025000	-3.229198000	-0.250473000
H	4.472998000	-2.806812000	0.765220000
B	-1.299232000	-0.061791000	-0.431520000
O	-2.526433000	0.501745000	-0.729973000
O	-1.437191000	-1.306613000	0.137730000
H	-2.776404000	-0.125333000	2.112798000
C	-3.021909000	-1.169951000	1.912003000
H	-2.347659000	-1.788763000	2.507397000
C	-2.825701000	-1.511335000	0.435239000
H	-4.046996000	-1.355379000	2.242879000
H	-2.894717000	-3.291743000	-0.808202000
C	-3.169602000	-2.973717000	0.197403000
H	-4.239599000	-3.152185000	0.341023000
H	-2.622272000	-3.598470000	0.906542000
C	-3.534865000	-0.502200000	-0.535231000
H	-4.564242000	0.725412000	0.932797000
C	-4.783890000	0.156236000	0.029068000
H	-5.547288000	-0.590994000	0.265303000

H	-4.636527000	-1.844820000	-1.862907000
H	-4.144672000	-0.301826000	-2.583280000
C	-3.835483000	-1.102548000	-1.908031000
H	-2.947654000	-1.574564000	-2.334924000
H	-5.201609000	0.845798000	-0.708037000
H	0.890518000	2.106046000	-2.065619000

XYZ-Coordinates for the endo-BpinBdan-TS

C	-0.999695000	2.301383000	-1.221715000
H	-1.989619000	2.565431000	-1.586224000
C	-0.793776000	1.008278000	-0.717638000
B	0.641455000	0.430990000	-0.569077000
N	1.810237000	1.196025000	-0.871053000
N	0.892106000	-0.890656000	-0.094960000
H	0.111136000	-1.491313000	0.122561000
H	1.733804000	2.143978000	-1.200198000
H	6.707823000	-0.620621000	-0.088537000
C	5.703588000	-0.241647000	-0.248795000
C	5.503002000	1.028738000	-0.717927000
C	4.207518000	1.530682000	-0.932718000
C	3.106014000	0.741824000	-0.670431000
C	3.285338000	-0.584242000	-0.176607000
C	4.597578000	-1.083141000	0.034198000
C	4.757169000	-2.405184000	0.519665000
C	3.658650000	-3.181513000	0.778552000
C	2.356023000	-2.695736000	0.576464000
C	2.157795000	-1.412241000	0.106479000
H	6.355059000	1.666492000	-0.932452000

H	4.070743000	2.540092000	-1.310608000
H	1.498619000	-3.327369000	0.789181000
H	3.788940000	-4.193981000	1.148617000
H	5.758865000	-2.790357000	0.680818000
H	-2.269648000	3.279397000	2.086664000
C	-0.907350000	1.707722000	1.712628000
C	-1.935191000	2.680184000	1.226253000
H	-1.129291000	0.798575000	2.256607000
H	-2.814161000	2.255378000	0.746963000
C	0.323948000	2.336188000	1.678964000
H	1.243615000	1.937337000	2.087228000
C	0.219143000	3.494833000	0.899918000
H	1.043824000	4.148269000	0.640610000
C	-1.073846000	3.572330000	0.363250000
H	-1.463785000	4.475420000	-0.093799000
B	-2.018073000	0.108852000	-0.501085000
O	-3.325082000	0.499306000	-0.714975000
C	-4.161550000	-0.668602000	-0.615588000
O	-1.956611000	-1.202407000	-0.056719000
C	-3.294543000	-1.620383000	0.274206000
C	-3.443318000	-3.099986000	-0.035689000
H	-4.477442000	-3.424698000	0.111295000
H	-2.808482000	-3.685253000	0.633496000
H	-3.149776000	-3.325979000	-1.060691000
H	-5.369459000	0.254564000	0.937722000
H	-6.028226000	0.394856000	-0.693487000
H	-6.123536000	-1.150337000	0.161836000
C	-5.496870000	-0.270249000	-0.009415000
H	-0.212014000	2.809758000	-1.772380000
C	-3.482529000	-1.371298000	1.770103000

H	-2.702822000	-1.902883000	2.319916000
H	-3.398766000	-0.308159000	2.009663000
H	-4.453676000	-1.727305000	2.122073000
C	-4.362944000	-1.197824000	-2.034184000
H	-5.044195000	-2.052009000	-2.055520000
H	-4.788043000	-0.403142000	-2.650468000
H	-3.414184000	-1.498813000	-2.484289000

XYZ-Coordinates for the exo-BpinBdan-TS

C	1.049581000	2.253358000	-1.210901000
H	0.262993000	2.831981000	-1.694503000
C	0.746746000	1.023866000	-0.607996000
H	0.044793000	3.830782000	1.967075000
C	0.878049000	1.905234000	1.759612000
C	0.201243000	3.097007000	1.162018000
H	0.373521000	1.153103000	2.351556000
H	-0.760542000	2.911498000	0.690087000
C	2.240153000	2.141494000	1.736430000
H	2.990511000	1.541463000	2.232472000
C	2.504891000	3.205624000	0.866361000
H	3.491797000	3.548609000	0.582114000
C	1.305513000	3.622593000	0.276077000
H	1.215950000	4.560468000	-0.261253000
B	-0.717150000	0.527964000	-0.428494000
N	-1.019574000	-0.713465000	0.204170000
H	-0.259489000	-1.309939000	0.496347000
H	-1.750223000	2.124179000	-1.393015000
N	-1.859070000	1.270184000	-0.871197000

C	-3.172005000	0.842882000	-0.710915000
C	-3.399126000	-0.408467000	-0.066485000
C	-2.302865000	-1.199273000	0.390092000
C	-2.545484000	-2.412670000	1.002298000
H	-1.711118000	-3.014544000	1.350017000
C	-3.864344000	-2.865273000	1.176720000
H	-4.030297000	-3.822418000	1.661734000
C	-4.934051000	-2.124605000	0.748867000
H	-5.948546000	-2.483697000	0.888754000
C	-4.727701000	-0.874858000	0.112559000
C	-5.802357000	-0.076339000	-0.354878000
H	-6.819238000	-0.430155000	-0.218751000
C	-5.555956000	1.120696000	-0.971555000
H	-6.383831000	1.725415000	-1.328733000
C	-4.243296000	1.589907000	-1.154813000
H	-4.070522000	2.543016000	-1.646850000
B	1.913694000	0.044607000	-0.405344000
O	1.790781000	-1.230840000	0.127441000
O	3.209328000	0.296273000	-0.798249000
C	2.979230000	-1.964953000	-0.224227000
C	4.031122000	-0.816591000	-0.399182000
H	1.800792000	-3.337334000	-1.381861000
C	2.677062000	-2.702419000	-1.528055000
H	2.450292000	-2.003816000	-2.336726000
H	3.512165000	-3.334604000	-1.839495000
H	5.654159000	-1.964478000	-1.260232000
C	5.071563000	-1.064869000	-1.479482000
H	4.610980000	-1.178437000	-2.460755000
H	5.760662000	-0.218771000	-1.527732000
C	3.294029000	-2.962362000	0.878064000

H	3.351730000	-2.481929000	1.854742000
H	2.511070000	-3.722750000	0.923090000
H	4.243582000	-3.468781000	0.682311000
C	4.721246000	-0.439999000	0.909689000
H	4.000362000	-0.321053000	1.719904000
H	5.451793000	-1.194849000	1.210833000
H	5.241972000	0.510576000	0.775930000
H	2.012152000	2.383232000	-1.694004000

XYZ-Coordinates for the 1,1,2-triBpin-TS

B	-0.667176000	1.616397000	1.005713000
O	-0.984040000	2.947334000	0.810795000
O	-1.786347000	0.856202000	1.263938000
C	-2.885471000	1.763277000	1.483482000
C	-2.418549000	3.048476000	0.725951000
C	-2.858723000	4.357033000	1.360224000
H	-2.464578000	4.463217000	2.370364000
H	-2.490557000	5.193108000	0.762782000
H	-3.949625000	4.420852000	1.399280000
C	-2.995406000	1.969069000	2.991822000
H	-3.846338000	2.602148000	3.253053000
H	-3.124152000	0.996435000	3.469253000
H	-2.086129000	2.422915000	3.392044000
C	0.772432000	1.100980000	0.883240000
C	1.791883000	2.052569000	0.712889000
H	2.803838000	1.782015000	1.002722000
C	-2.786800000	3.026421000	-0.755909000

H	-2.235434000	3.820682000	-1.263281000
H	-2.527568000	2.071607000	-1.218424000
H	-3.855160000	3.199583000	-0.905774000
C	-4.155233000	1.139743000	0.933705000
H	-4.021933000	0.850715000	-0.107724000
H	-4.404727000	0.246775000	1.512264000
H	-4.994140000	1.837458000	1.007430000
B	1.236303000	-0.338995000	1.170897000
O	0.501679000	-1.372481000	1.705002000
C	1.430907000	-2.403034000	2.094645000
C	1.742687000	-2.179553000	3.572926000
H	0.806314000	-2.191270000	4.132907000
H	-0.050816000	-3.886207000	2.571639000
H	1.516812000	-4.561515000	2.107272000
H	0.438807000	-3.887552000	0.869691000
C	0.795870000	-3.767803000	1.892259000
H	2.398669000	-2.956418000	3.972162000
H	2.216263000	-1.207729000	3.728721000
C	2.657100000	-2.123373000	1.168129000
H	4.082395000	-3.487705000	2.058815000
C	4.009874000	-2.430656000	1.788384000
H	4.187142000	-1.827790000	2.678509000
H	4.799616000	-2.211004000	1.066915000
C	2.529451000	-2.802784000	-0.193185000
H	2.691171000	-3.880929000	-0.117808000
H	1.544262000	-2.624183000	-0.629191000
H	3.286817000	-2.389508000	-0.863628000
O	2.554061000	-0.704008000	0.955181000
H	1.537988000	3.097539000	0.870222000

C	2.496780000	1.141727000	-1.746101000
C	3.906704000	0.615804000	-1.899205000
C	4.938451000	1.721276000	-2.112327000
C	4.770993000	2.799407000	-1.043518000
C	3.387867000	3.439088000	-1.155042000
C	2.270585000	2.418267000	-1.226633000
H	3.933225000	-0.108728000	-2.718907000
H	4.152517000	0.060049000	-0.984204000
H	5.947776000	1.302696000	-2.086233000
H	4.802210000	2.169223000	-3.103369000
H	5.542777000	3.567584000	-1.138027000
H	4.894224000	2.346228000	-0.053317000
H	3.212831000	4.127832000	-0.323558000
H	3.347489000	4.049189000	-2.066183000
H	1.289785000	2.852131000	-1.396625000
C	1.433159000	0.229226000	-1.840929000
C	0.145874000	0.513714000	-1.436504000
H	1.685626000	-0.804687000	-2.067978000
H	-0.175360000	1.550973000	-1.440426000
C	-1.875094000	-3.978698000	-1.606449000
H	-2.855568000	-4.460813000	-1.648196000
H	-1.244725000	-4.556349000	-0.927428000
H	-1.425447000	-4.010979000	-2.598702000
O	-0.711551000	-1.903146000	-1.202249000
B	-0.960524000	-0.573194000	-1.432584000
O	-2.279828000	-0.331632000	-1.742332000
H	-1.716650000	-1.974025000	-3.765880000
C	-2.765218000	-1.913123000	-3.466317000
H	-3.230542000	-1.099335000	-4.024692000

H	-3.259996000	-2.848147000	-3.737635000
C	-2.892676000	-1.623155000	-1.972478000
H	-4.482210000	-1.291586000	-0.529199000
C	-2.000928000	-2.554204000	-1.098716000
C	-2.406775000	-2.523116000	0.371568000
H	-1.631958000	-3.003396000	0.966523000
H	-3.351277000	-3.049253000	0.531028000
H	-2.492419000	-1.494607000	0.730835000
H	-4.816630000	-2.559963000	-1.717891000
H	-4.885999000	-0.858013000	-2.195214000
C	-4.355317000	-1.579655000	-1.570534000

XYZ-Coordinates for the 1,1,3-triBpin-TS

B	1.339941000	1.587775000	-0.328348000
O	0.657589000	2.783736000	-0.171329000
O	2.697063000	1.791359000	-0.445418000
C	2.956169000	3.187303000	-0.196955000
C	1.572907000	3.842992000	-0.506696000
C	1.385254000	4.143058000	-1.992491000
H	1.628145000	3.269138000	-2.601166000
H	0.339062000	4.397548000	-2.169408000
H	2.007329000	4.979420000	-2.318912000
C	4.096221000	3.641669000	-1.092024000
H	4.254673000	4.719898000	-1.002032000
H	5.016374000	3.135225000	-0.795118000
H	3.900551000	3.401097000	-2.136394000
C	0.611211000	0.240061000	-0.380703000
C	-0.781470000	0.282671000	-0.602795000

H	-1.248430000	-0.587950000	-1.055588000
C	1.242980000	5.071747000	0.323138000
H	0.262326000	5.453550000	0.033819000
H	1.213506000	4.839946000	1.387153000
H	1.980021000	5.861984000	0.155600000
C	3.347548000	3.326819000	1.270984000
H	2.516313000	3.050895000	1.923271000
H	4.181769000	2.654935000	1.480487000
H	3.650871000	4.348404000	1.510817000
B	1.313210000	-1.093840000	-0.689316000
O	2.642772000	-1.401889000	-0.478213000
C	2.957020000	-2.539705000	-1.303964000
C	3.516360000	-1.981165000	-2.611405000
H	4.374620000	-1.348986000	-2.379241000
H	4.927679000	-2.819374000	-0.503425000
H	4.227346000	-4.285119000	-1.205700000
H	3.684559000	-3.710269000	0.379580000
C	4.005954000	-3.393731000	-0.612253000
H	3.835747000	-2.773975000	-3.291331000
H	2.774139000	-1.361538000	-3.119539000
C	1.561478000	-3.228703000	-1.484069000
H	2.074542000	-4.636338000	-3.048293000
C	1.337869000	-3.851972000	-2.853718000
H	1.400497000	-3.107459000	-3.646271000
H	0.343987000	-4.301542000	-2.890029000
C	1.241273000	-4.250752000	-0.397267000
H	1.848442000	-5.153230000	-0.499246000
H	1.400645000	-3.834426000	0.597377000
H	0.188868000	-4.528188000	-0.479807000

O	0.652109000	-2.127387000	-1.323803000
H	-1.230892000	1.232459000	-0.881383000
C	0.043139000	-0.947508000	2.278222000
C	0.545613000	-2.259509000	2.853620000
C	2.069762000	-2.371682000	2.838168000
C	2.699305000	-1.090127000	3.373721000
C	2.371972000	0.073355000	2.435755000
C	0.911771000	0.110472000	2.056399000
H	0.087734000	-3.095643000	2.316244000
H	0.193061000	-2.337699000	3.889400000
H	2.387021000	-3.243623000	3.416764000
H	2.423905000	-2.508464000	1.813062000
H	3.782903000	-1.198060000	3.465632000
H	2.309785000	-0.880480000	4.376654000
H	2.642262000	1.022809000	2.904855000
H	2.978605000	-0.000124000	1.525752000
H	0.482729000	1.096630000	1.928126000
C	-1.281466000	-0.855922000	1.834111000
C	-1.793617000	0.248216000	1.149431000
H	-1.880222000	-1.765680000	1.842190000
H	-1.405107000	1.232428000	1.397328000
C	-5.811349000	-1.657592000	-0.923784000
H	-6.782234000	-1.360772000	-1.329804000
H	-5.939443000	-2.599925000	-0.388390000
H	-5.122521000	-1.829229000	-1.750034000
O	-3.947352000	-0.964925000	0.440819000
B	-3.235821000	0.199513000	0.593090000
O	-3.913068000	1.296082000	0.123445000
H	-3.803962000	0.023113000	-2.207573000

C	-4.630579000	0.725562000	-2.077248000
H	-4.288772000	1.710803000	-2.398196000
H	-5.456441000	0.416133000	-2.721429000
C	-5.054355000	0.806695000	-0.612874000
H	-6.416300000	1.974124000	0.605256000
C	-5.281911000	-0.600054000	0.028739000
C	-6.137902000	-0.542159000	1.290967000
H	-6.080405000	-1.506203000	1.798627000
H	-7.184452000	-0.332388000	1.059723000
H	-5.771748000	0.224189000	1.977532000
H	-7.113482000	1.390381000	-0.918901000
H	-5.955632000	2.728941000	-0.922254000
C	-6.207747000	1.779895000	-0.446164000

References:

1. Shimizu, M.; Kurahashi, T.; Shimono, K.; Tanaka, K.; Nagao, I.; Kiyomoto, S. i.; Hiyama, T., Facile Synthesis and Palladium-Catalyzed Cross-Coupling Reactions of 2, 3-Bis (pinacolatoboryl)-1, 3-butadiene. *J. Chem. Asia.* **2007,** *2,* 1400-1408.

2. Wang, Y.; Guan, R.; Sivaguru, P.; Cong, X.; Bi, X., Silver-Catalyzed anti-Markovnikov Hydroboration of C–C Multiple Bonds. *Org. Lett.* **2019,** *21,* 4035-4038.

3. Legault, C. Y. C., Available online: http://www.cylview.org *(accessed on 11 February),* Université de Sherbrooke.: 2018.

4. Kumar, N.; Eghbarieh, N.; Stein, T.; Shames, A. I.; Masarwa, A., Photoredox-Mediated Reaction of gem-Diborylalkenes: Reactivity Toward Diverse 1, 1-Bisborylalkanes. *Chem. Eur. J.* **2020,** *26*, 5360-5364.

5. Yao, H.; Richardson, D. E., Epoxidation of alkenes with bicarbonate-activated hydrogen peroxide. *J. Am. Chem. Soc.* **2000,** *122*, 3220-3221.

6. Lee, H.; Lee, Y.; Cho, S. H., Palladium-catalyzed chemoselective negishi cross-coupling of bis [(pinacolato) boryl] methylzinc halides with aryl (pseudo) halides. *Org. lett.* **2019,** *21*, 5912-5916.

7. Quast, H.; Schön, N., 1-Aza-und 1, 9-Diazatriptycene. *J. Liebig. Annalen. Der. Chem.* **1984,** *1984*, 381-388.

8. Olbrich, M.; Mayer, P.; Trauner, D., A step toward polytwistane: synthesis and characterization of C-2-symmetric tritwistane. *Org. Biomol. Chem.* **2014,** *12*, 108-112.

9. Jiang, G.-J.; Fu, X.-F.; Li, Q.; Yu, Z.-X., Rh (I)-Catalyzed [5+ 1] Cycloaddition of Vinylcyclopropanes and CO for the Synthesis of α, β-and β, γ-Cyclohexenones. *Org. lett.* **2012,** *14*, 692-695.

10. Kumar, N.; Reddy, R. R.; Masarwa, A., Stereoselective Desymmetrization of gem-Diborylalkanes by “Trifluorination”. *Chem. Eur. J.* **2019,** *25*, 8008-8012.

11. Autenrieth, B.; Jeong, H.; Forrest, W. P.; Axtell, J. C.; Ota, A.; Lehr, T.; Buchmeiser, M. R.; Schrock, R. R., Stereospecific Ring-Opening Metathesis Polymerization (ROMP) of endo-Dicyclopentadiene by Molybdenum and Tungsten Catalysts. *Macromolecules.* **2015,** *48,* 2480-2492.

12. Frisch, M. J.; Trucks, G. W.; Schlegel, H. B.; Scuseria, G. E.; Robb, M. A.; Cheeseman, J. R.; Scalmani, G.; Barone, V.; Petersson, G. A.; Nakatsuji, H.; Li, X.; Caricato, M.; Marenich, A. V.; Bloino, J.; Janesko, B. G.; Gomperts, R.; Mennucci, B.; Hratchian, H. P.; Ortiz, J. V.; Izmaylov, A. F.; Sonnenberg, J. L.; Williams; Ding, F.; Lipparini, F.; Egidi, F.; Goings, J.; Peng, B.; Petrone, A.; Henderson, T.; Ranasinghe, D.; Zakrzewski, V. G.; Gao, J.; Rega, N.; Zheng, G.; Liang, W.; Hada, M.; Ehara, M.; Toyota, K.; Fukuda, R.; Hasegawa, J.; Ishida, M.; Nakajima, T.; Honda, Y.; Kitao, O.; Nakai, H.; Vreven, T.; Throssell, K.; Montgomery Jr., J. A.; Peralta, J. E.; Ogliaro, F.; Bearpark, M. J.; Heyd, J. J.; Brothers, E. N.; Kudin, K. N.; Staroverov, V. N.; Keith, T. A.; Kobayashi, R.; Normand, J.; Raghavachari, K.; Rendell, A. P.; Burant, J. C.; Iyengar, S. S.; Tomasi, J.; Cossi, M.; Millam, J. M.; Klene, M.; Adamo, C.; Cammi, R.; Ochterski, J. W.; Martin, R. L.; Morokuma, K.; Farkas, O.; Foresman, J. B.; Fox, D. J. *Gaussian 16 Rev. C.01*, Wallingford, CT, 2016.
13. Zhao, Y.; Truhlar, D. G., The M06 suite of density functionals for main group thermochemistry, thermochemical kinetics, noncovalent interactions, excited states, and transition elements: two new functionals and systematic testing of four M06-class functionals and 12 other functionals. *J. Theory. Chem. Accou.* **2008,** *120,* 215-241.
14. Frisch, M. J.; Pople, J. A.; Binkley, J. S., Self-consistent molecular orbital methods 25. Supplementary functions for Gaussian basis sets. *J. Chem. Phys.* **1984,** *80,* 3265-3269.
15. Marenich, A. V.; Cramer, C. J.; Truhlar, D. G., Universal solvation model based on solute electron density and on a continuum model of the solvent defined by the bulk dielectric constant and atomic surface tensions. *J. B. Phys. Chem.* **2009,** *113,* 6378-6396.
16. Ho, J.; Klamt, A.; Coote, M. L., Comment on the correct use of continuum solvent models. *J. Phys. Chem.* **2010,** *114,* 13442-13444.
17. Grimme, S., Supramolecular binding thermodynamics by dispersion-corrected density functional theory. *Chem. Eur. J.* **2012,** *18,* 9955-9964.
18. Luchini., G. bobbypaton/GoodVibes.
19. Chirlian, L. E.; Francl, M. M., Atomic Charges Derived from Electrostatic Potentials - a Detailed Study. *J. Comp. Chem.* **1987,** *8,* 894-905.
20. Niebel, C.; Lokshin, V.; Sigalov, M.; Krief, P.; Khodorkovsky, V., Intra-and Intermolecular C (sp2)–H··· O Hydrogen Bonds in a Series of Isobenzofuranone Derivatives: Manifestation and Energetics. *Eur. J. Org. Chem.* **2008**, *2008*, 3689–3699

9. NMR spectra Reprint

^{1}H NMR (400 MHz, $CDCl_3$) of compound (**2**)

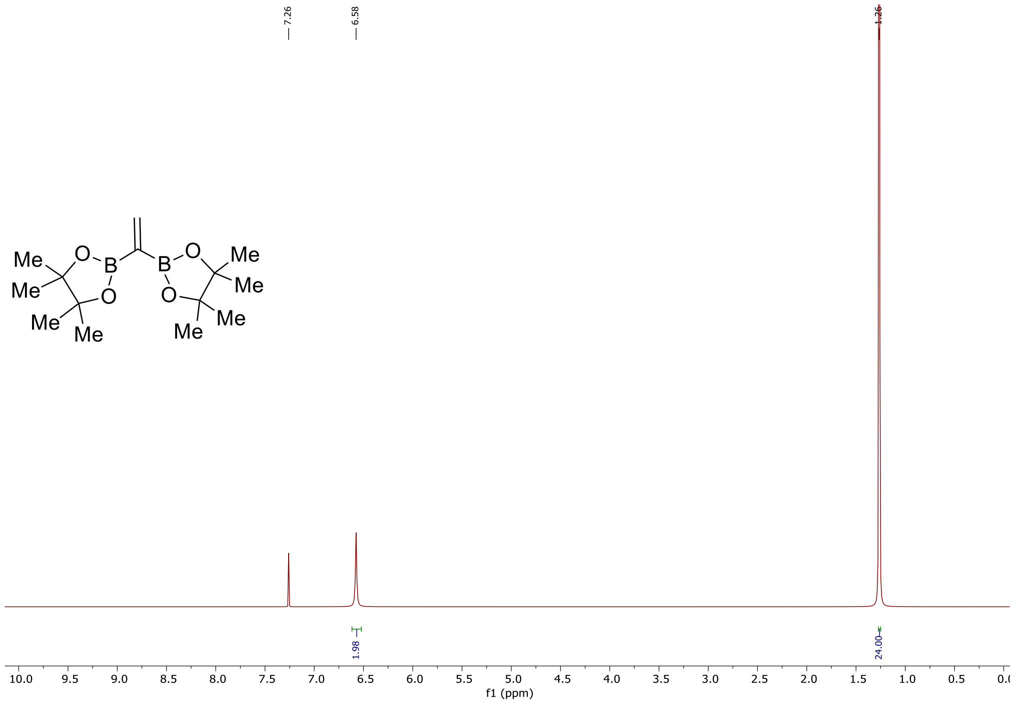

^{11}B NMR (128 MHz, $CDCl_3$) of compound (**2**)

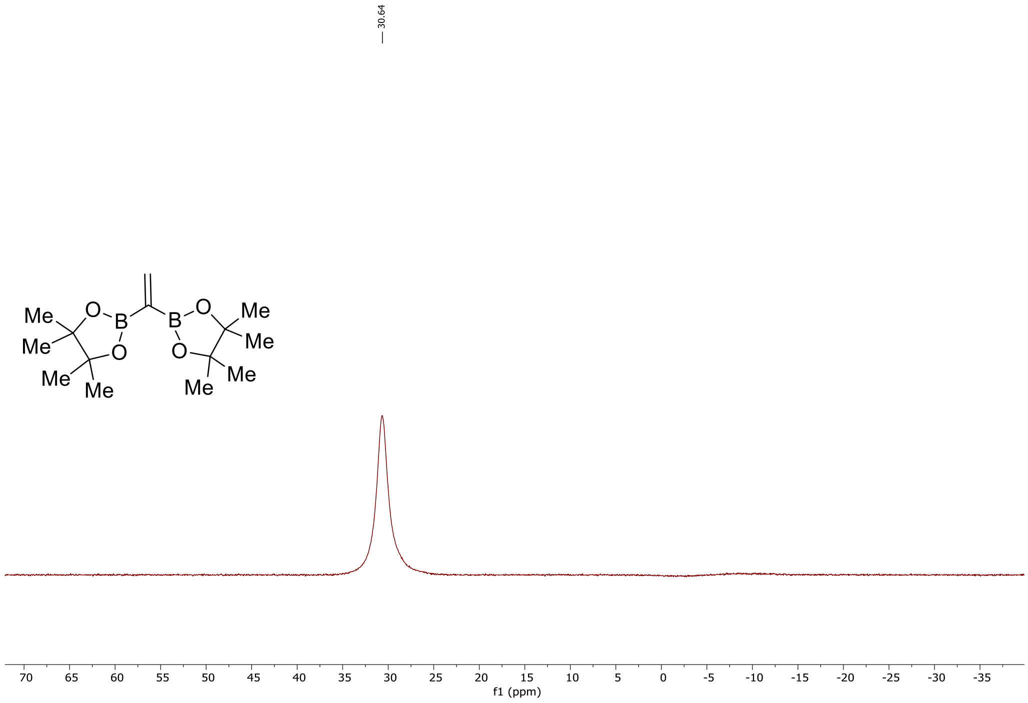

^{1}H NMR (400 MHz, $CDCl_3$) of compound (**3l**)

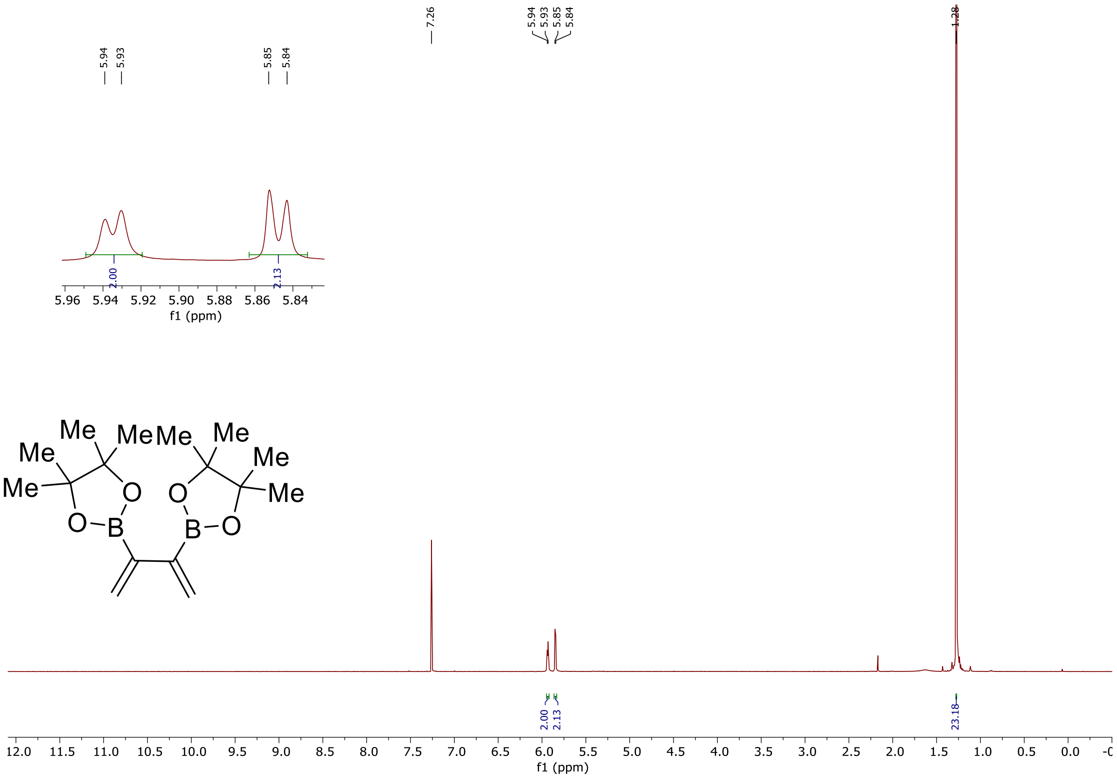

^{13}C NMR (101 MHz, $CDCl_3$) of compound (**3l**)

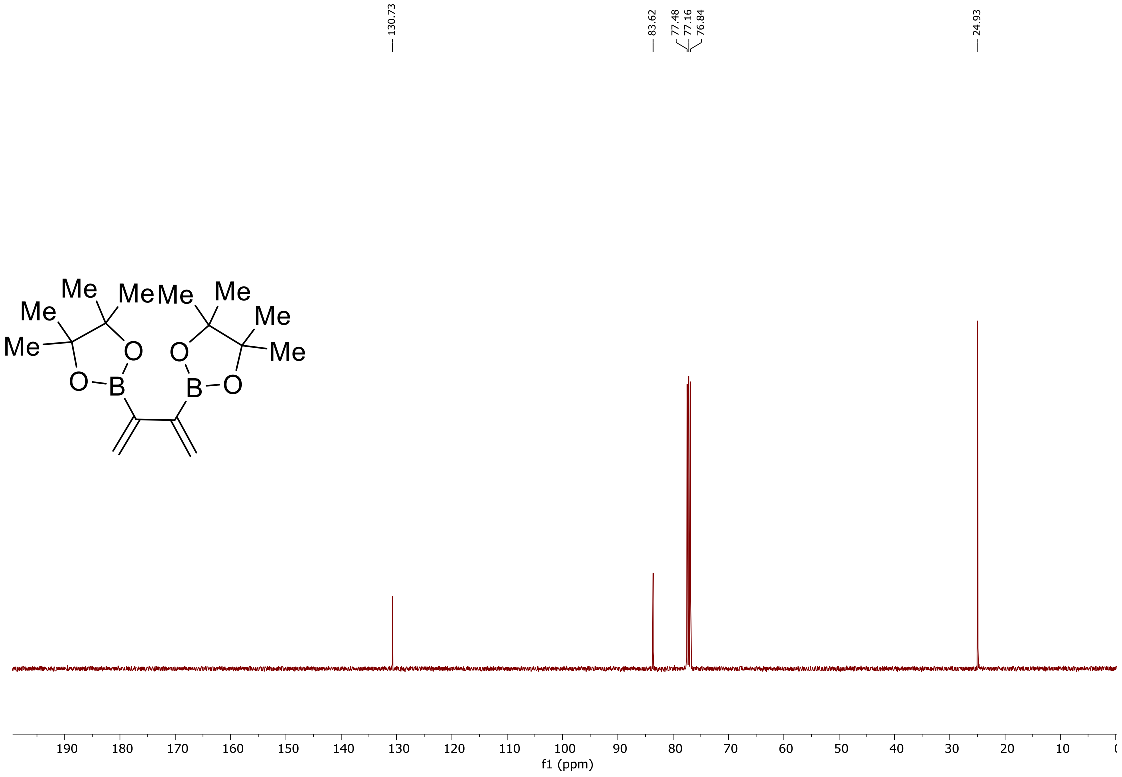

^{11}B NMR (128 MHz, $CDCl_3$) of compound (**3l**)

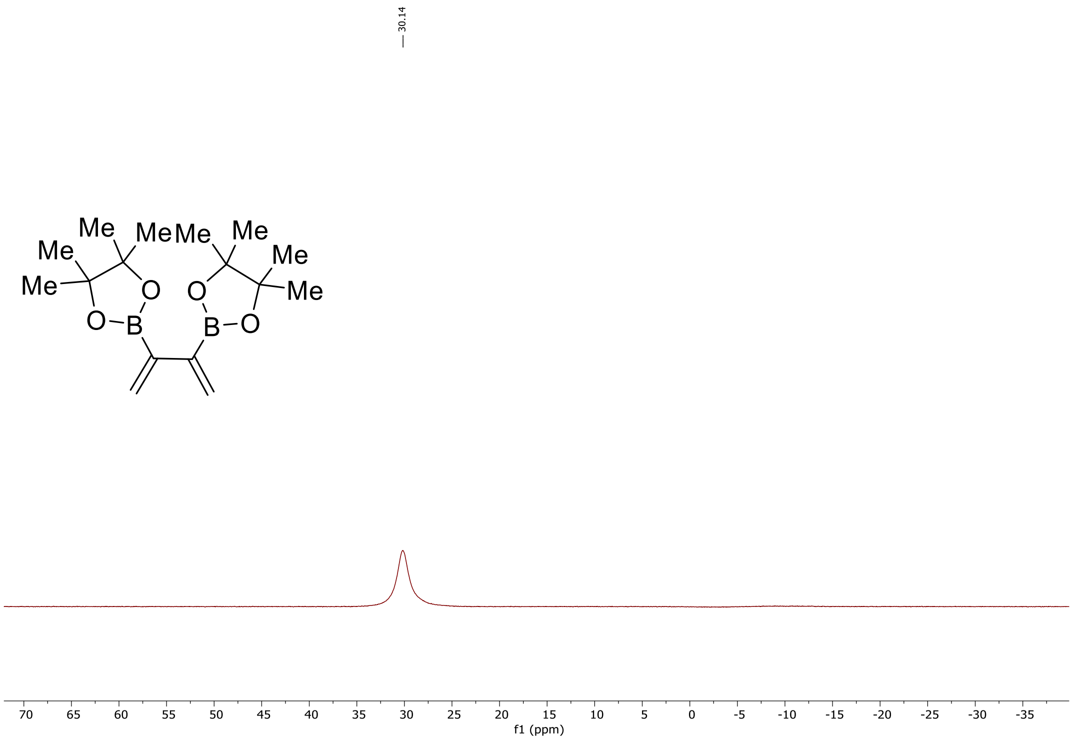

^{1}H NMR (400 MHz, $CDCl_3$) of compound (***E*-3k**)

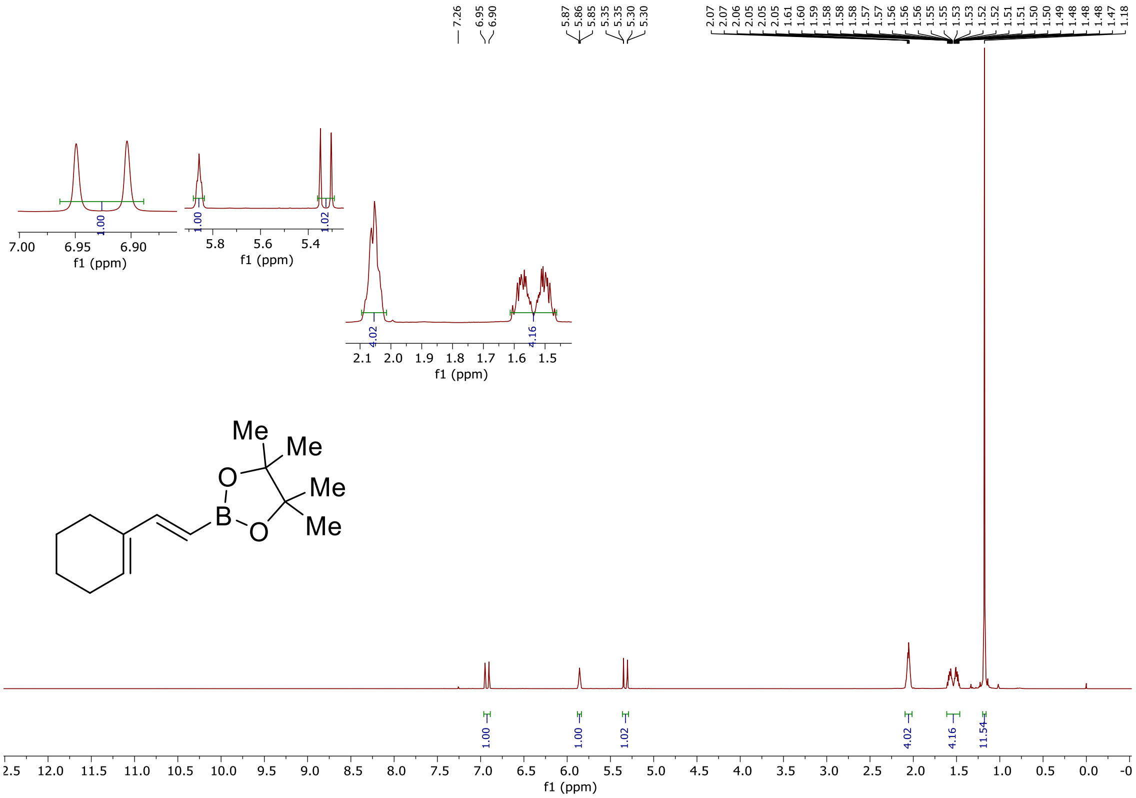

^{13}C NMR (101 MHz, $CDCl_3$) of compound (***E*-3k**)

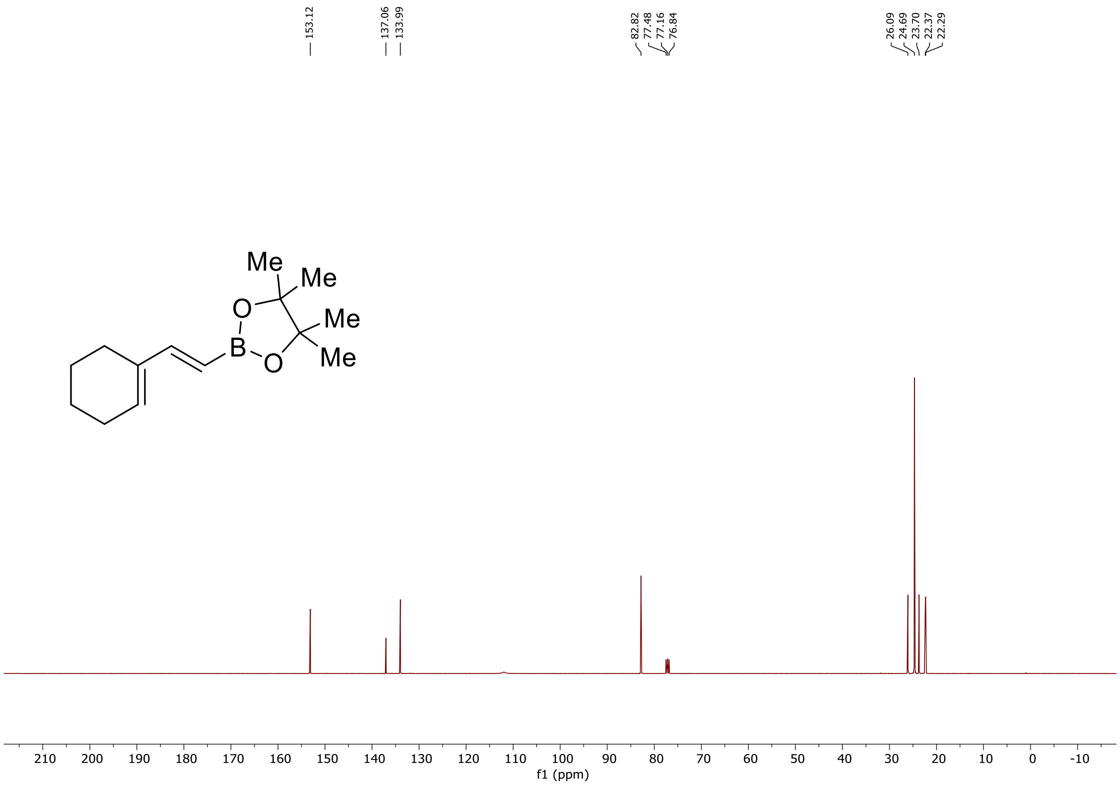

^{11}B NMR (128 MHz, $CDCl_3$) of compound (***E*-3k**)

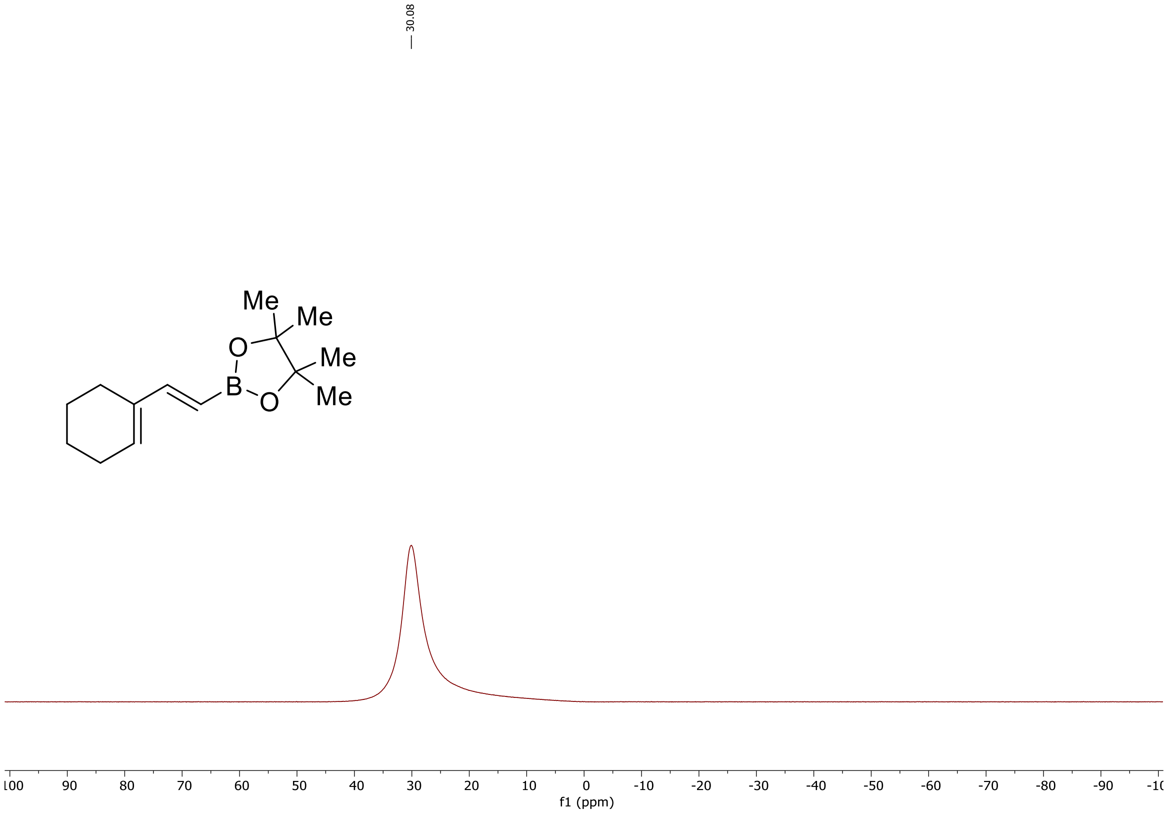

^{1}H NMR (400 MHz, $CDCl_3$) of compound (**4a**)

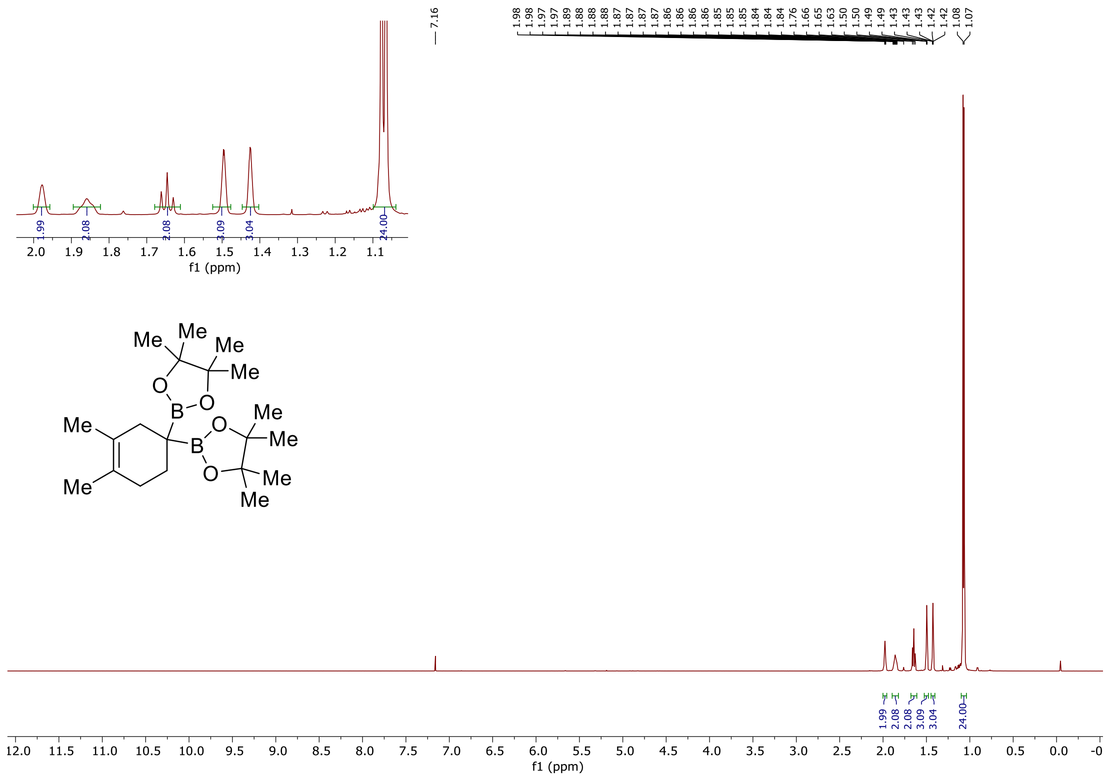

^{13}C NMR (126 MHz, $CDCl_3$) of compound (**4a**)

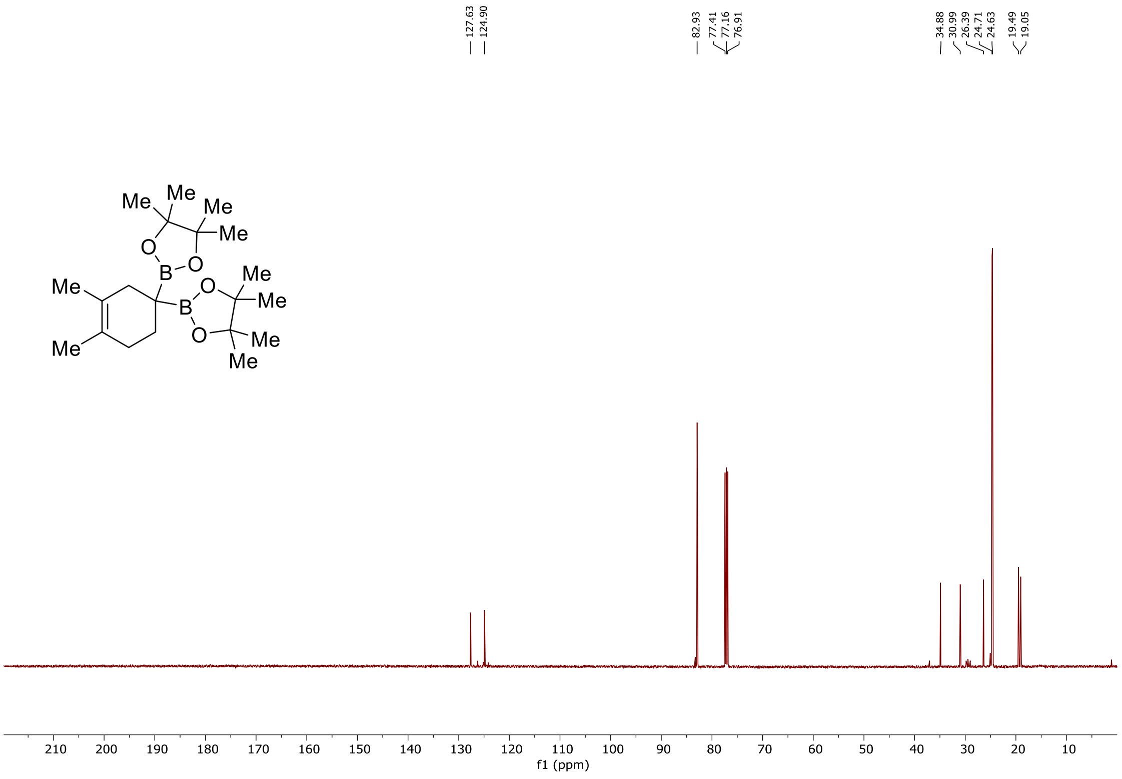

^{11}B NMR (128 MHz, $CDCl_3$) of compound (**4a**)

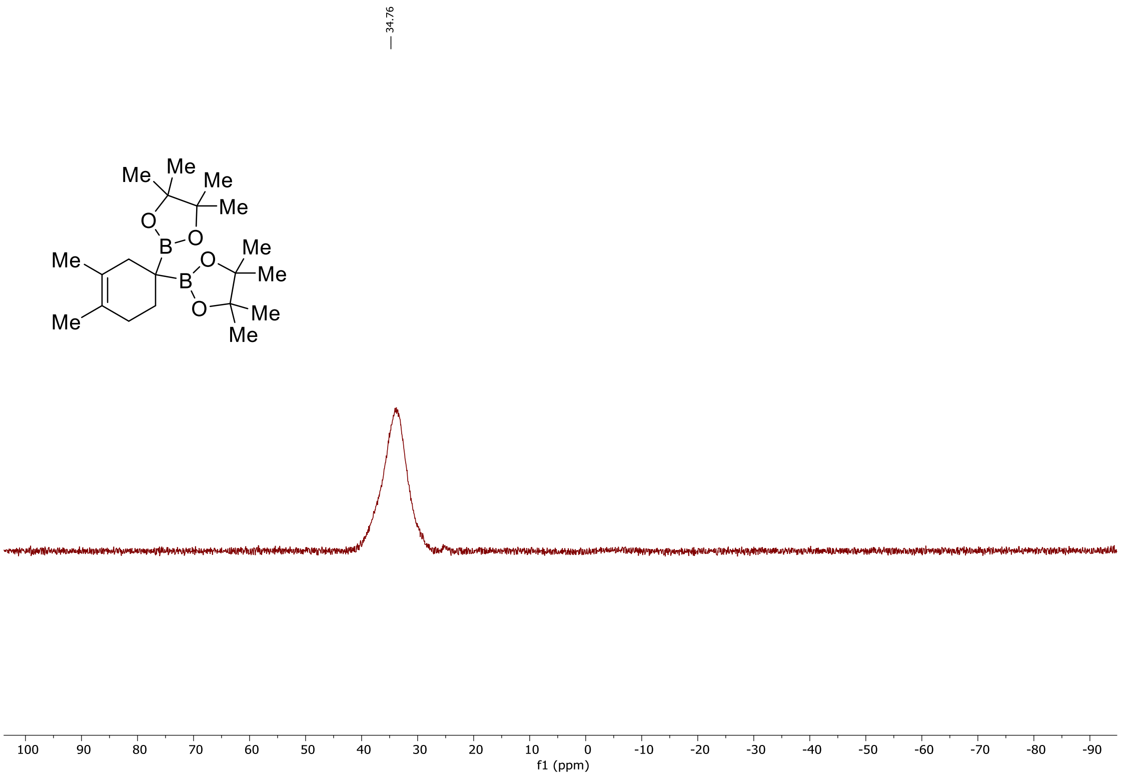

^{1}H NMR (400 MHz, CD_3CN) of compound (**4b**)

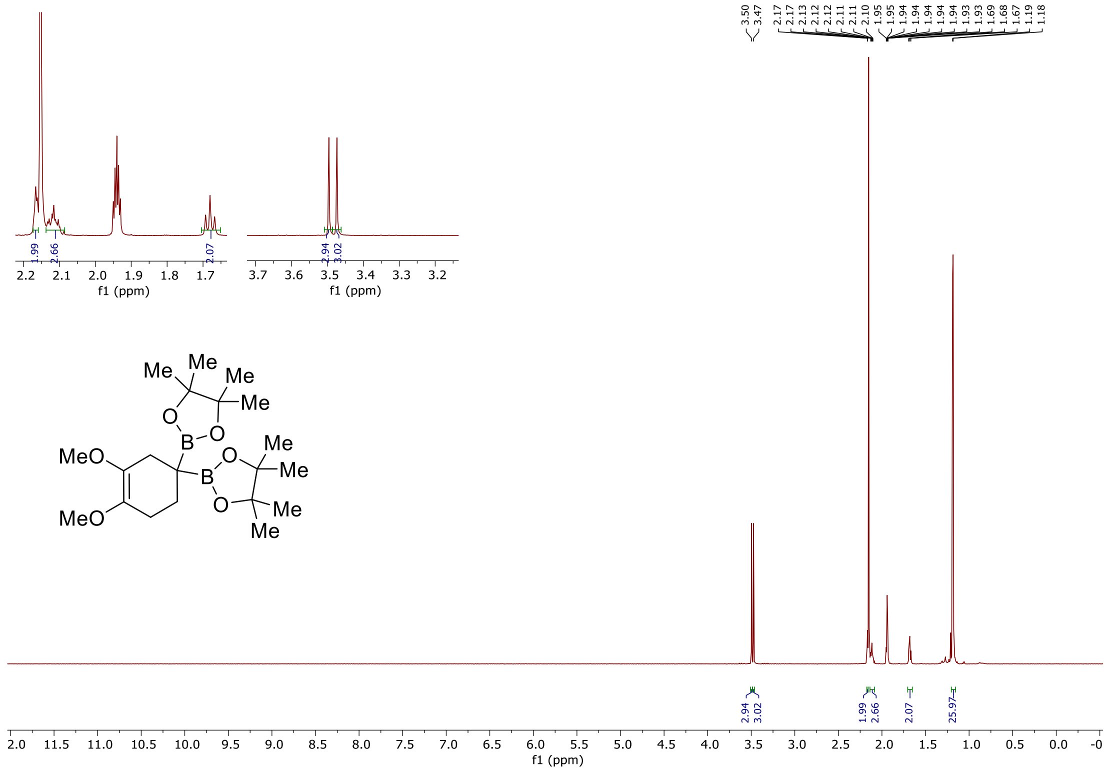

^{13}C NMR (126MHz, CD_3CN) of compound (**4b**)

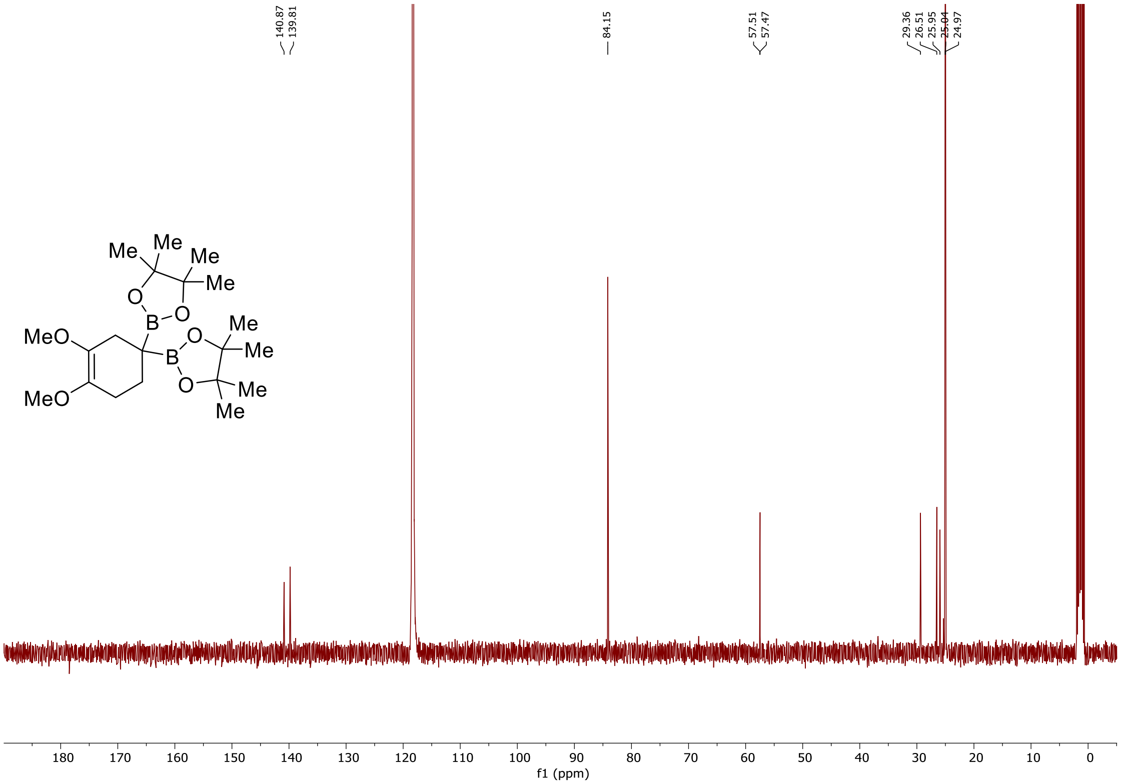

^{11}B NMR (128 MHz, CD_3CN) of compound (**4b**)

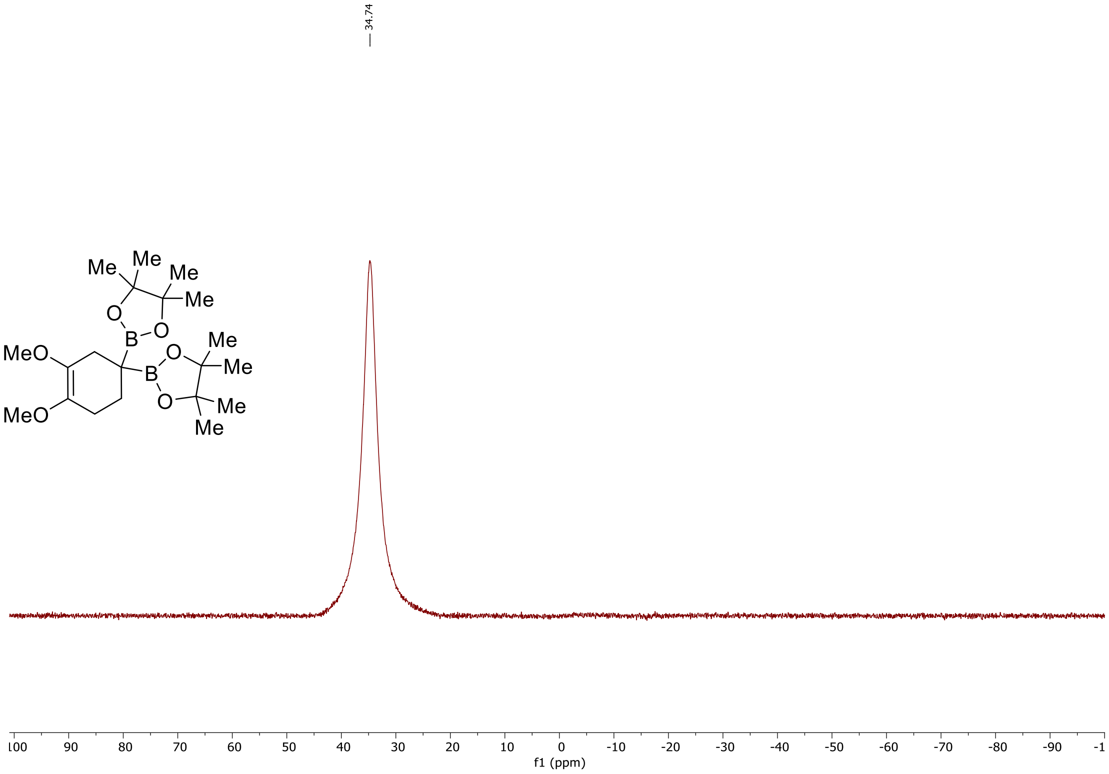

^{1}H NMR (400 MHz, $CDCl_3$) of compound (**4c**)

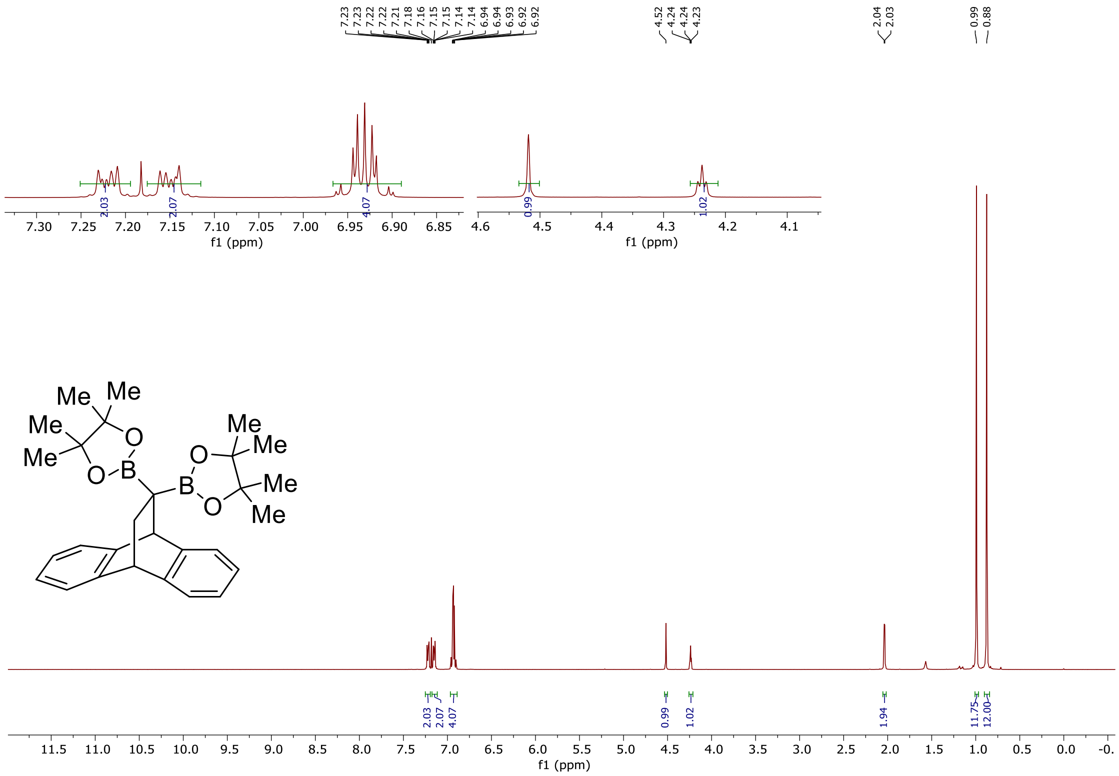

^{13}C NMR (101 MHz, $CDCl_3$) of compound (**4c**)

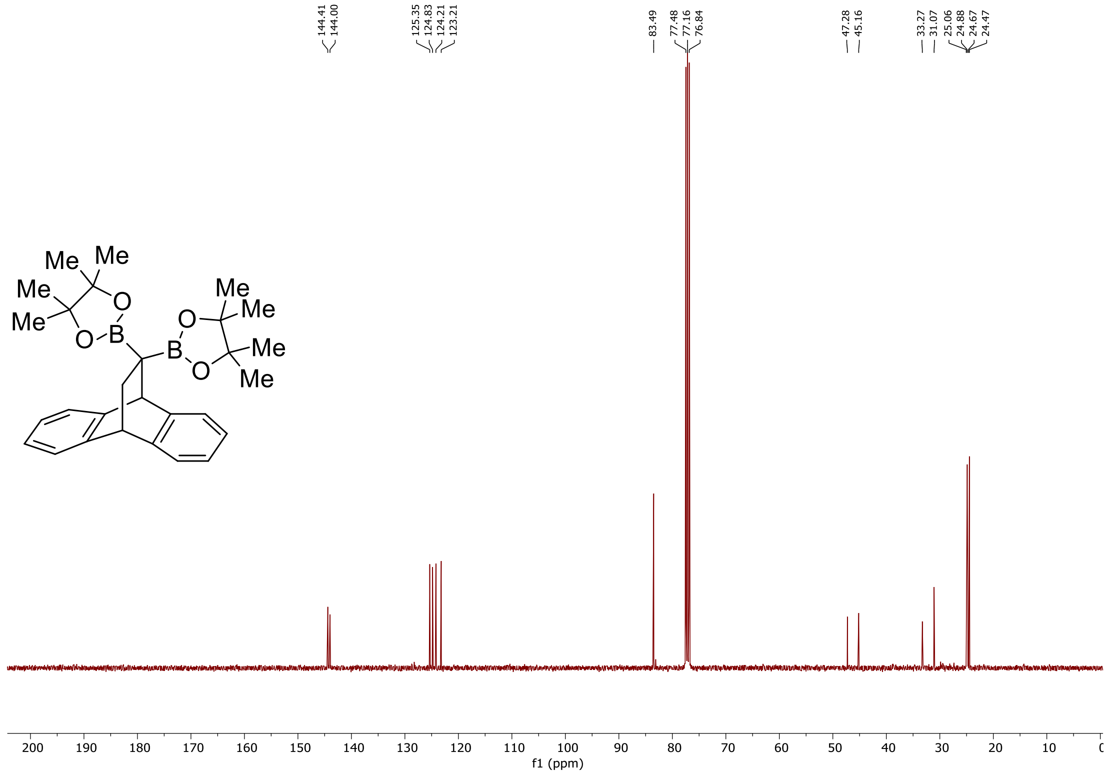

^{11}B NMR (128 MHz, CDCl$_3$) of compound (**4c**)

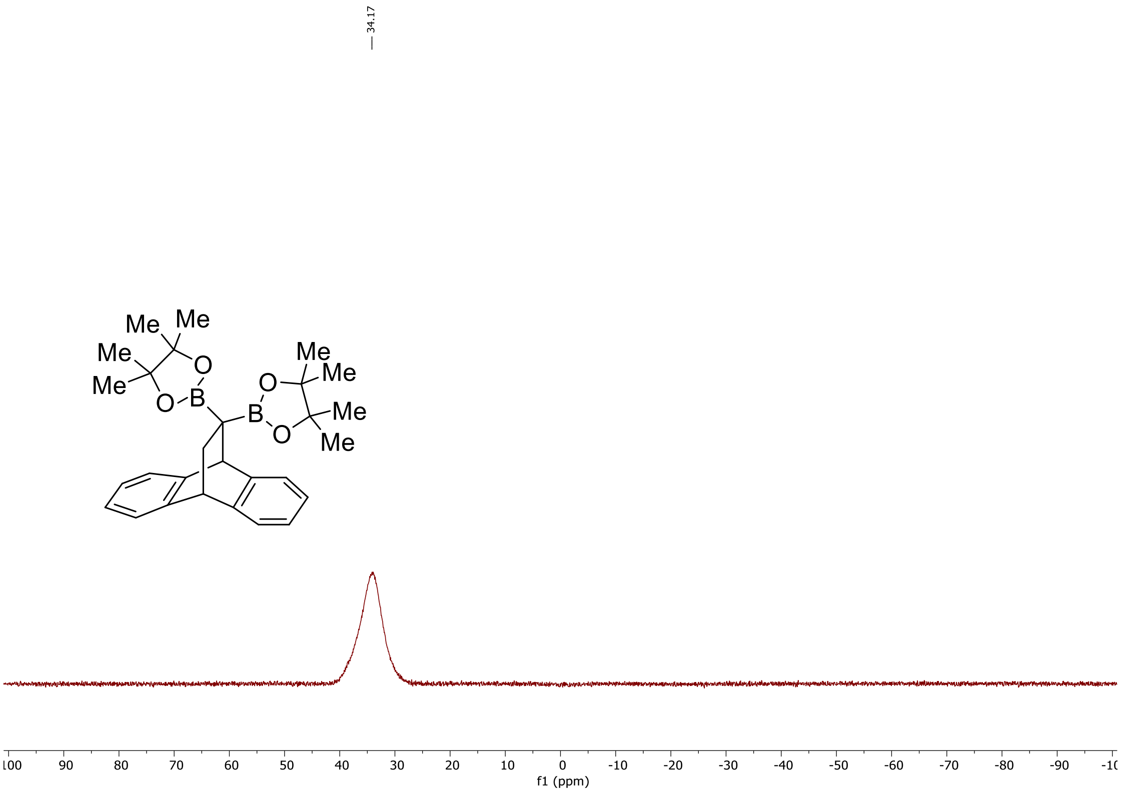

^{1}H NMR (400 MHz, CDCl$_3$) of compound (**4d**)

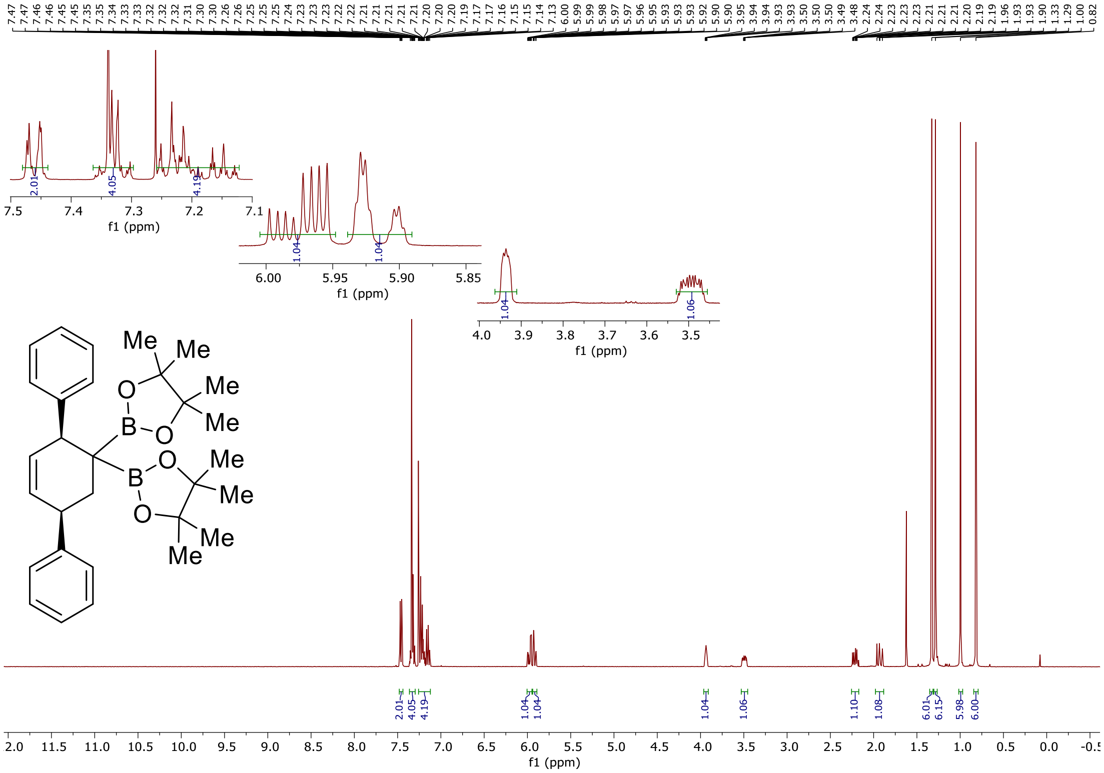

^{13}C NMR (101 MHz, $CDCl_3$) of compound (**4d**)

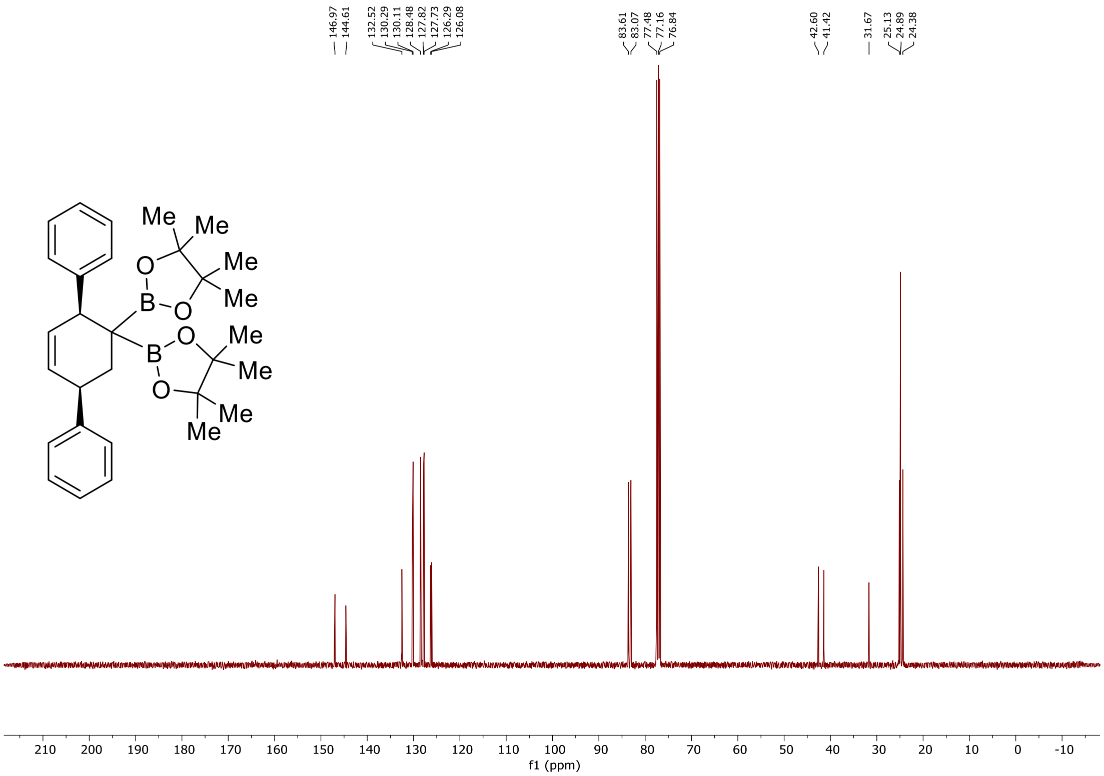

^{11}B NMR (128 MHz, $CDCl_3$) of compound (**4d**)

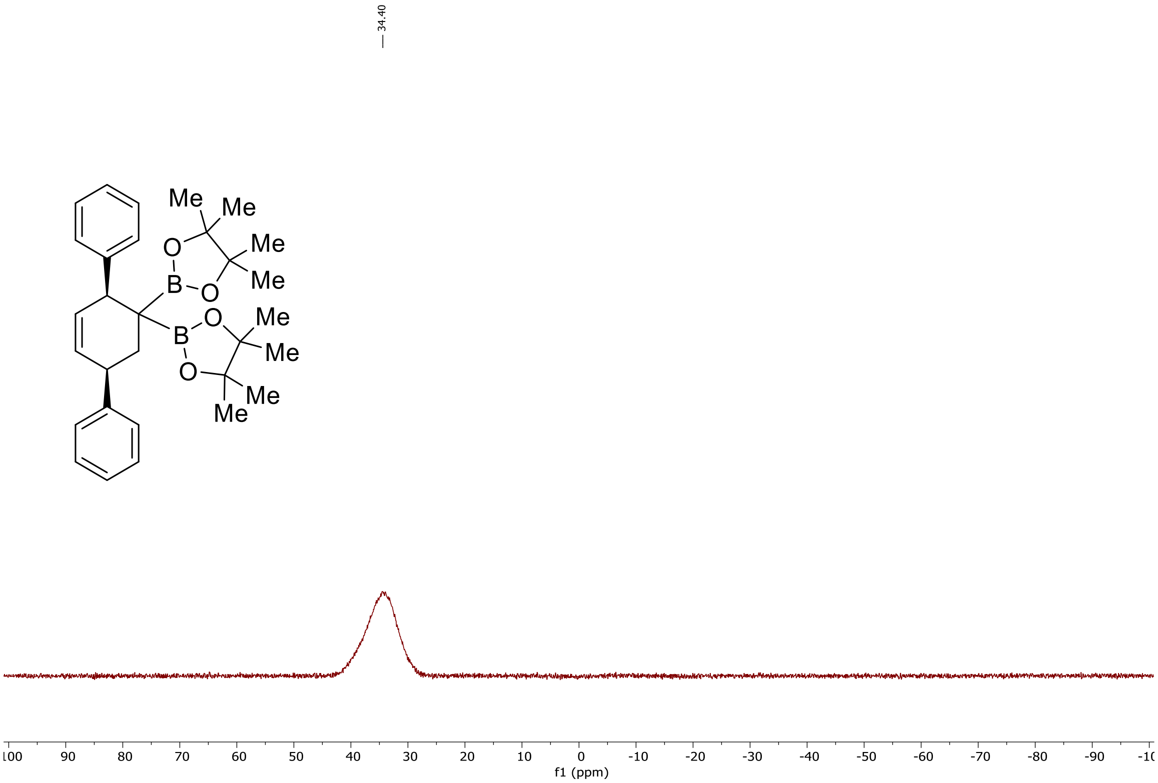

^{1}H NMR (400 MHz, $CDCl_3$) of compound (**4e**)

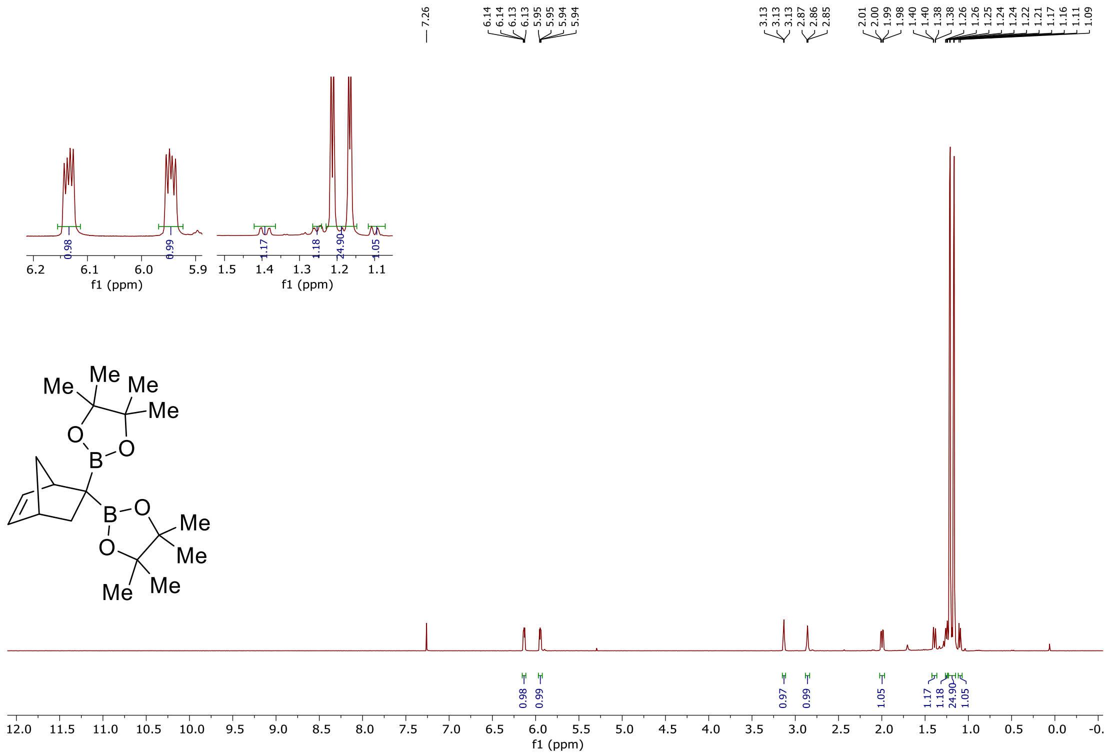

^{13}C NMR (126 MHz, $CDCl_3$) of compound (**4e**)

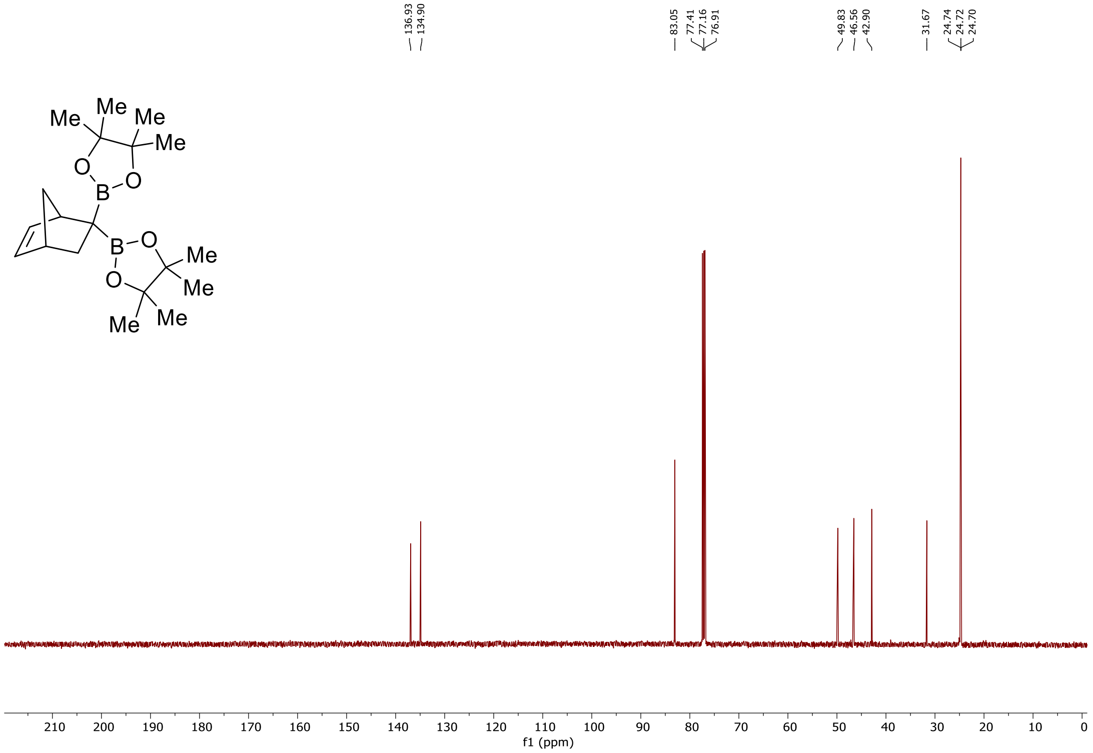

^{11}B NMR (128 MHz, $CDCl_3$) of compound (**4e**)

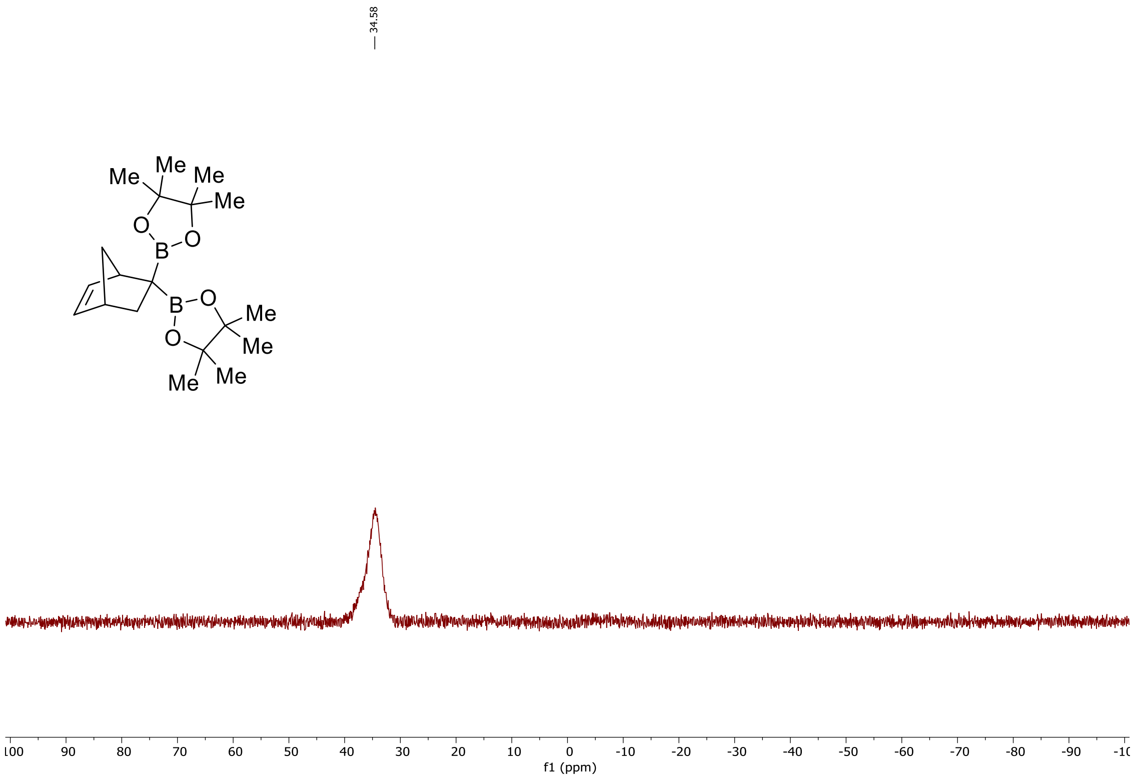

^{1}H NMR (400 MHz, $CDCl_3$) of compound (**4e-6Cl**)

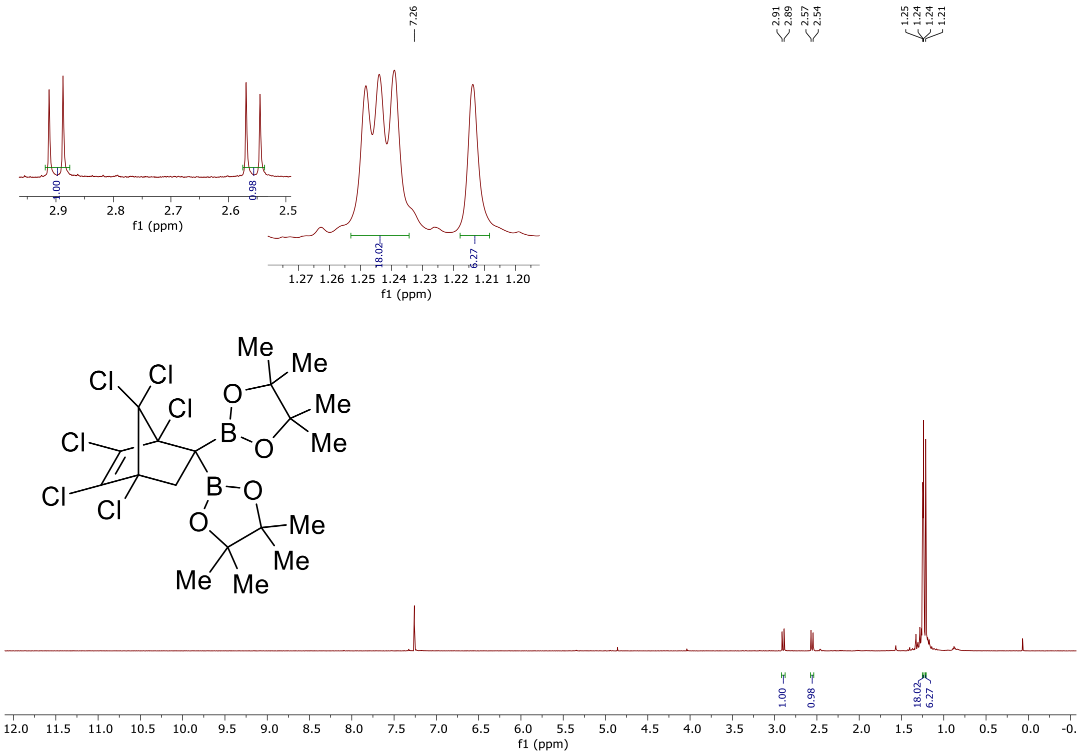

^{13}C NMR (101 MHz, $CDCl_3$) of compound (**4e-6Cl**)

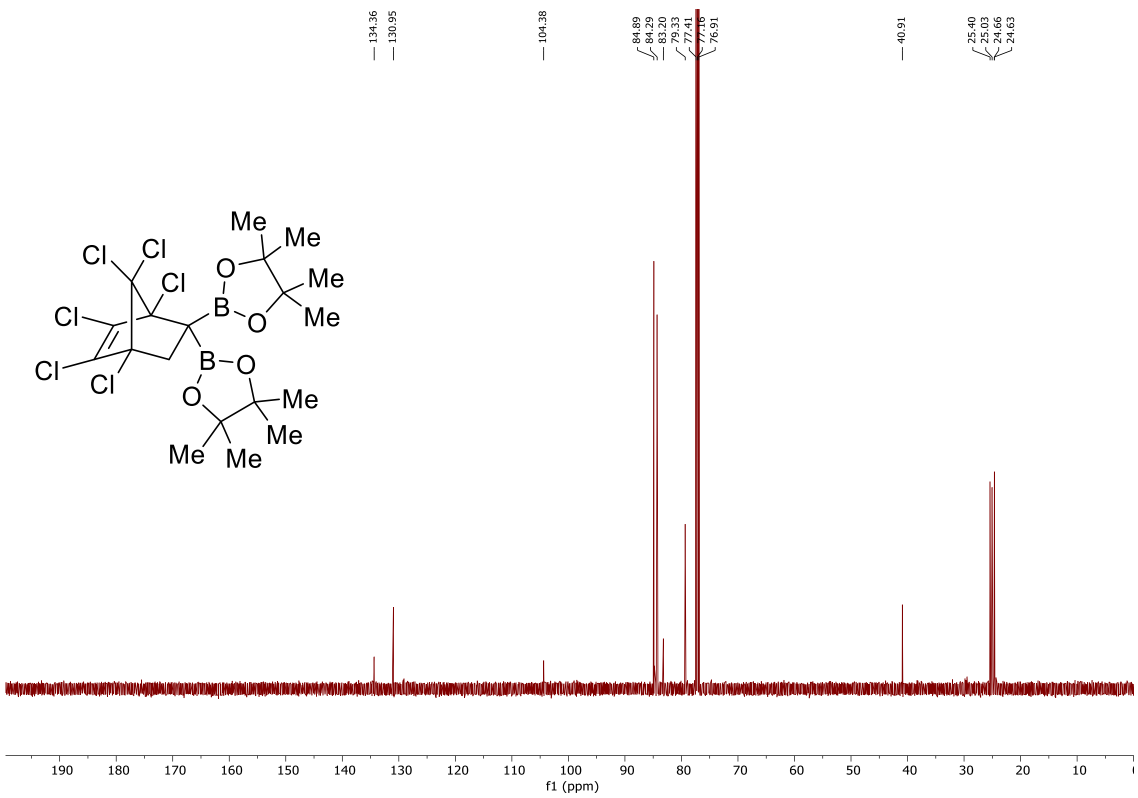

^{11}B NMR (128 MHz, $CDCl_3$) of compound (**4f**)

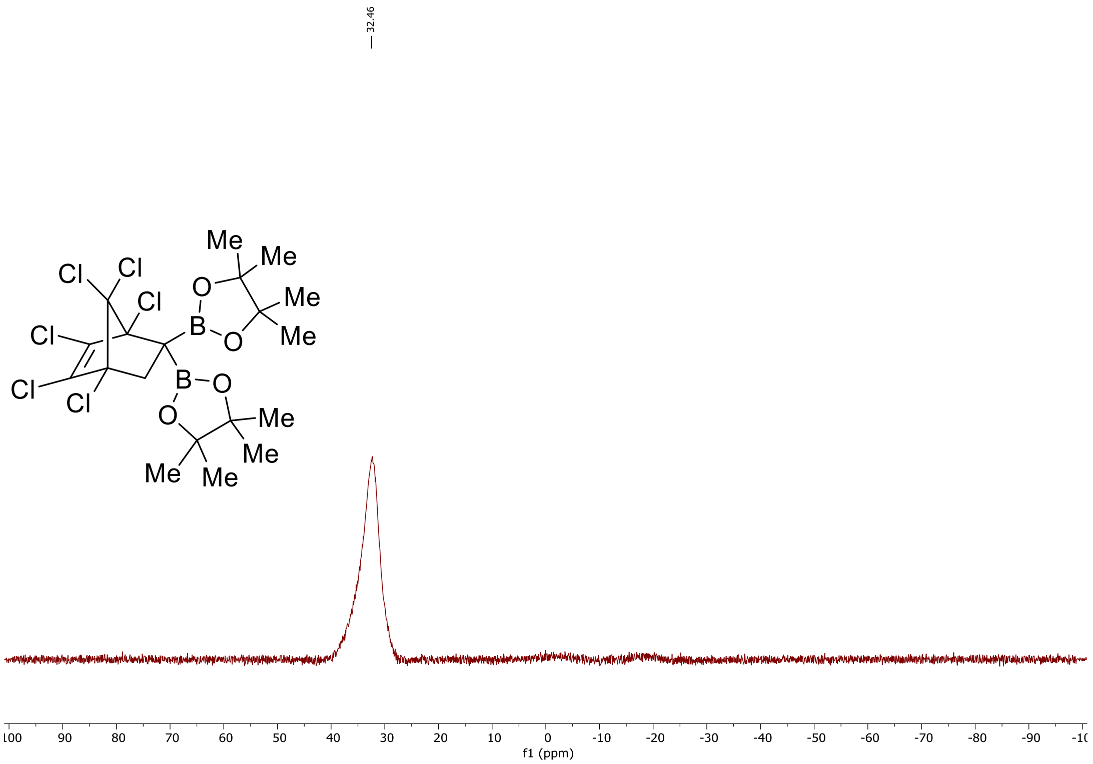

^{1}H NMR (400 MHz, $CDCl_3$) of compound (**4f**)

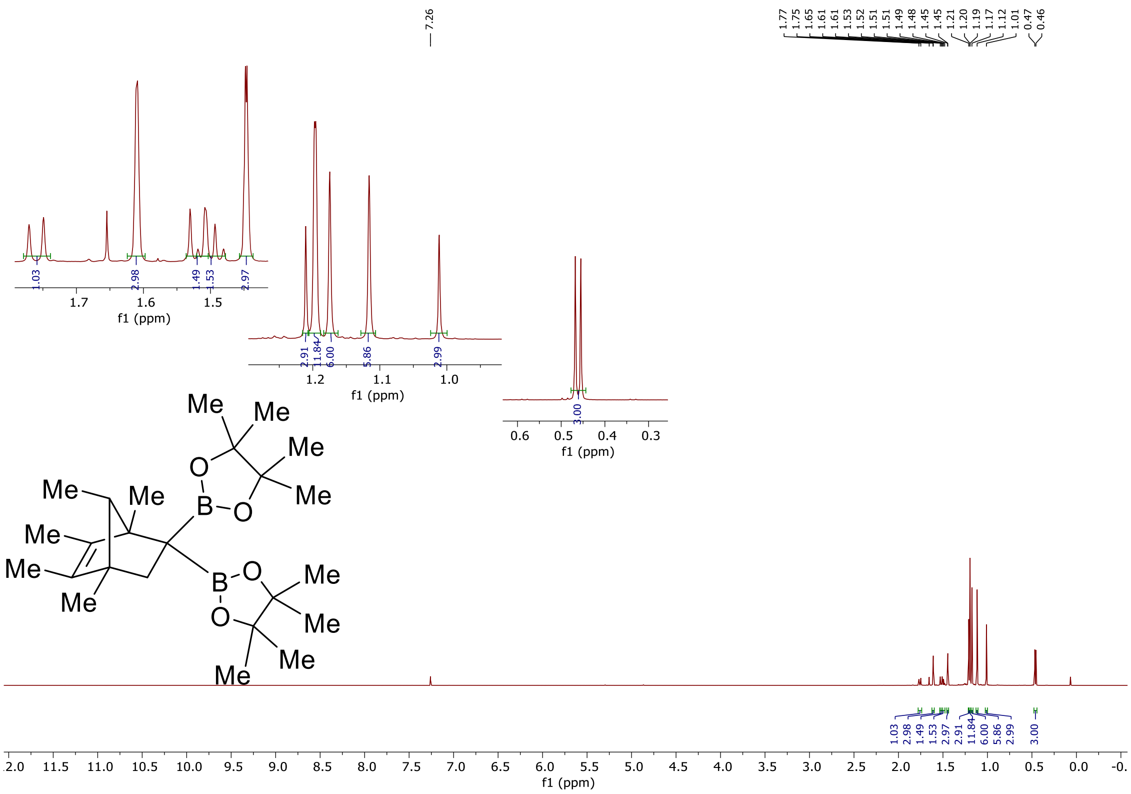

^{13}C NMR (101 MHz, $CDCl_3$) of compound (**4f**)

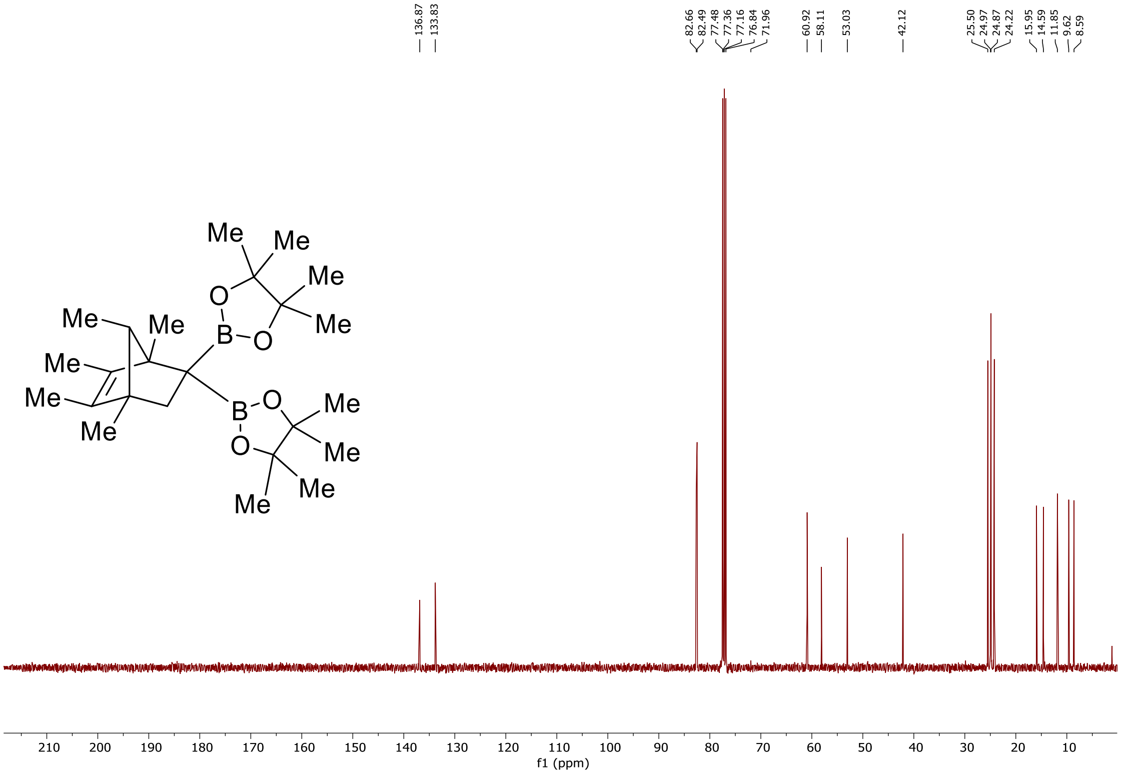

^{11}B NMR (128 MHz, $CDCl_3$) of compound (**4f**)

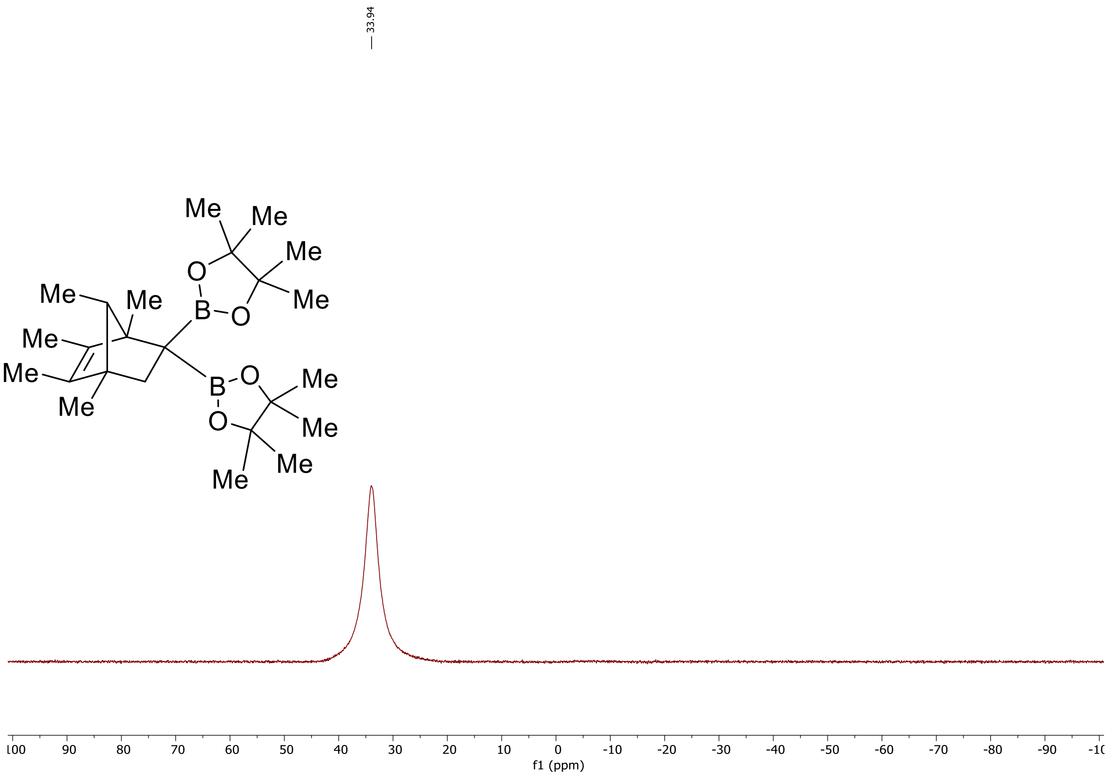

^{1}H NMR (400 MHz, $CDCl_3$) of compound (**4g**)

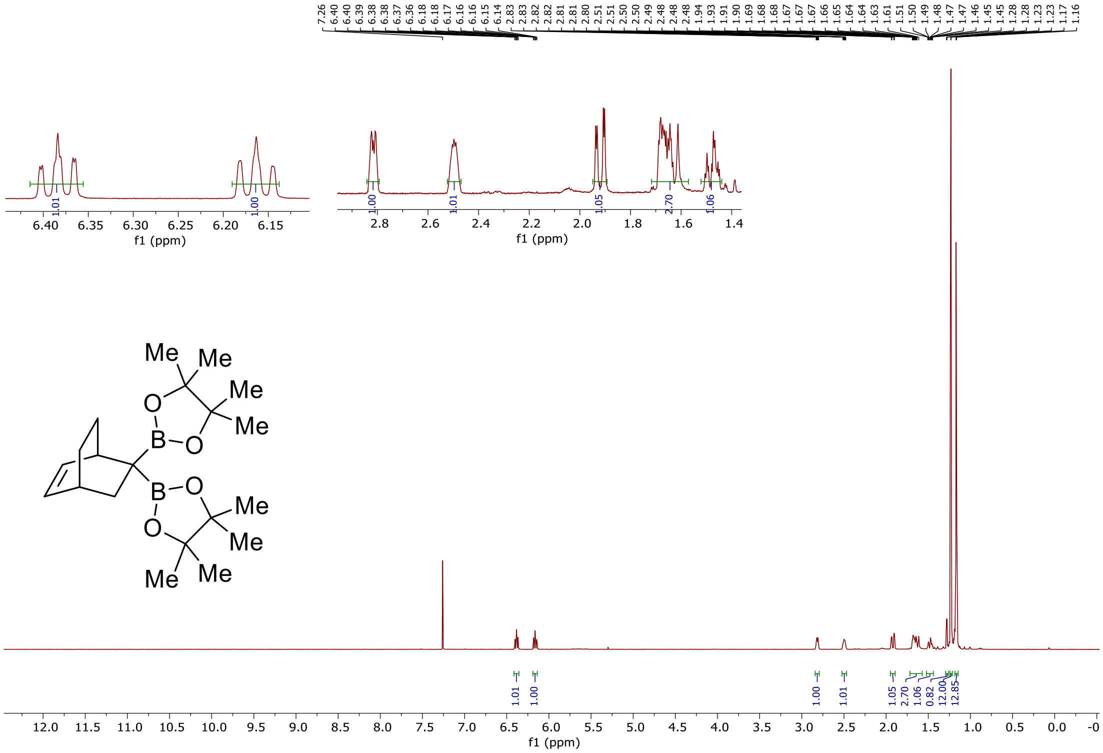

^{13}C NMR (101 MHz, $CDCl_3$) of compound (**4g**)

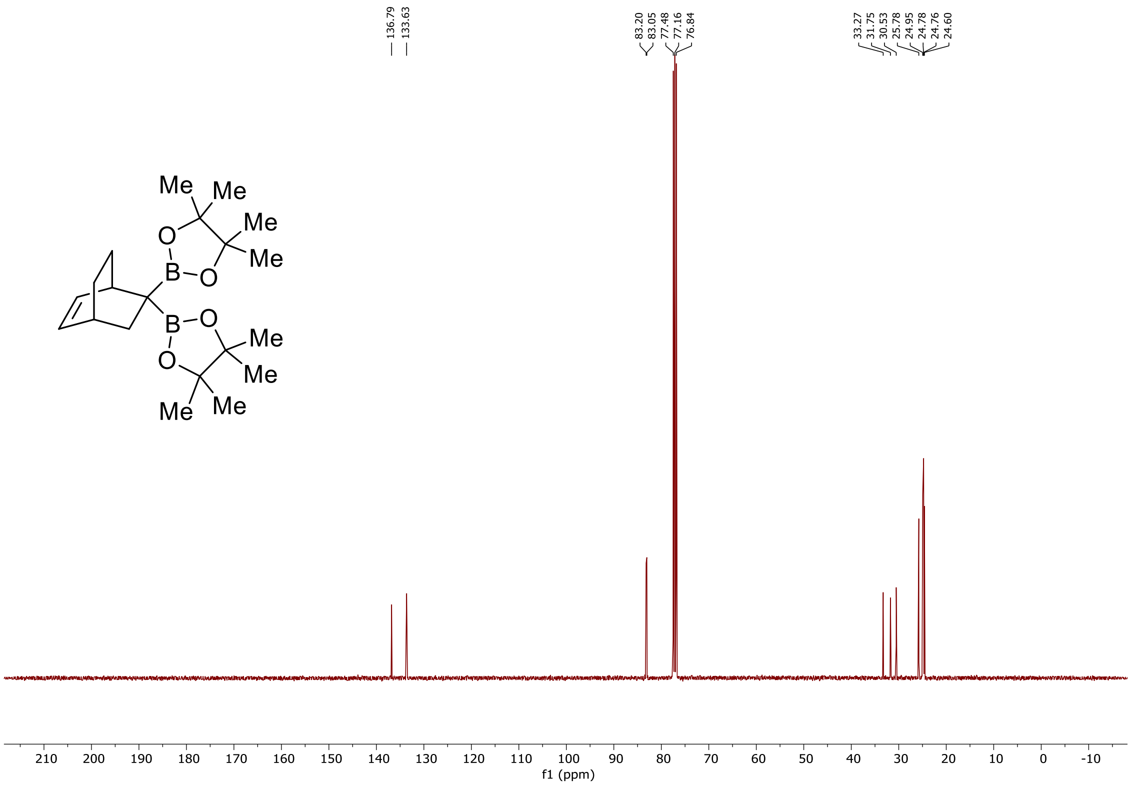

^{11}B NMR (128 MHz, $CDCl_3$) of compound (**4g**)

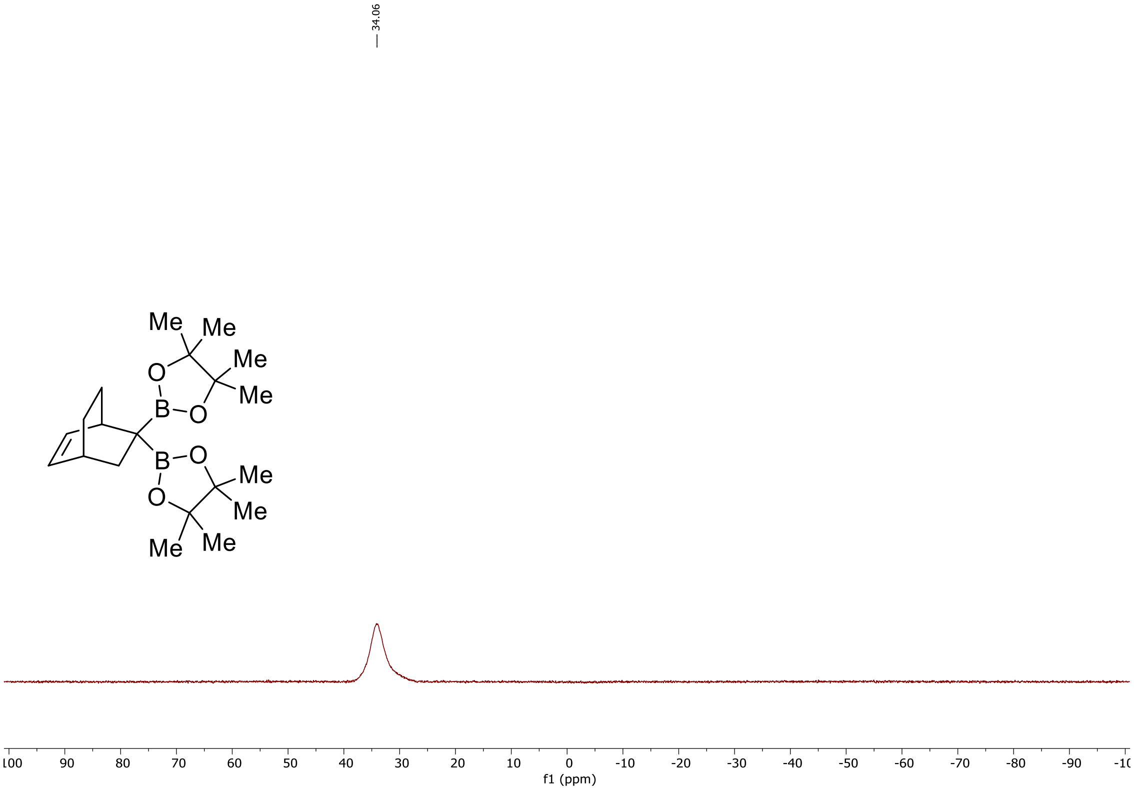

^{1}H NMR (400 MHz, $CDCl_3$) of compound (**4g-Si**)

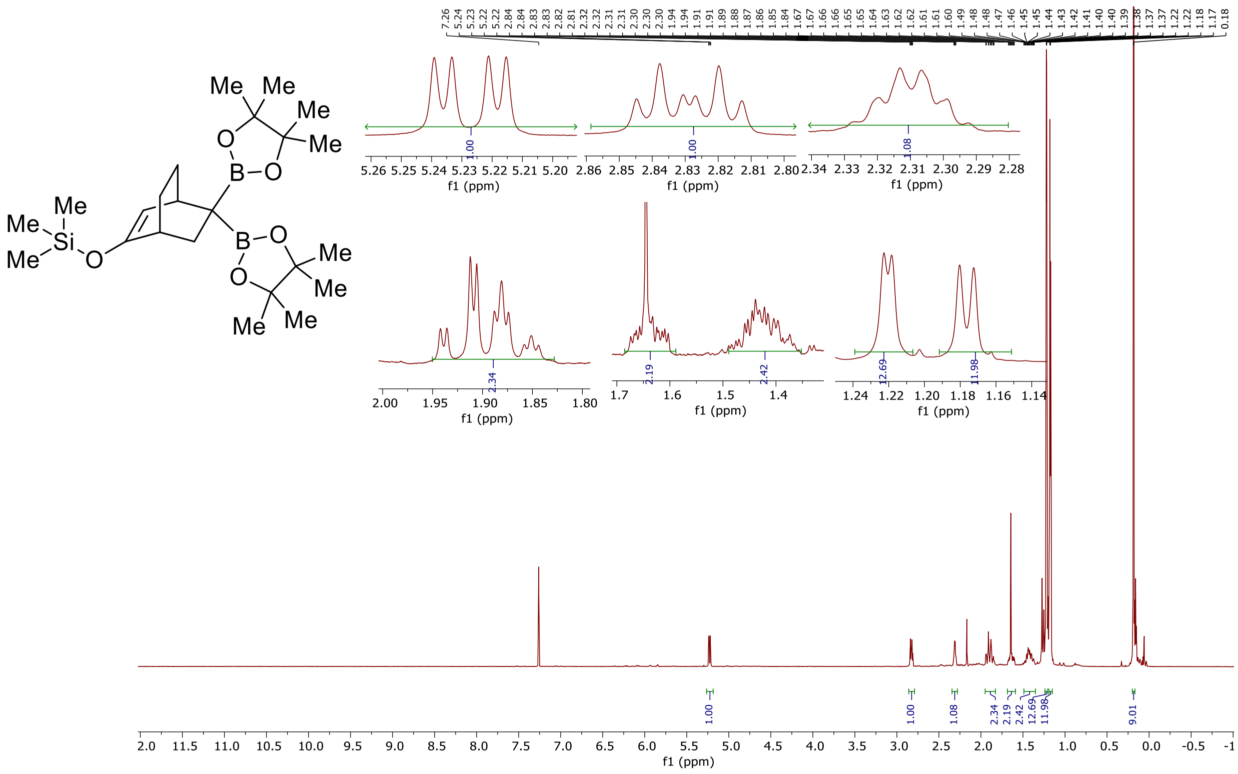

^{13}C NMR (101 MHz, $CDCl_3$) of compound (**4g-Si**)

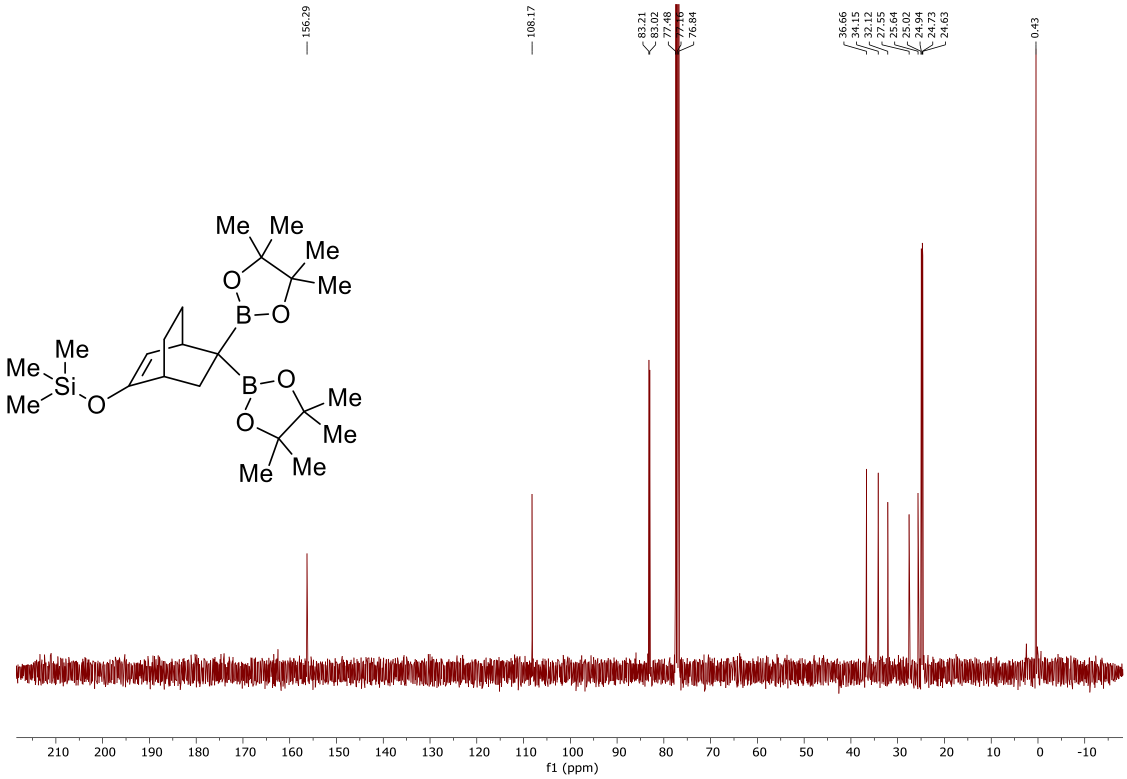

^{11}B NMR (128 MHz, $CDCl_3$) of compound (**4g-Si**)

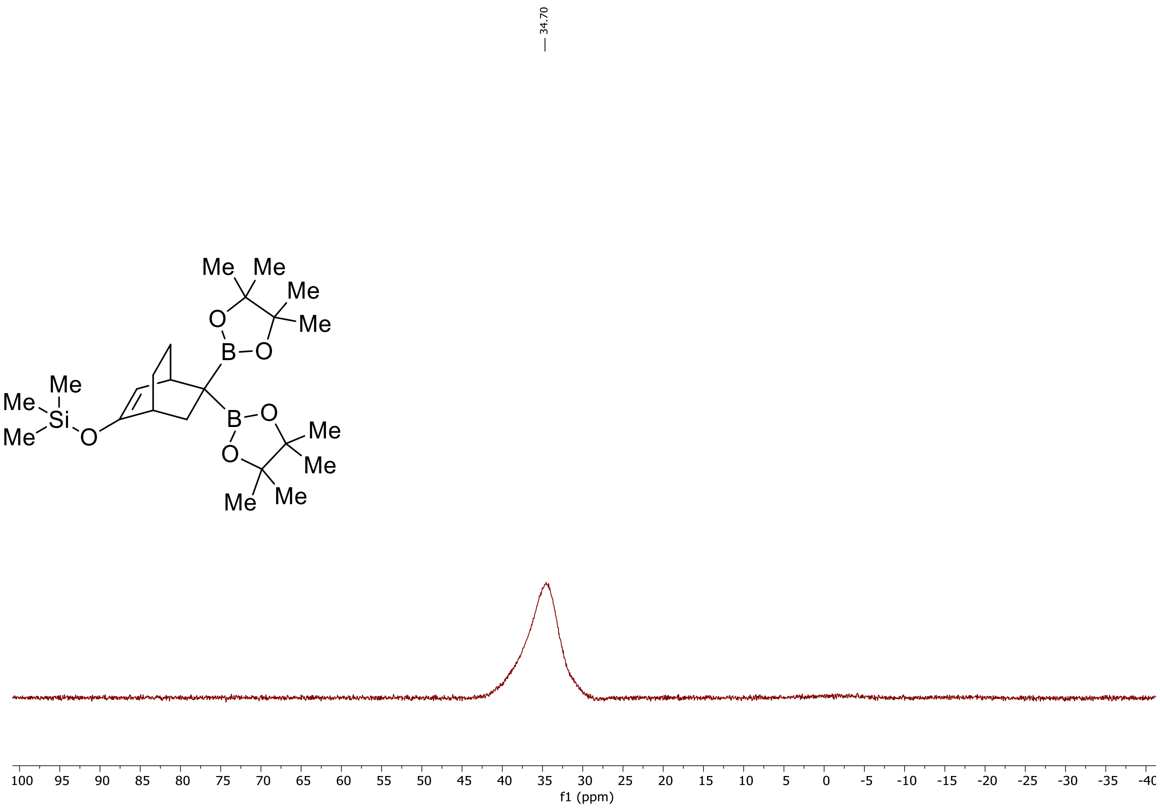

^{1}H NMR (400 MHz, $CDCl_3$) of compound (**4h**)

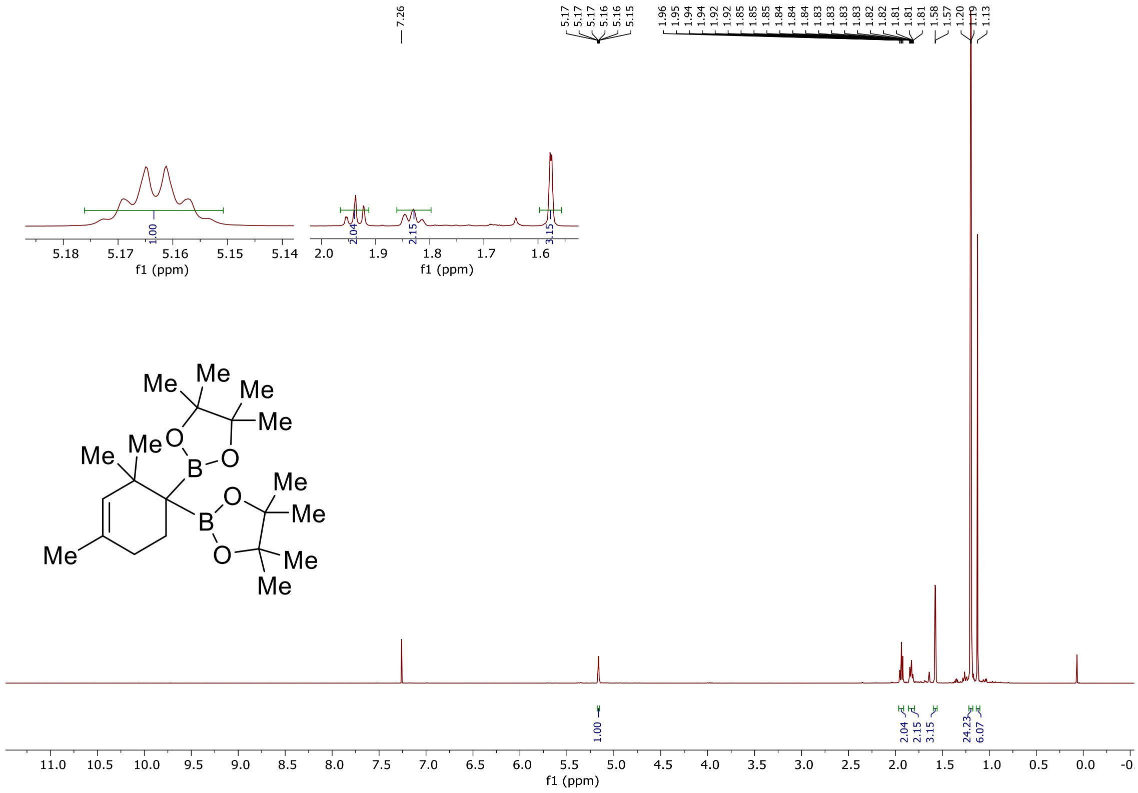

^{13}C NMR (101 MHz, $CDCl_3$) of compound (**4h**)

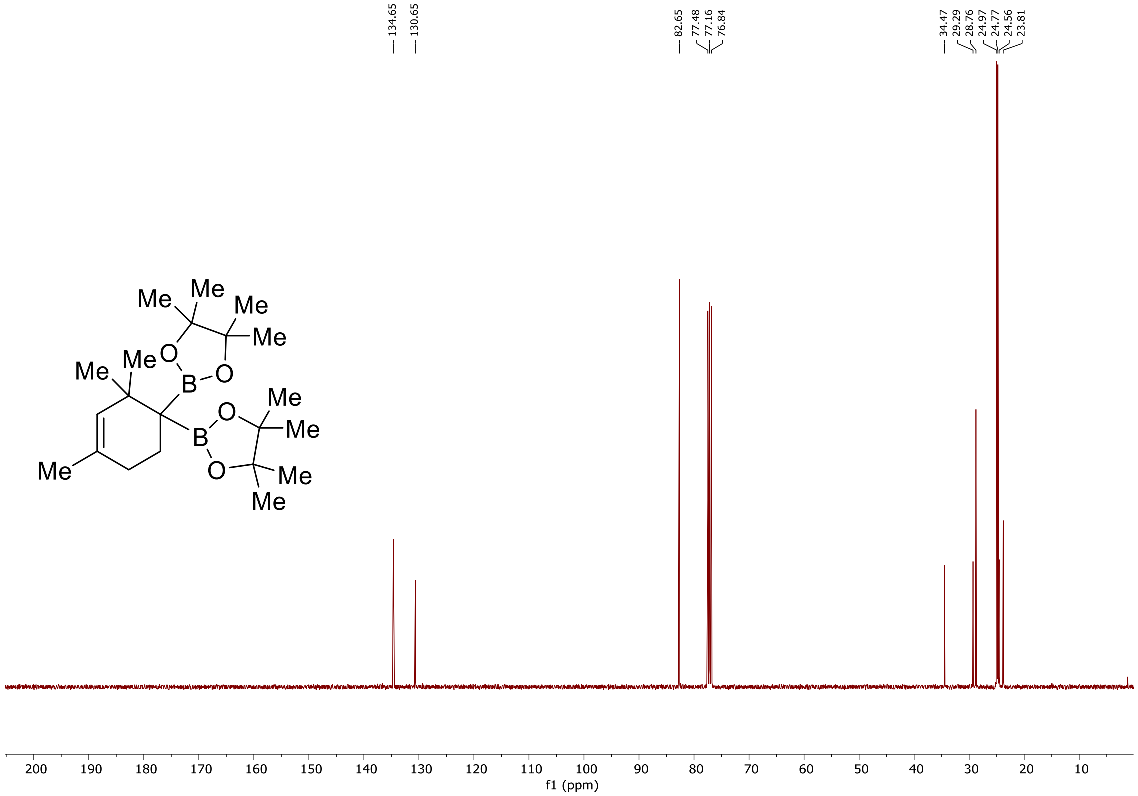

^{11}B NMR (128 MHz, $CDCl_3$) of compound (**4h**)

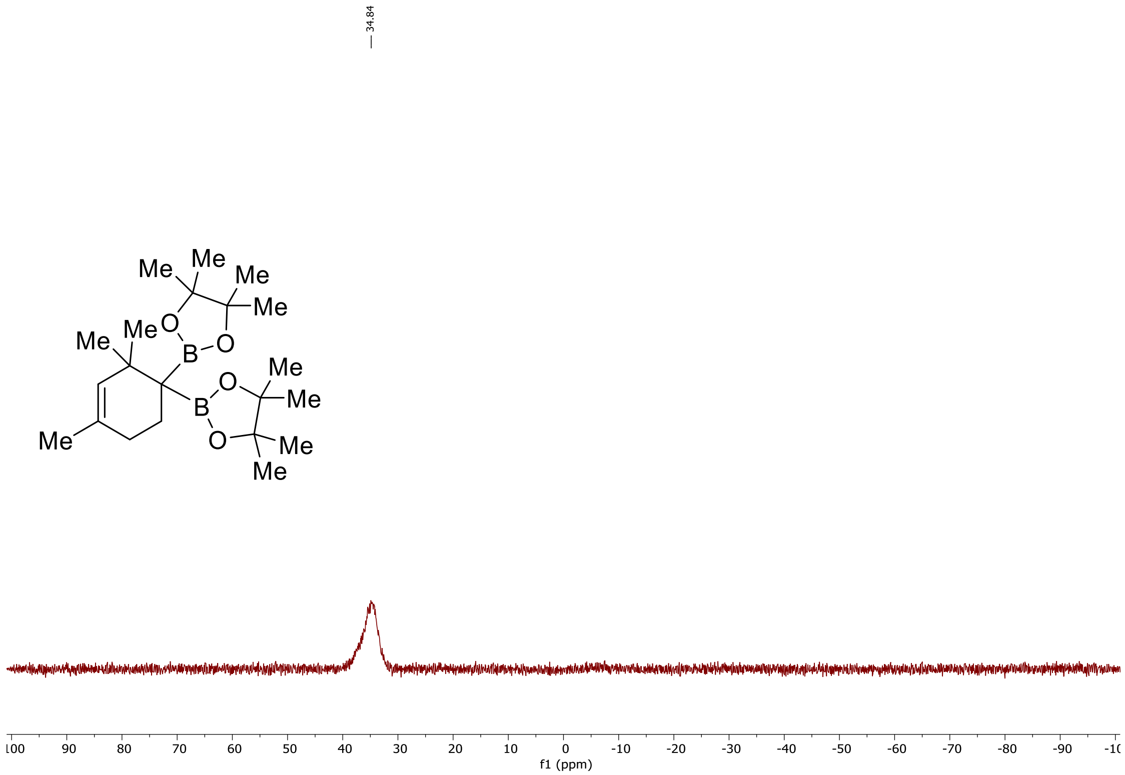

^{1}H NMR (400 MHz, $CDCl_3$) of compound (**4h'**)

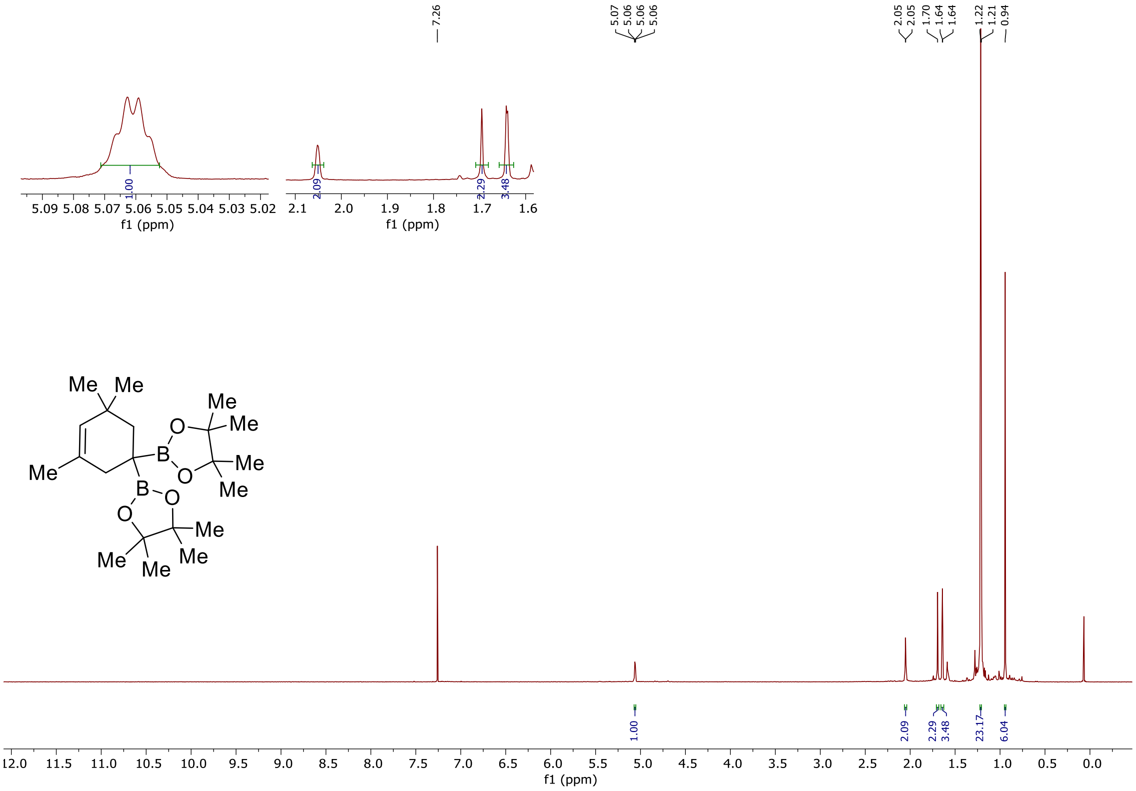

^{13}C NMR (101 MHz, $CDCl_3$) of compound (**4h'**)

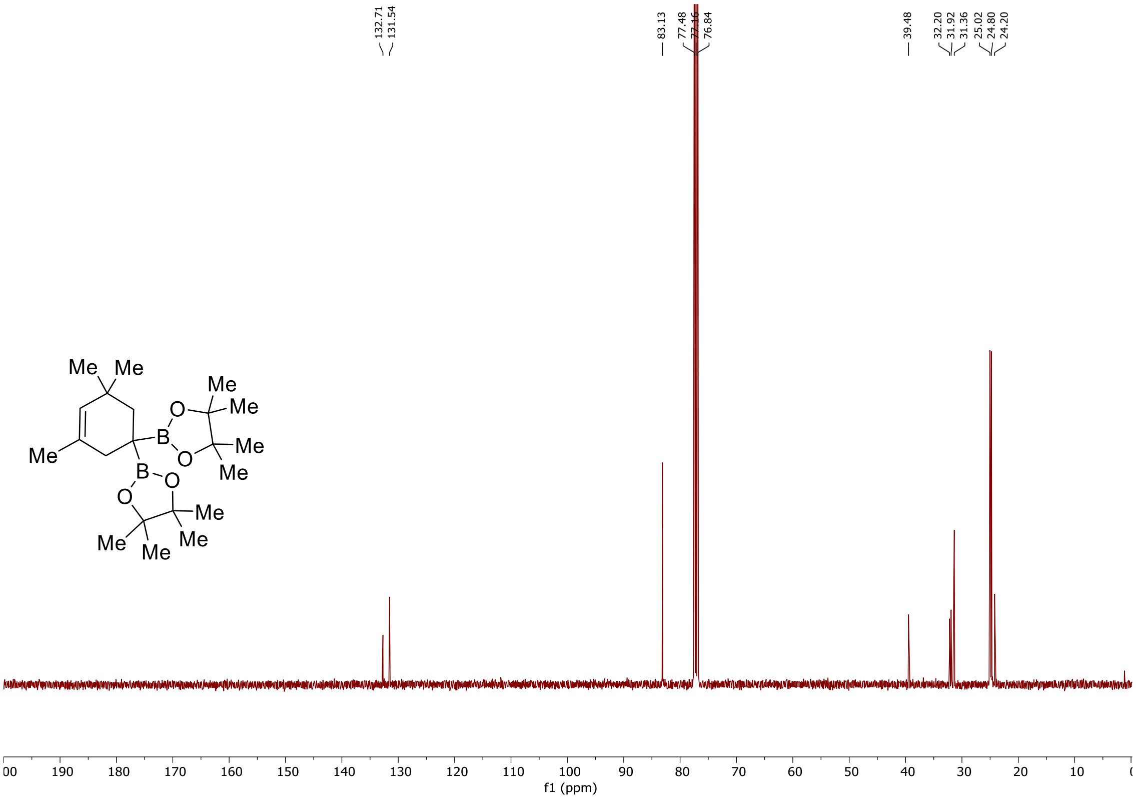

^{11}B NMR (128 MHz, $CDCl_3$) of compound (**4h'**)

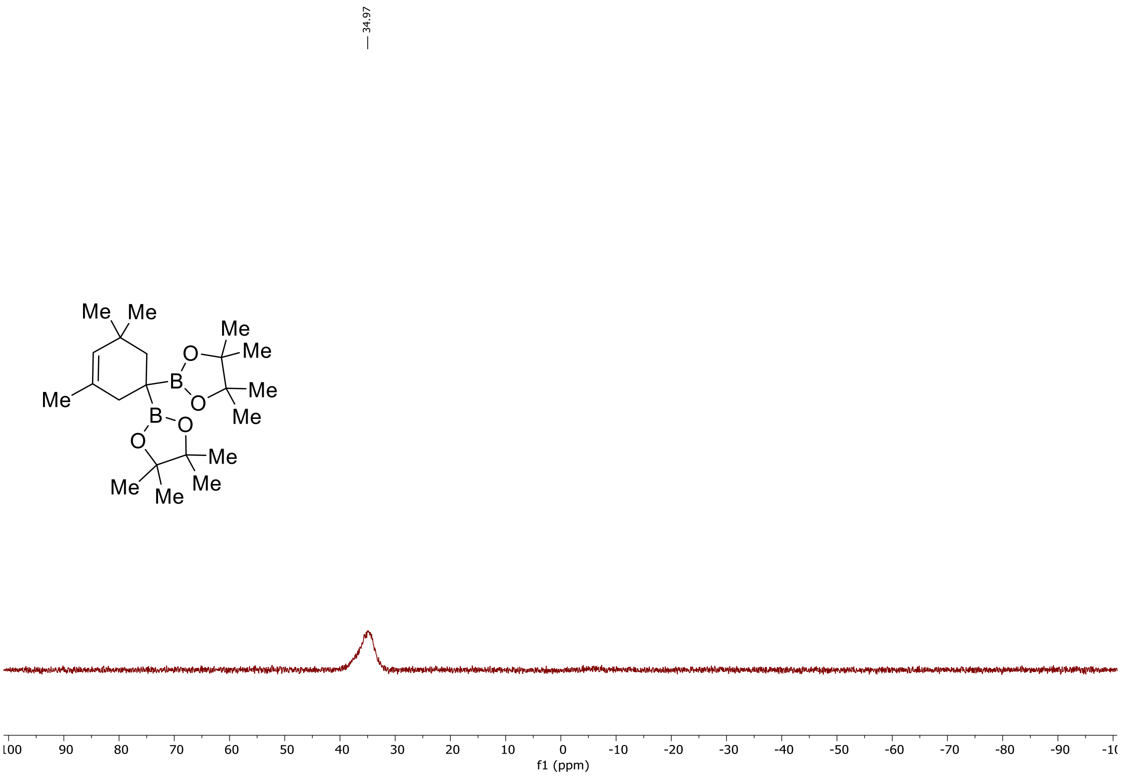

^{1}H NMR (400 MHz, $CDCl_3$) of compound (**4i + 4i'**)

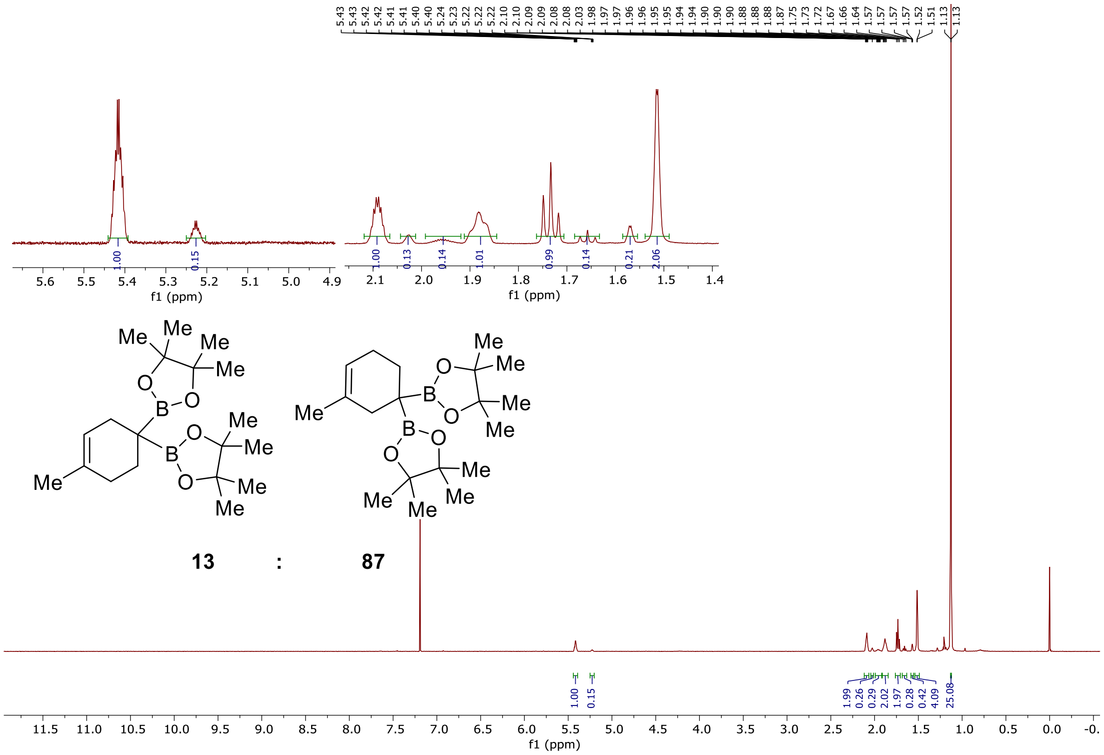

^{13}C NMR (101 MHz, $CDCl_3$) of compound (**4i + 4i'**)

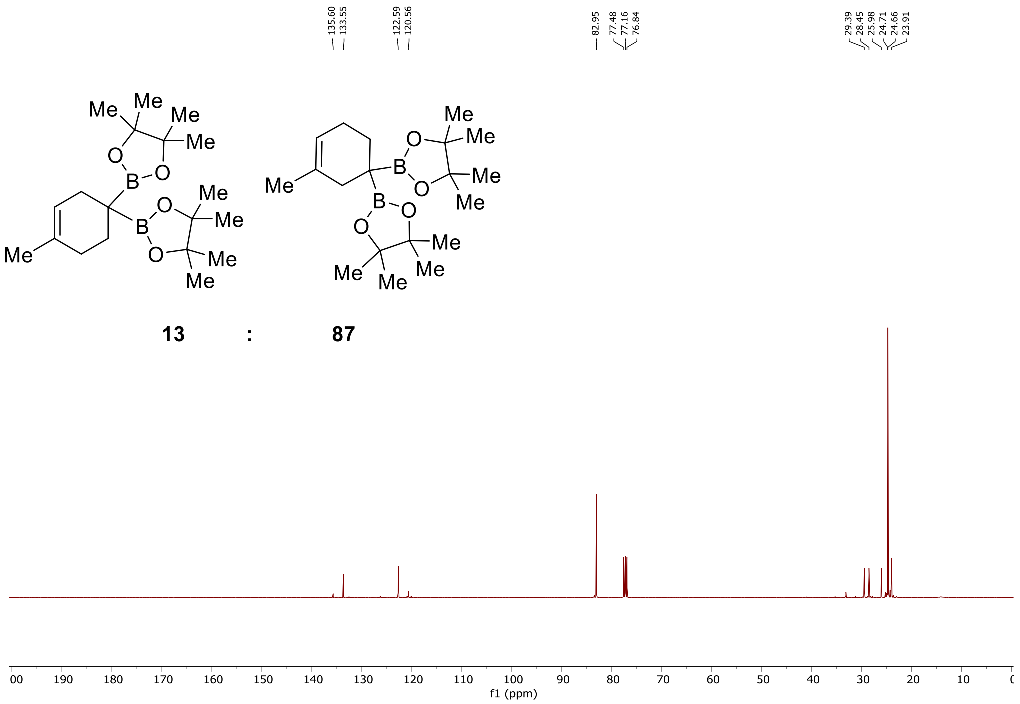

^{11}B NMR (128 MHz, $CDCl_3$) of compound (**4i + 4i'**)

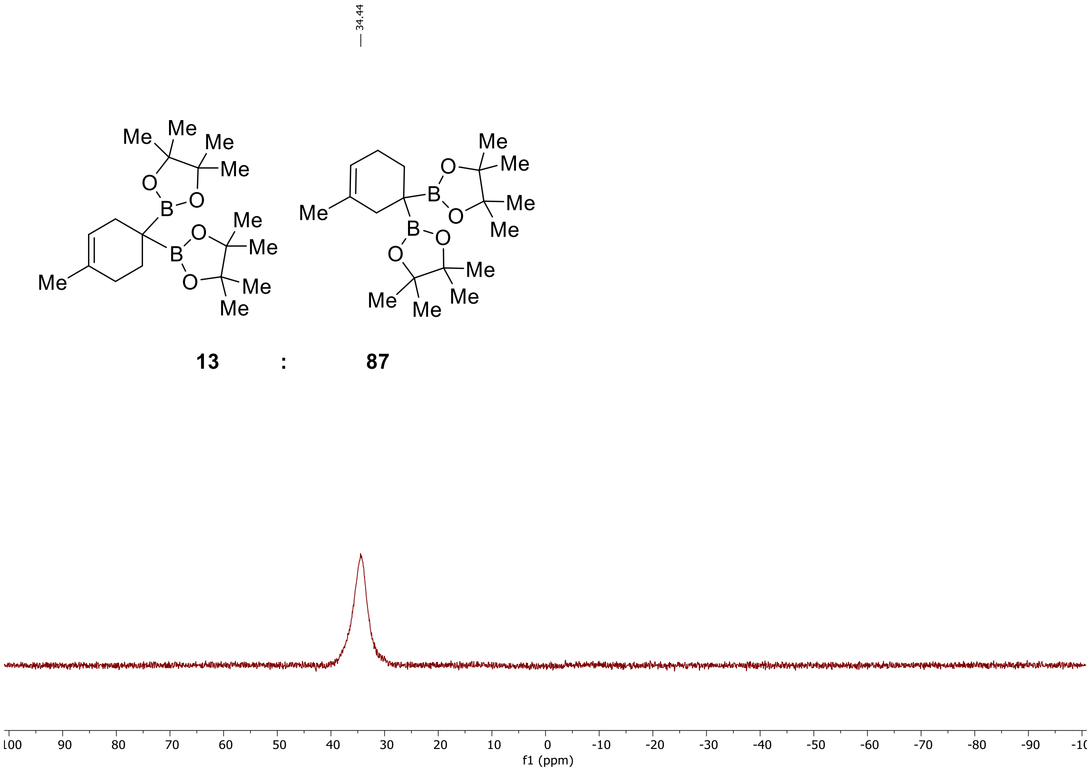

^{1}H NMR (400 MHz, $CDCl_3$) of compound (**4i**)

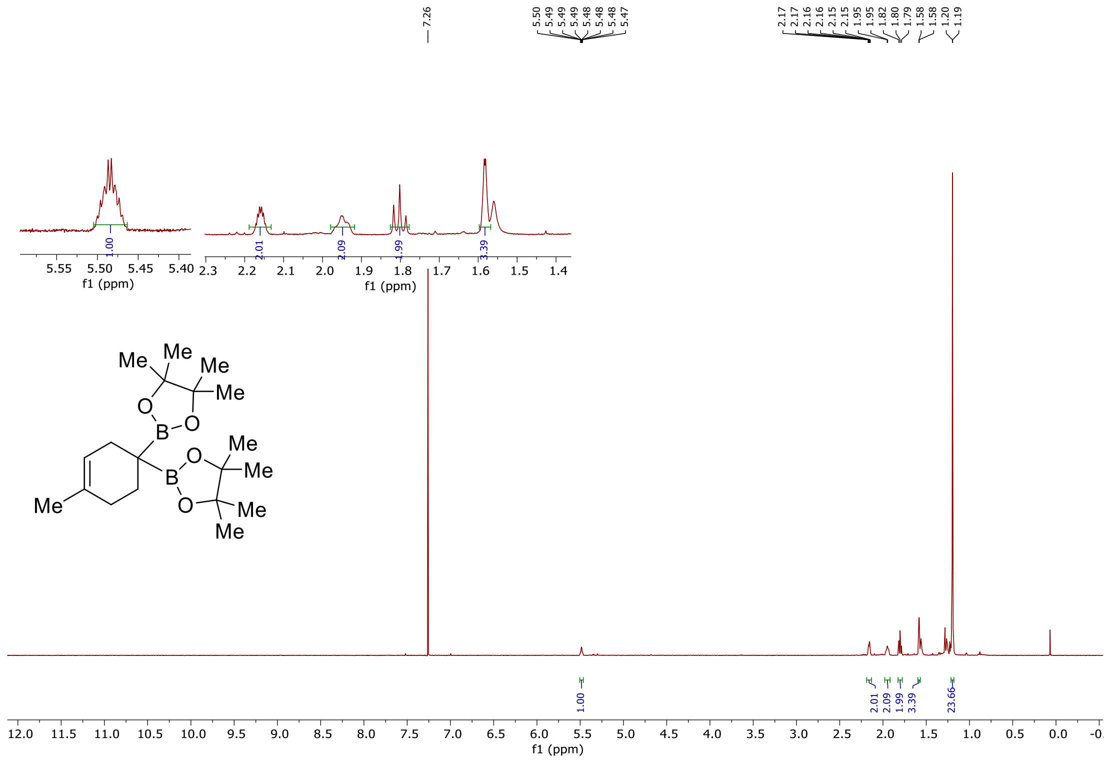

^{13}C NMR (126 MHz, $CDCl_3$) of compound (**4i**)

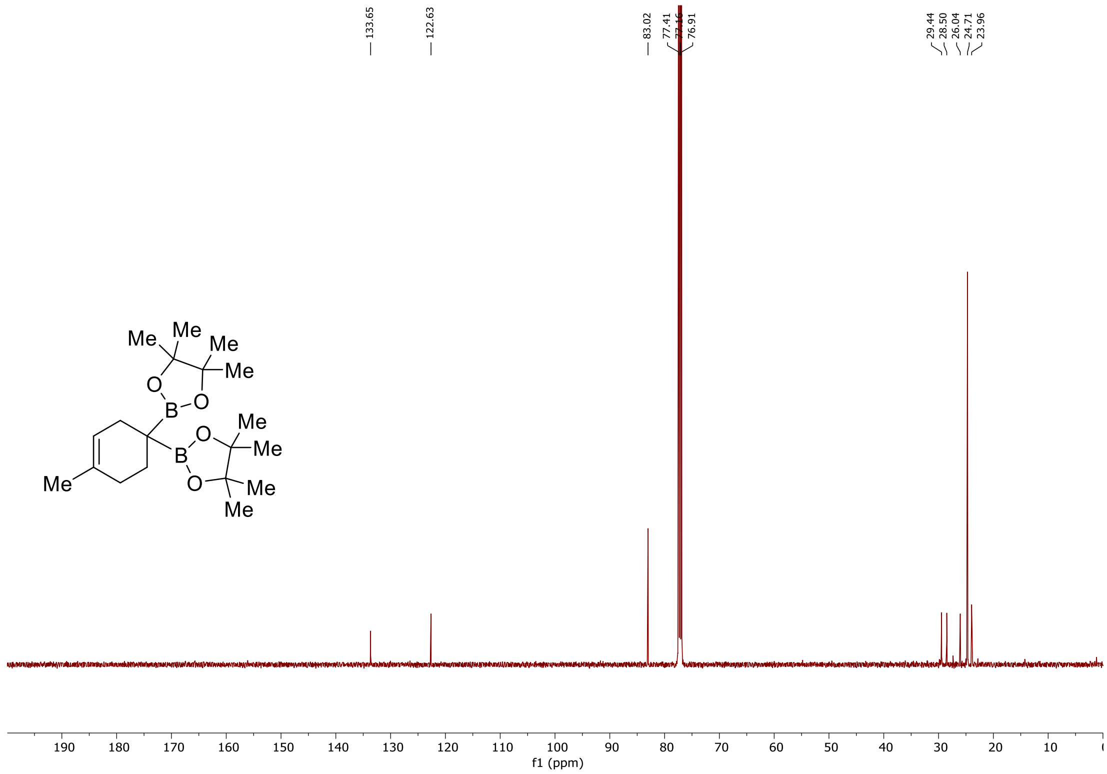

^{11}B NMR (128 MHz, $CDCl_3$) of compound (**4i**)

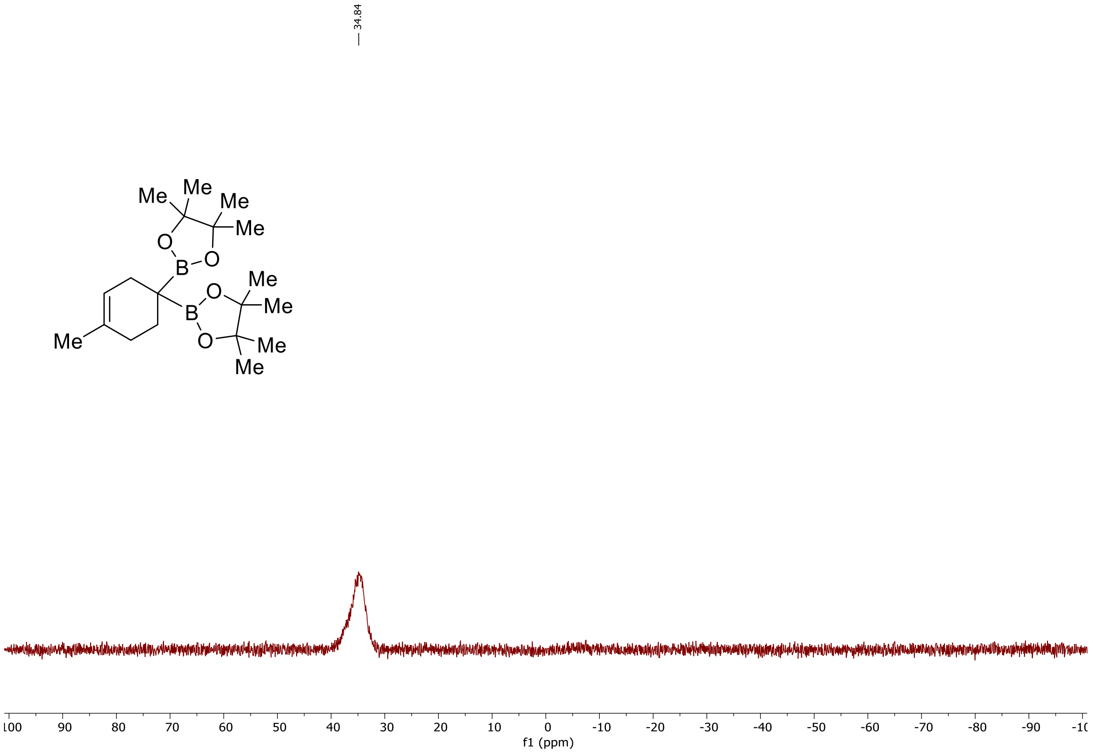

^{1}H NMR (400 MHz, $CDCl_3$) of compound (**4j + 4j'**)

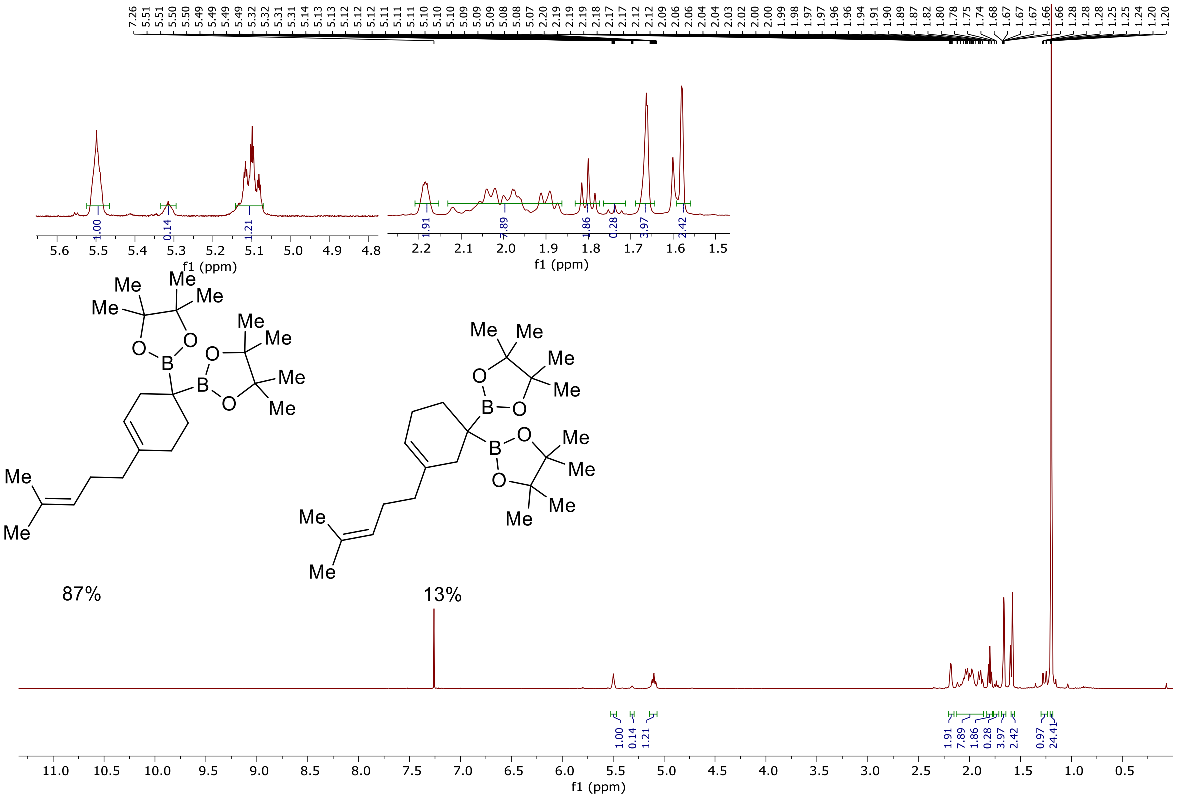

^{13}C NMR (126MHz, CDCl$_3$) of compound (**4j** + **4j'**)

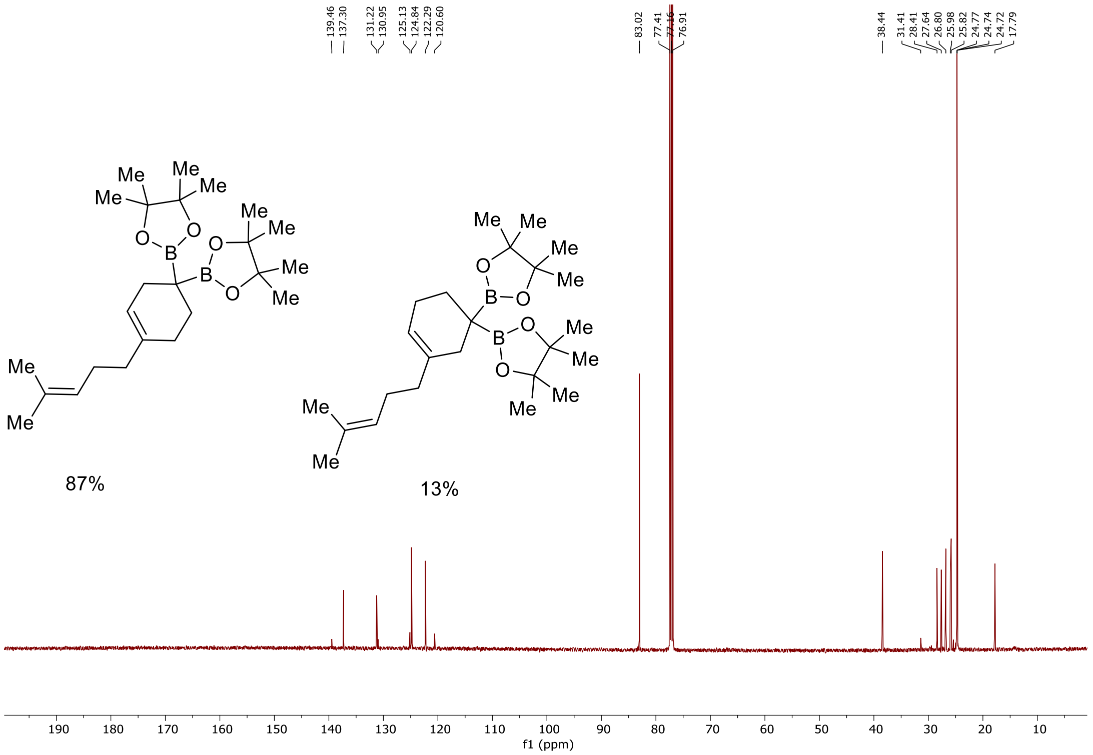

^{11}B NMR (128 MHz, CDCl$_3$) of compound (**4j** + **4j'**)

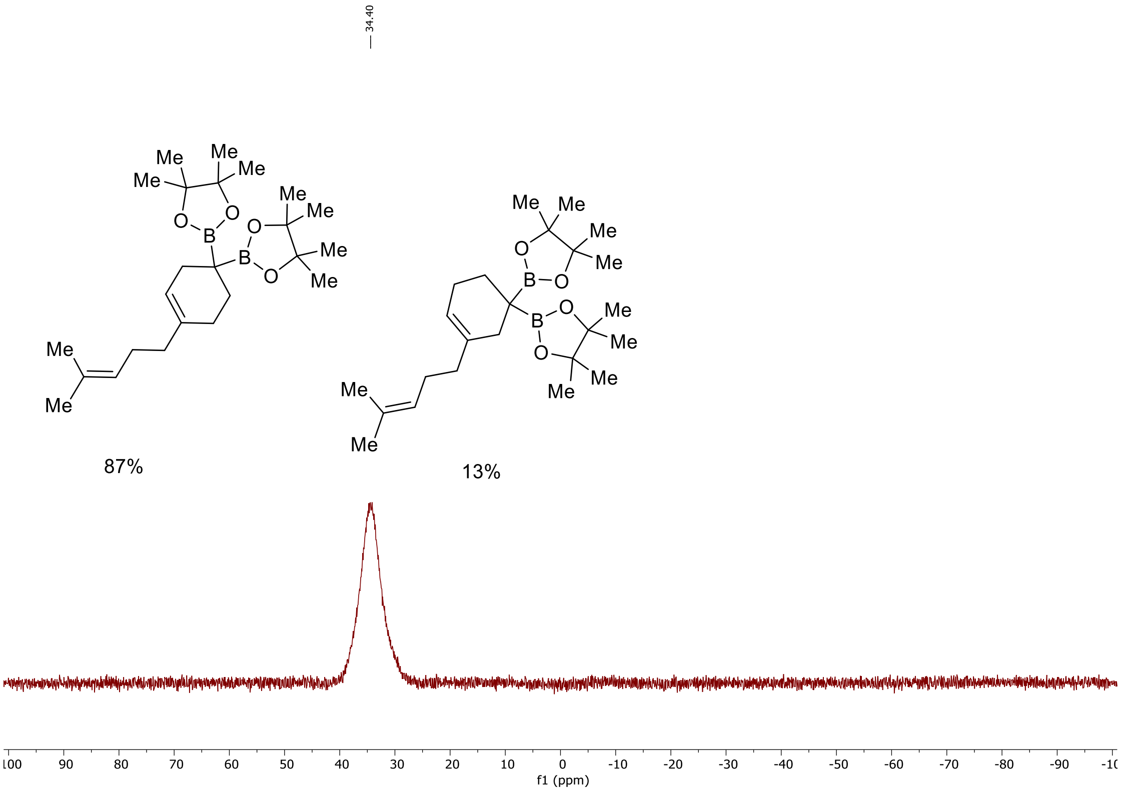

^{1}H NMR (400 MHz, $CDCl_3$) of compound (**4k**)

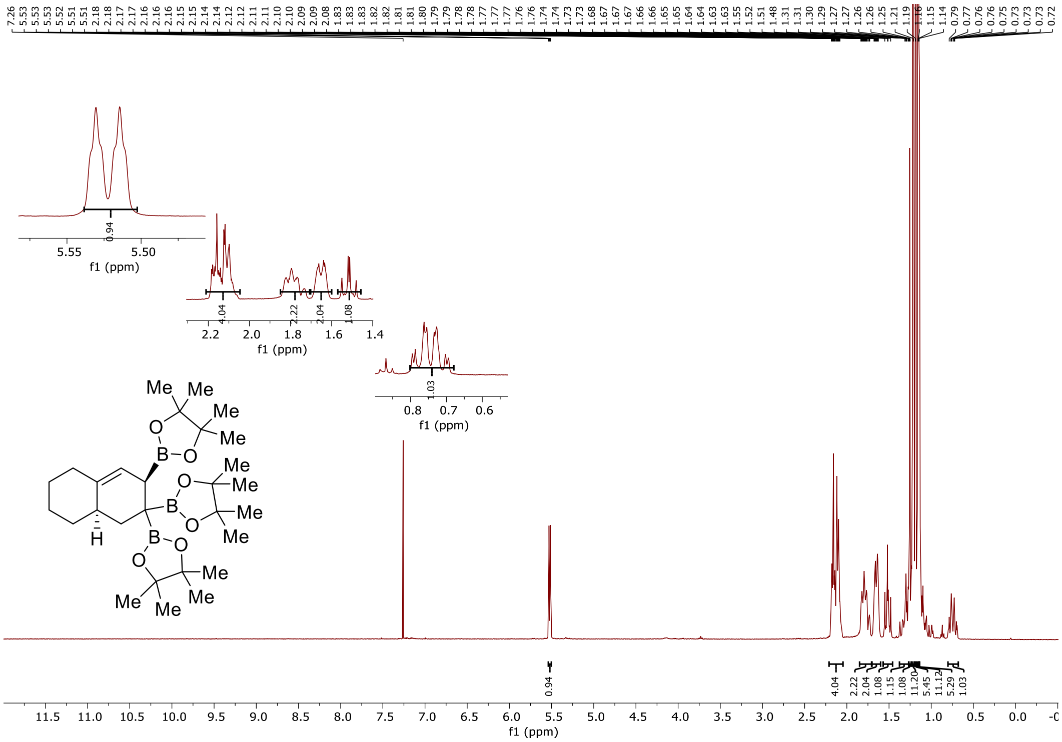

^{13}C NMR (126MHz, $CDCl_3$) of compound (**4k**)

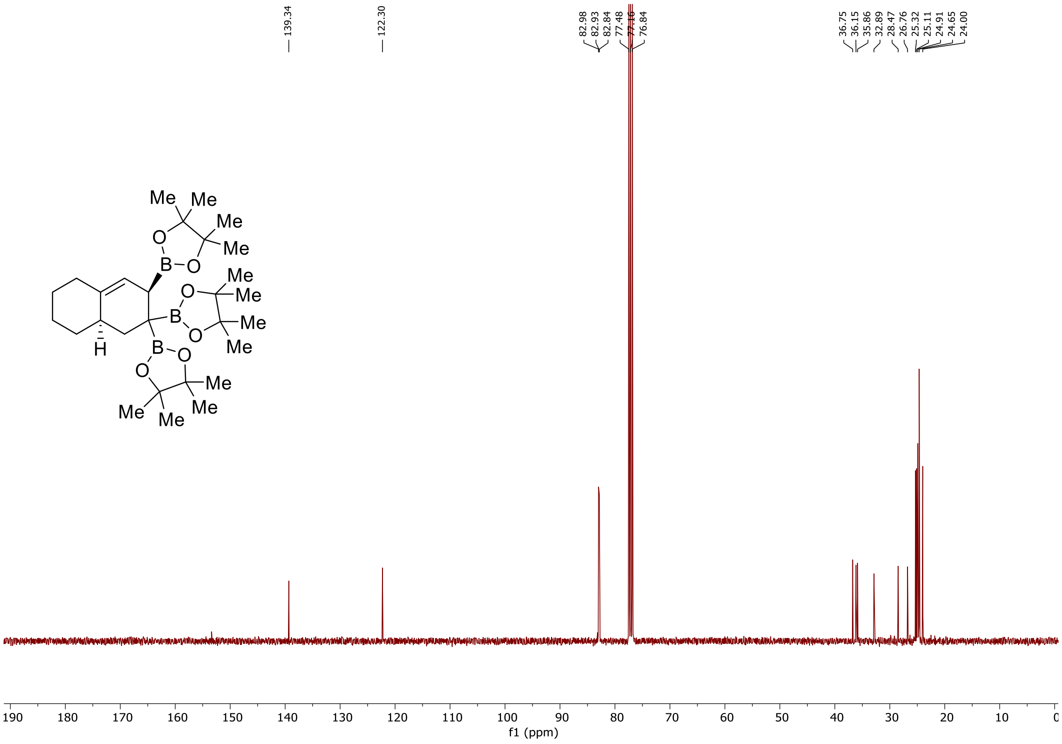

^{11}B NMR (128 MHz, $CDCl_3$) of compound (**4k**)

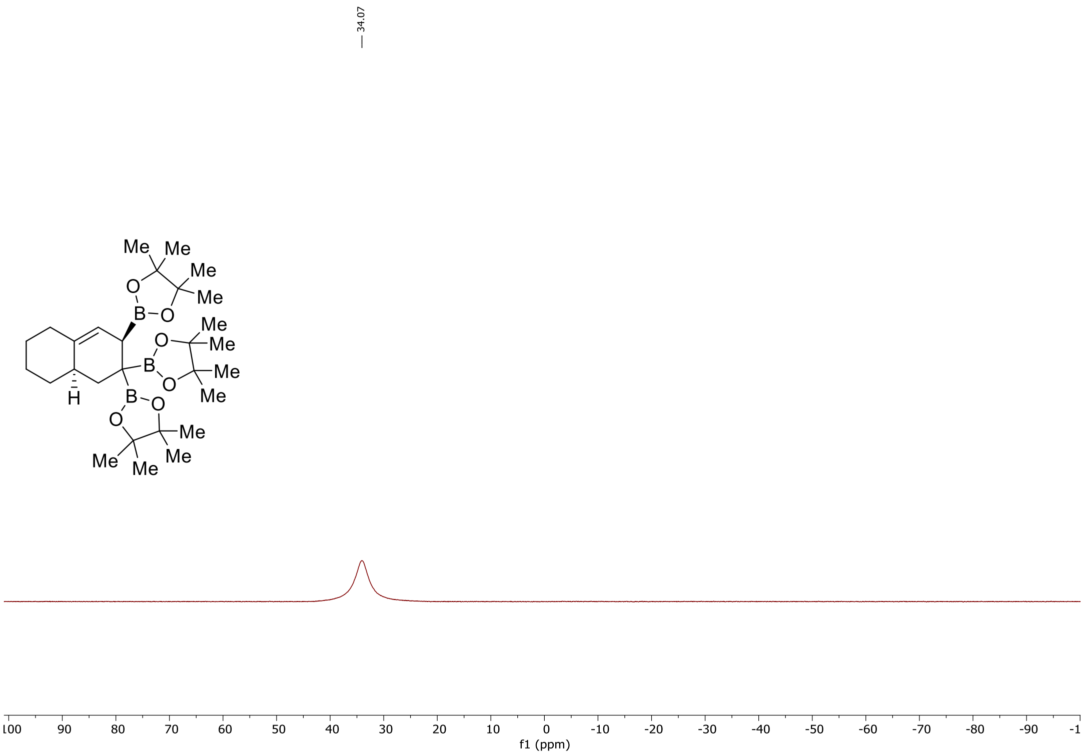

^{1}H NMR (500 MHz, $CDCl_3$) of compound (**4l**)

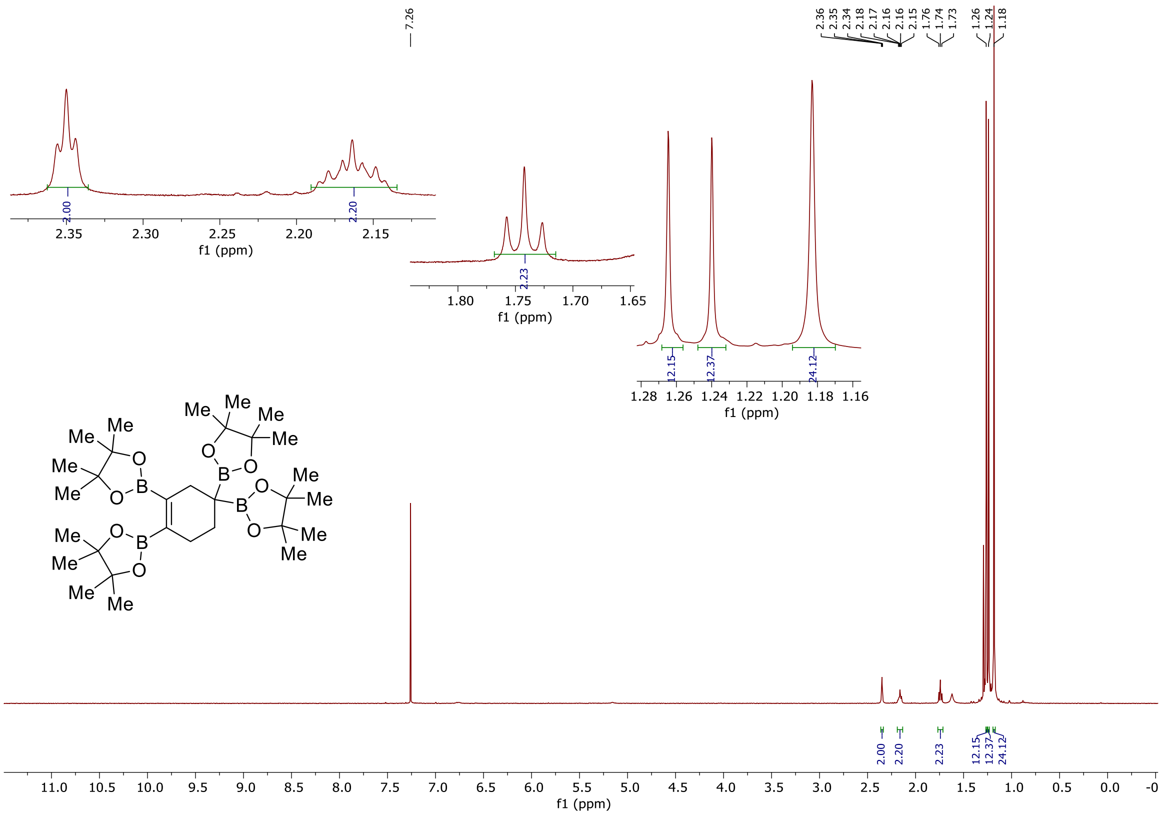

^{13}C NMR (126 MHz, $CDCl_3$) of compound (**4l**)

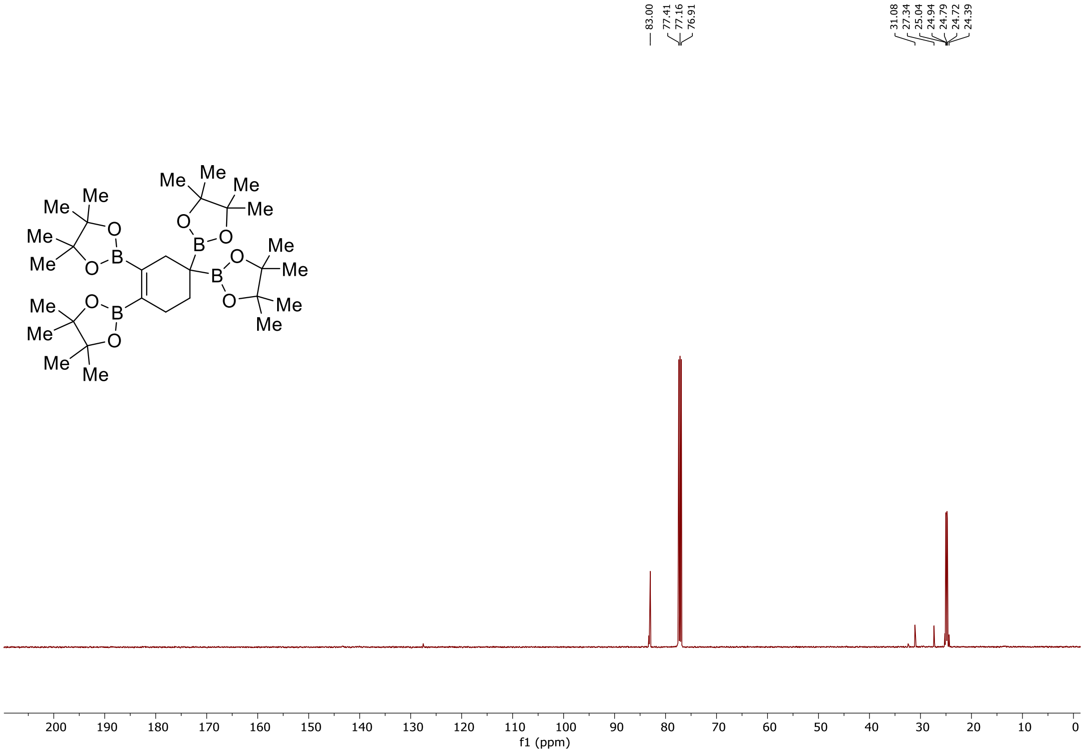

^{11}B NMR (128 MHz, $CDCl_3$) of compound (**4l**)

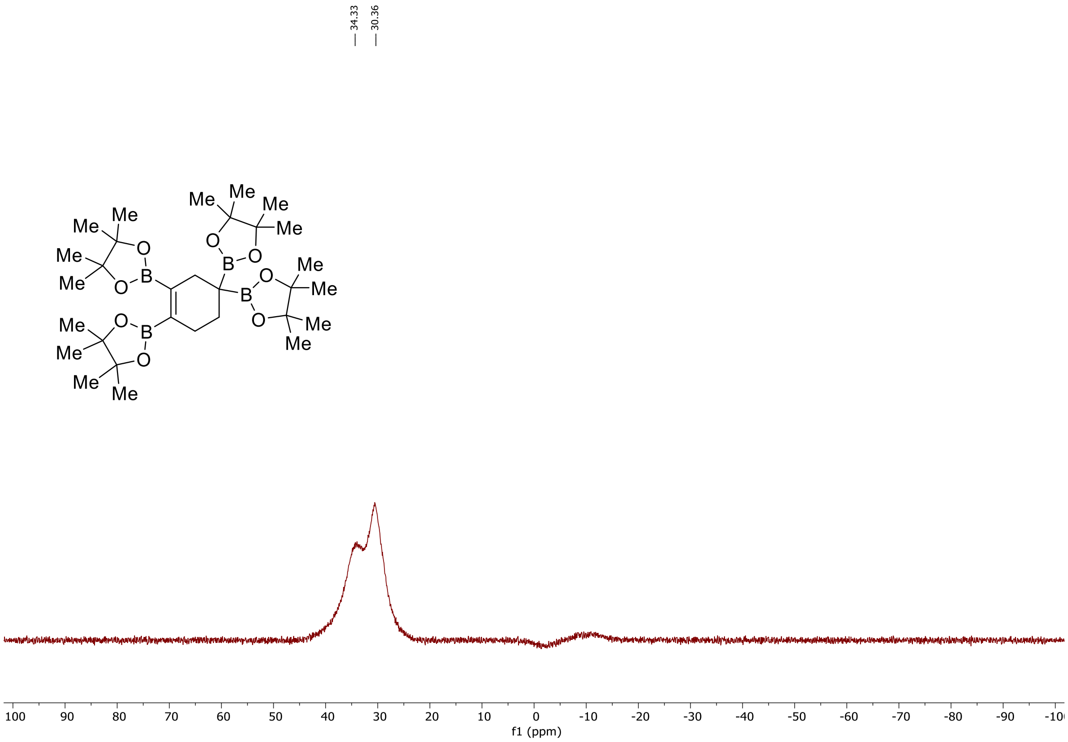

^{1}H NMR (400 MHz, $CDCl_3$) of compound (**5a**)

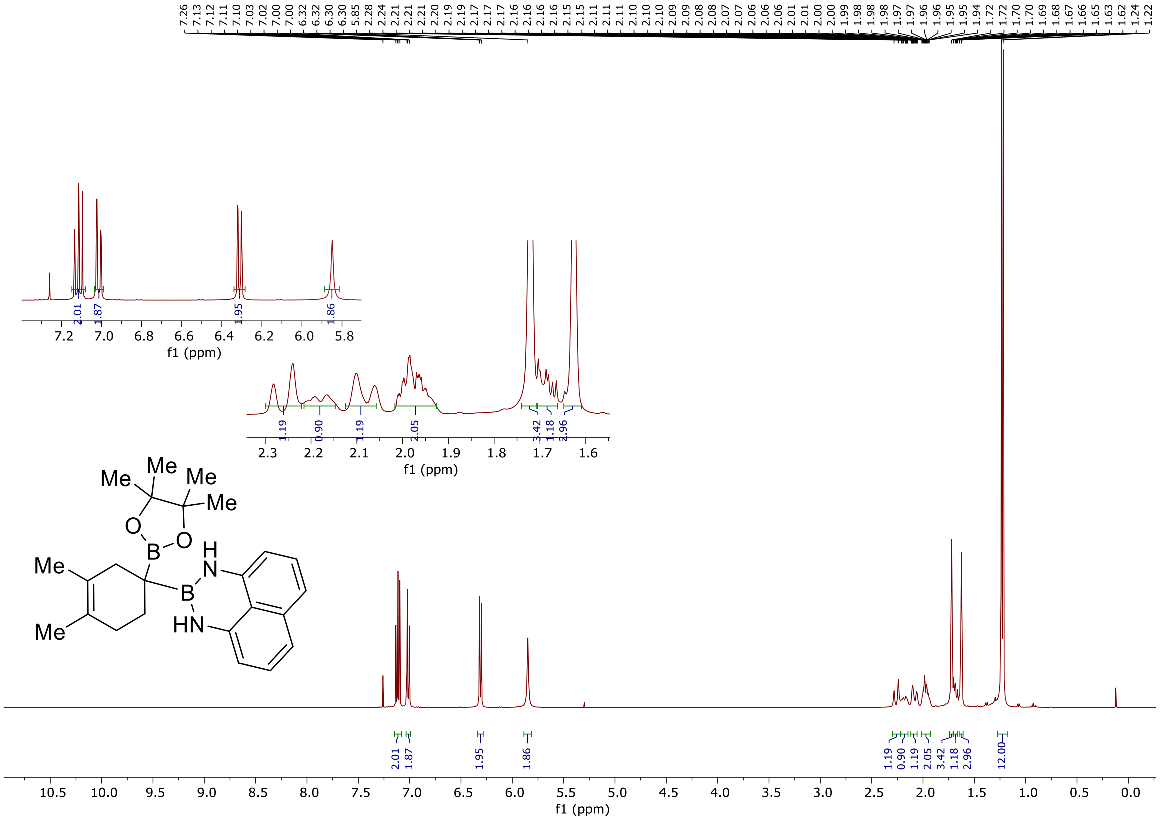

^{13}C NMR (101 MHz, $CDCl_3$) of compound (**5a**)

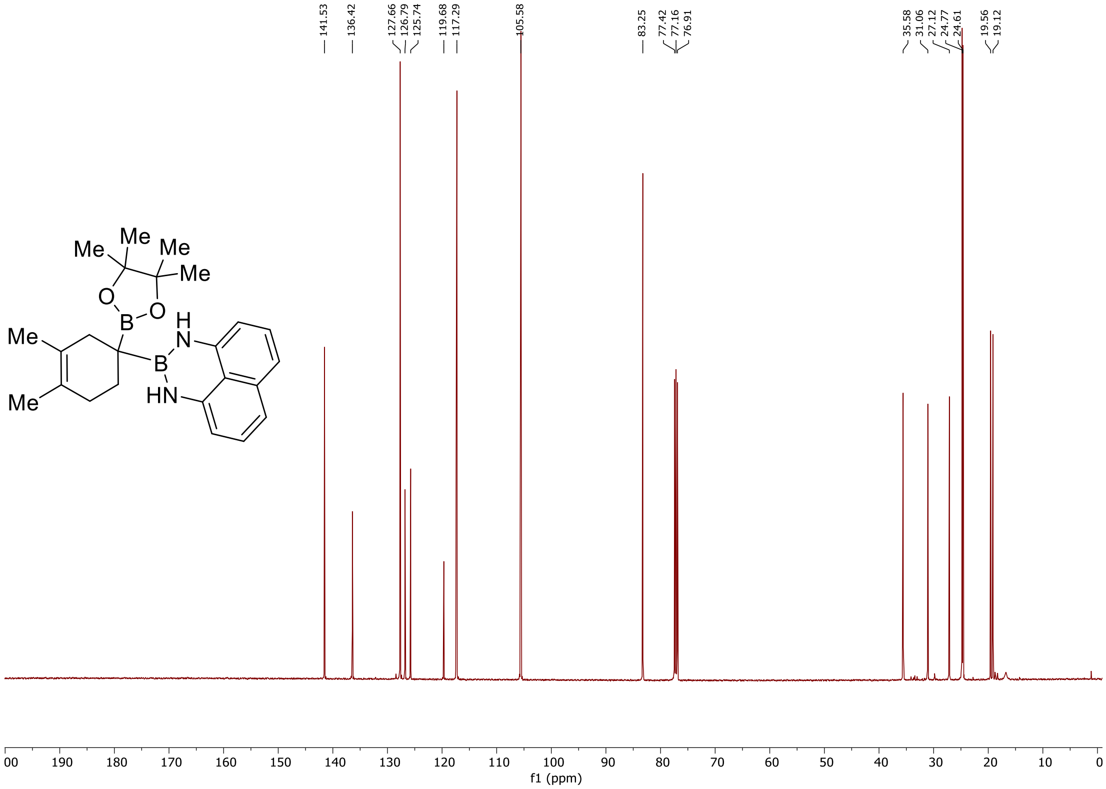

^{11}B NMR (128 MHz, $CDCl_3$) of compound (**5a**)

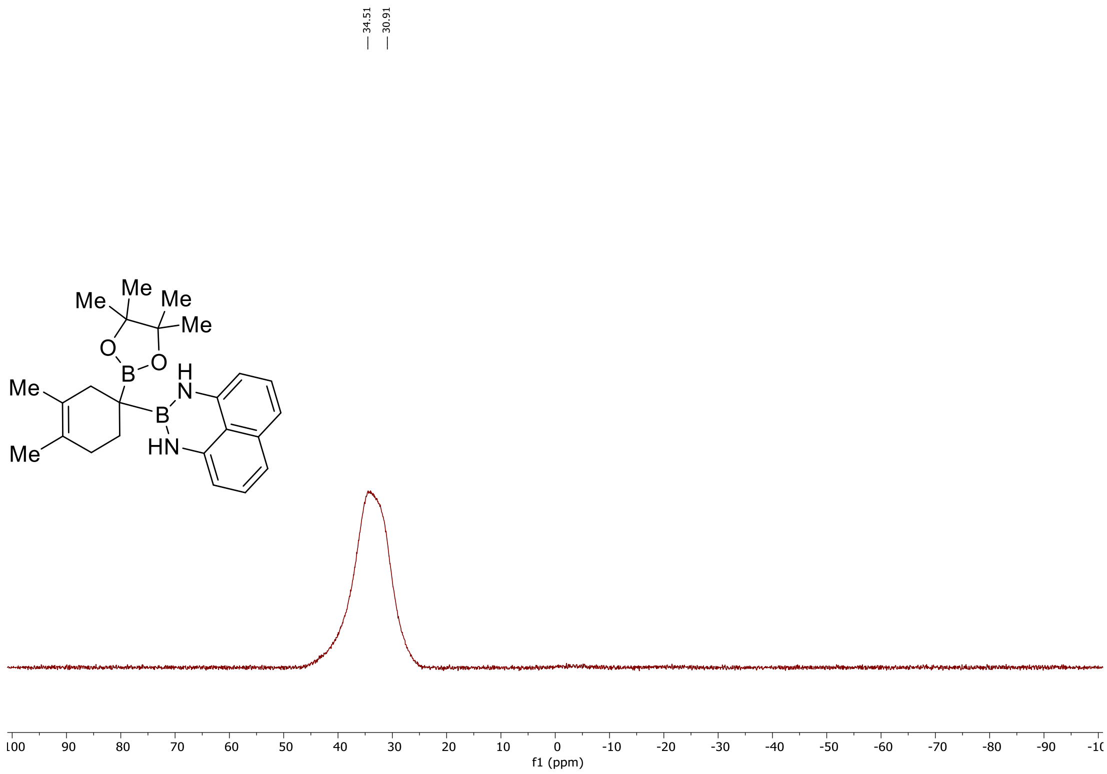

^{1}H NMR (400 MHz, $CDCl_3$) of compound (**5b**)

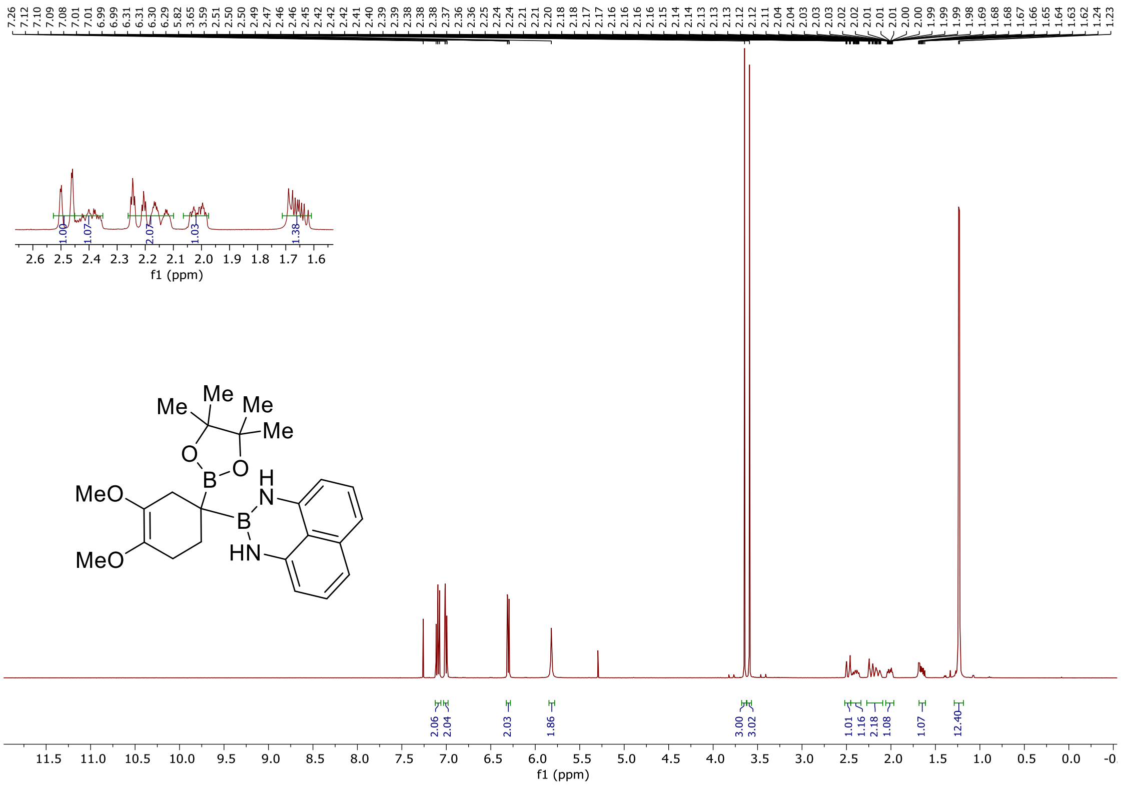

^{13}C NMR (126MHz, CD_3CN) of compound (**5b**)

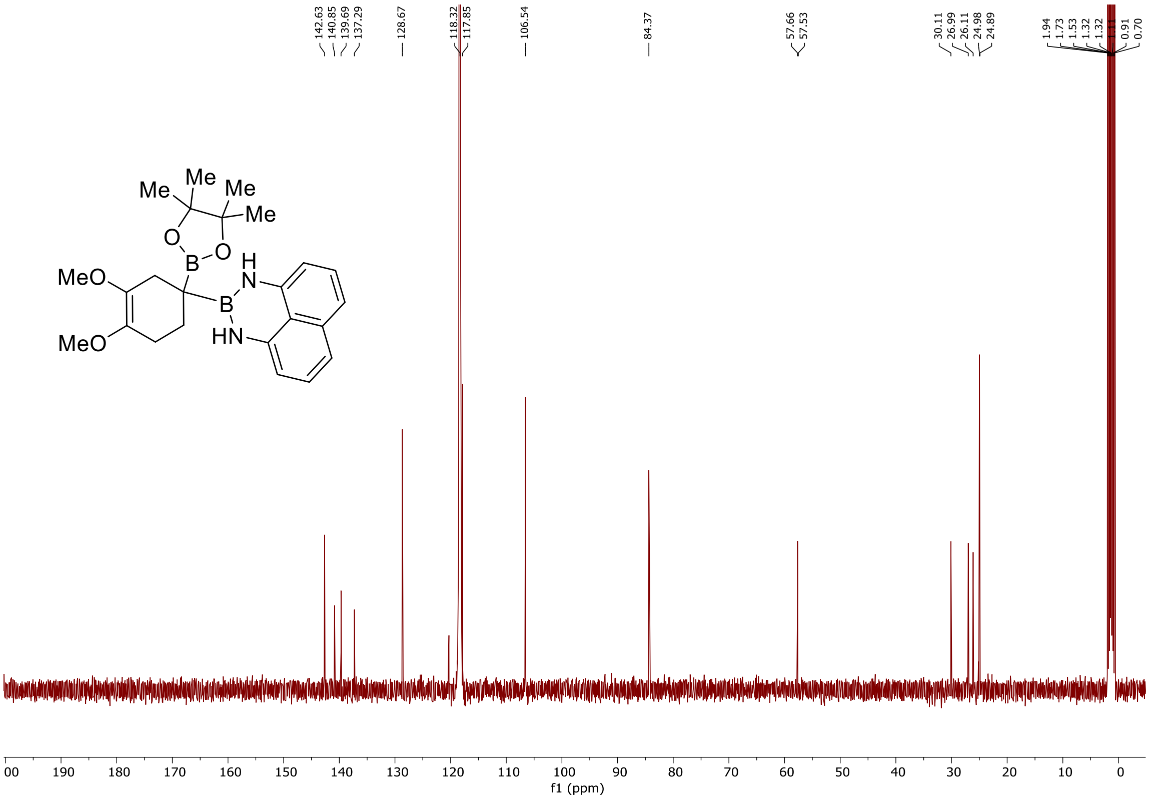

^{11}B NMR (128 MHz, $CDCl_3$) of compound (**5b**)

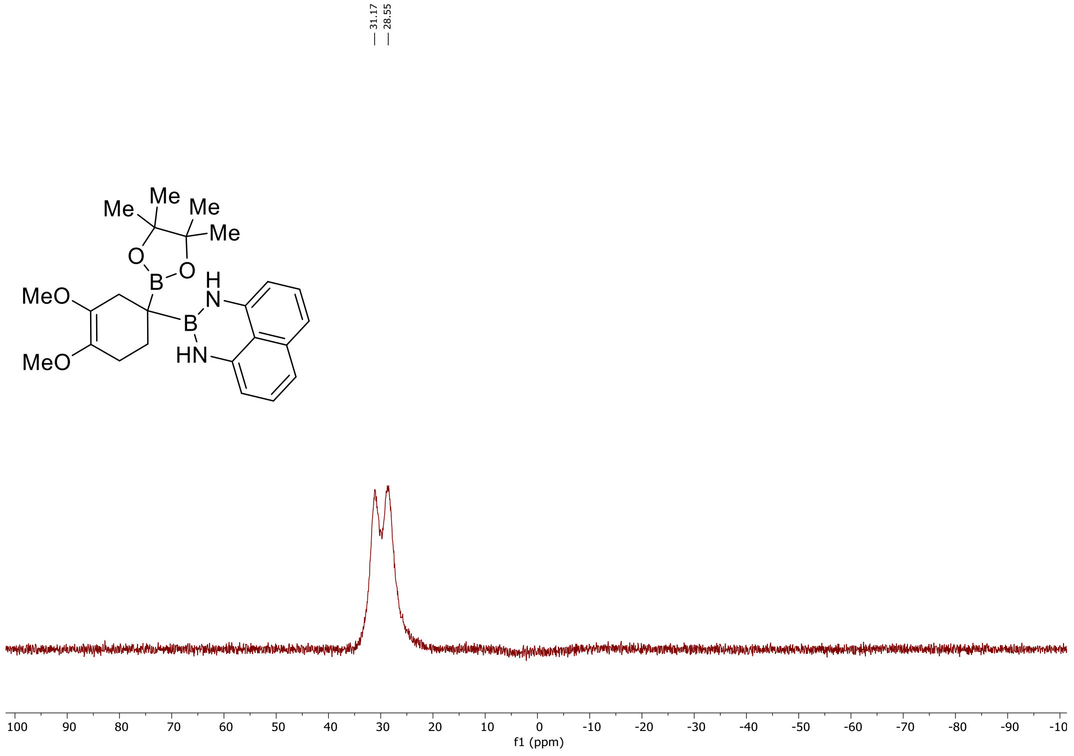

^{1}H NMR (400 MHz, $CDCl_3$) of compound (**5c**)

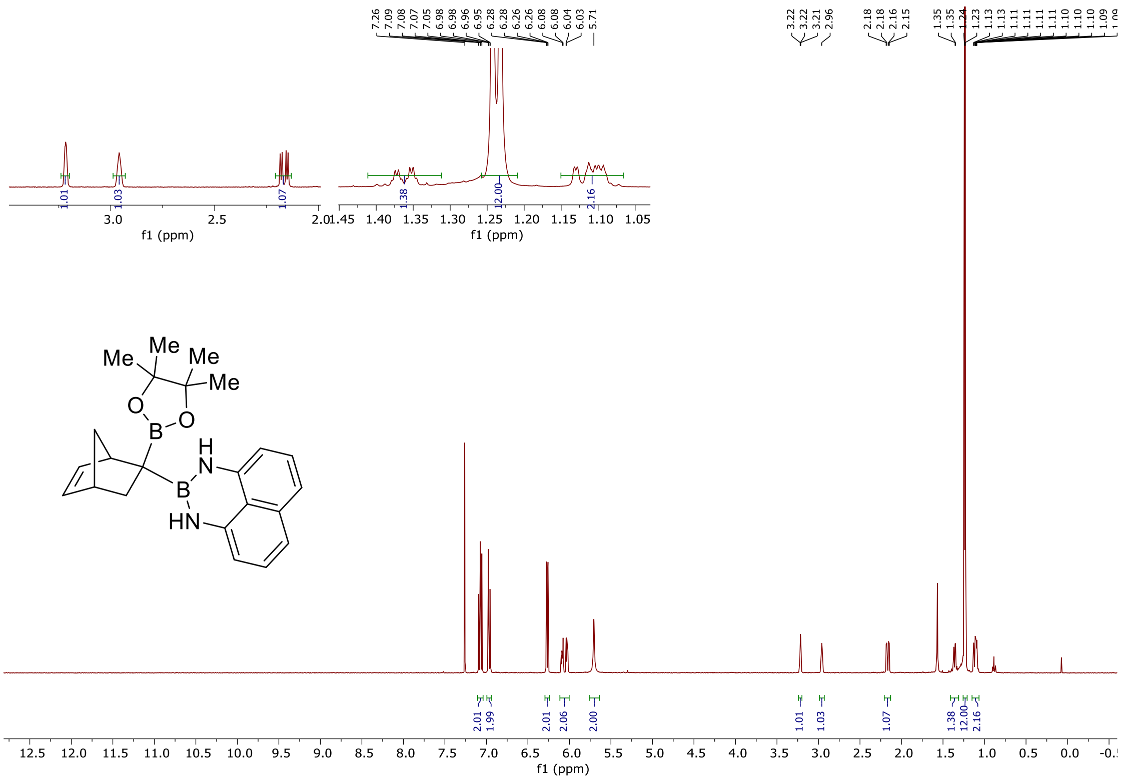

^{13}C NMR (126 MHz, $CDCl_3$) of compound (**5c**)

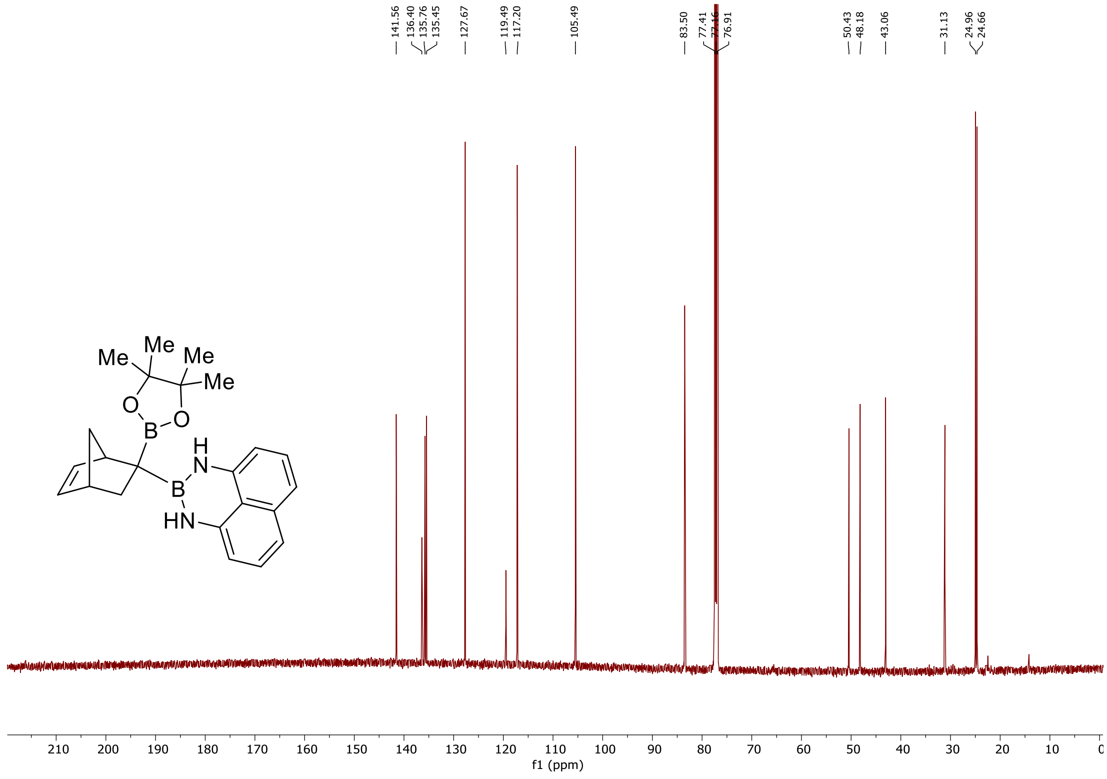

^{11}B NMR (128 MHz, $CDCl_3$) of compound (**5c**)

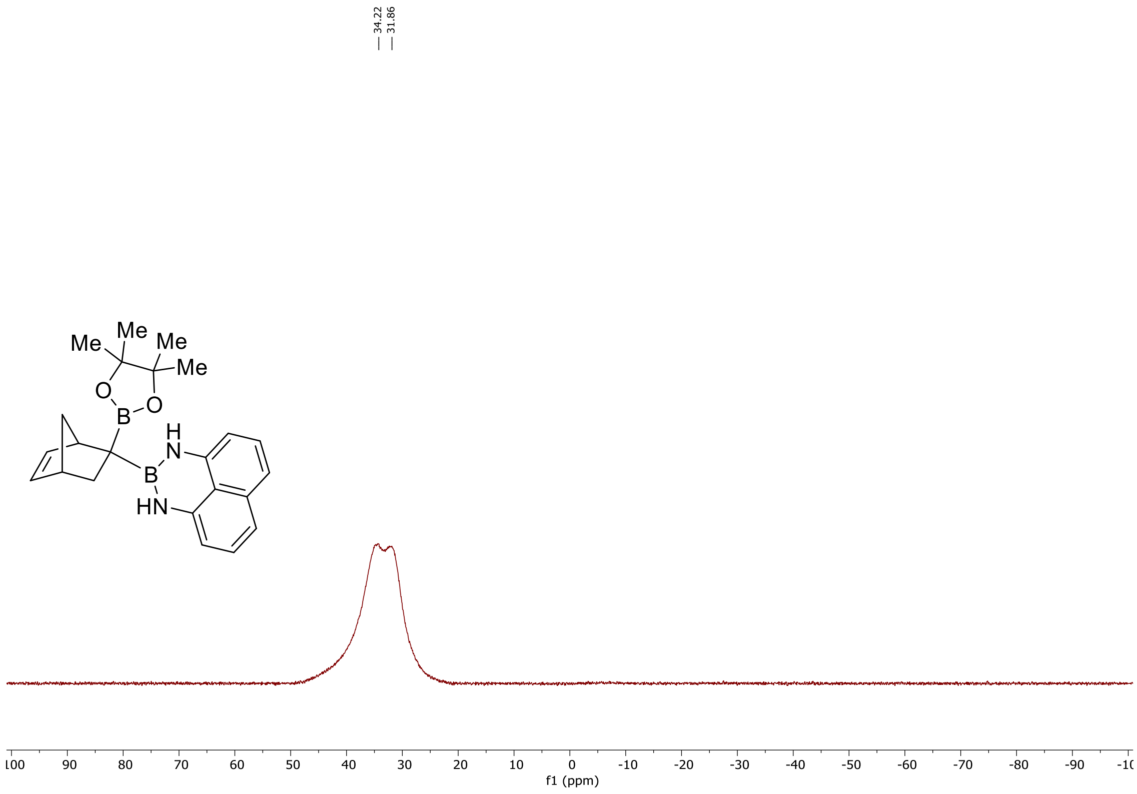

^{1}H NMR (400 MHz, $CDCl_3$) of compound (**5d**)

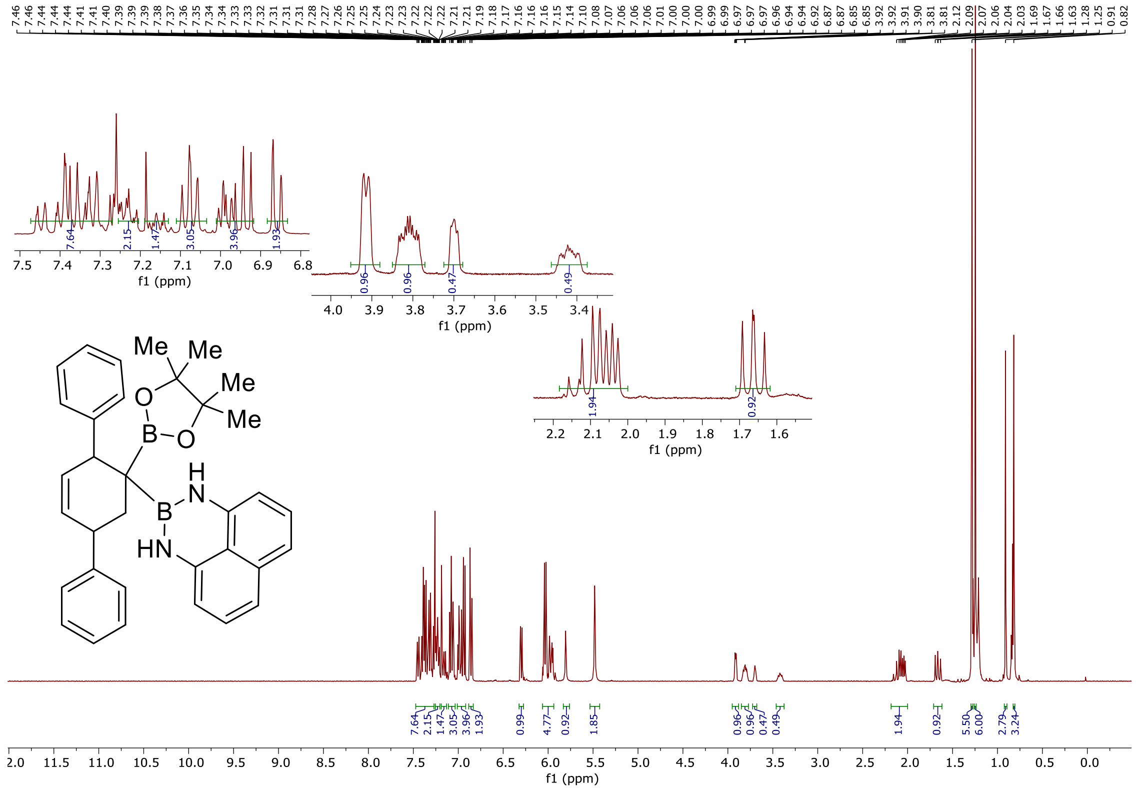

^{13}C NMR (126 MHz, $CDCl_3$) of compound (**5d**)

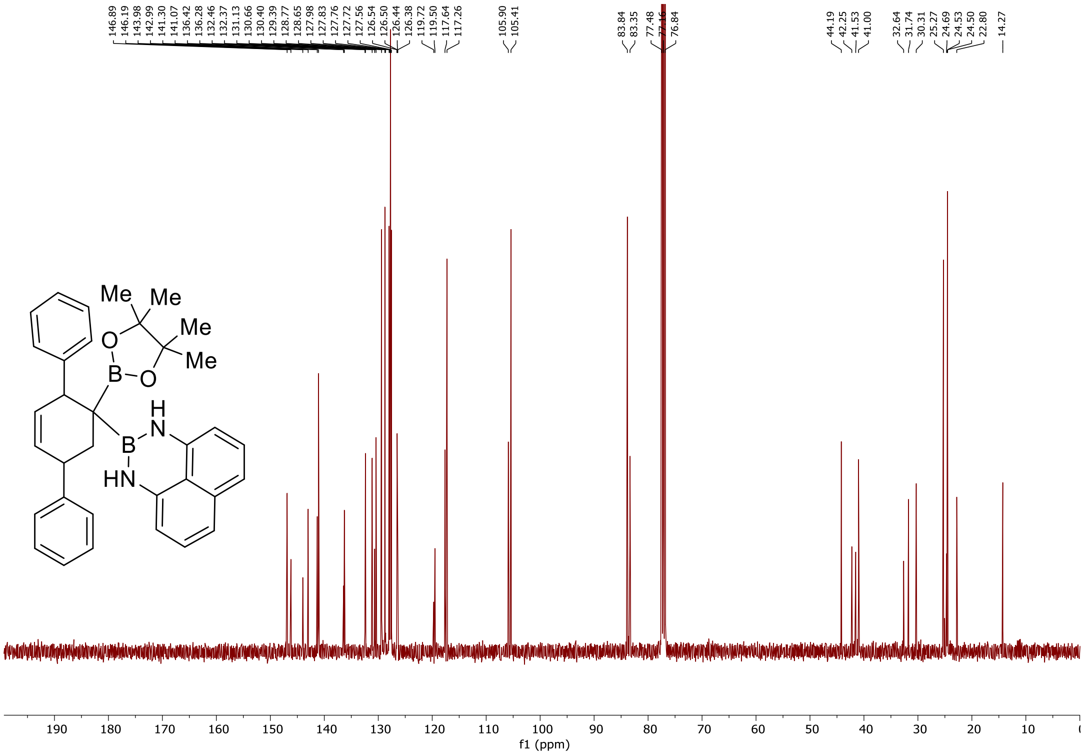

^{11}B NMR (128 MHz, $CDCl_3$) of compound (**5d**)

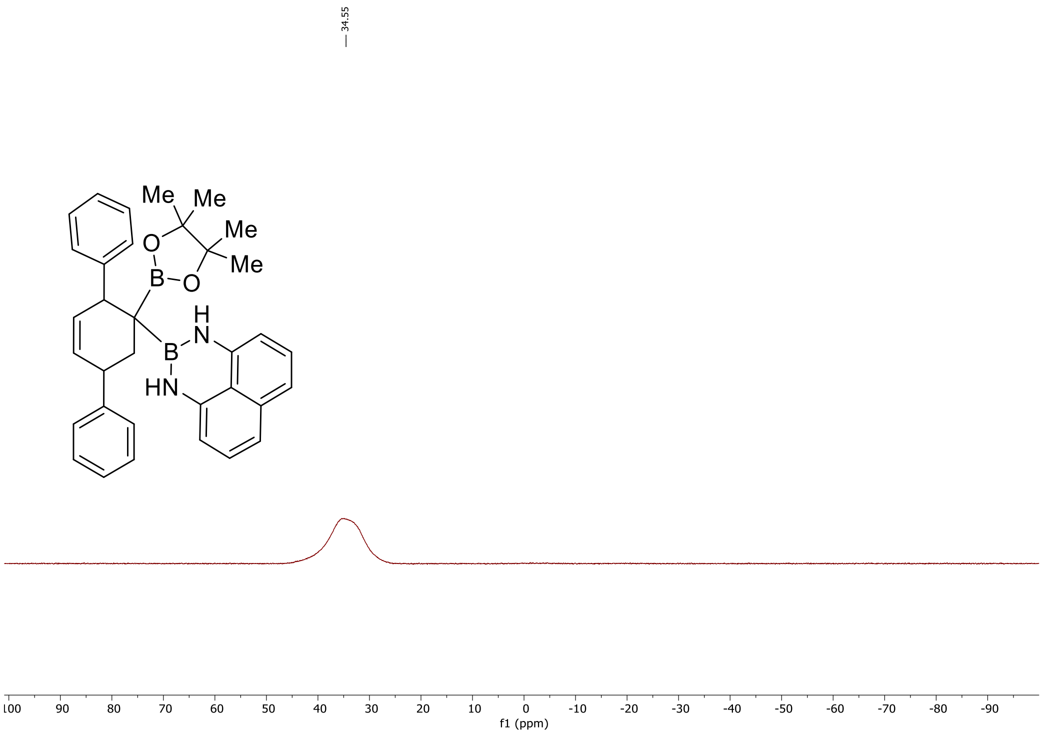

^{1}H NMR (500 MHz, $CDCl_3$) of compound (**5f-*endo***)

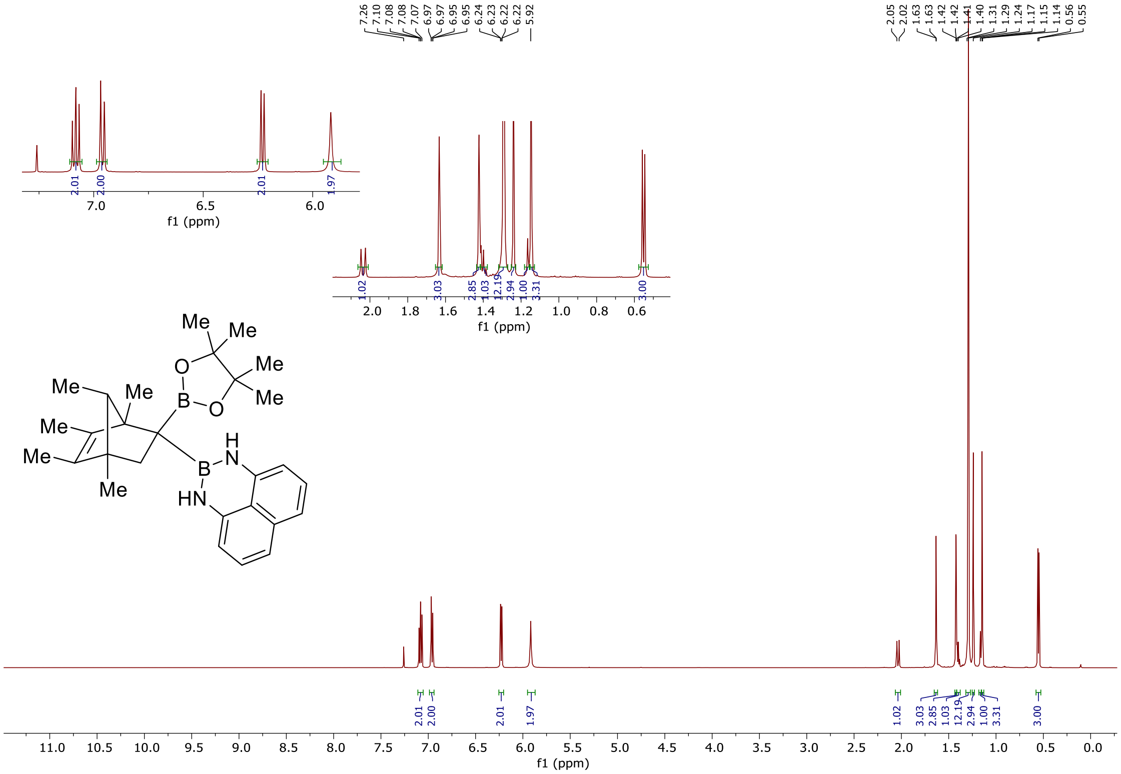

^{13}C NMR (126 MHz, $CDCl_3$) of compound (**5f-*endo***)

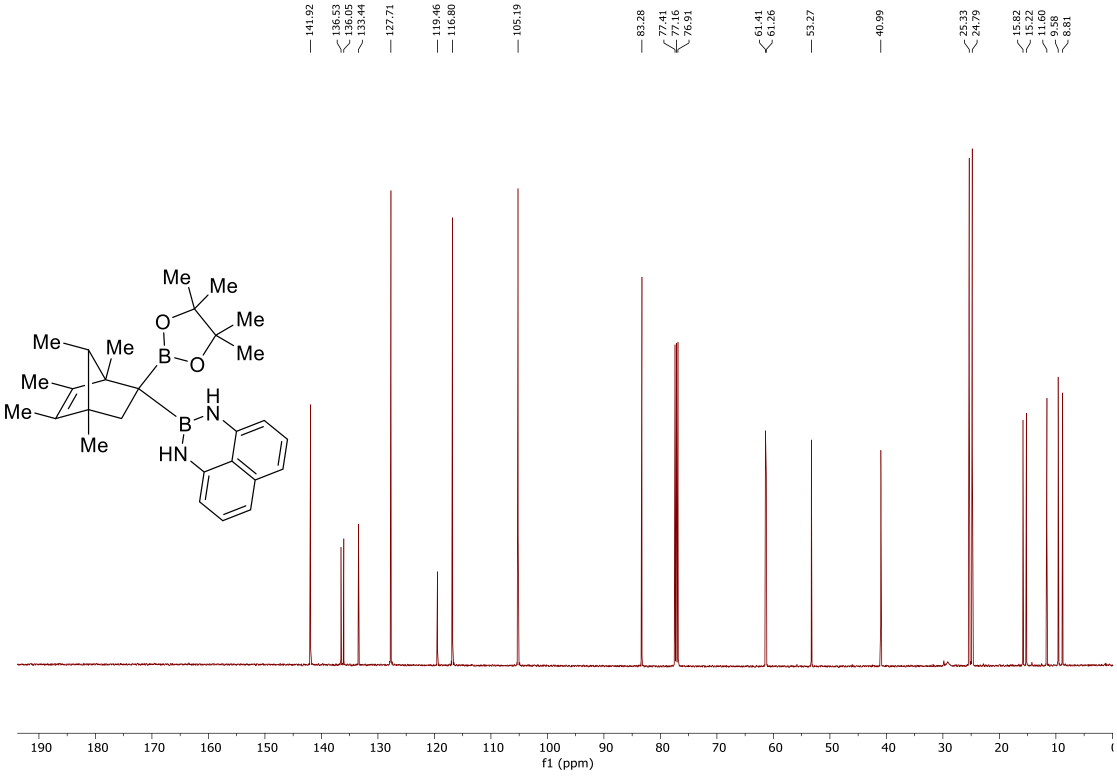

^{11}B NMR (128 MHz, $CDCl_3$) of compound (**5f-*endo***)

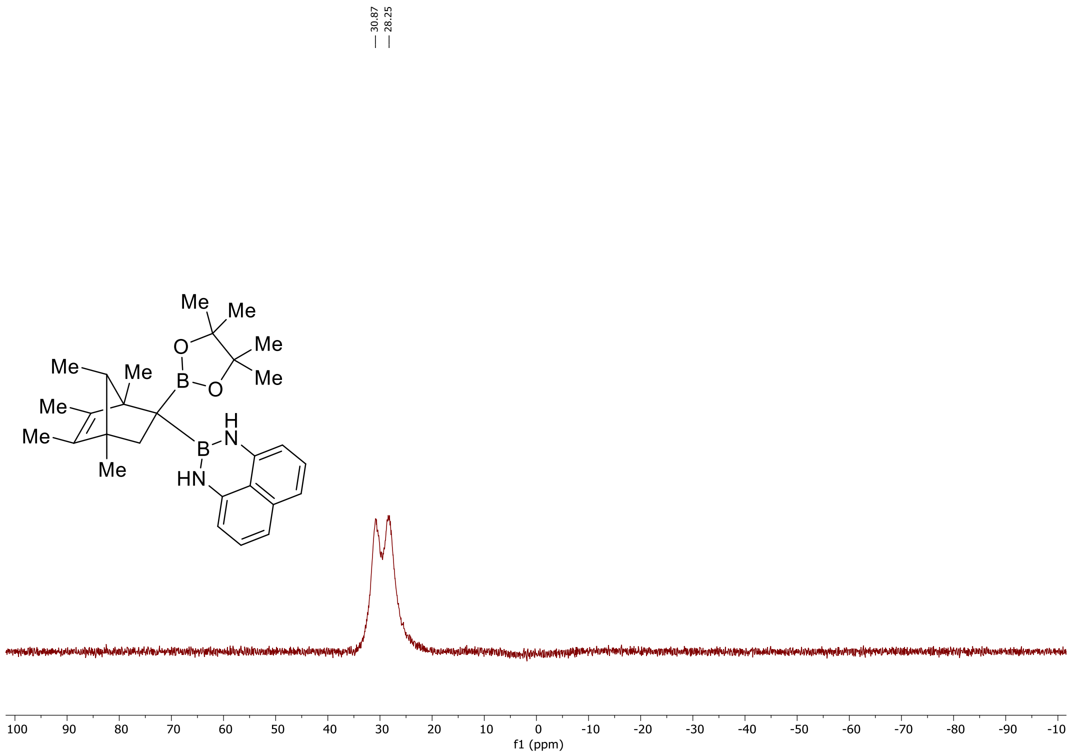

^{1}H NMR (500 MHz, $CDCl_3$) of compound (**5l**)

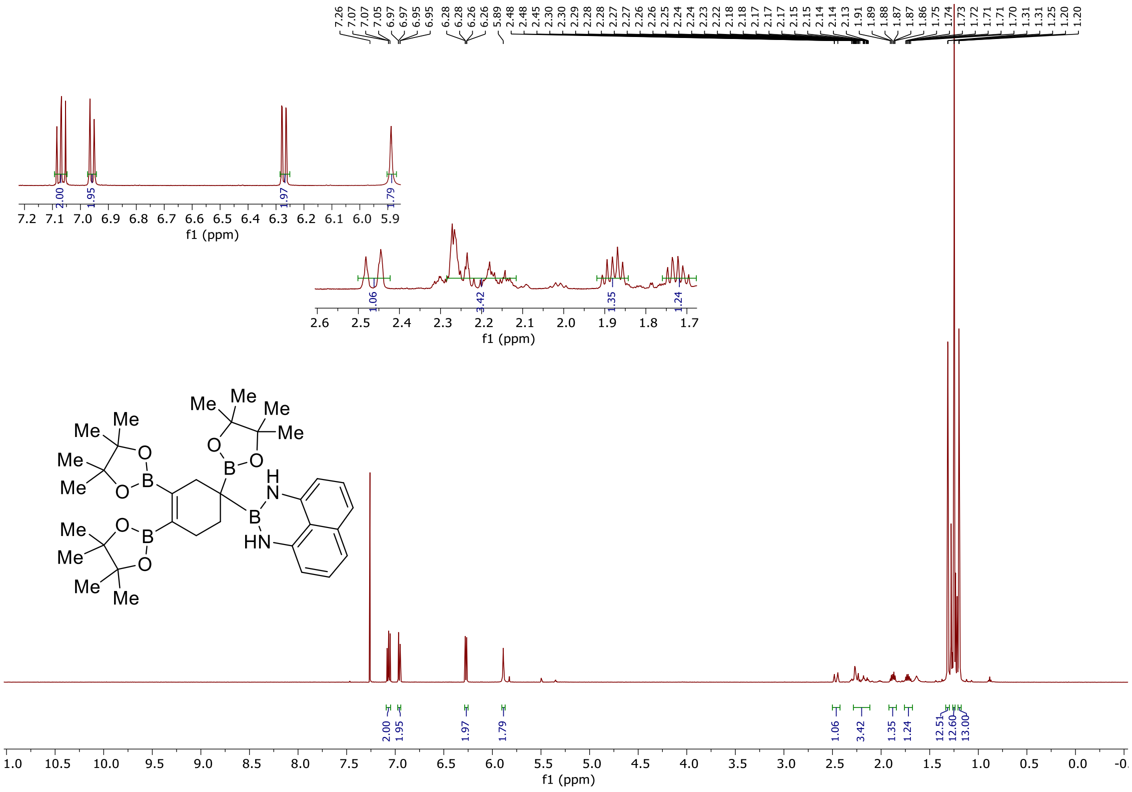

^{13}C NMR (126 MHz, $CDCl_3$) of compound (**5l**)

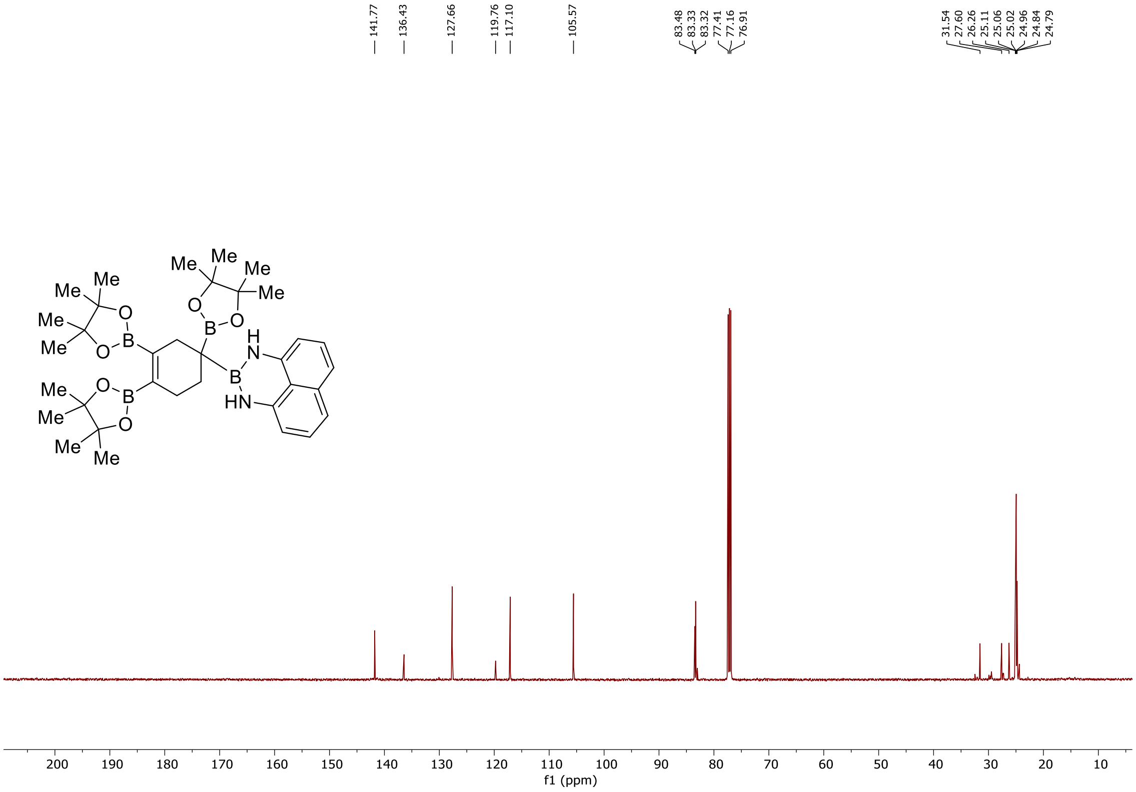

^{11}B NMR (128 MHz, $CDCl_3$) of compound (**5l**)

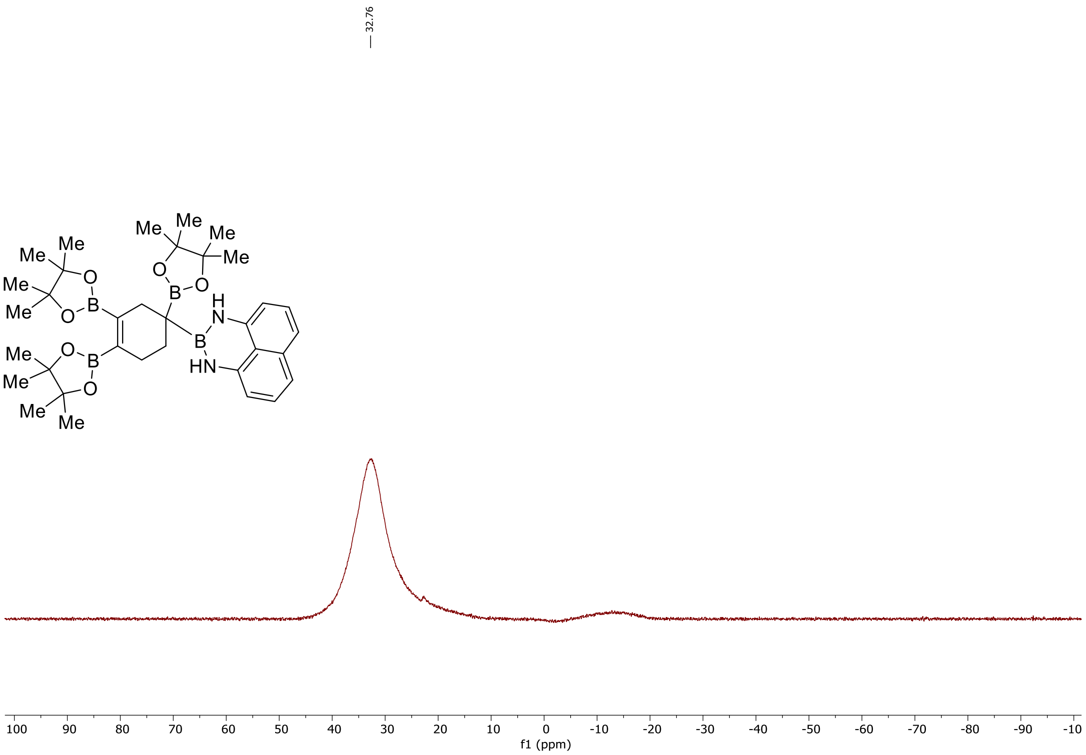

^{1}H NMR (400 MHz, $CDCl_3$) of compound (**5k**)

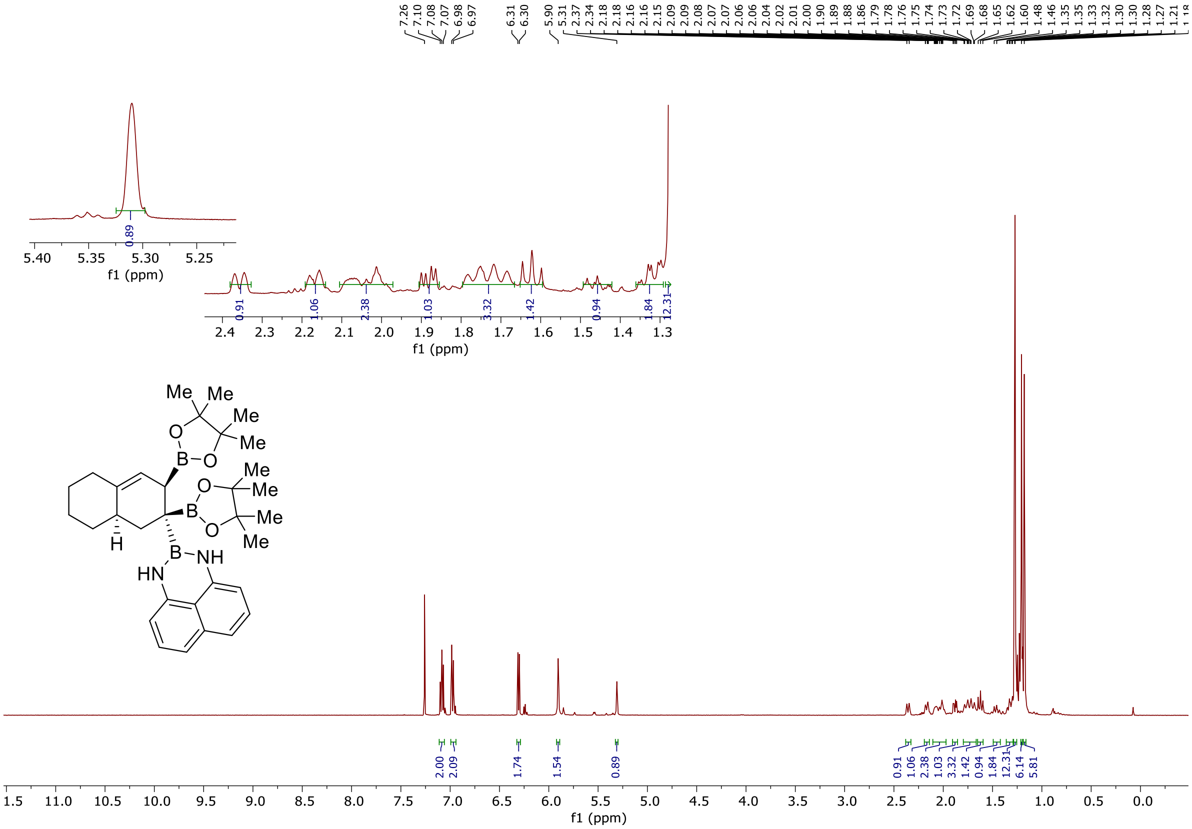

^{13}C NMR (126 MHz, $CDCl_3$) of compound (**5k**)

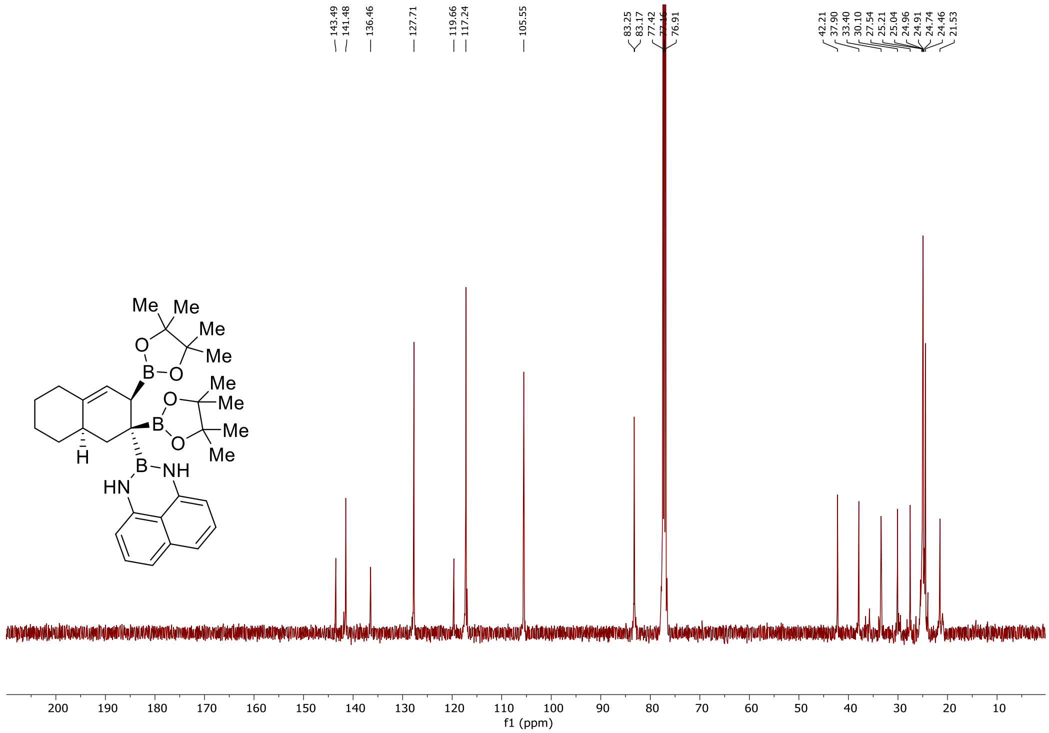

^{11}B NMR (128 MHz, $CDCl_3$) of compound (**5k**)

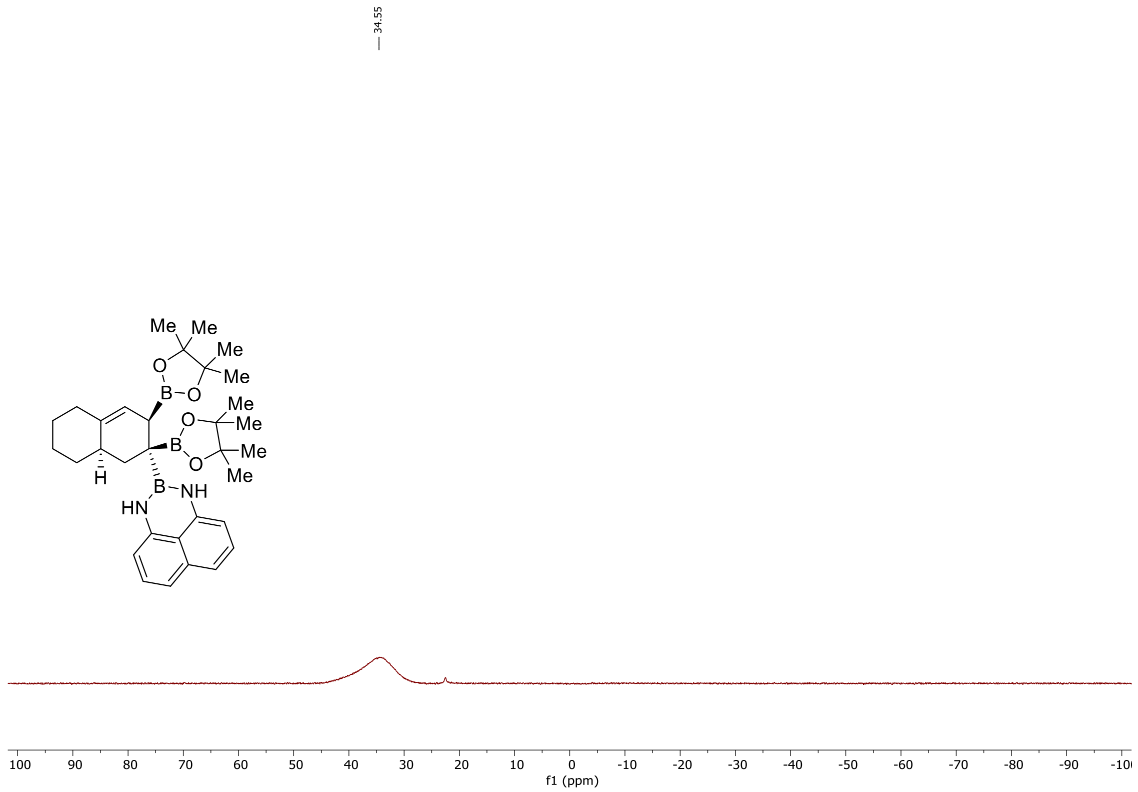

^{1}H NMR (400 MHz, cdcl3) of compound (**5k'**)

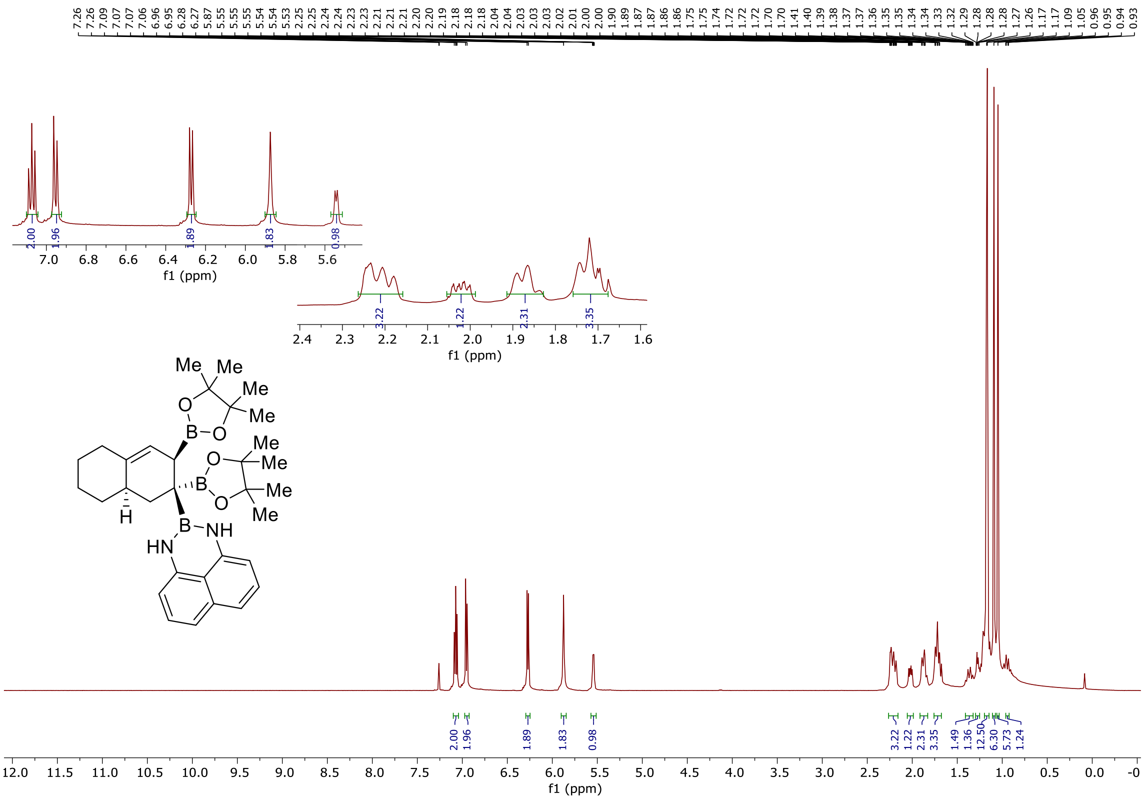

^{13}C NMR (126 MHz, $CDCl_3$) of compound (**5k’**)

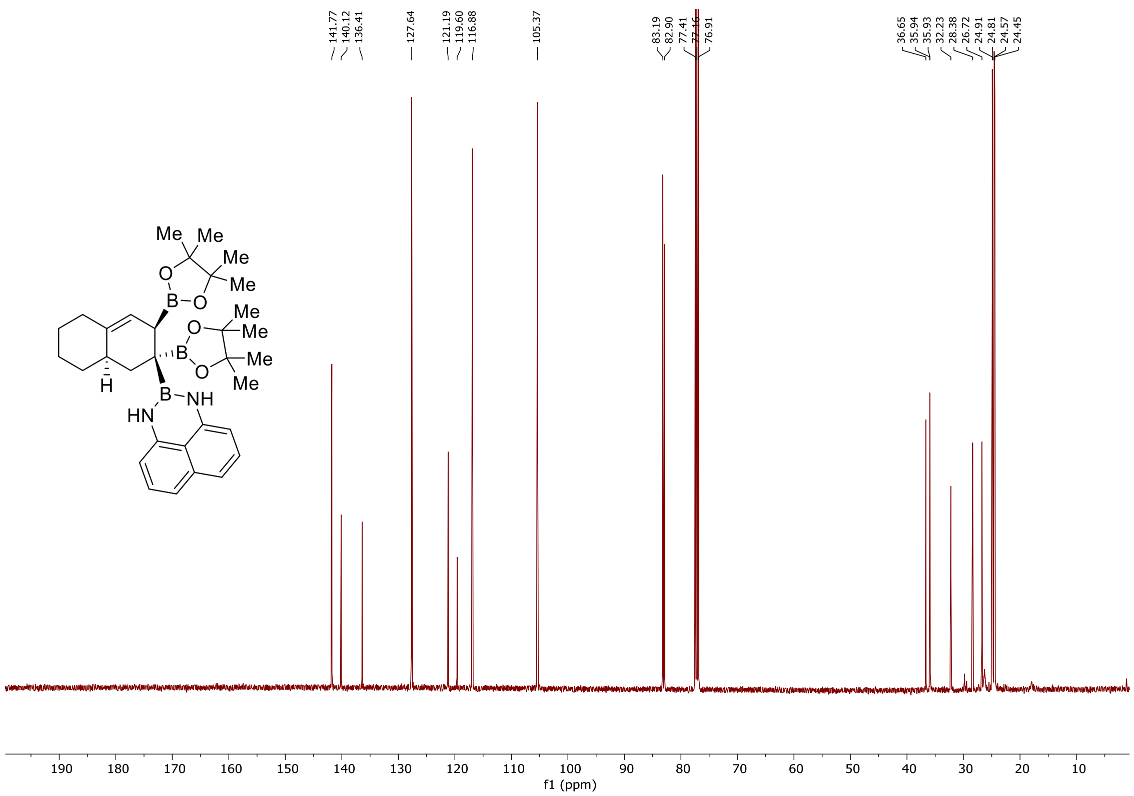

^{11}B NMR (128 MHz, $CDCl_3$) of compound (**5k’**)

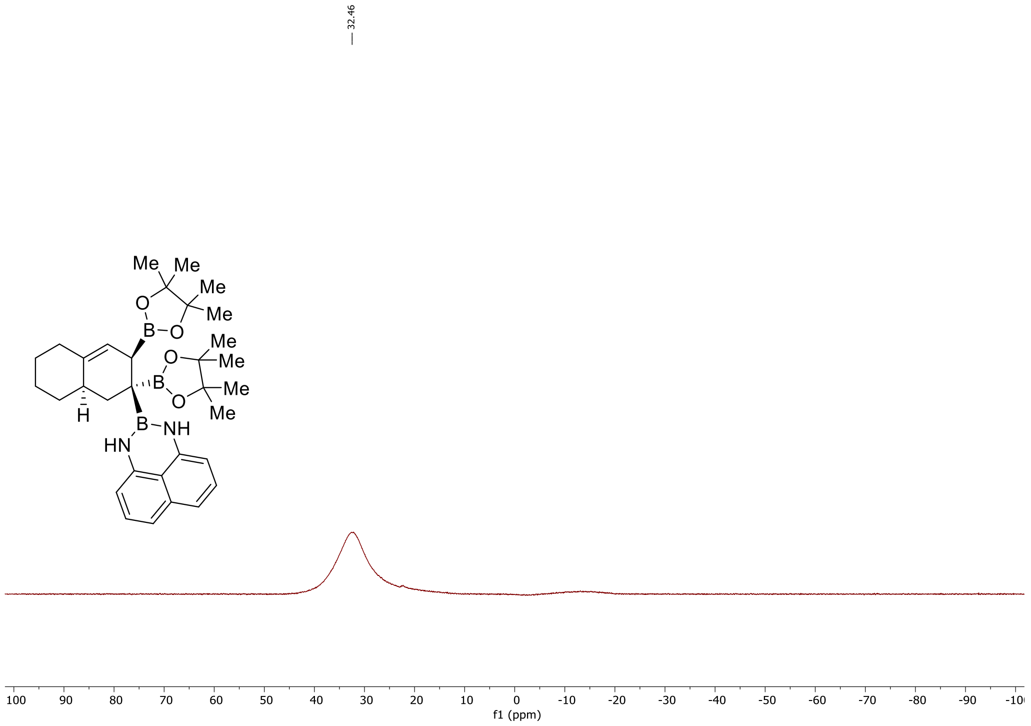

^{1}H NMR (400 MHz, $CDCl_3$) of compound (**4O-d**)

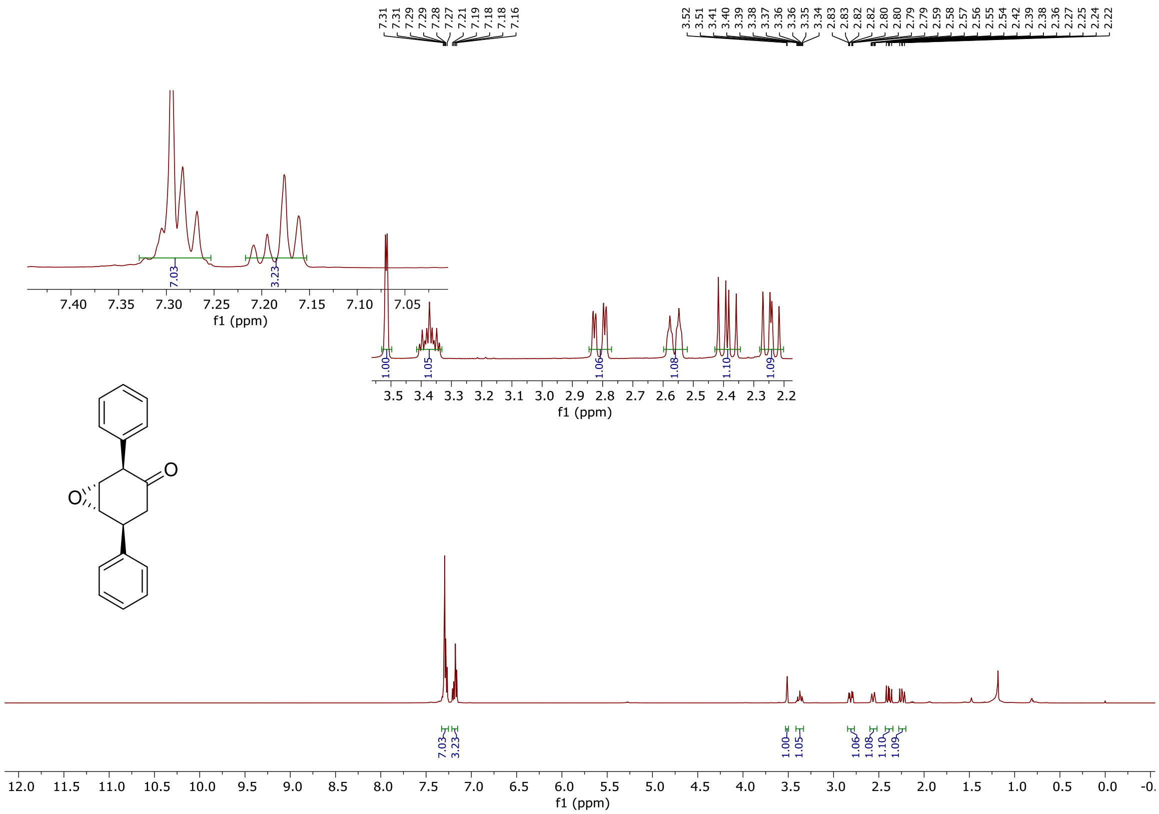

^{13}C NMR (101 MHz, $CDCl_3$) of compound (**4O-d**)

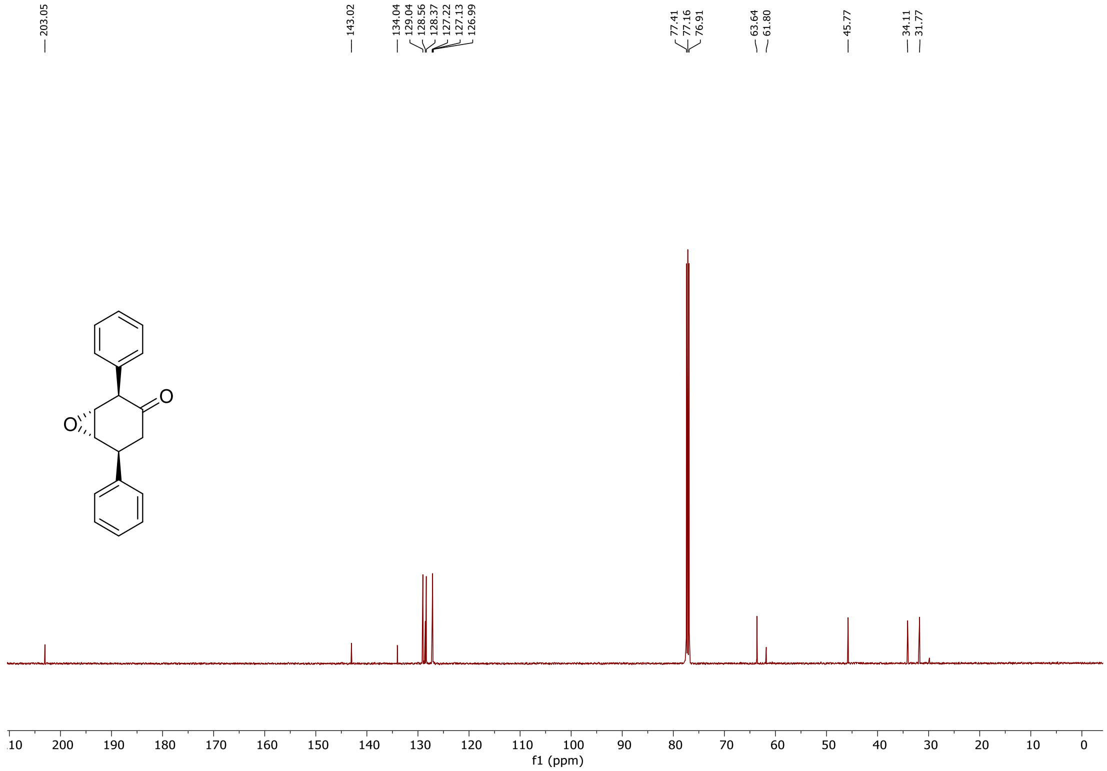

^{1}H NMR (400 MHz, $CDCl_3$) of compound (**4O-d'**)

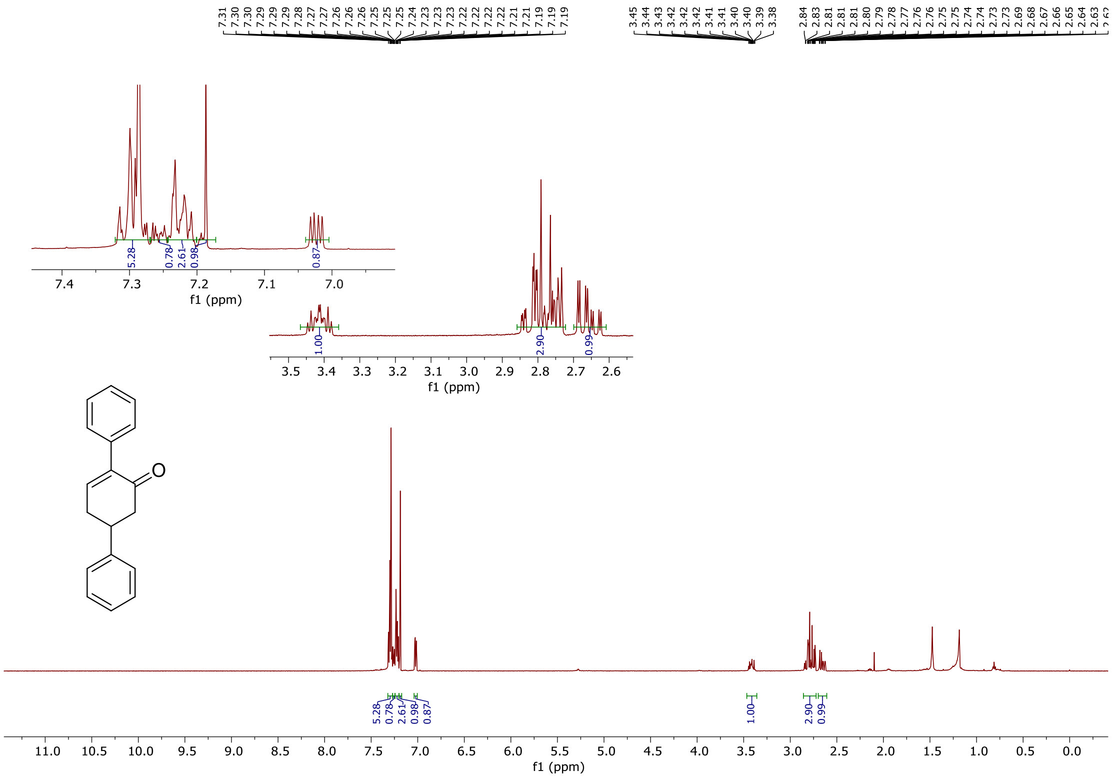

^{13}C NMR (101 MHz, $CDCl_3$) of compound (**4O-d'**)

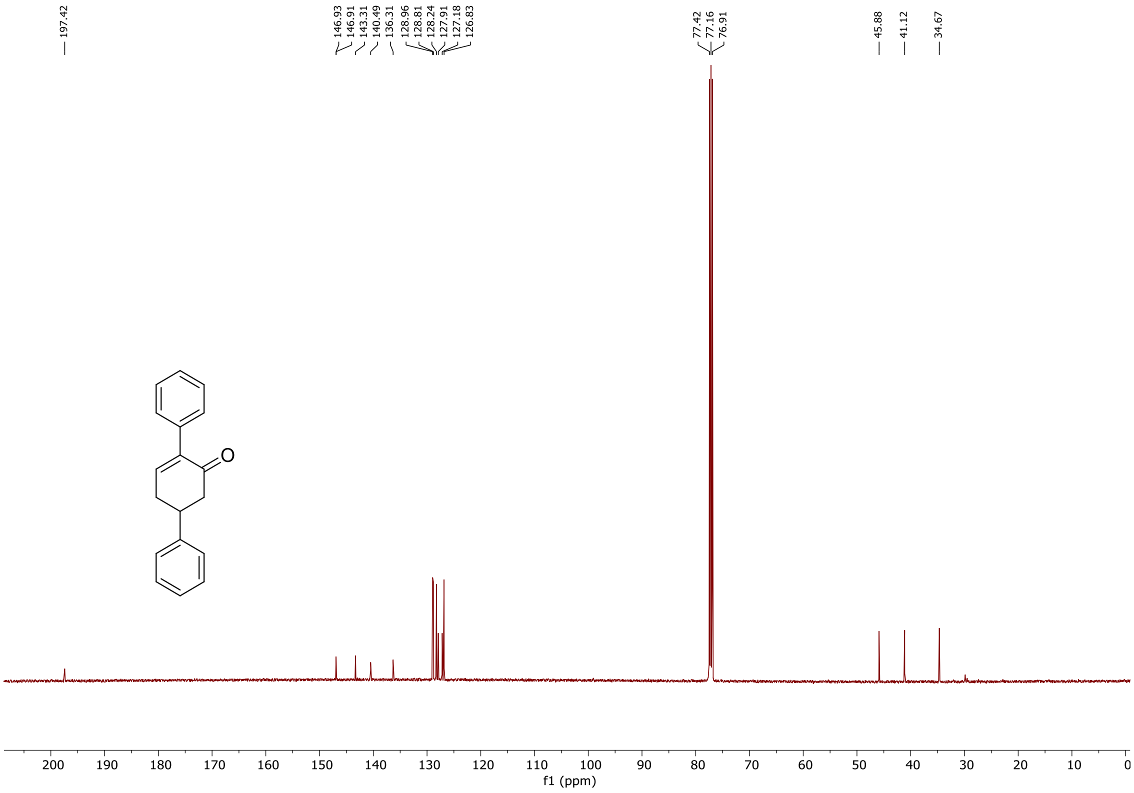

^{1}H NMR (400 MHz, $CDCl_3$) of compound (**4O-c**)

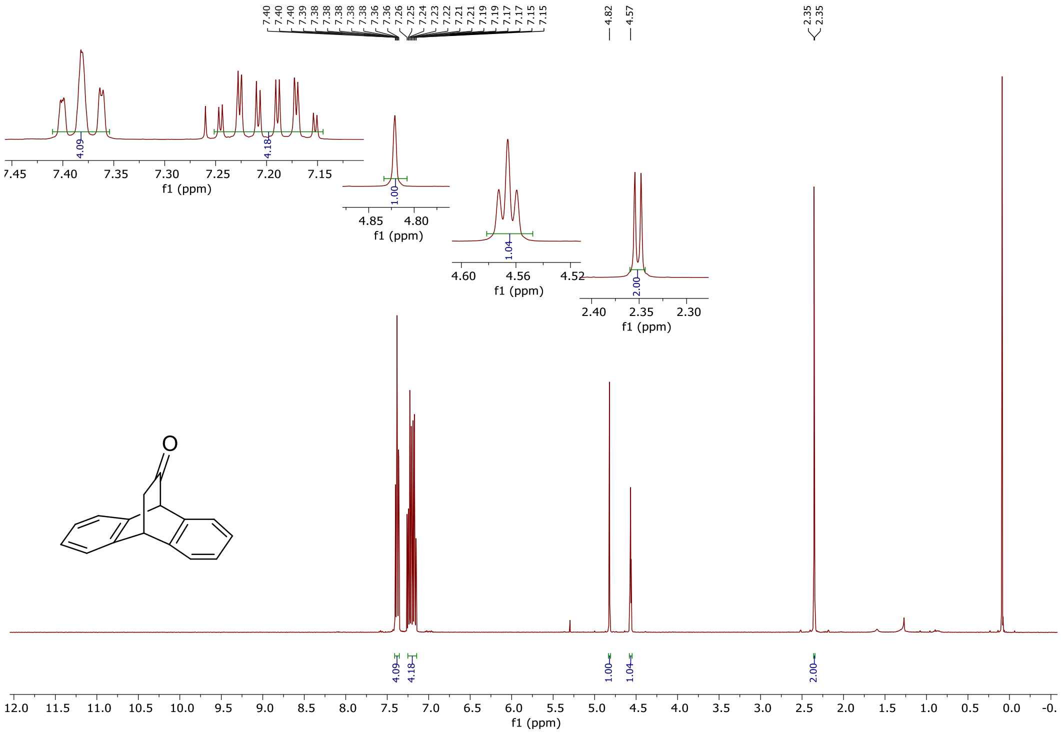

^{13}C NMR (101 MHz, $CDCl_3$) of compound (**4O-c**)

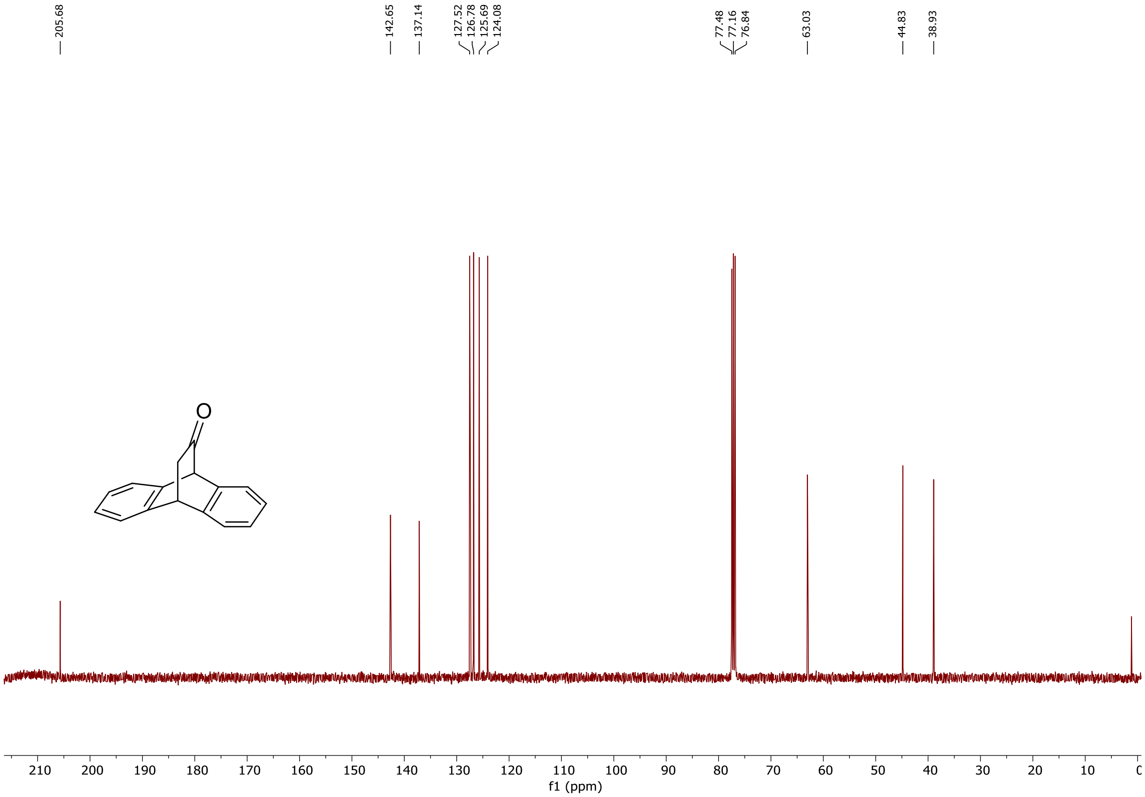

^{1}H NMR (400 MHz, $CDCl_3$) of compound (**4O-f**)

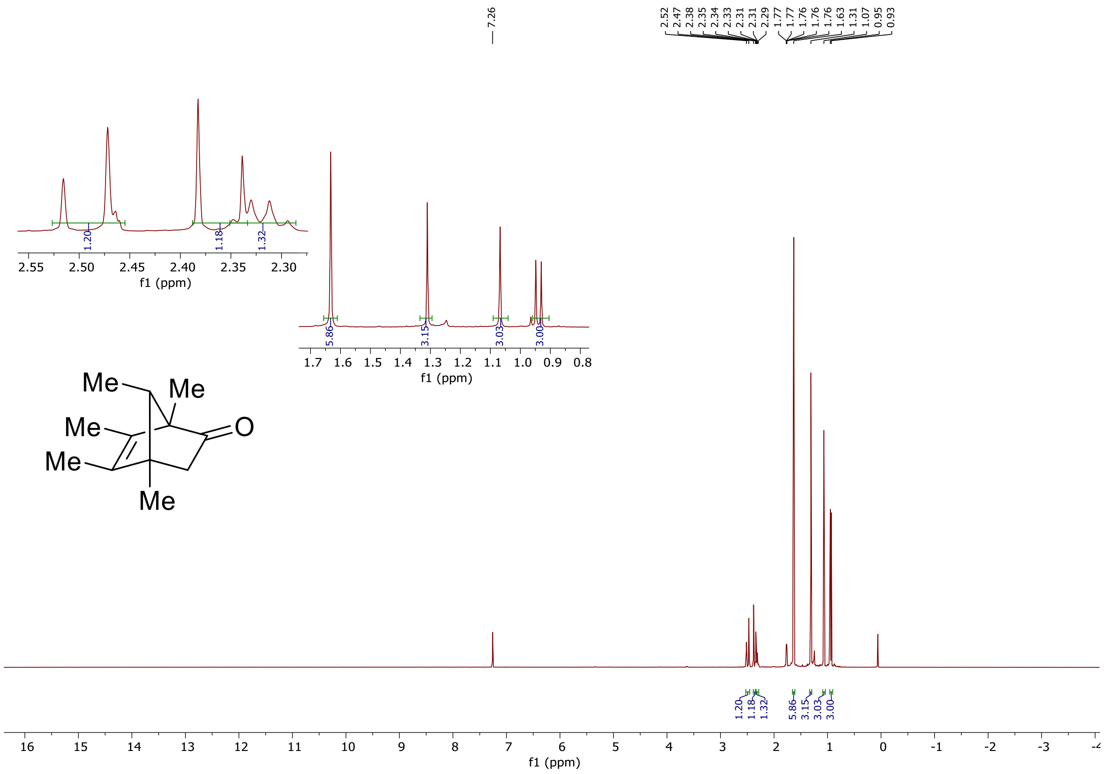

^{13}C NMR (101 MHz, $CDCl_3$) of compound (**4O-f**)

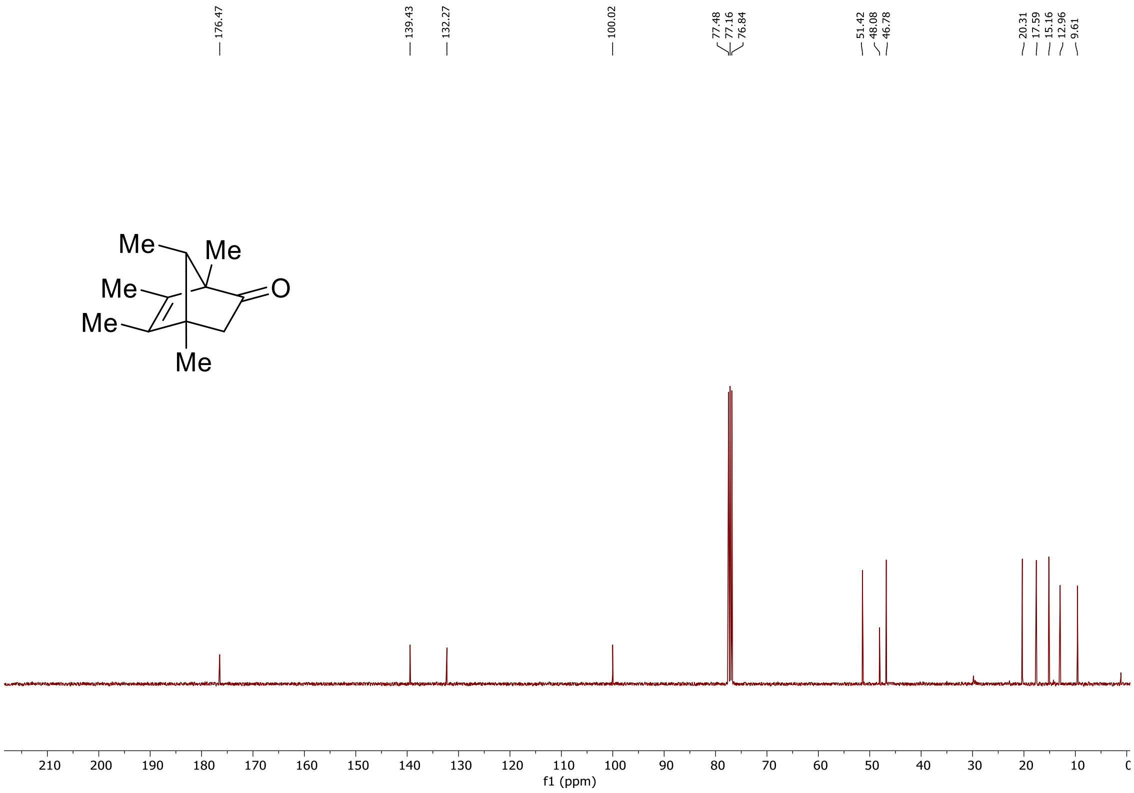

^{1}H NMR (400 MHz, $CDCl_3$) of compound (**4O-g**)

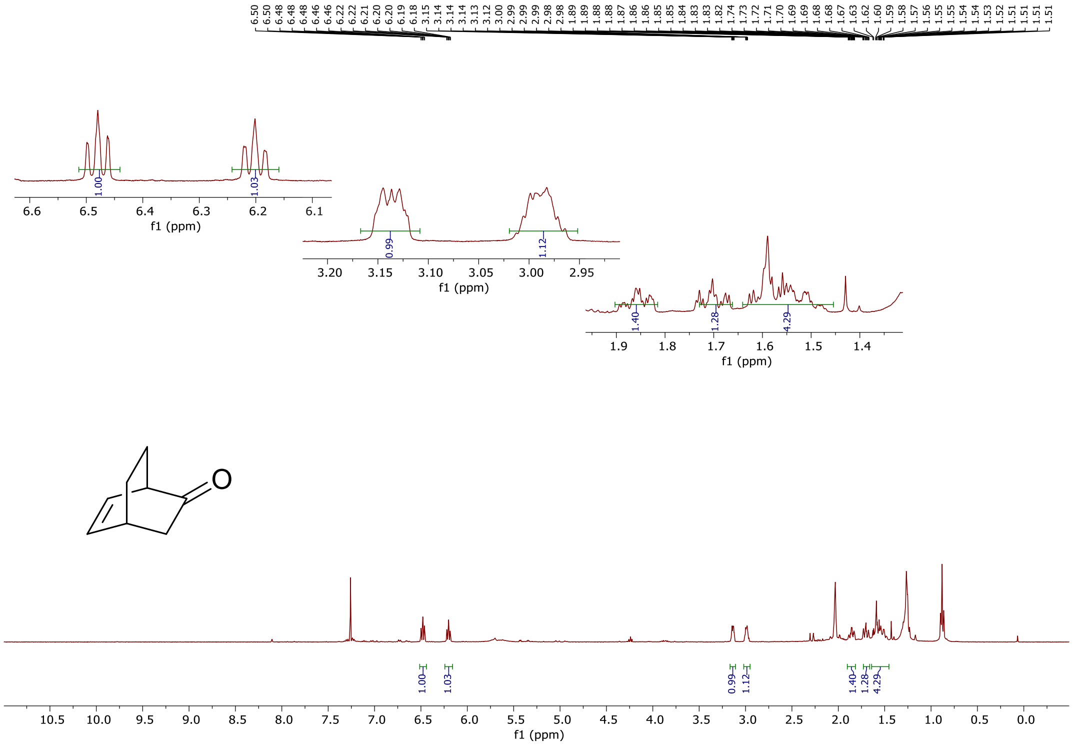

^{1}H NMR (400 MHz, $CDCl_3$) of compound (**4O-k**)

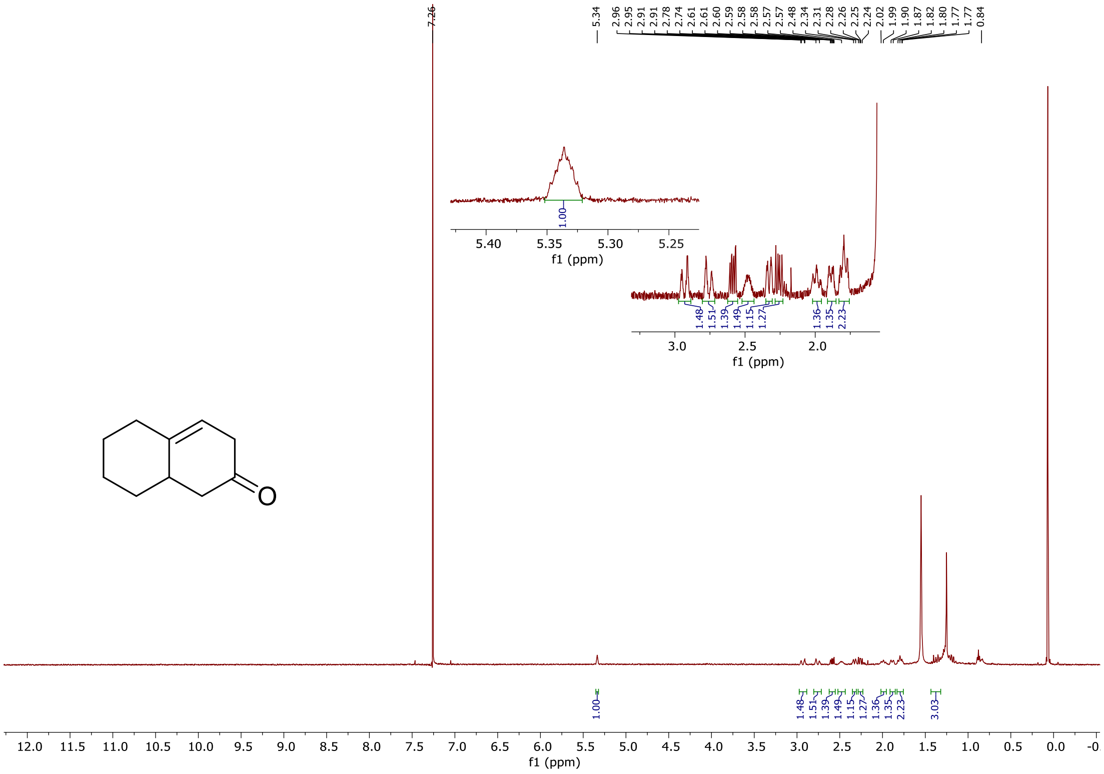

NMR Spectral Graphics: [for Trifluoronation]

^{1}H NMR (400 MHz, DMSO-d_6) of compound (**6a**)

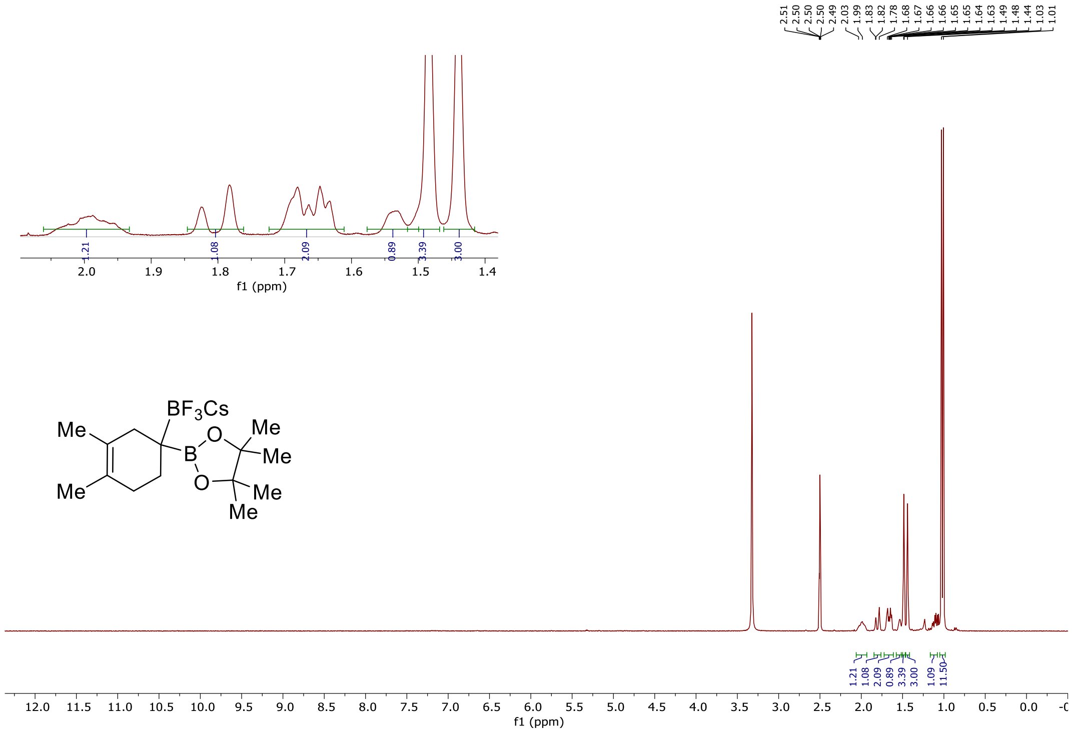

^{13}C NMR (101 MHz, DMSO-d_6) of compound (**6a**)

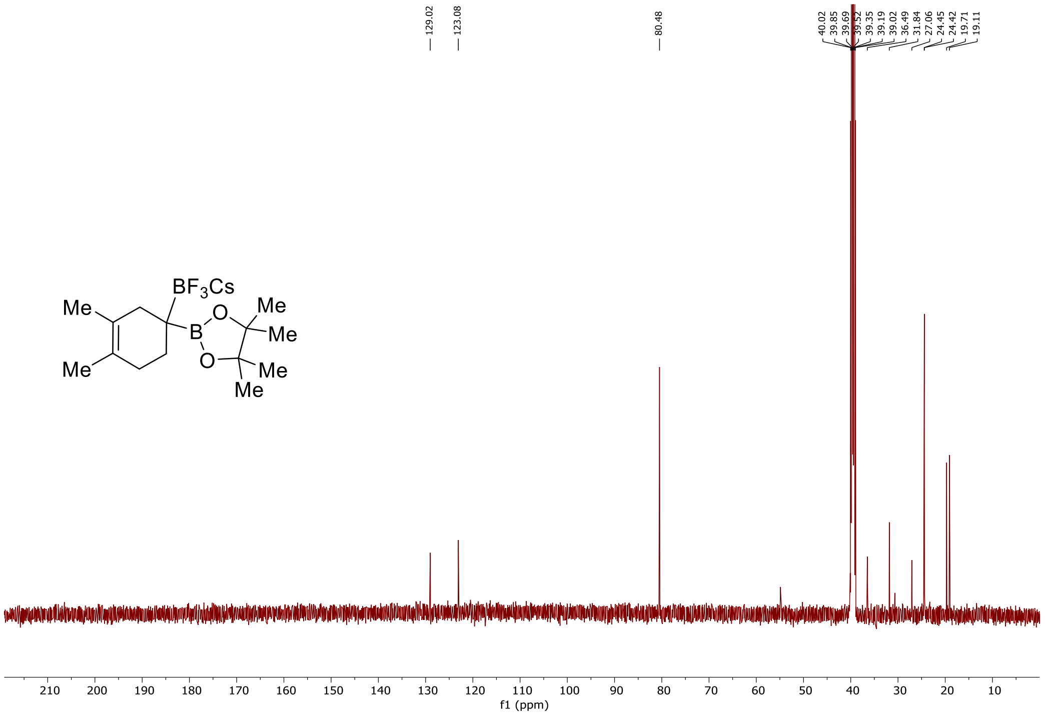

^{11}B NMR (128 MHz, DMSO-d_6) of compound (**6a**)

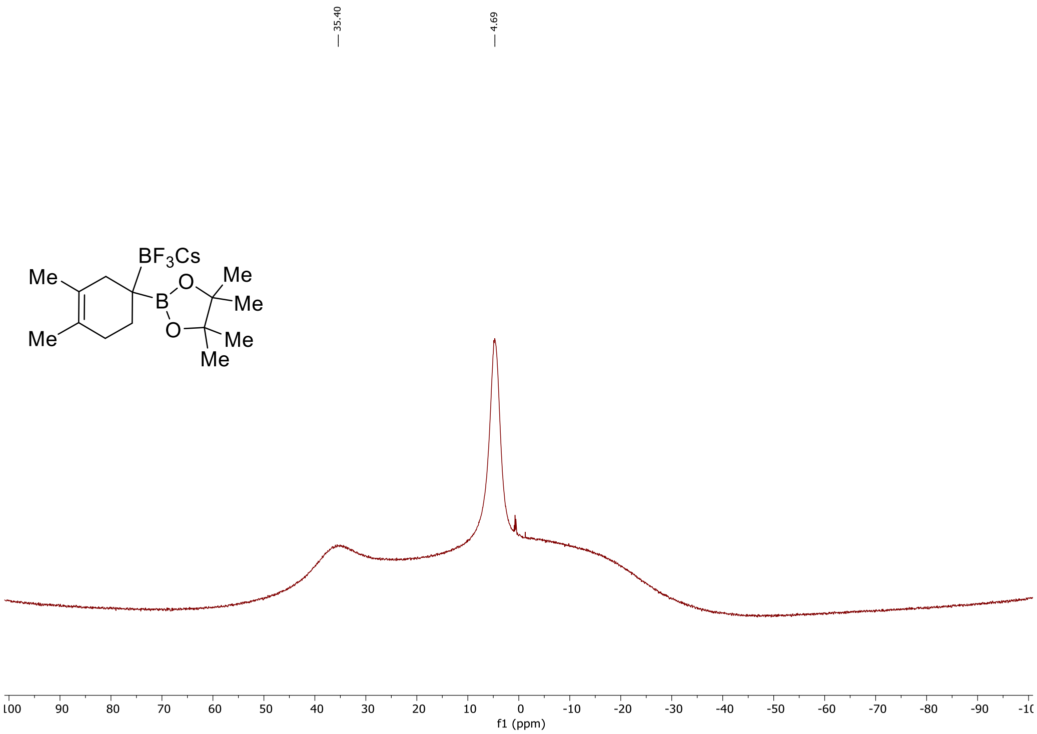

^{19}F NMR (376 MHz, DMSO-d_6) of compound (**6a**)

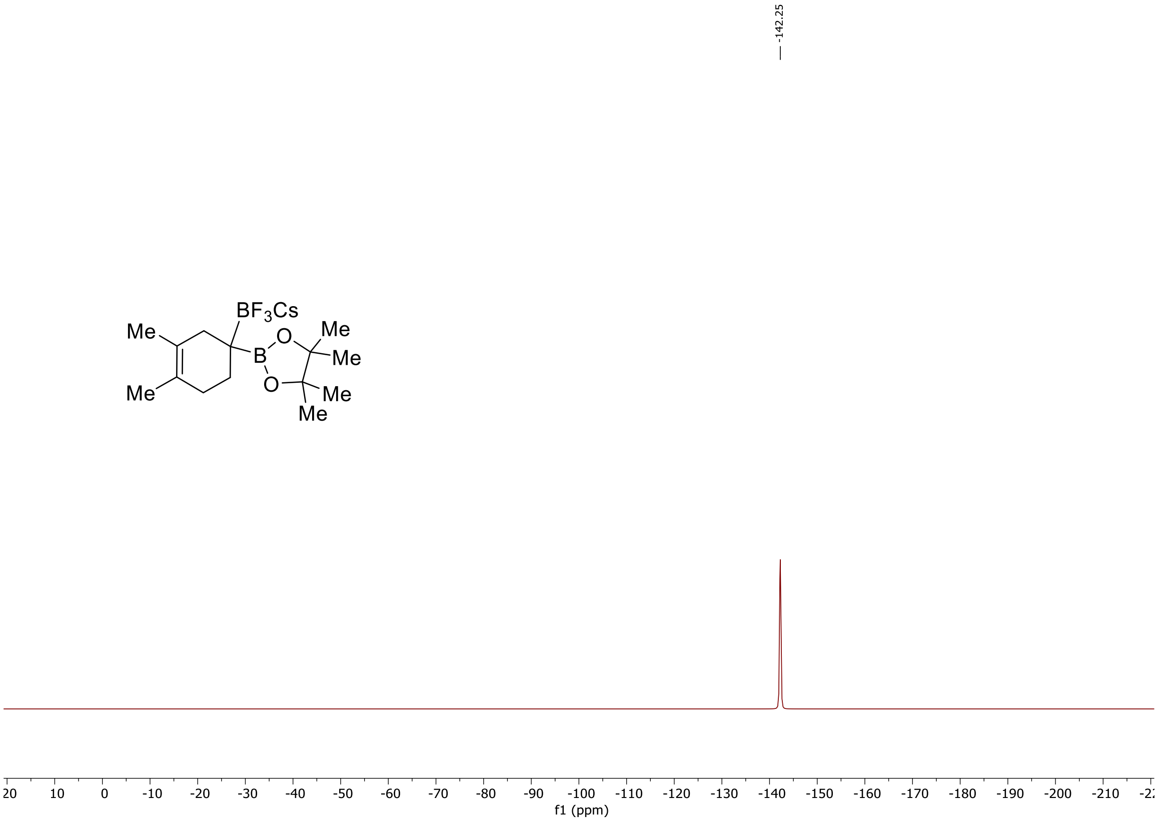

^{1}H NMR (400 MHz, DMSO-d_6) of compound (**6b**)

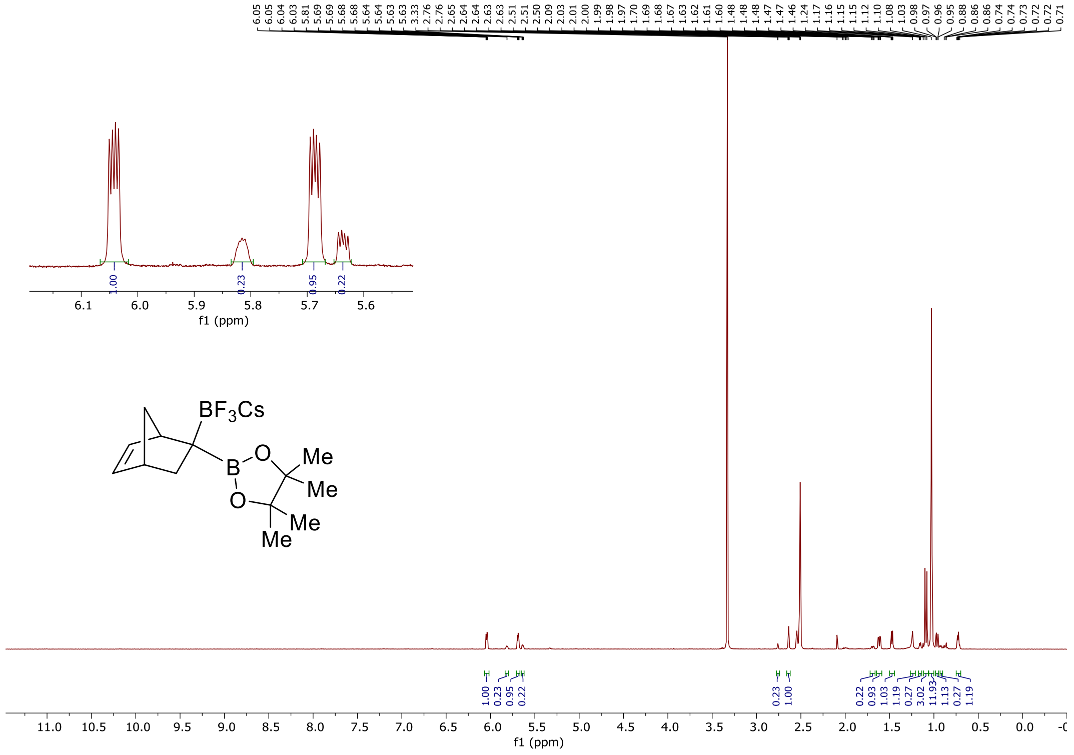

^{13}C NMR (101 MHz, DMSO-d_6) of compound (**6b**)

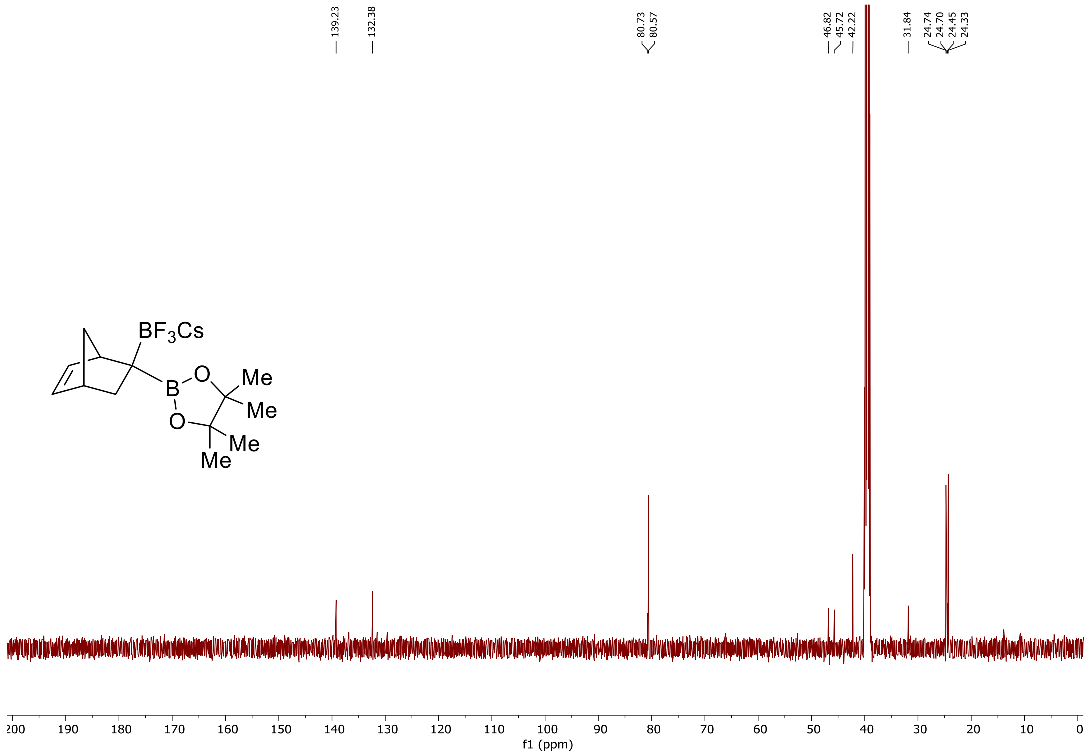

^{11}B NMR (128 MHz, DMSO-d_6) of compound (**6b**)

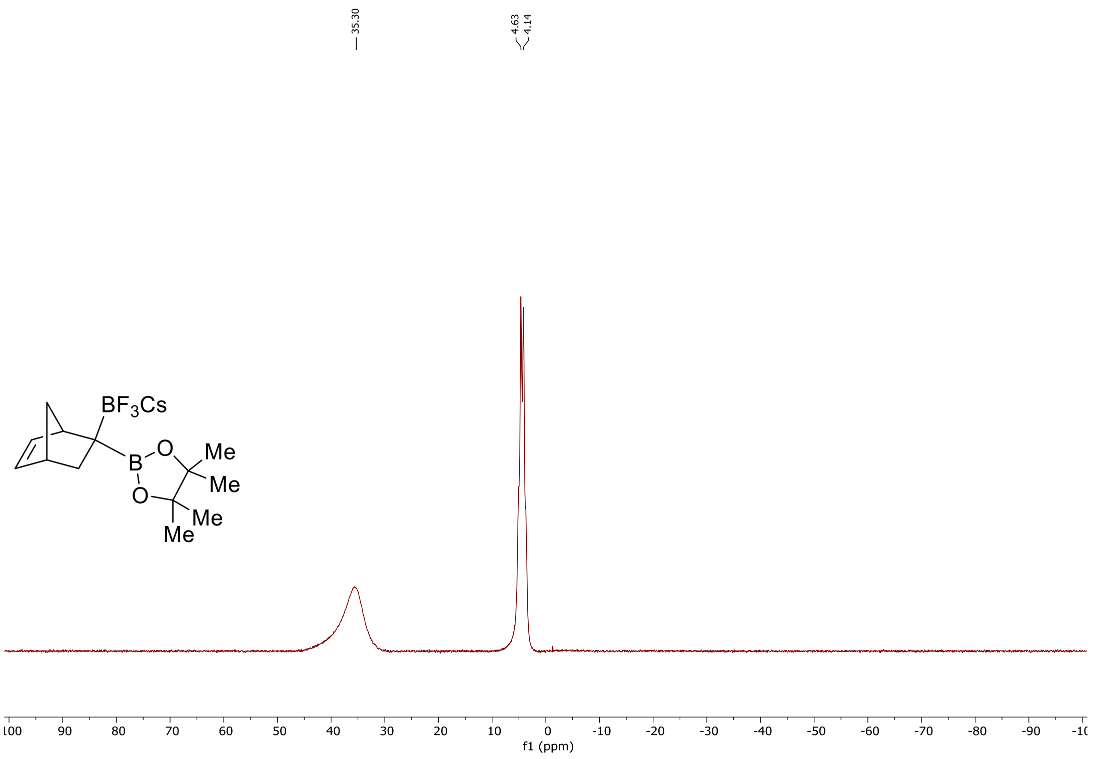

^{19}F NMR (376 MHz, DMSO-d_6) of compound (**6b**)

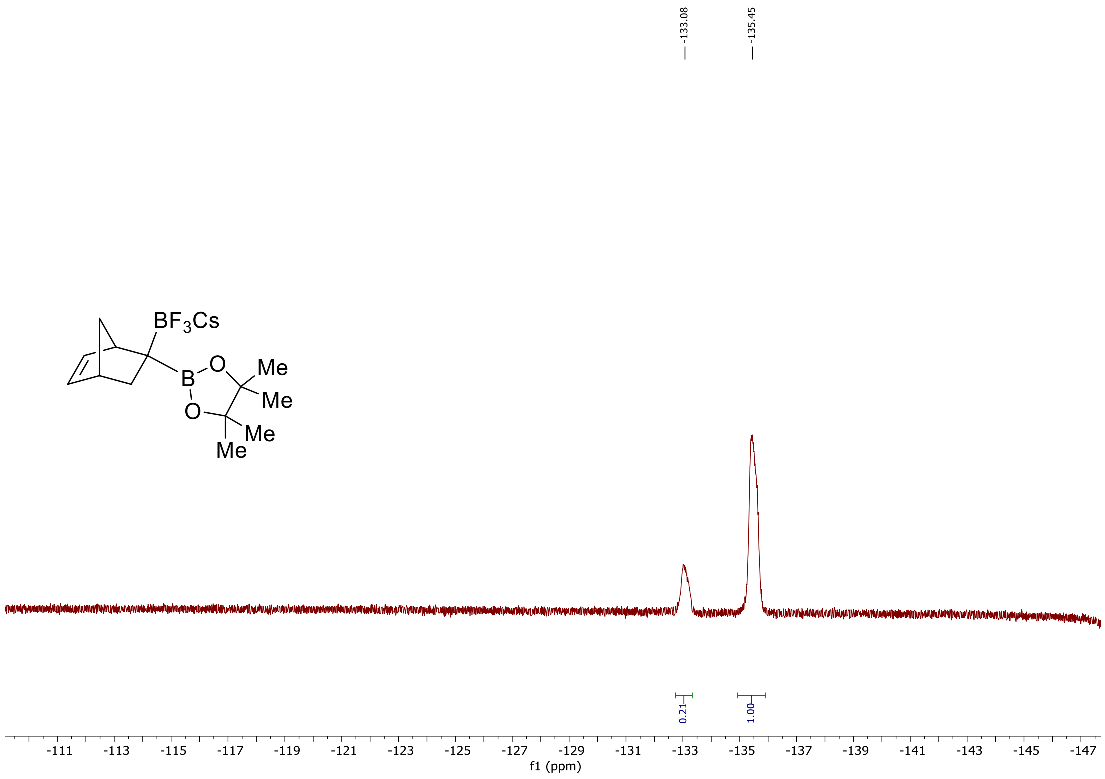

^{1}H NMR (400 MHz, DMSO-d_6) of compound (**6c**)

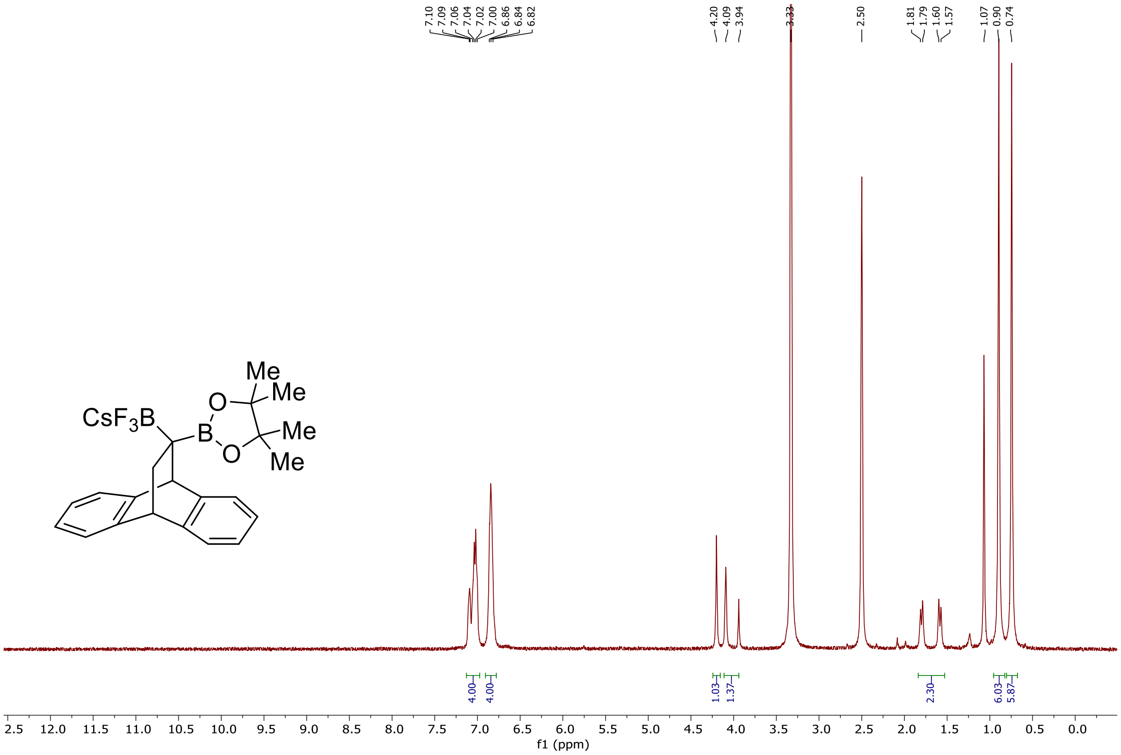

^{13}C NMR (101 MHz, DMSO-d_6) of compound (**6c**)

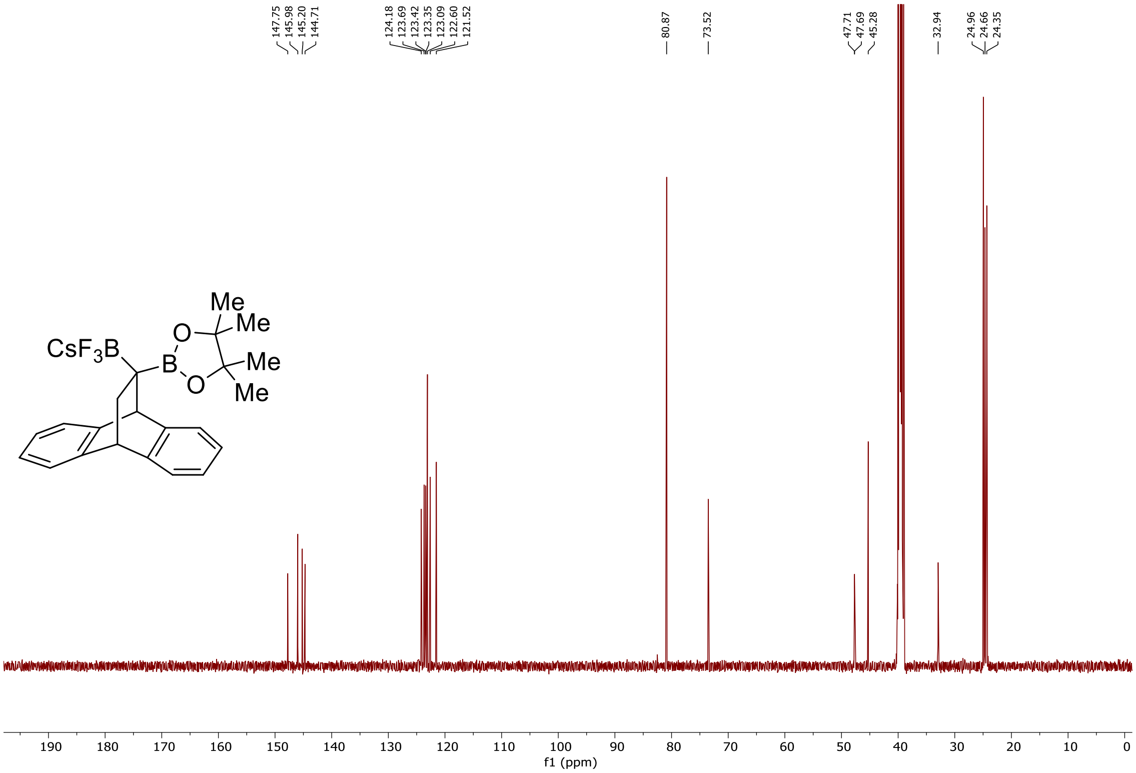

^{11}B NMR (128 MHz, DMSO-d_6) of compound (**6c**)

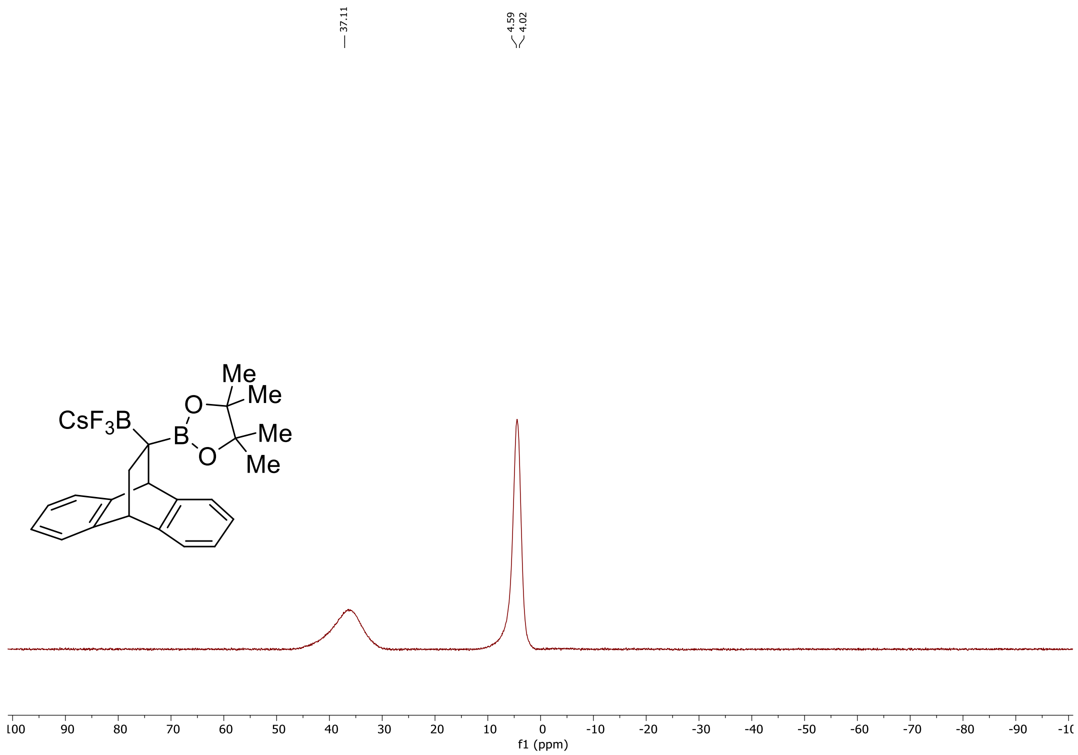

^{19}F NMR (376 MHz, DMSO-d_6) of compound (**6c**)

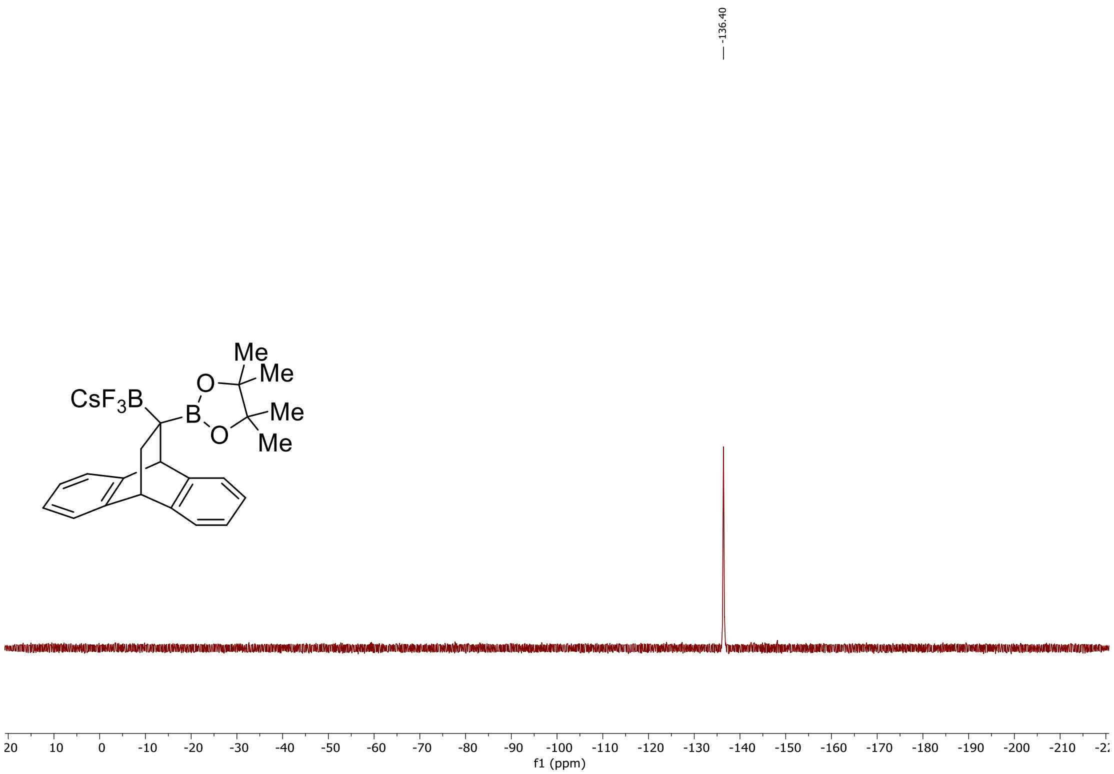

^{1}H NMR (400 MHz, DMSO-d_6) of compound (**6d**)

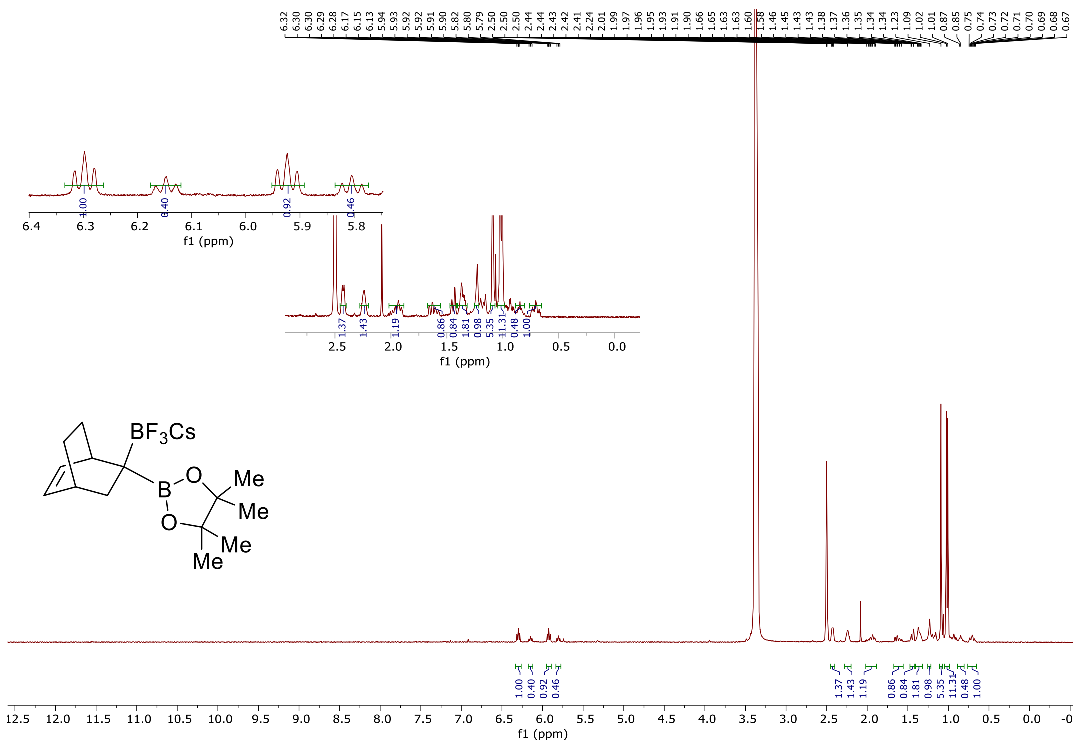

^{13}C NMR (101 MHz, DMSO-d_6) of compound (**6d**)

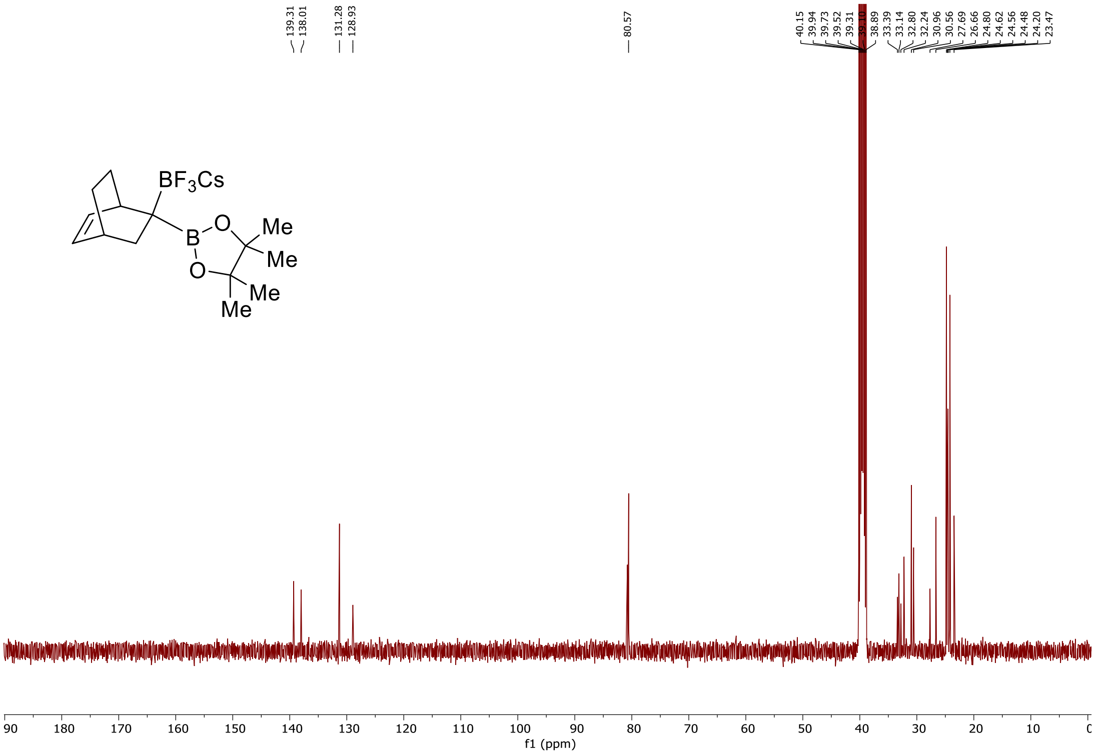

^{11}B NMR (128 MHz, DMSO-d_6) of compound (**6d**)

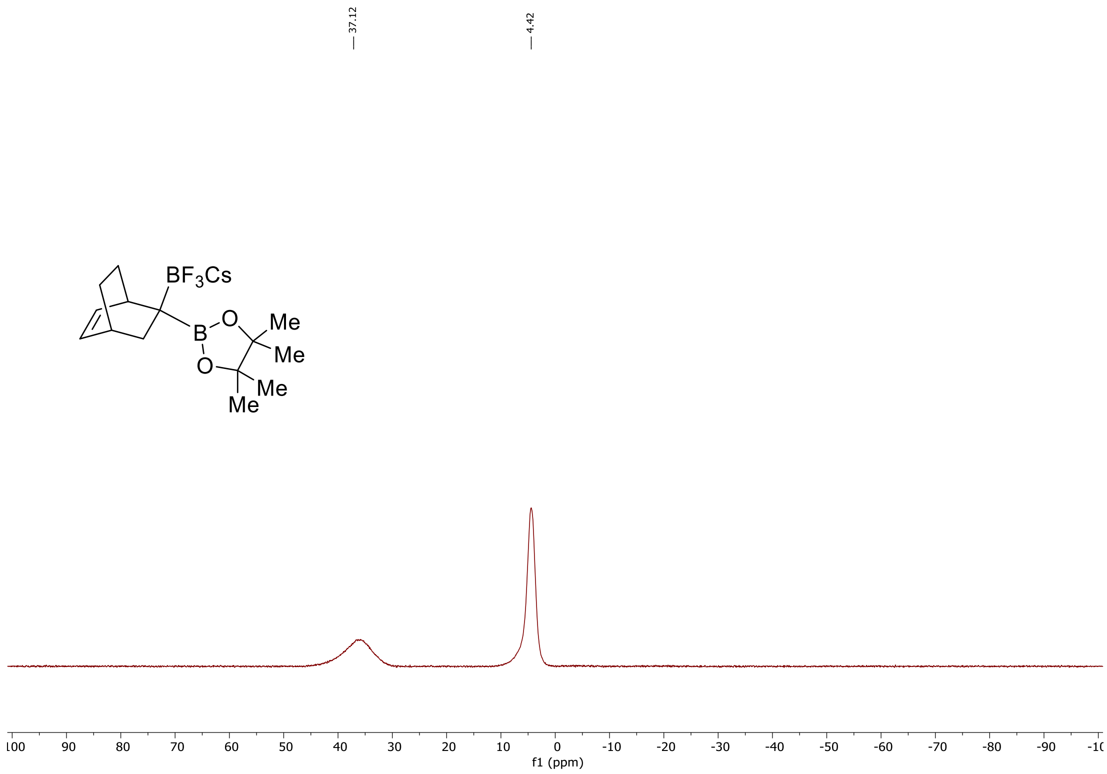

^{19}F NMR (376 MHz, DMSO-d_6) of compound (**6d**)

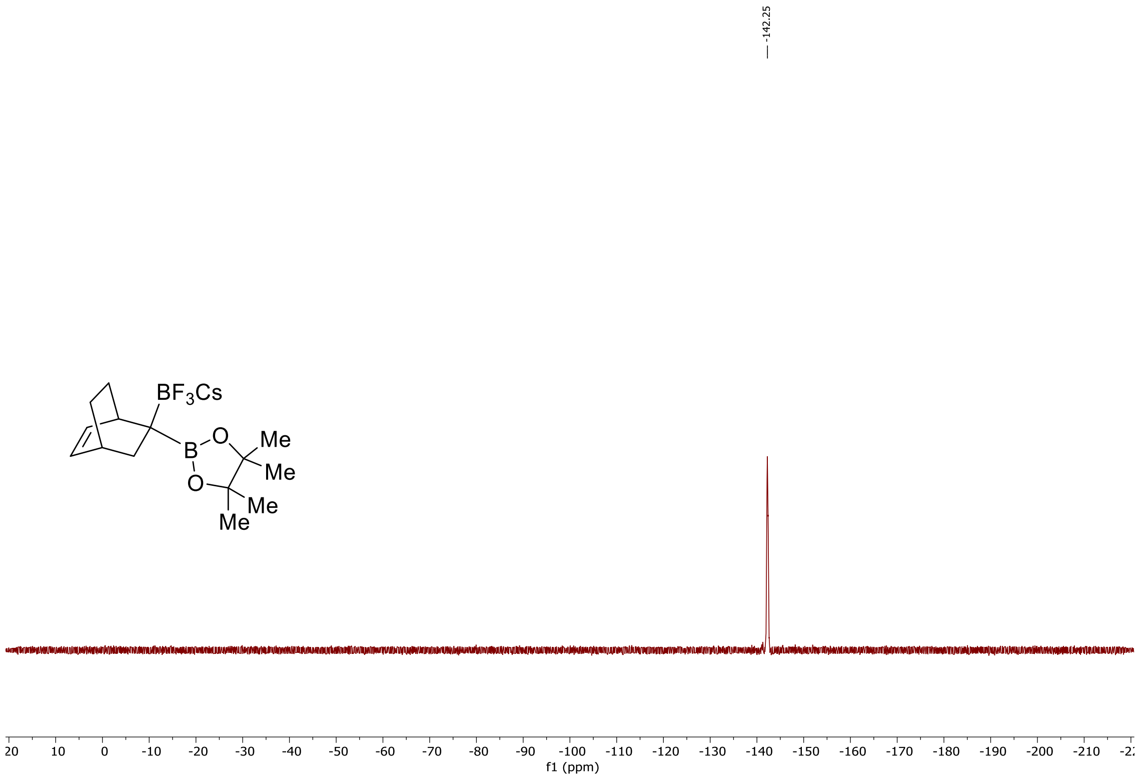

^{1}H NMR (400 MHz, DMSO-d_6) of compound (**6f**)

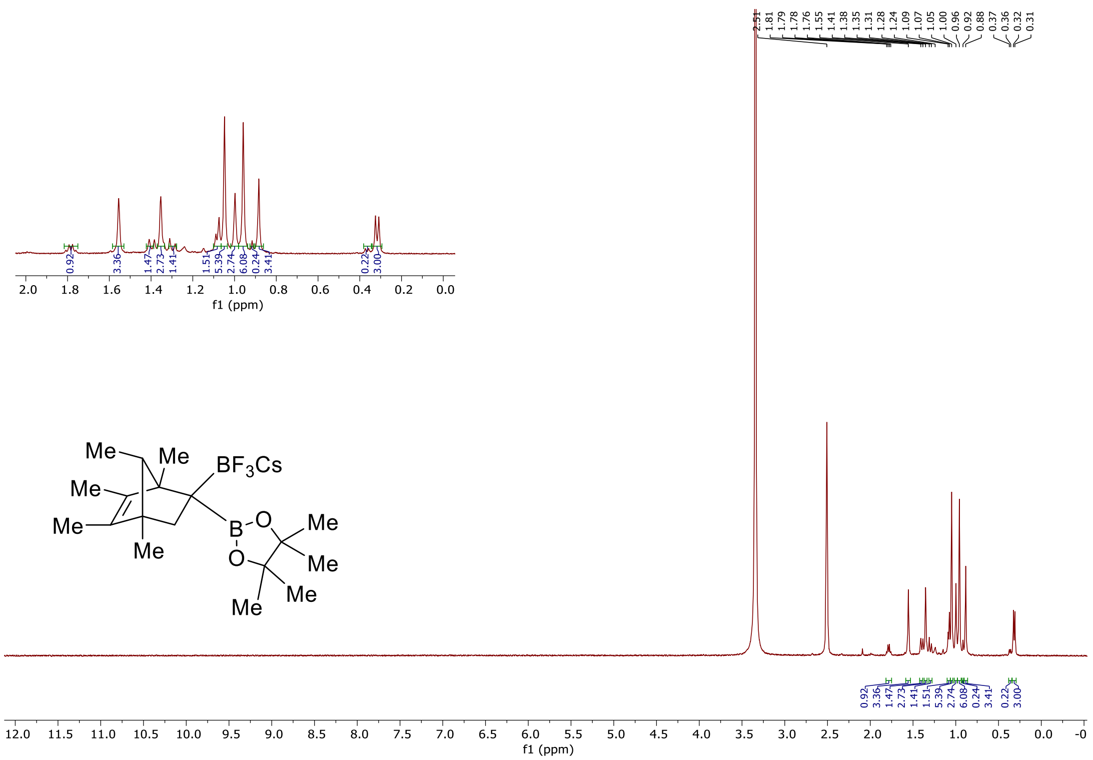

^{13}C NMR (101 MHz, DMSO-d_6) of compound (**6f**)

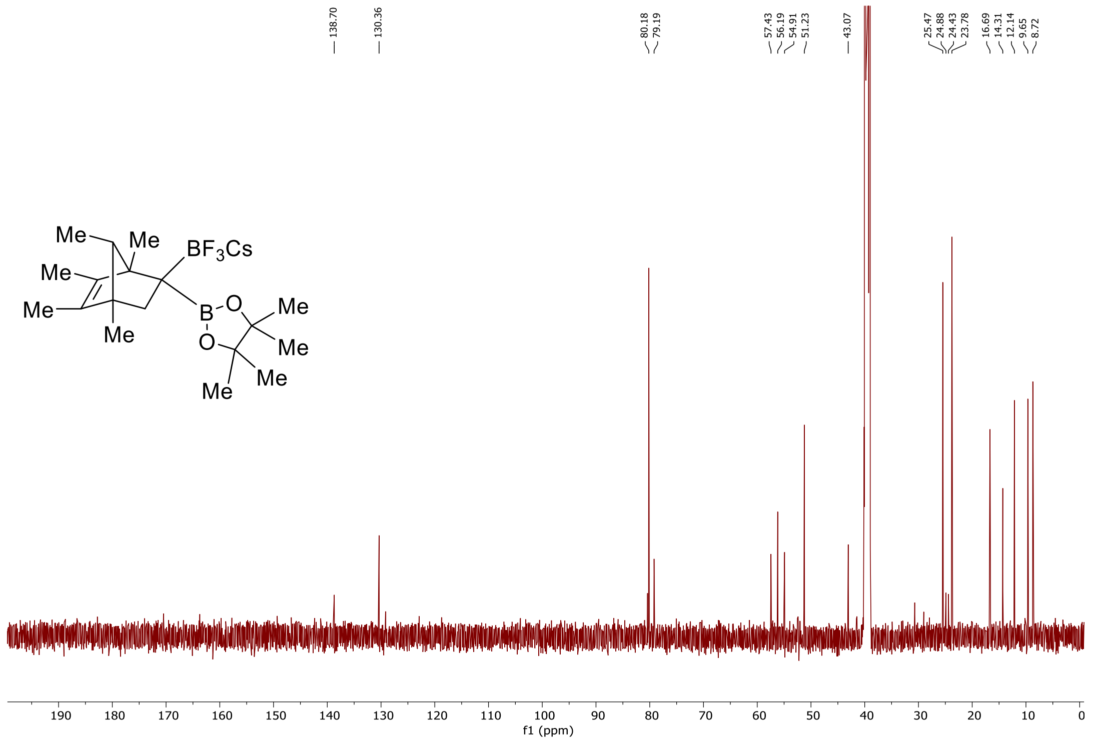

^{11}B NMR (128 MHz, DMSO-d_6) of compound (**6f**)

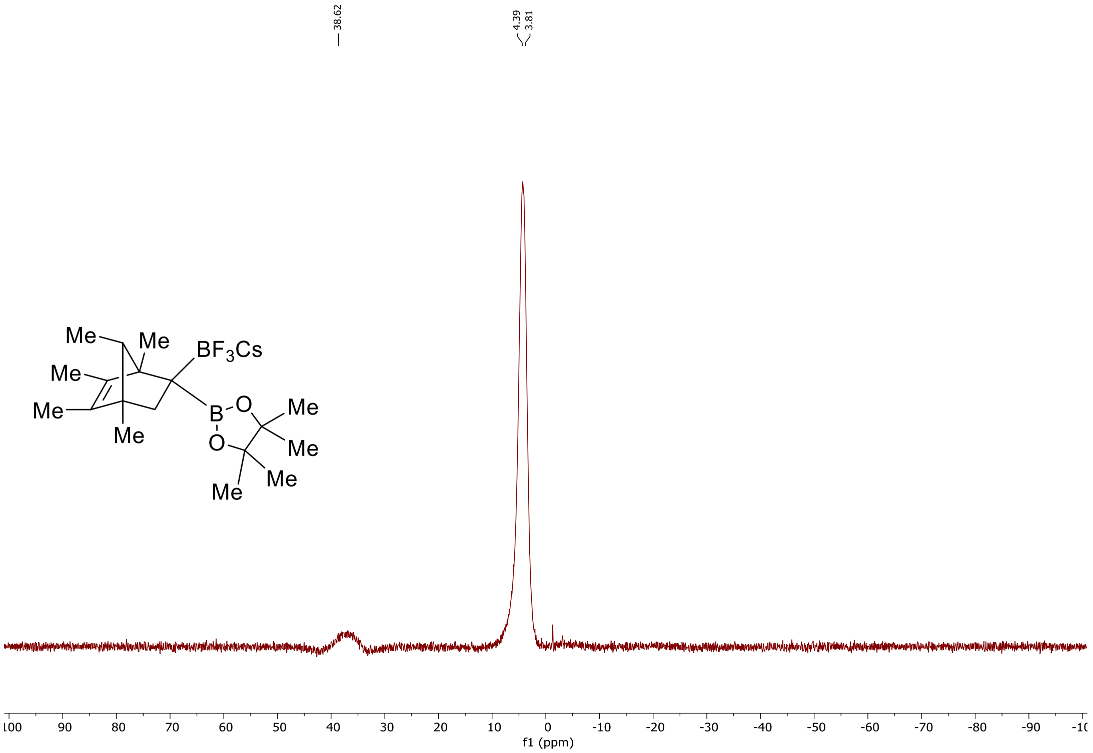

^{19}F NMR (376 MHz, DMSO-d_6) of compound (**6f**)

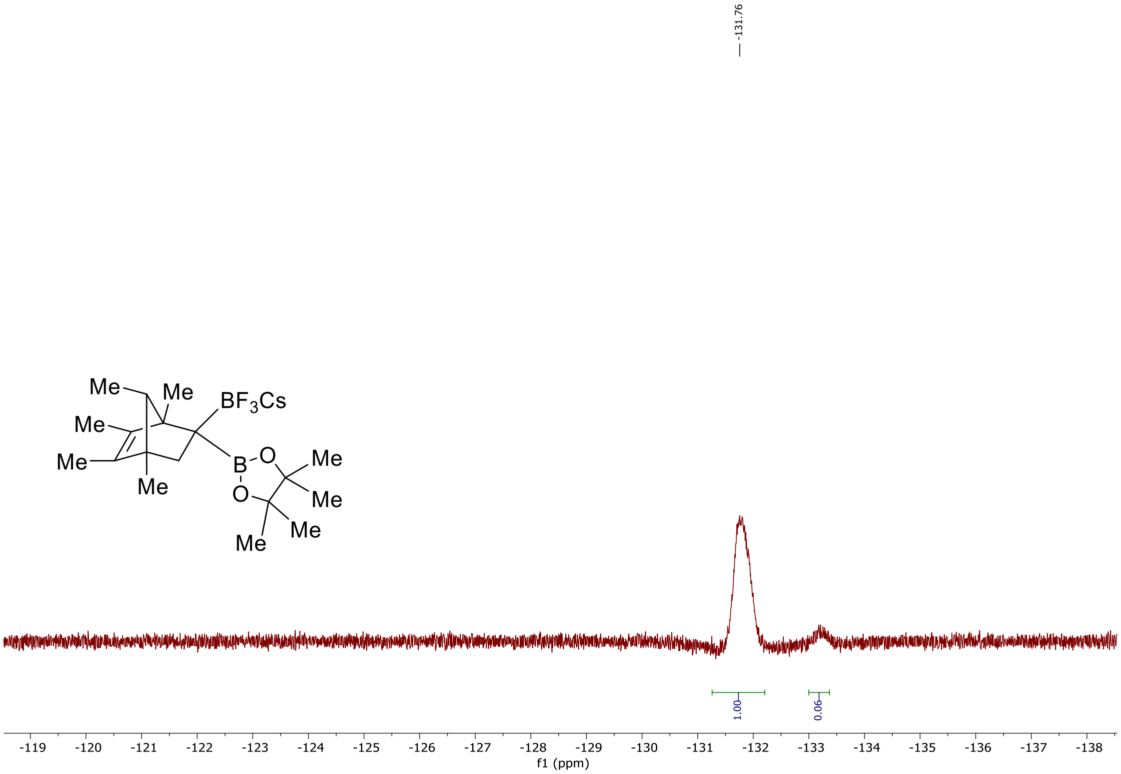

^{1}H NMR (500 MHz, $CDCl_3$) of compound (**5f-*exo***)

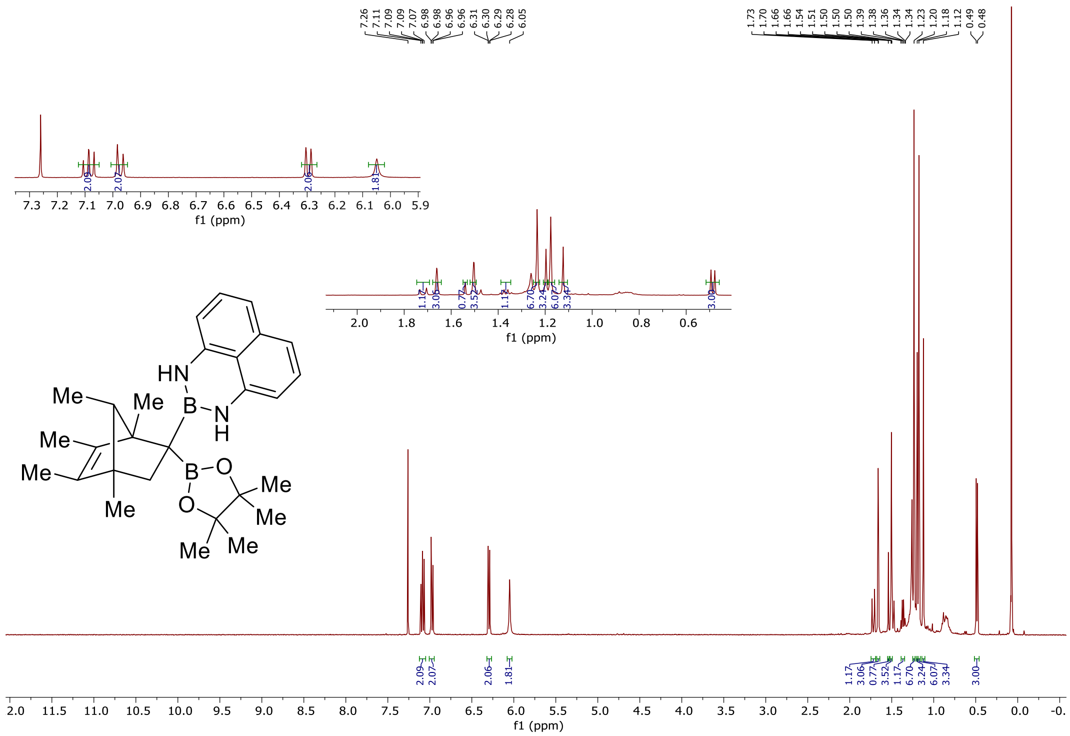

^{13}C NMR (126 MHz, $CDCl_3$) of compound (**5f-*exo***)

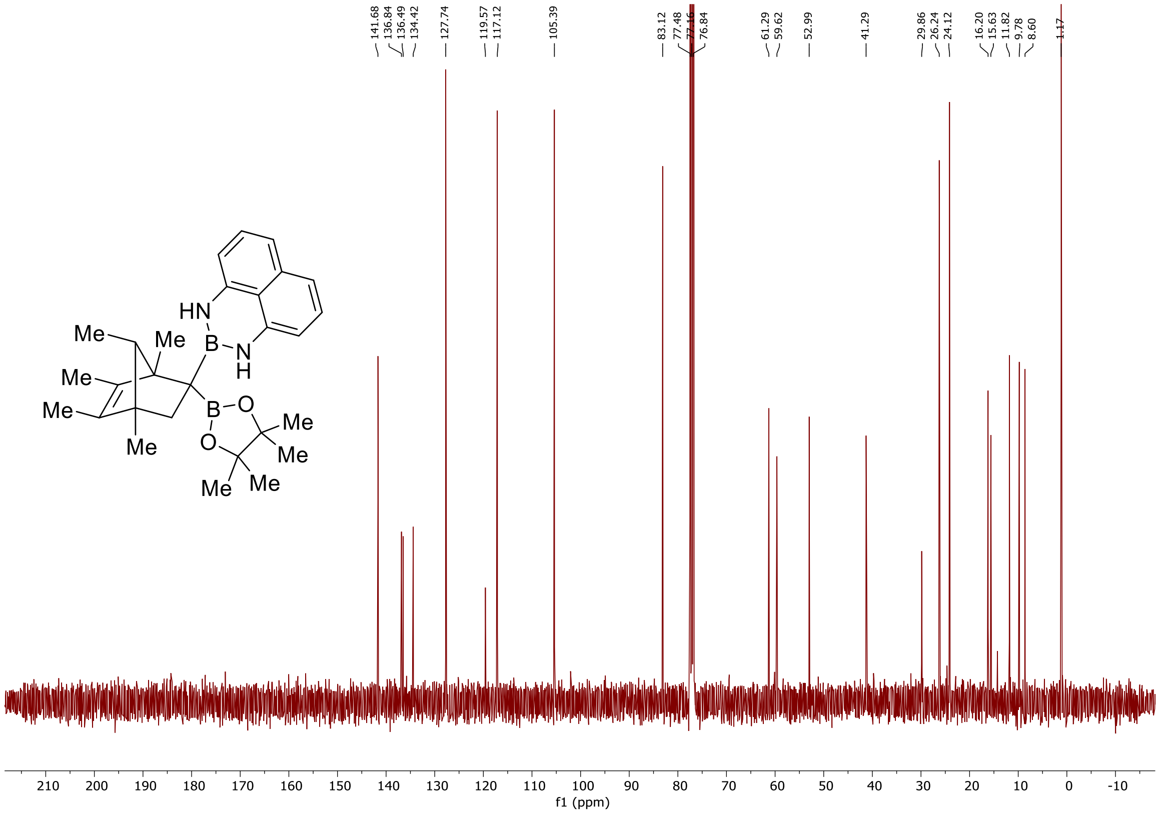

^{11}B NMR (128 MHz, $CDCl_3$) of compound (**5f-*exo***)

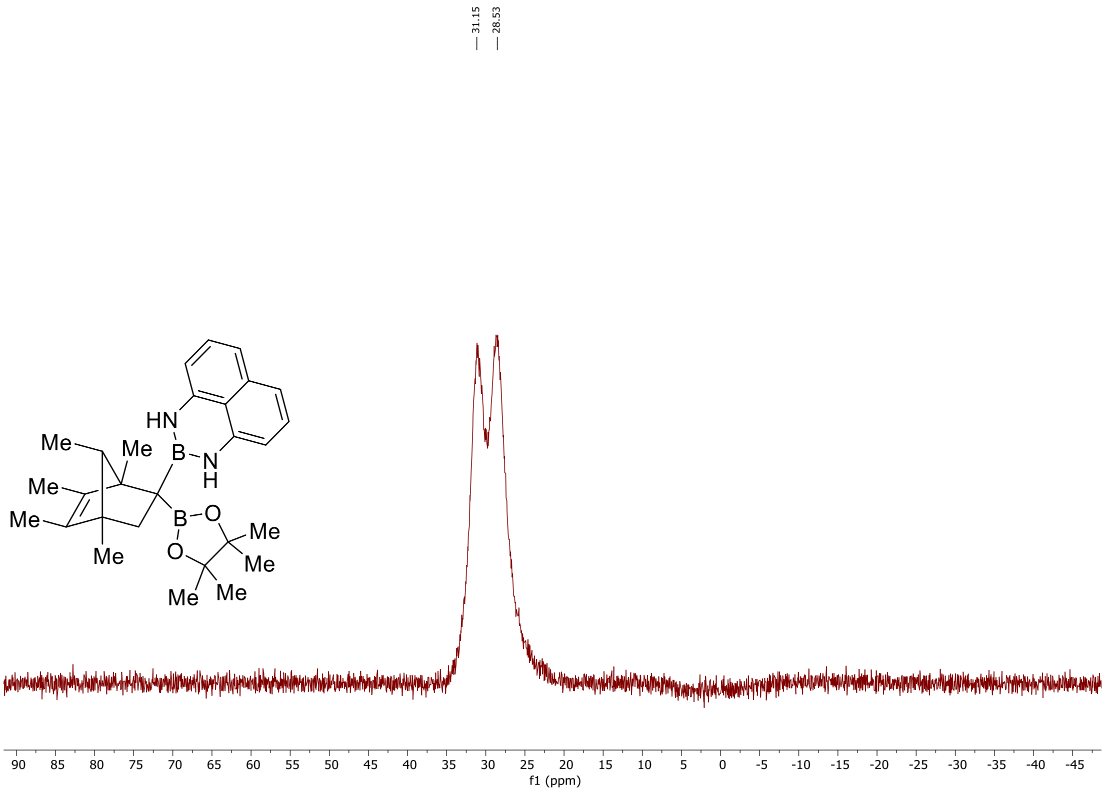

NMR Spectral Graphics: [for Polymers]

^{1}H NMR (400 MHz, $CDCl_3$) of compound (**poly-7-BpinBpin**)

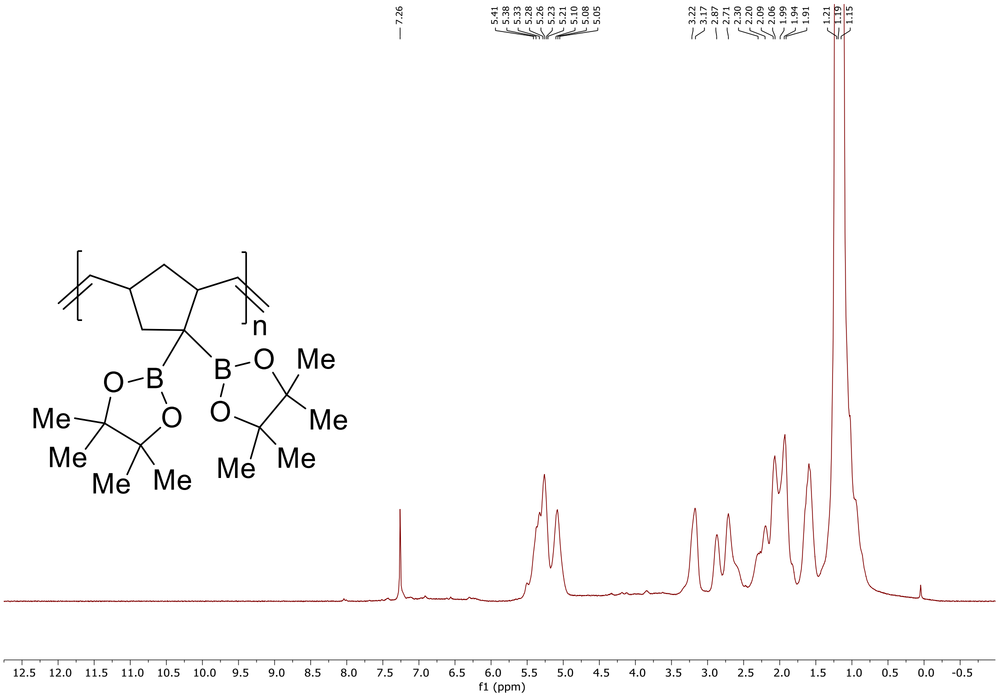

^{13}C NMR (126 MHz, $CDCl_3$) of compound (**poly-7-BpinBpin**)

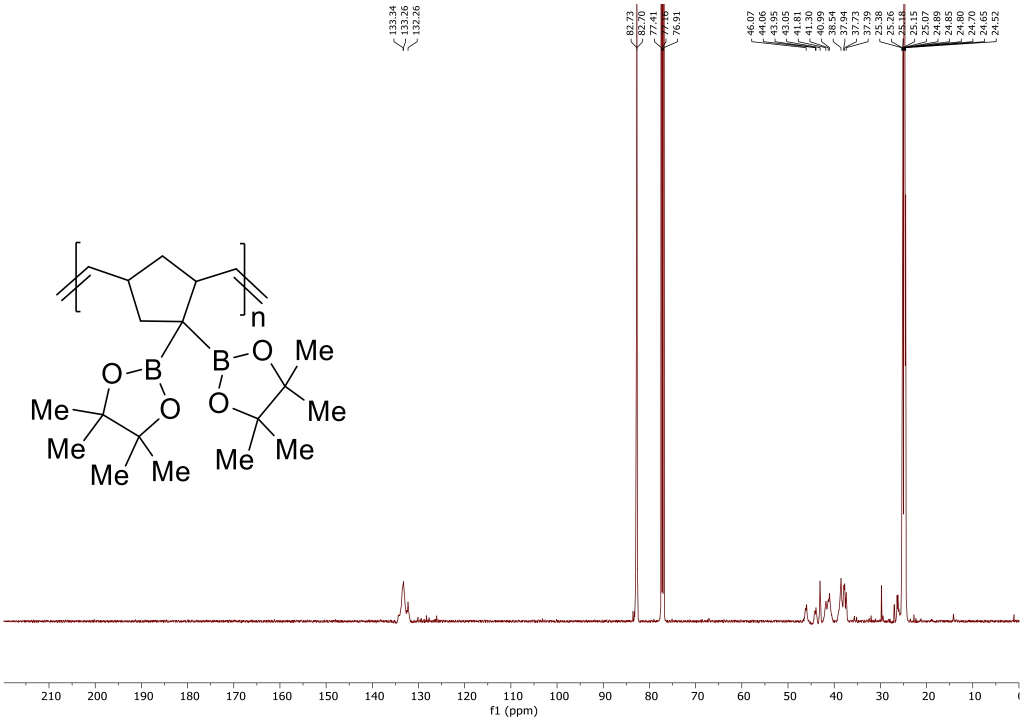

^{11}B NMR (128 MHz, $CDCl_3$) of compound (**poly-7-BpinBpin**)

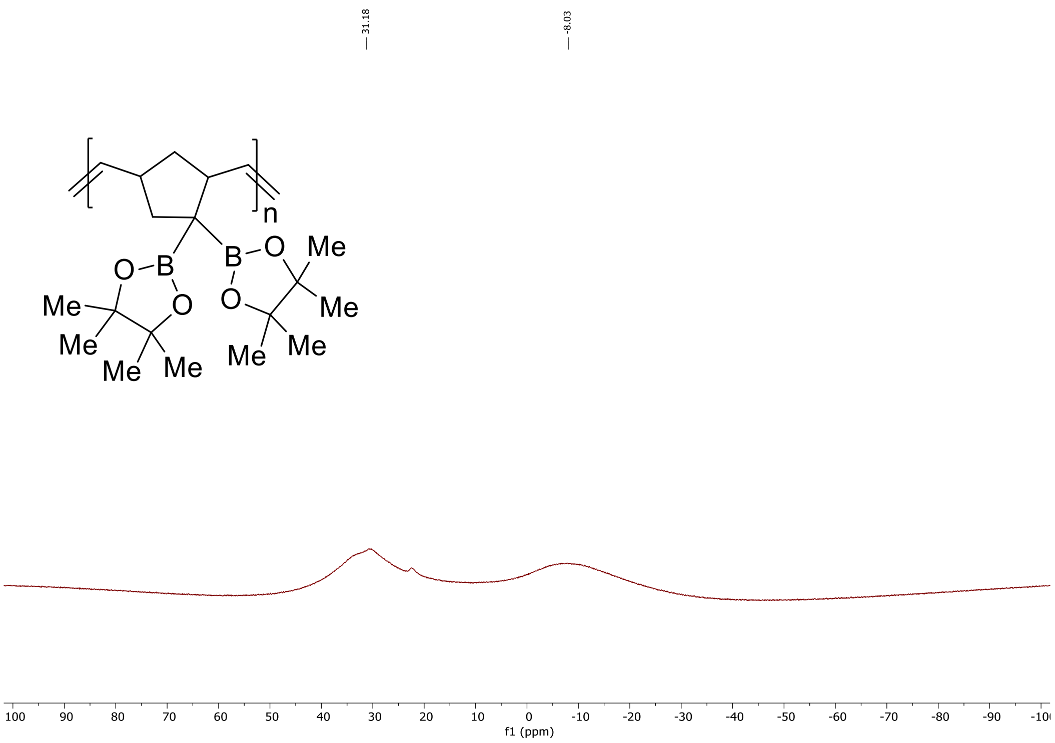

^{1}H NMR (400 MHz, $CDCl_3$) of compound (**poly-7-BpinBdan**)

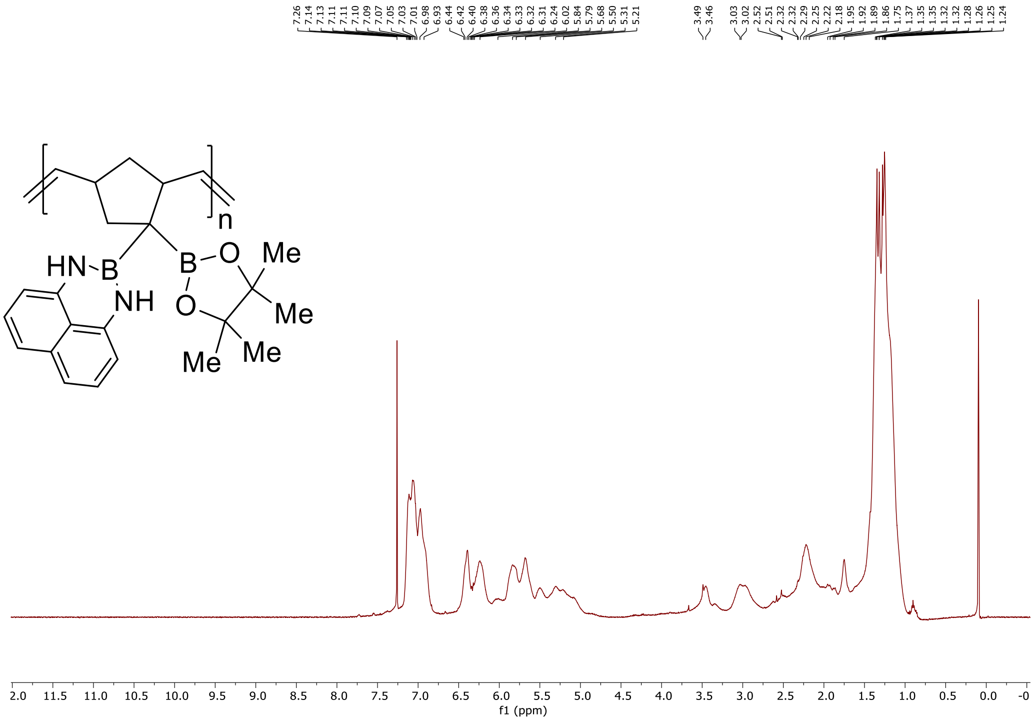

^{13}C NMR (126 MHz, $CDCl_3$) of compound (**poly-7-BpinBdan**)

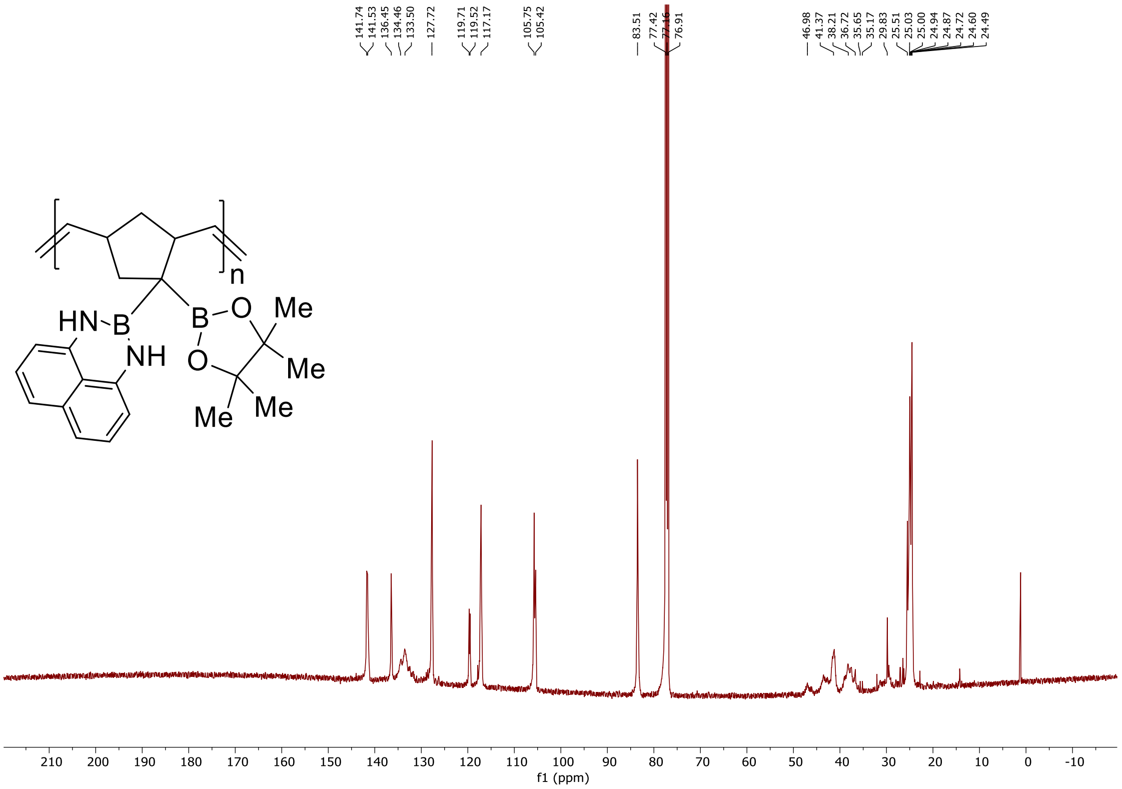

^{11}B NMR (128 MHz, $CDCl_3$) of compound (**poly-7-BpinBdan**)

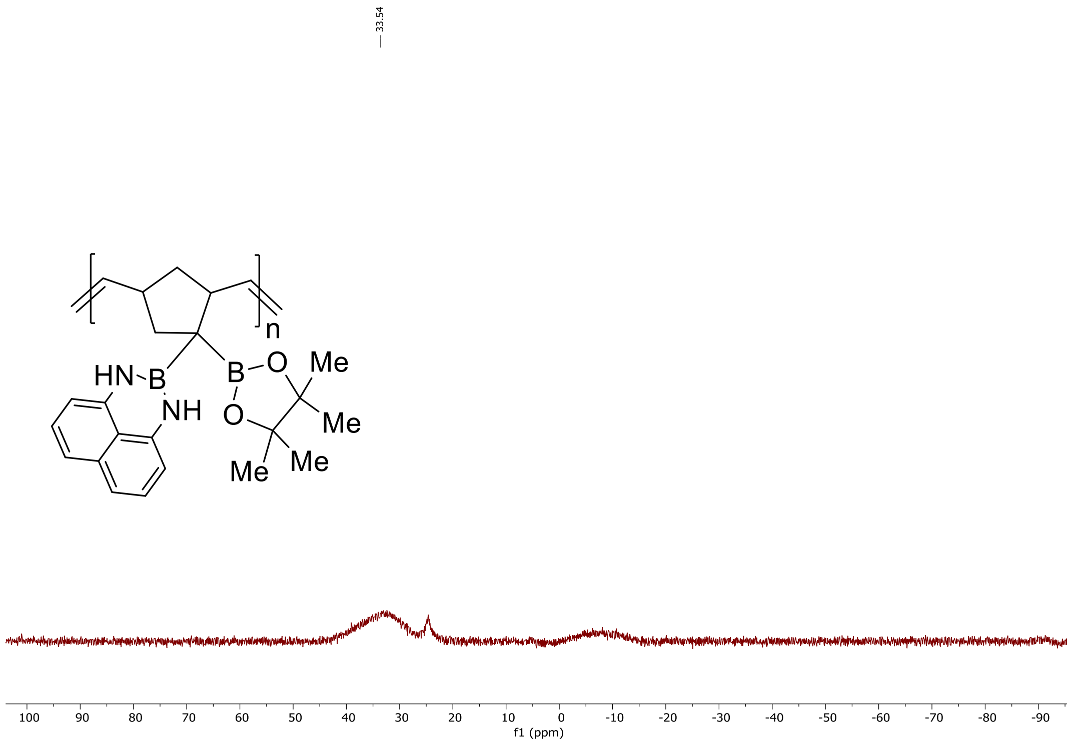

^{1}H NMR (500 MHz, $CDCl_3$) of compound (**poly-8-BpinBdan**)

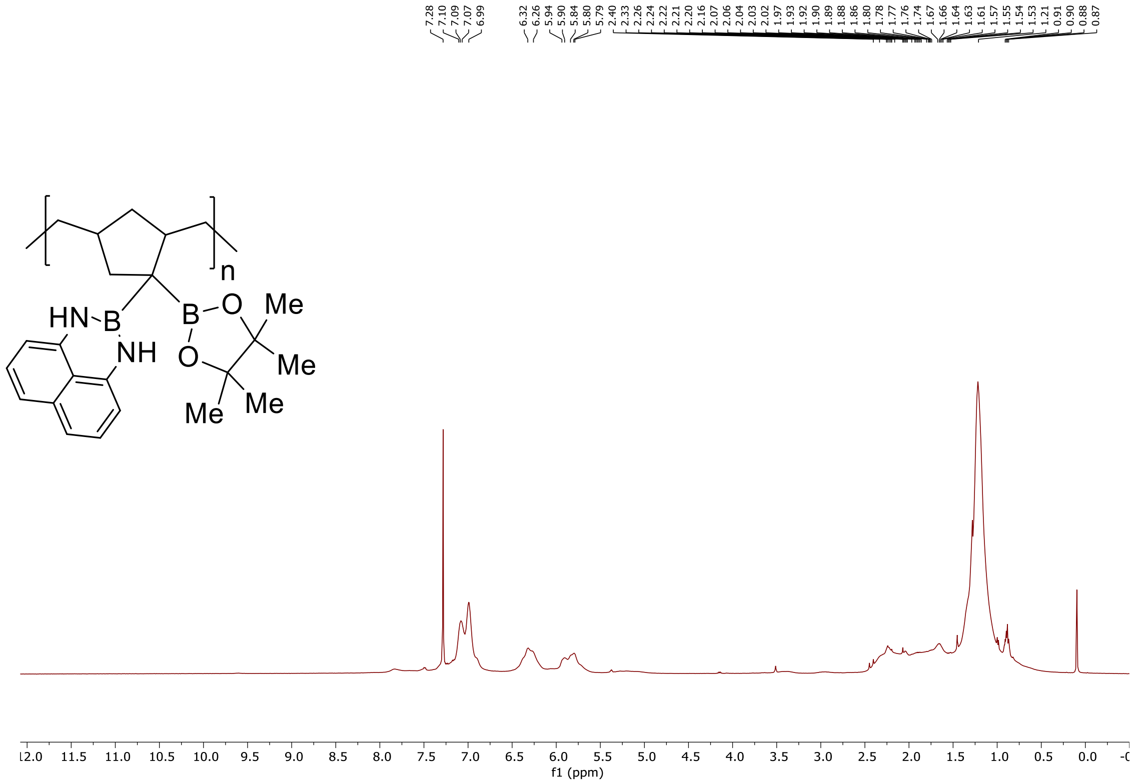

^{13}C NMR (126 MHz, $CDCl_3$) of compound (**poly-8-BpinBdan**)

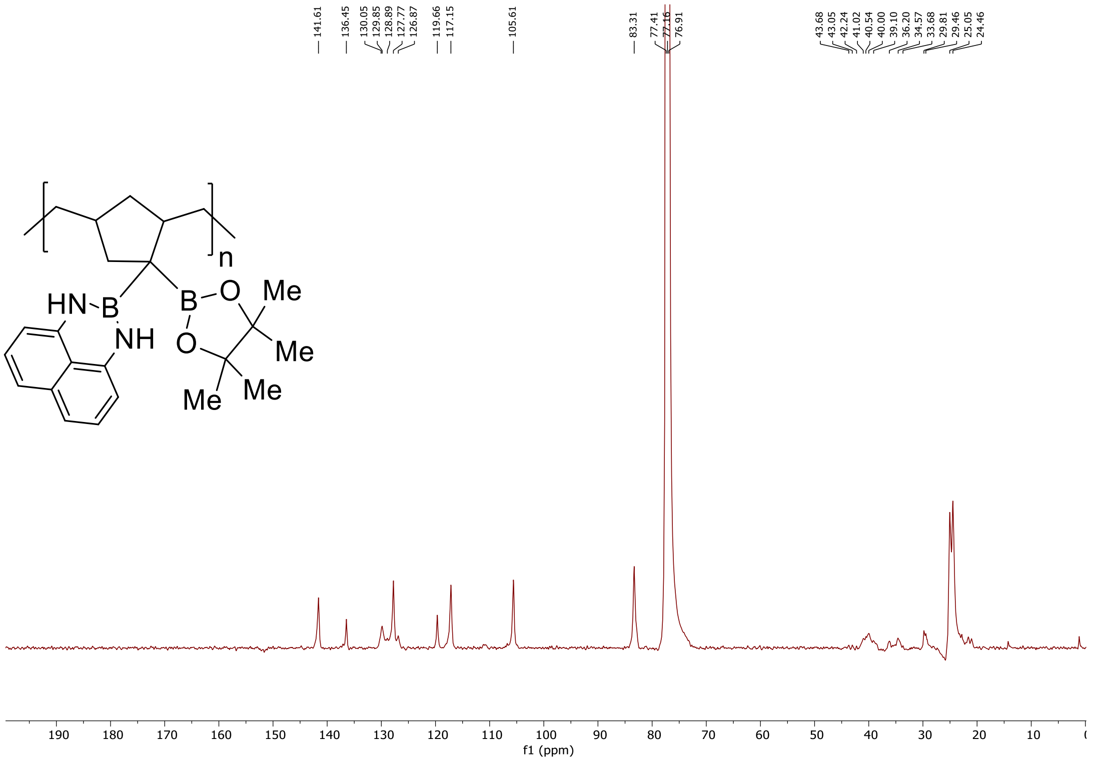

^{11}B NMR (161 MHz, $CDCl_3$) of compound (**poly-8-BpinBdan**)

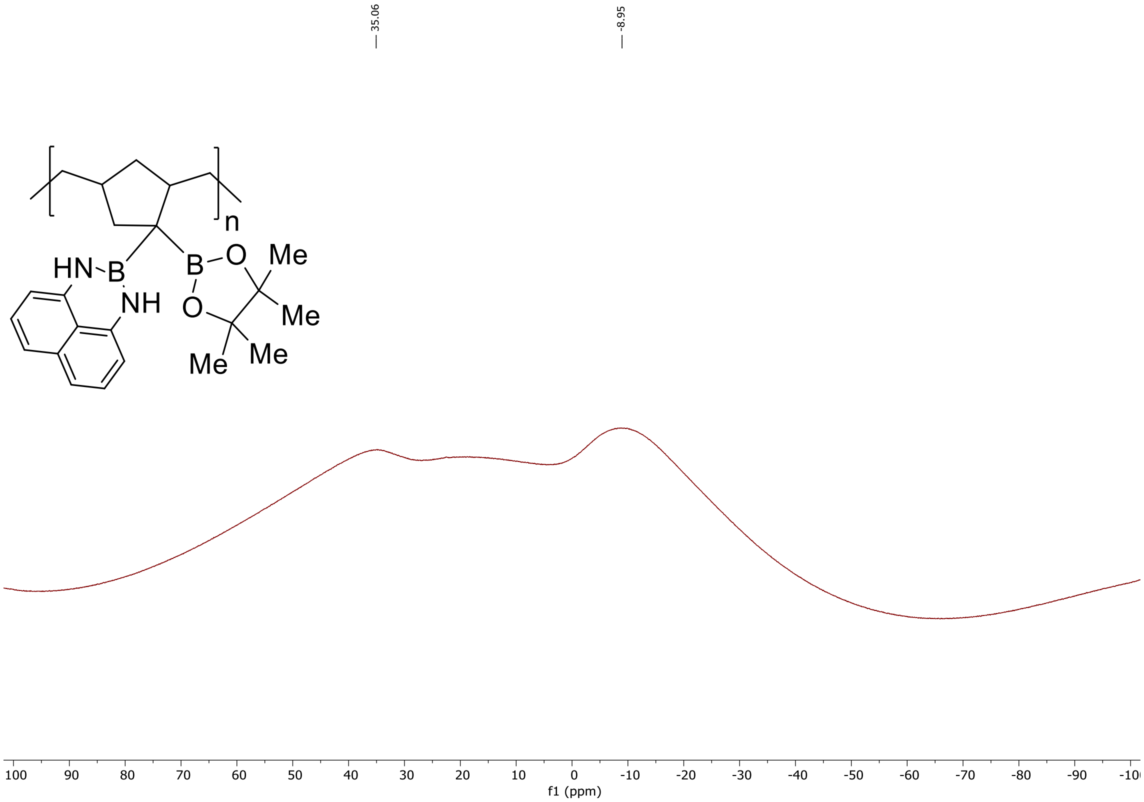

^{1}H NMR (500 MHz, $CDCl_3$) of compound (**poly-8-BpinBpin**)

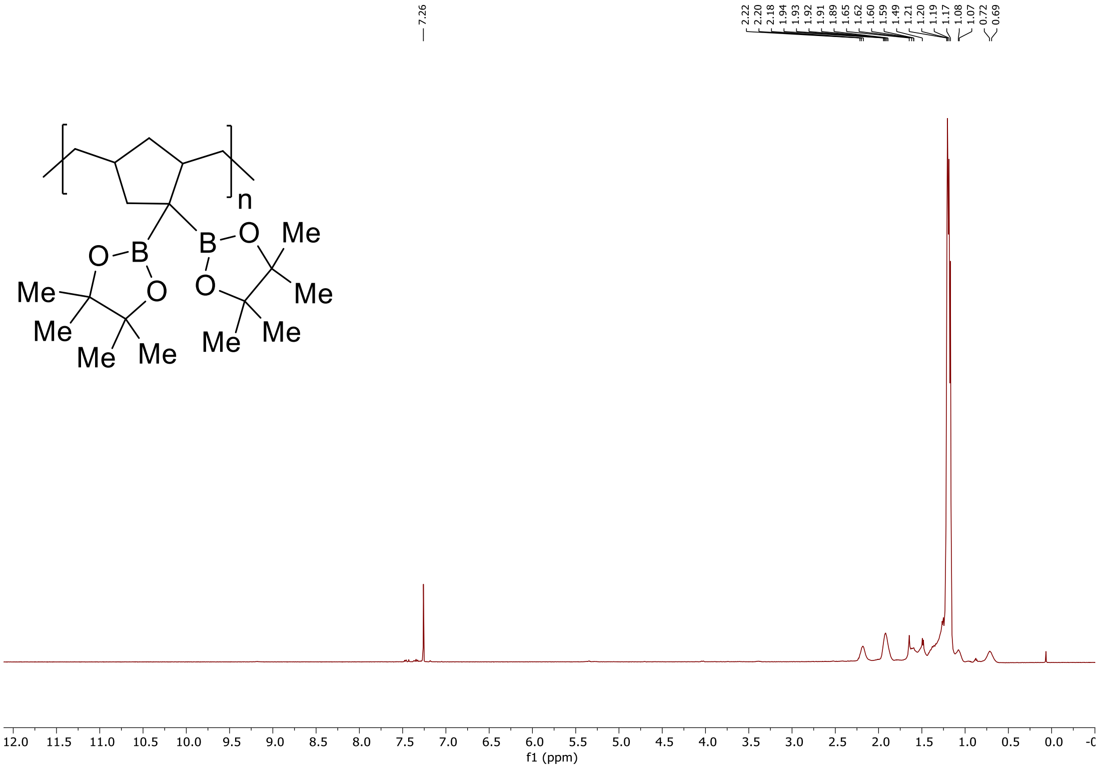

^{13}C NMR (126 MHz, CDCl$_3$) of compound (**poly-8-BpinBpin**)

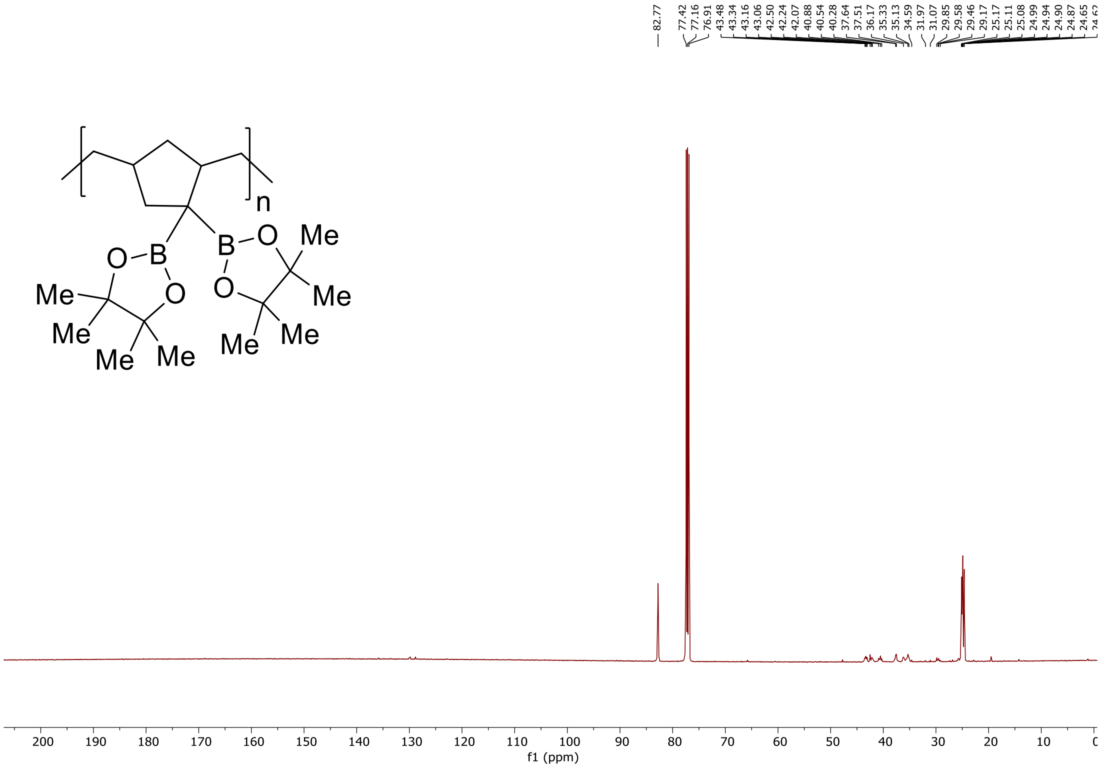

^{11}B NMR (161 MHz, CDCl$_3$) of compound (**poly-8-BpinBpin**)

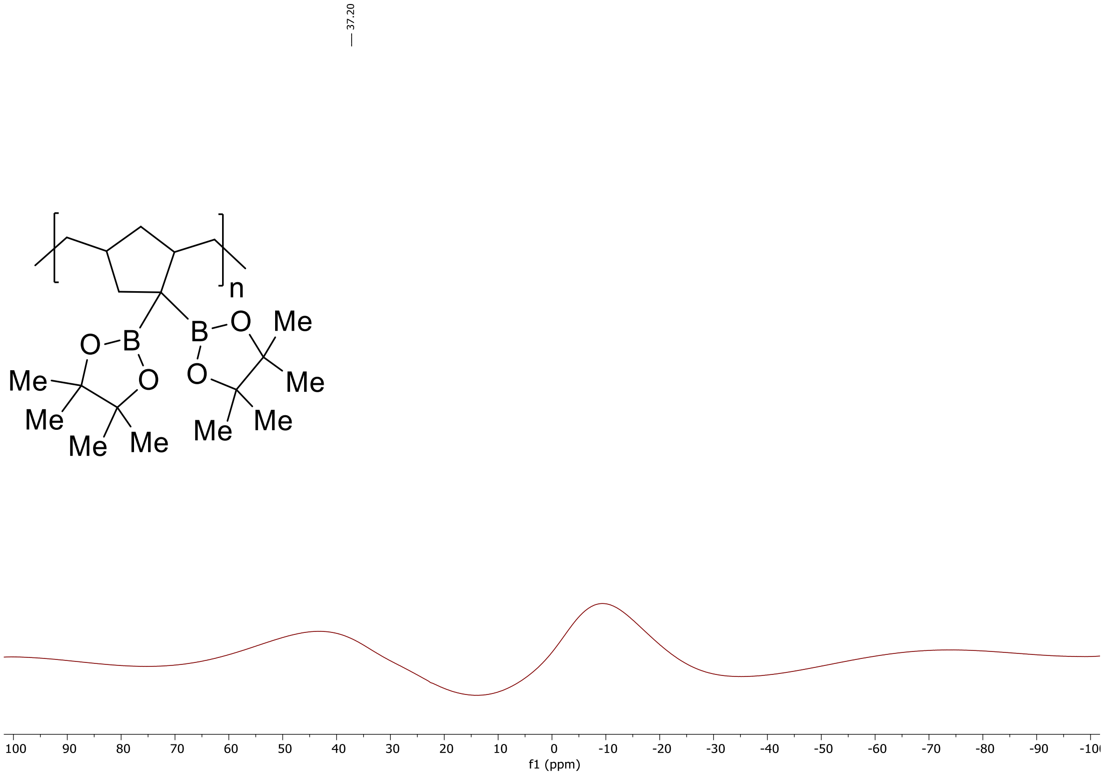

^{1}H NMR (500 MHz, DMSO-d_6) of compound (**poly-9-BpinBF$_3$K**)

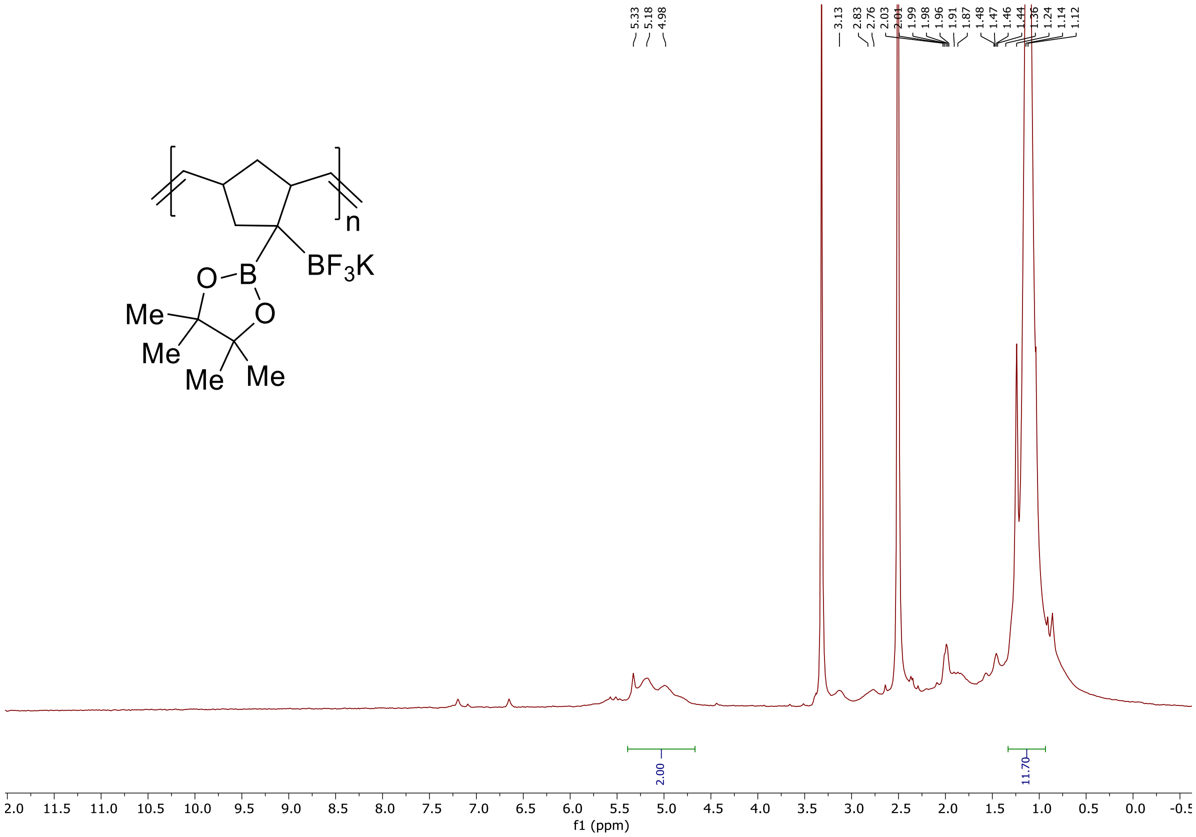

^{11}B NMR (161 MHz, DMSO-d_6) of compound (**poly-9-BpinBF$_3$K**)

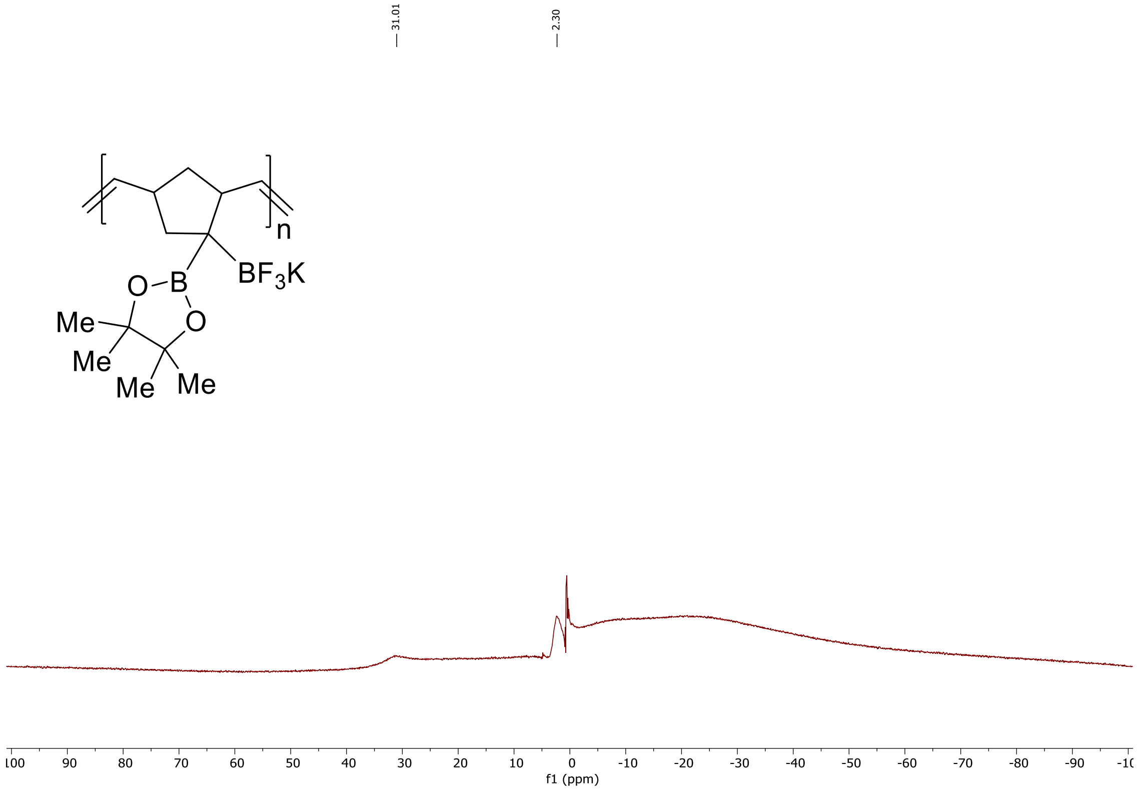

^{19}F NMR (471 MHz, DMSO-d_6) of compound (**poly-9-BpinBF3K**)

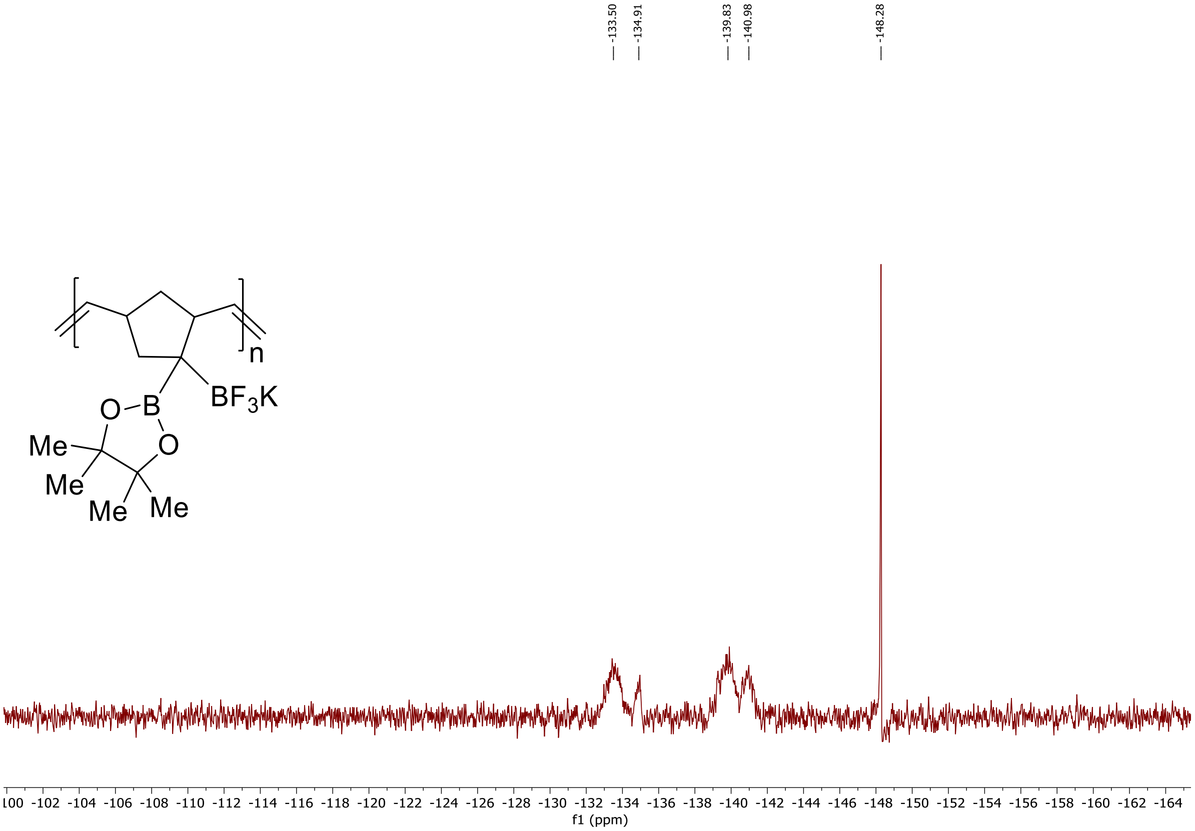

^{1}H NMR (500 MHz, $CDCl_3$) of compound (**poly-10-Bpin-H**)

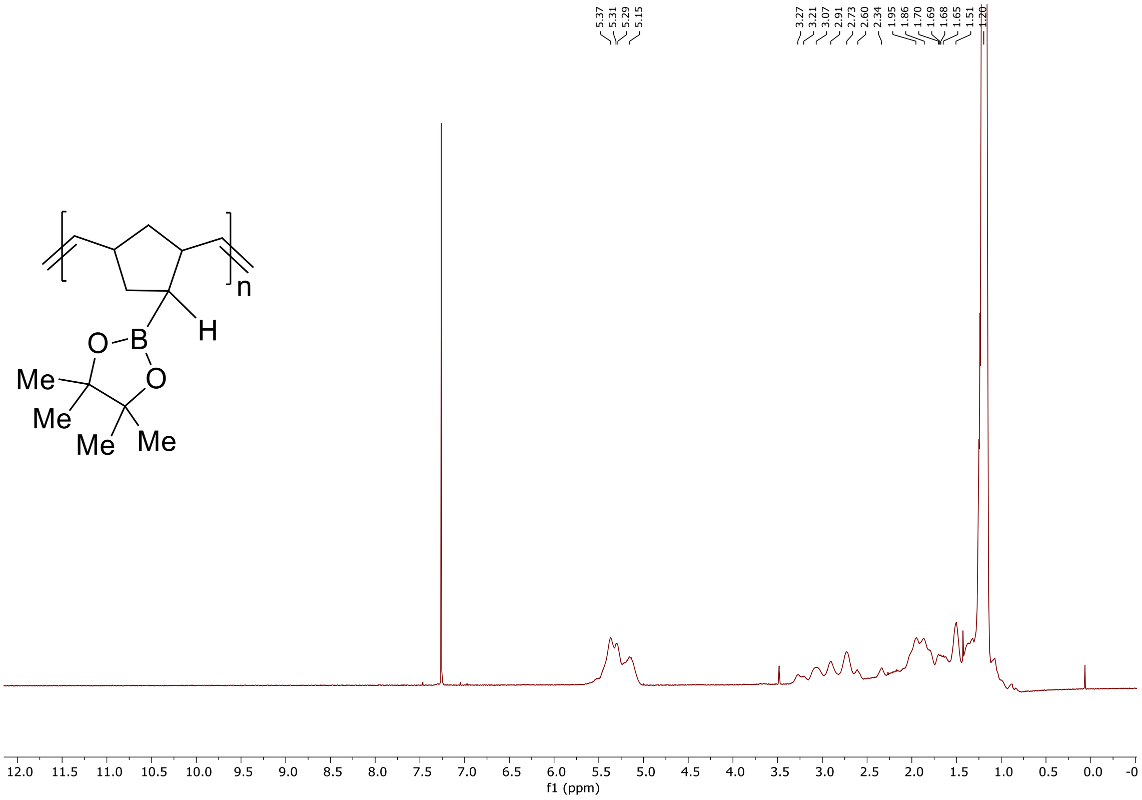

^{13}C NMR (126 MHz, $CDCl_3$) of compound (**poly-10-Bpin-H**)

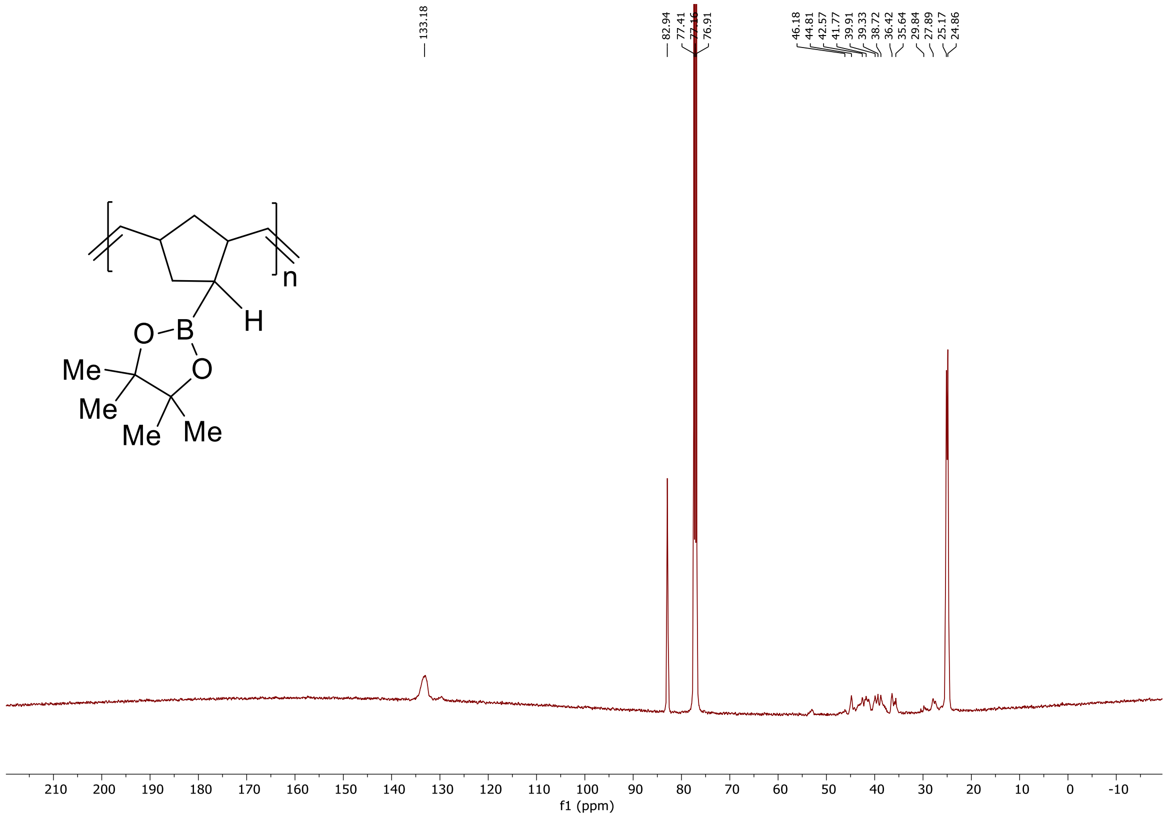

^{11}B NMR (161 MHz, $CDCl_3$) of compound (**poly-10-Bpin-H**)

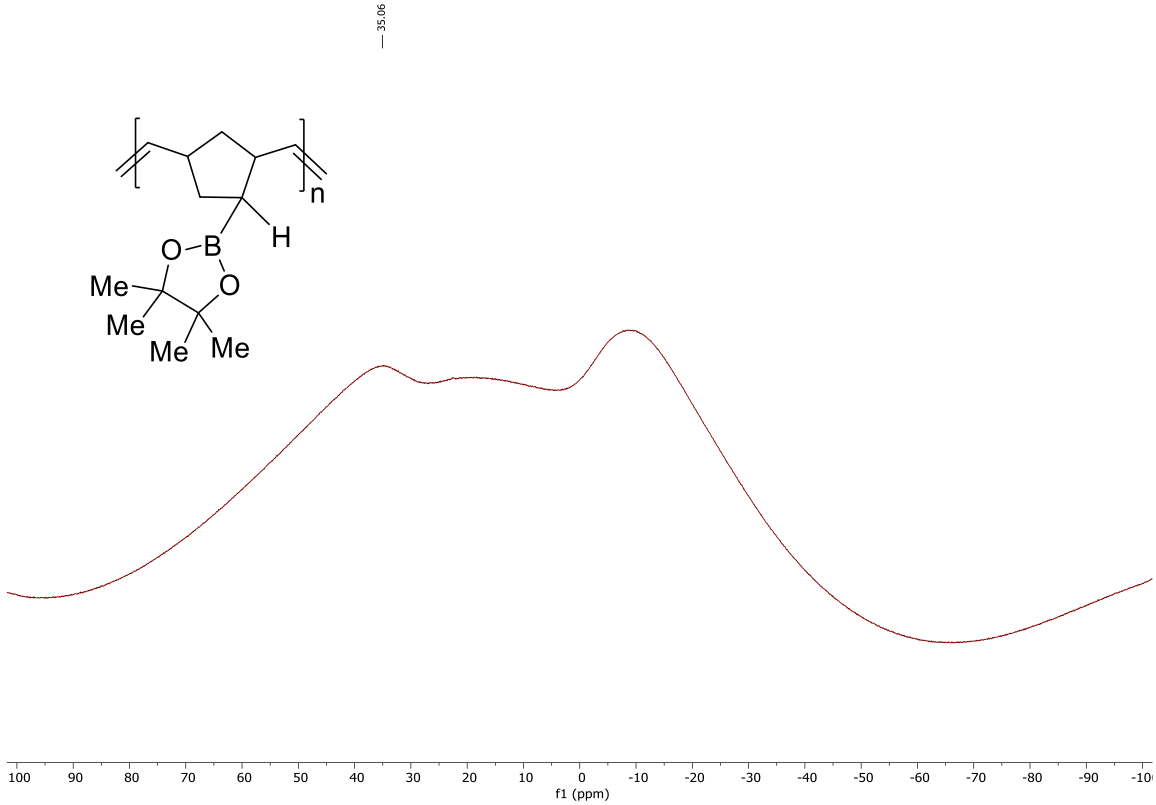

^{1}H NMR (500 MHz, $CDCl_3$) of compound (**poly-10-Bpin-D**)

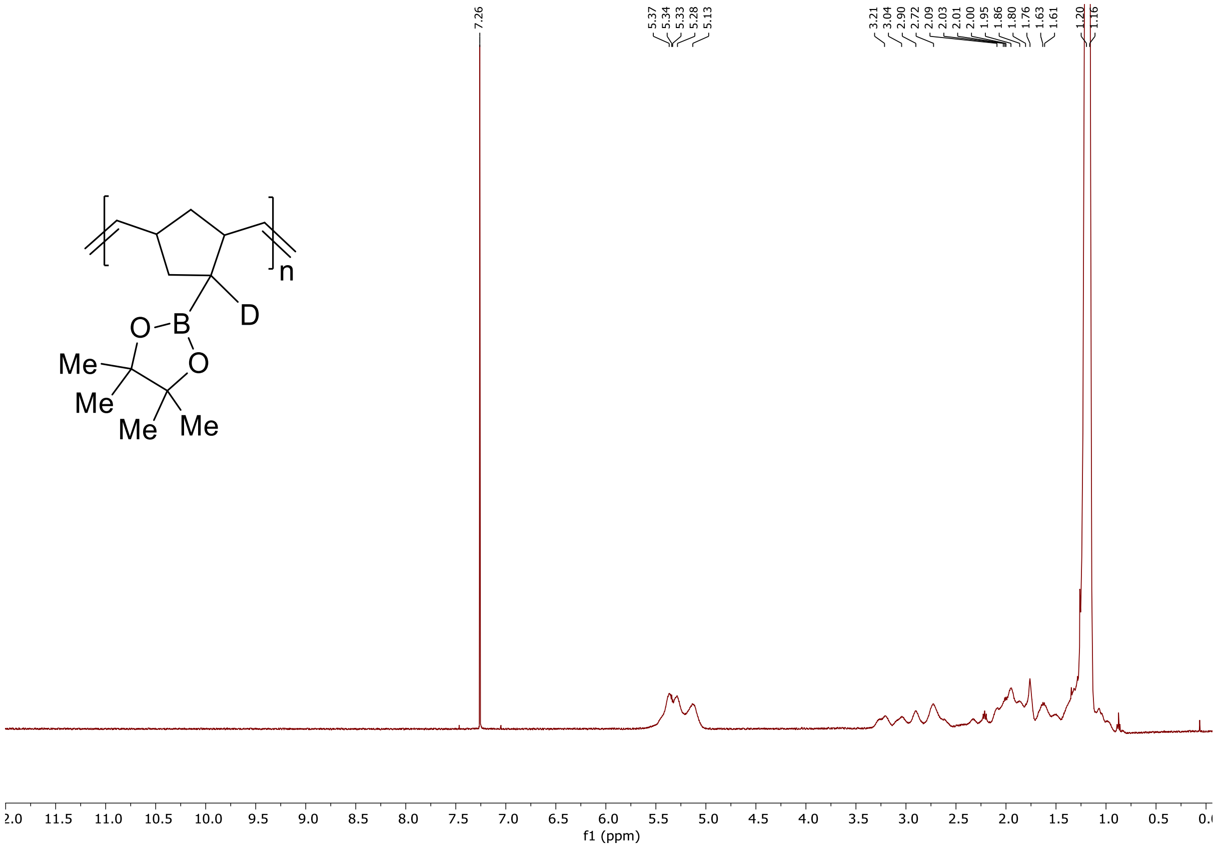

^{13}C NMR (126 MHz, $CDCl_3$) of compound (**poly-10-Bpin-D**)

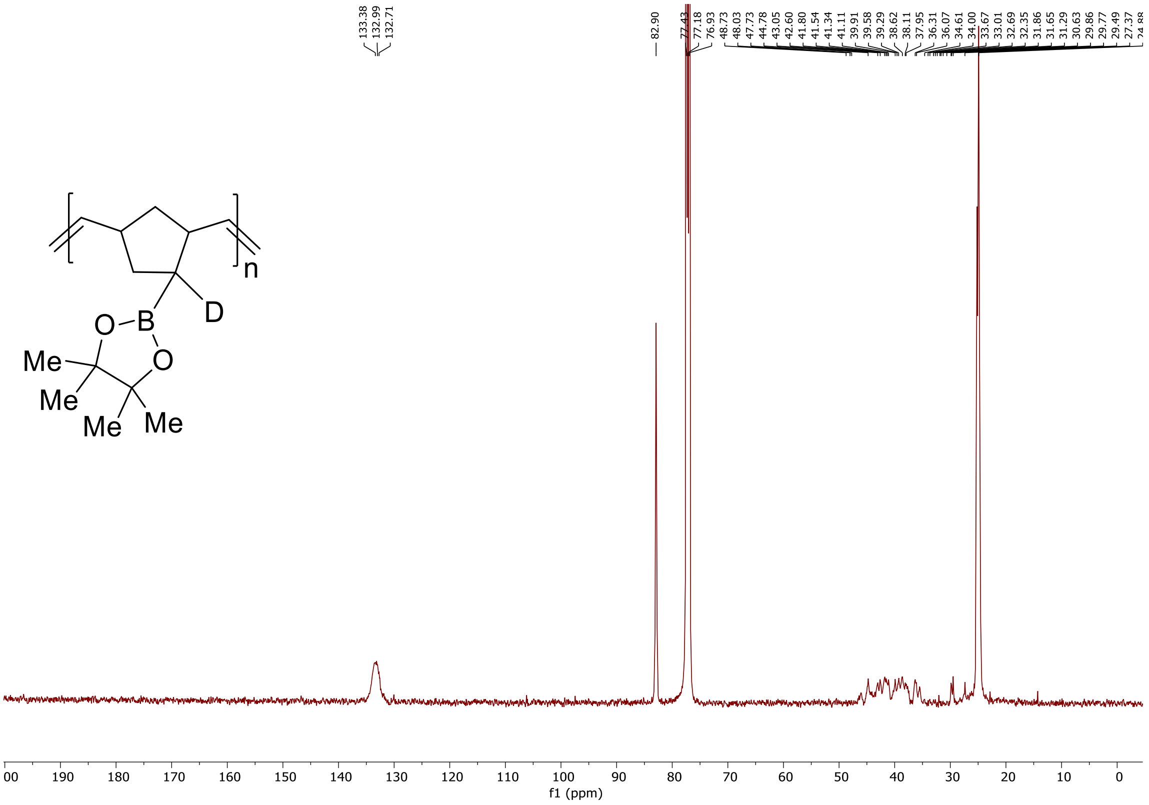

^{11}B NMR (161 MHz, $CDCl_3$) of compound (**poly-10-Bpin-D**)

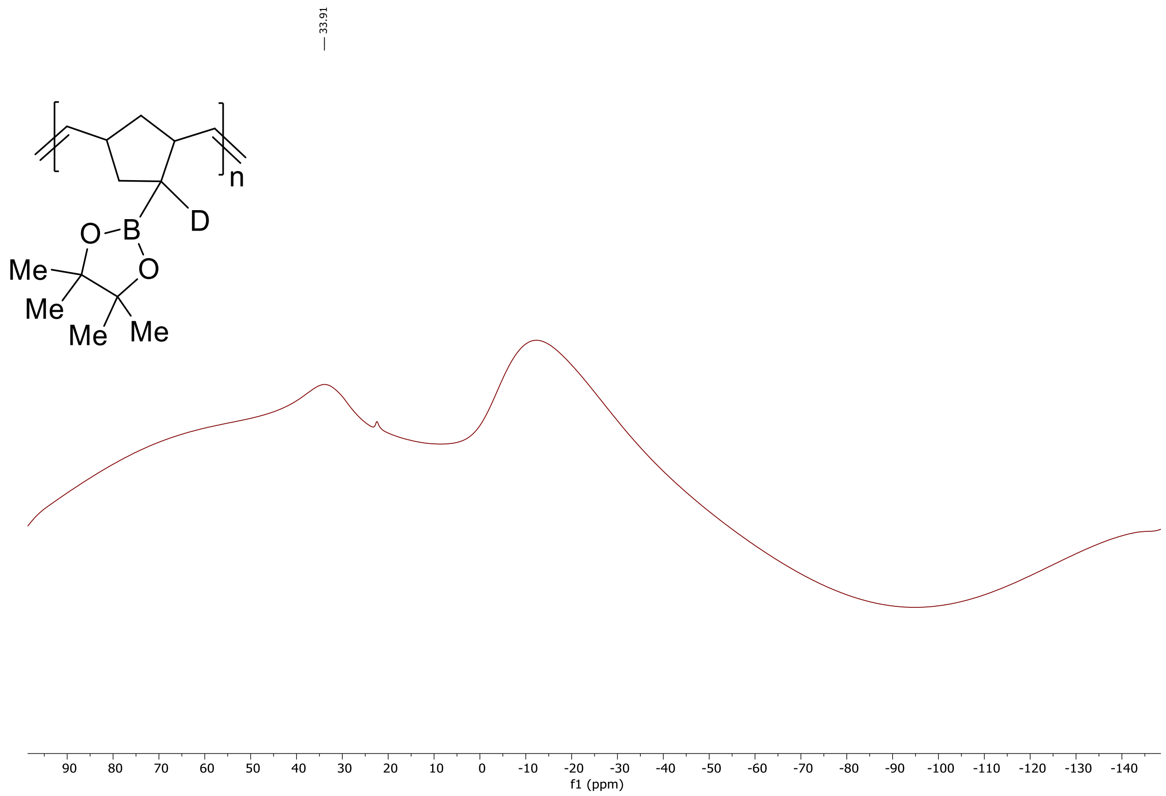

D NMR (77 MHz, $CDCl_3$) of compound (**poly-10-Bpin-D**)

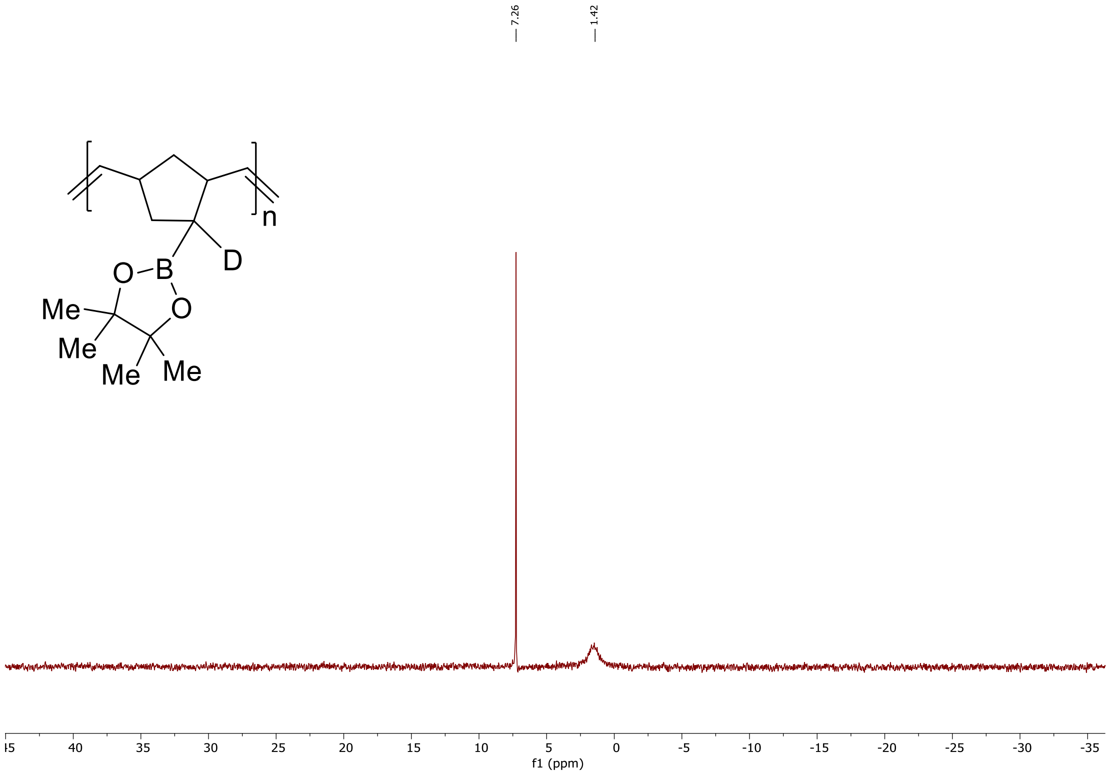

^{1}H NMR (500 MHz, $CDCl_3$) of compound (**poly-11-Bpin-H**)

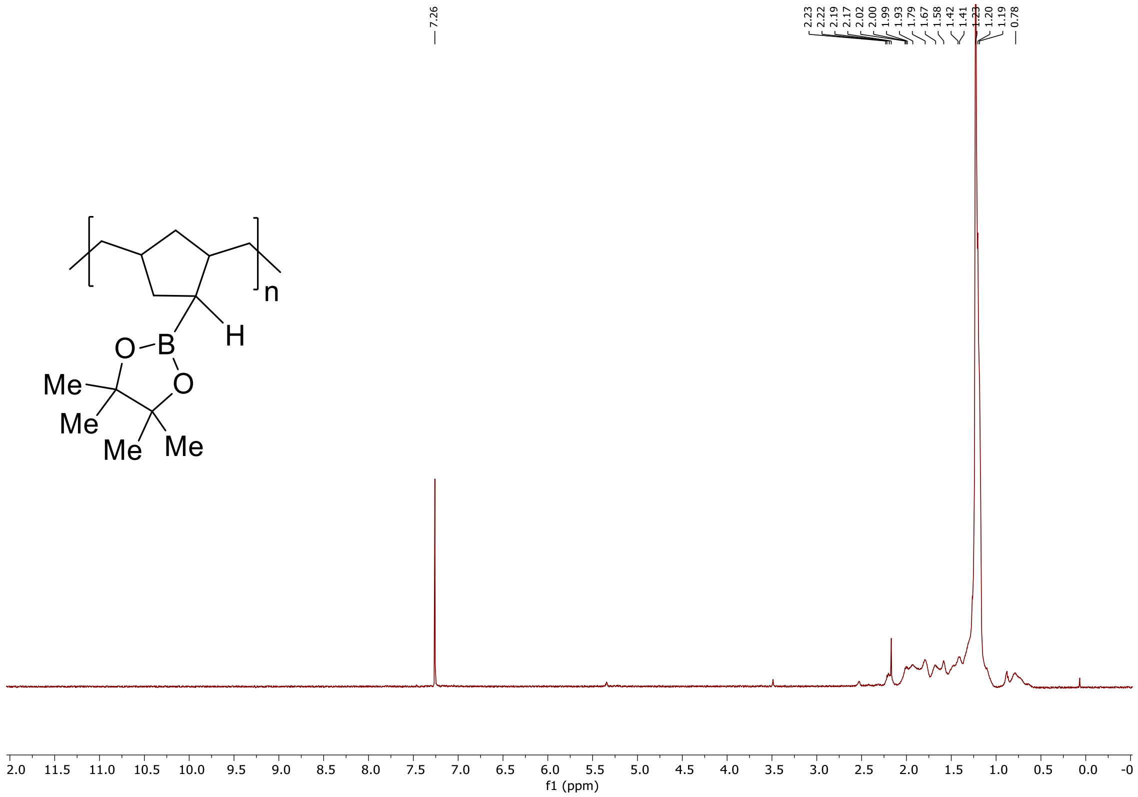

^{13}C NMR (126 MHz, $CDCl_3$) of compound (**poly-11-Bpin-H**)

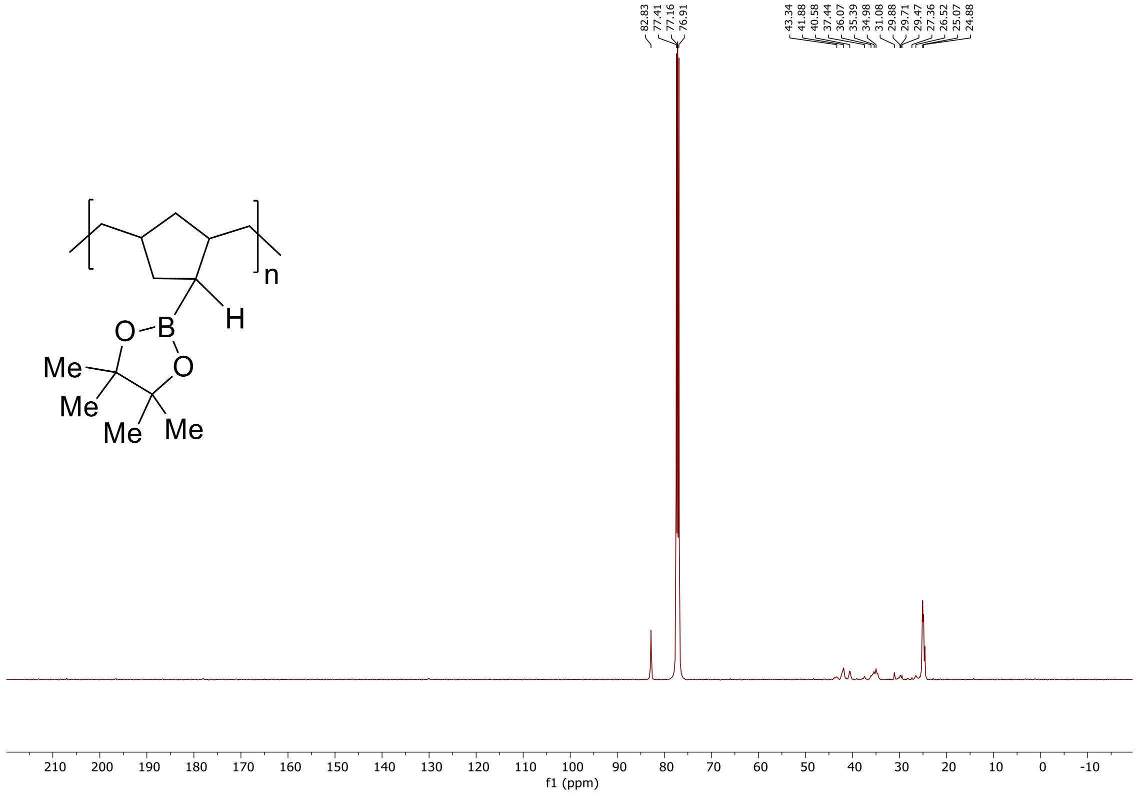

^{11}B NMR (161 MHz, $CDCl_3$) of compound (**poly-11-Bpin-H**)

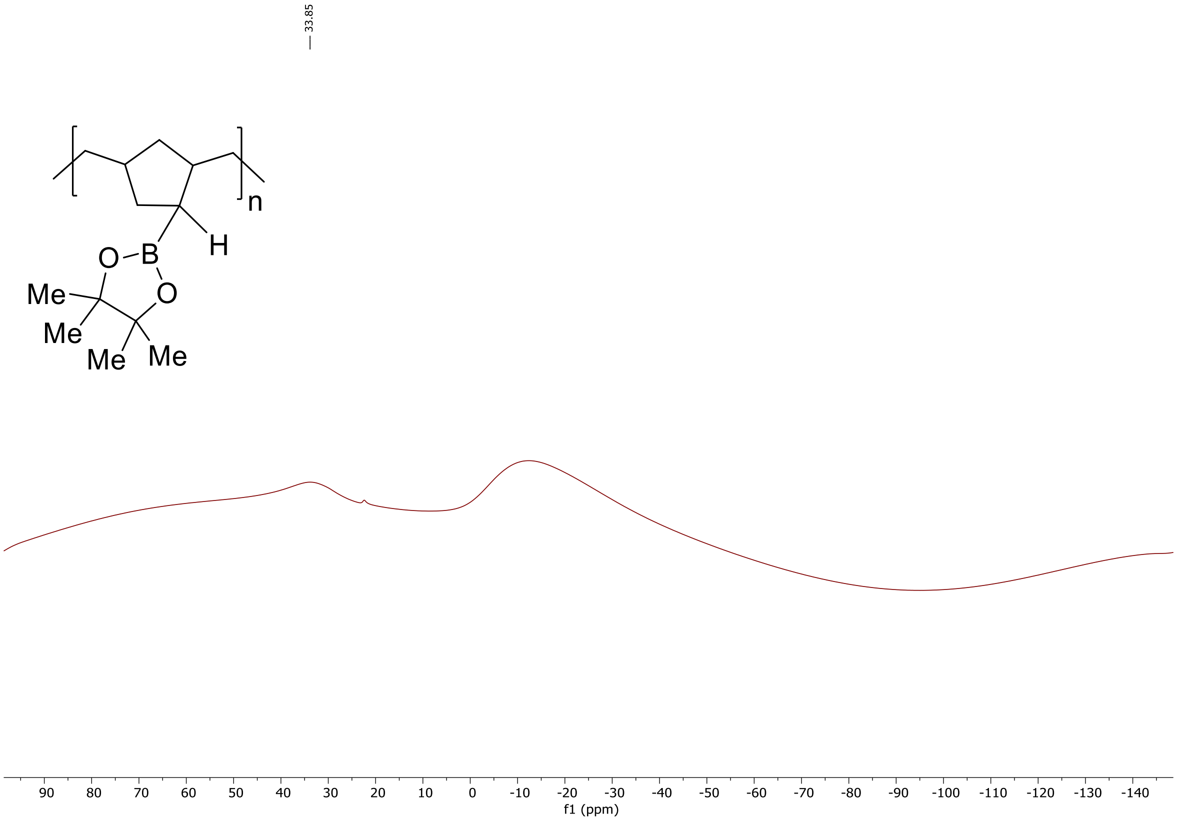

^{1}H NMR (500 MHz, $CDCl_3$) of compound (**poly-11-Bpin-D**)

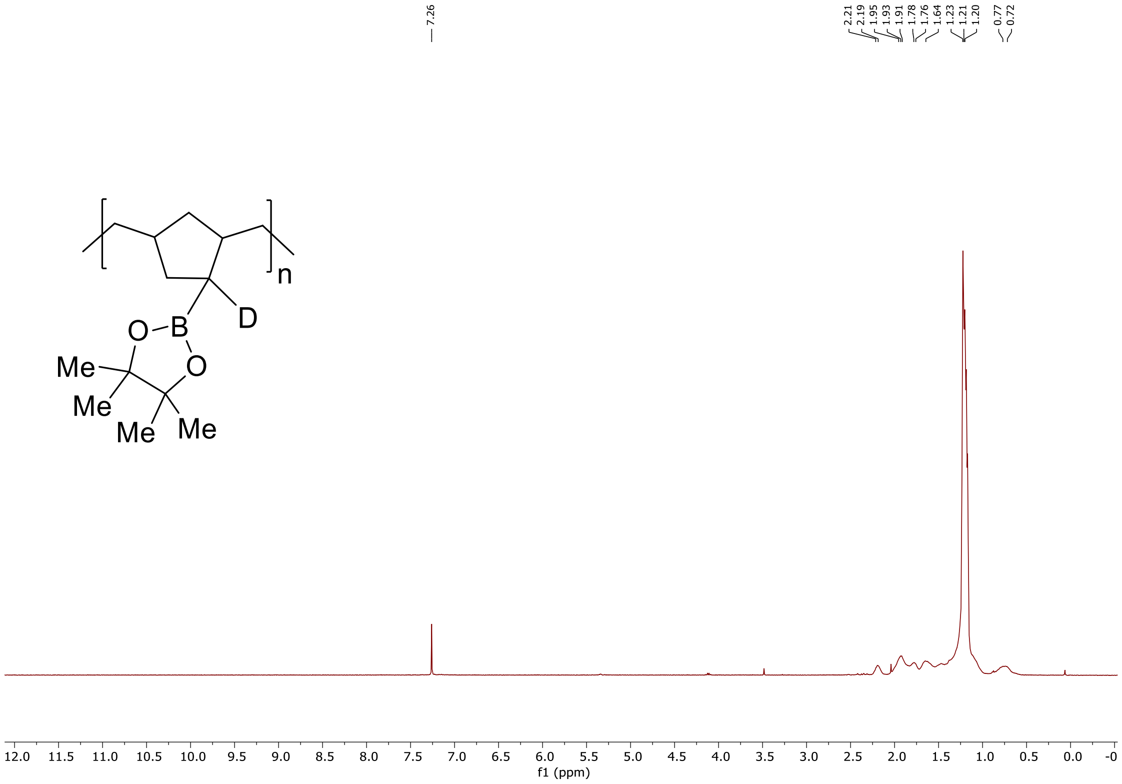

^{13}C NMR (126 MHz, $CDCl_3$) of compound (**poly-11-Bpin-D**)

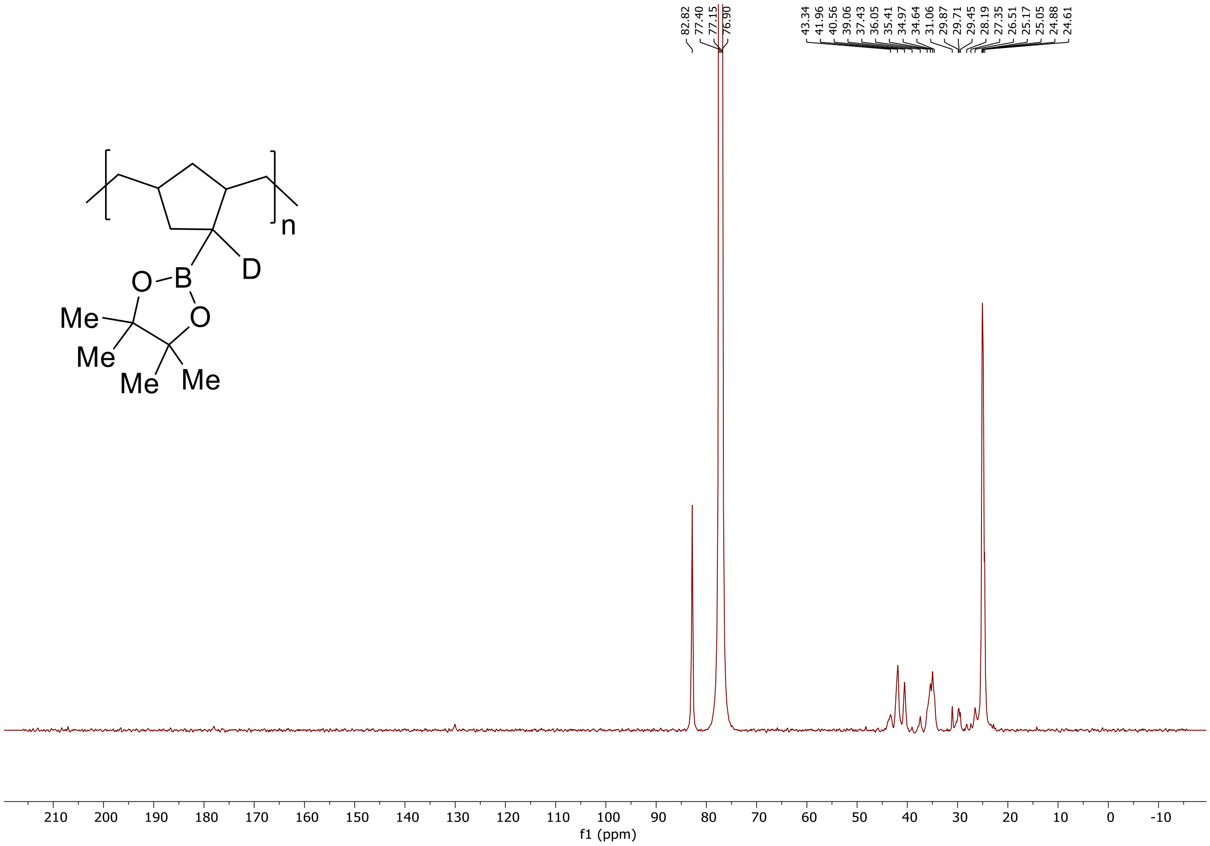

^{11}B NMR (161 MHz, $CDCl_3$) of compound (**poly-11-Bpin-D**)

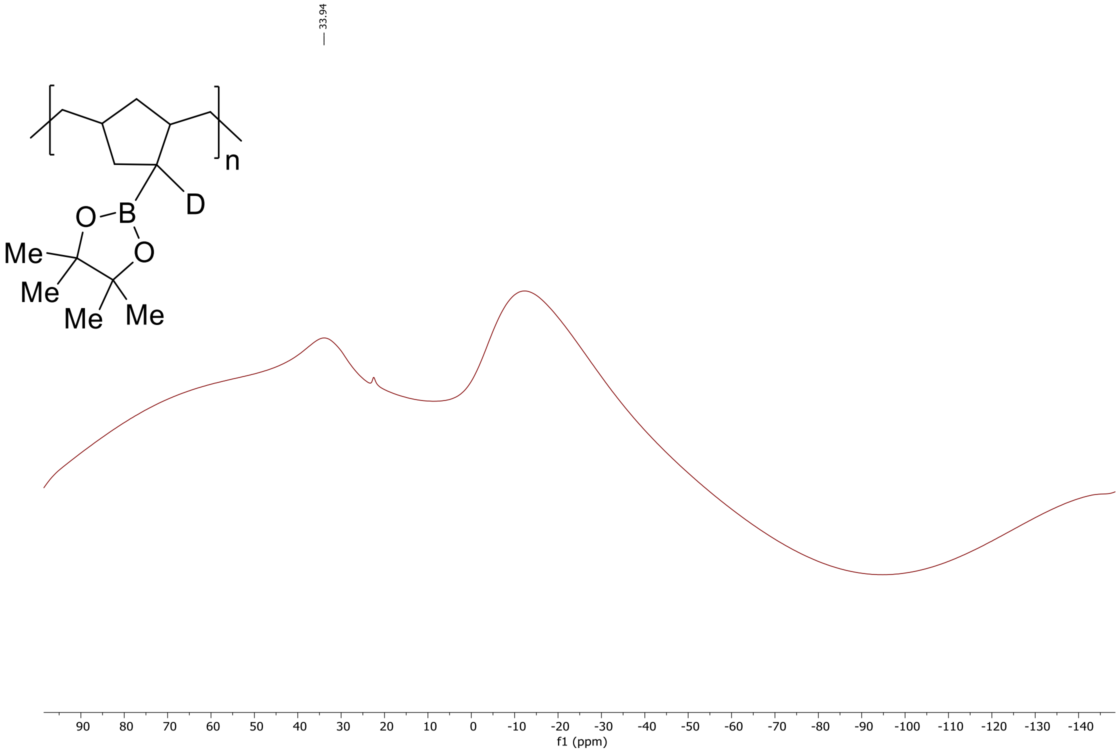

D NMR (77 MHz, $CDCl_3$) of compound (**poly-11-Bpin-D**)

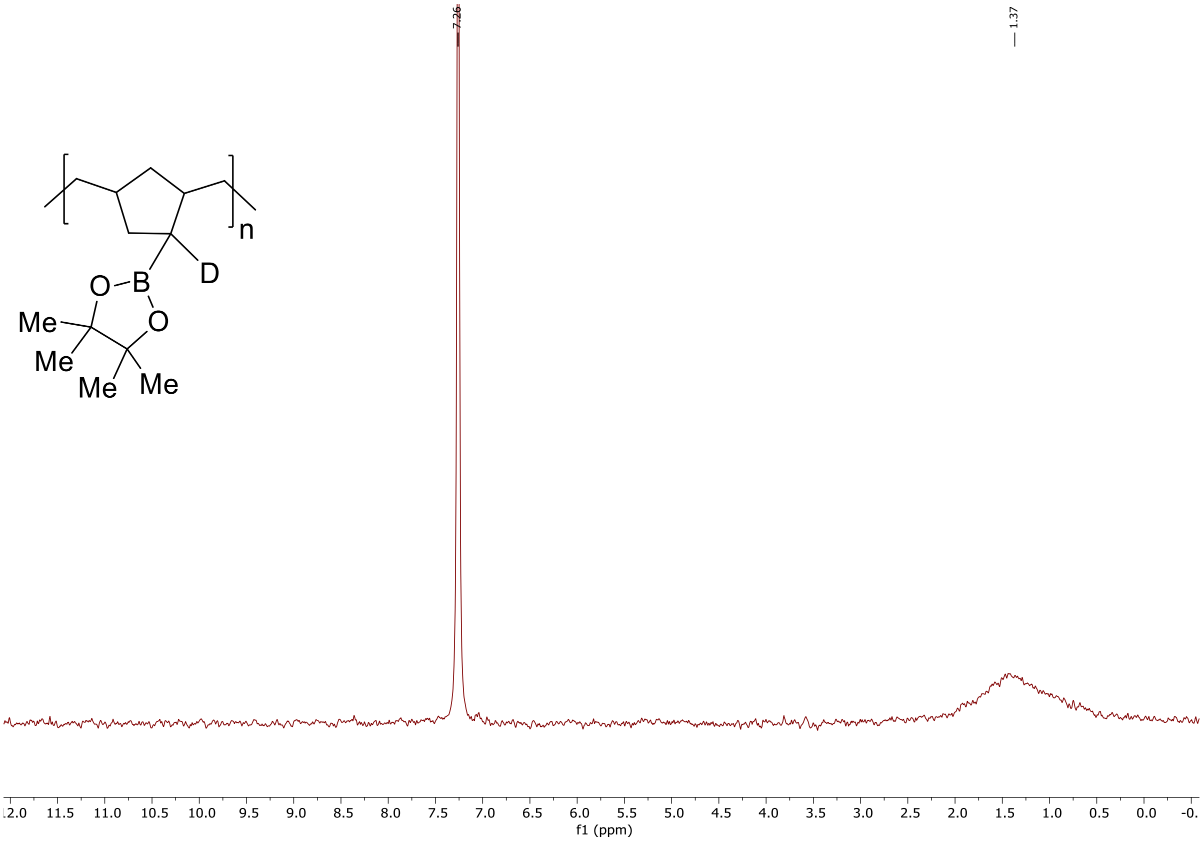

^{1}H NMR (500 MHz, DMSO-d_6) of compound (**poly-12-BF$_3$K-H**)

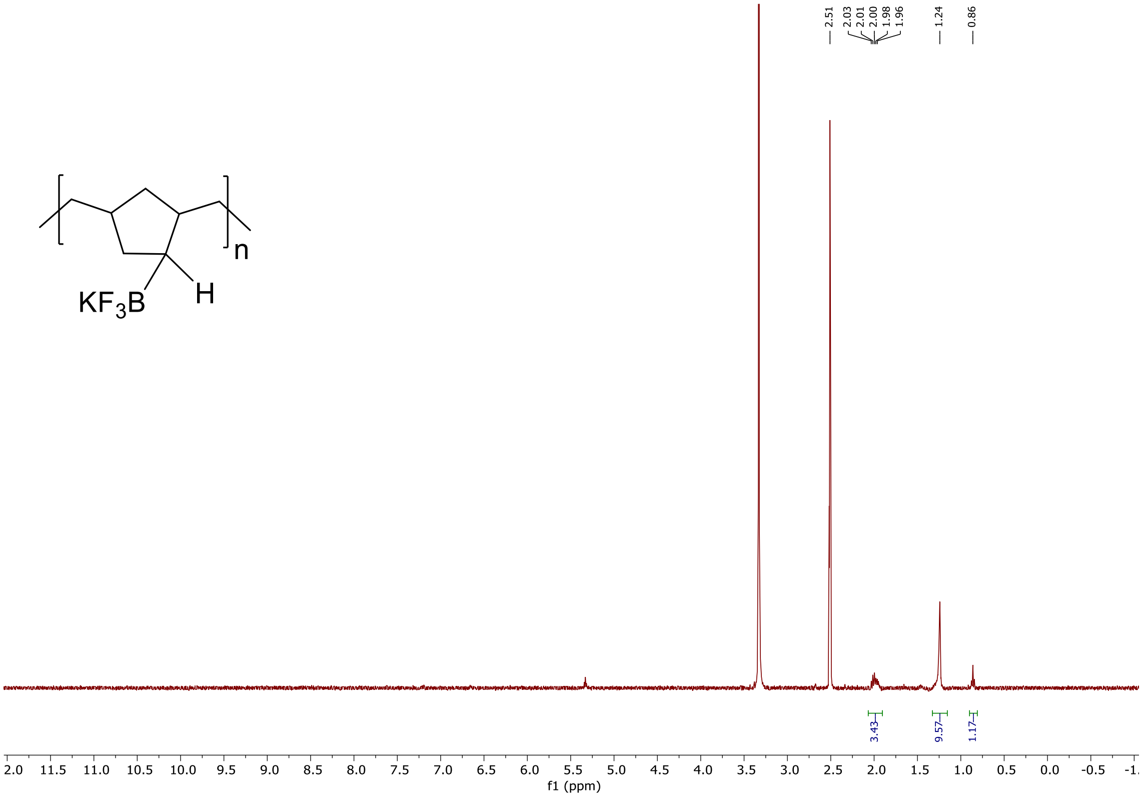

^{11}B NMR (161 MHz, DMSO-d_6) of compound (**poly-12-BF$_3$K-H**)

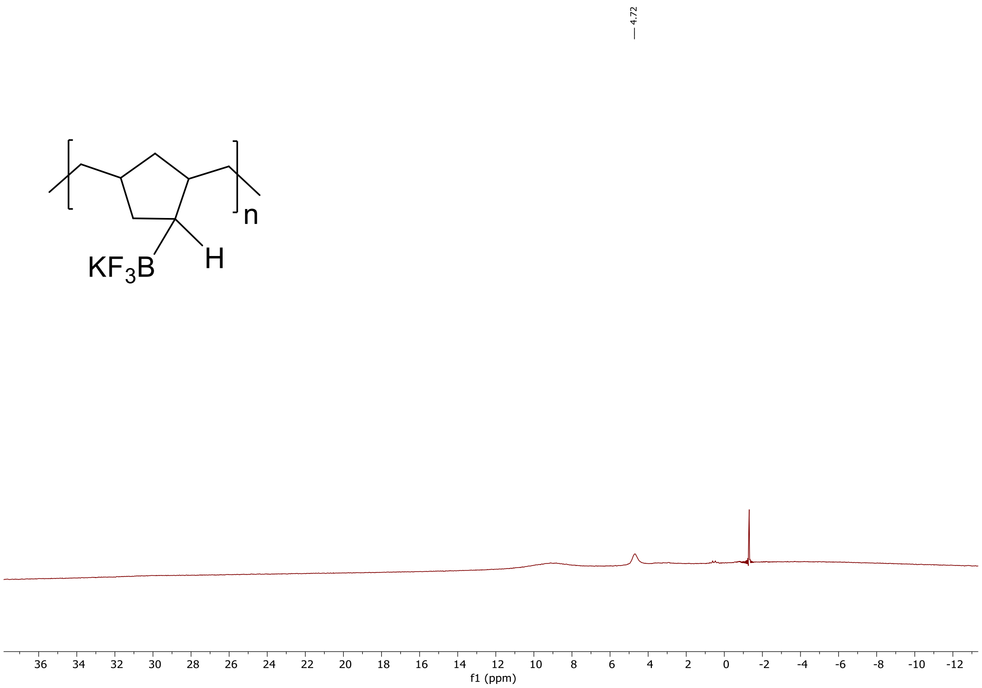

^{19}F NMR (471 MHz, DMSO-d_6) of compound (**poly-12-BF$_3$K-H**)

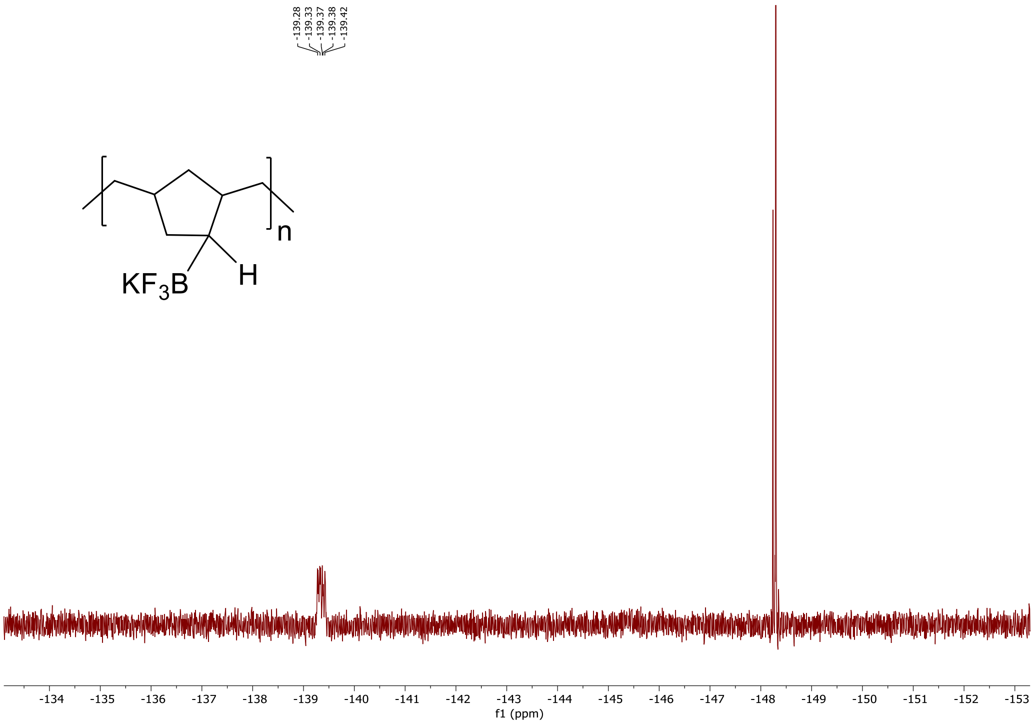

^{1}H NMR (500 MHz, $CDCl_3$) of compound (**poly-14-Ar-H**)

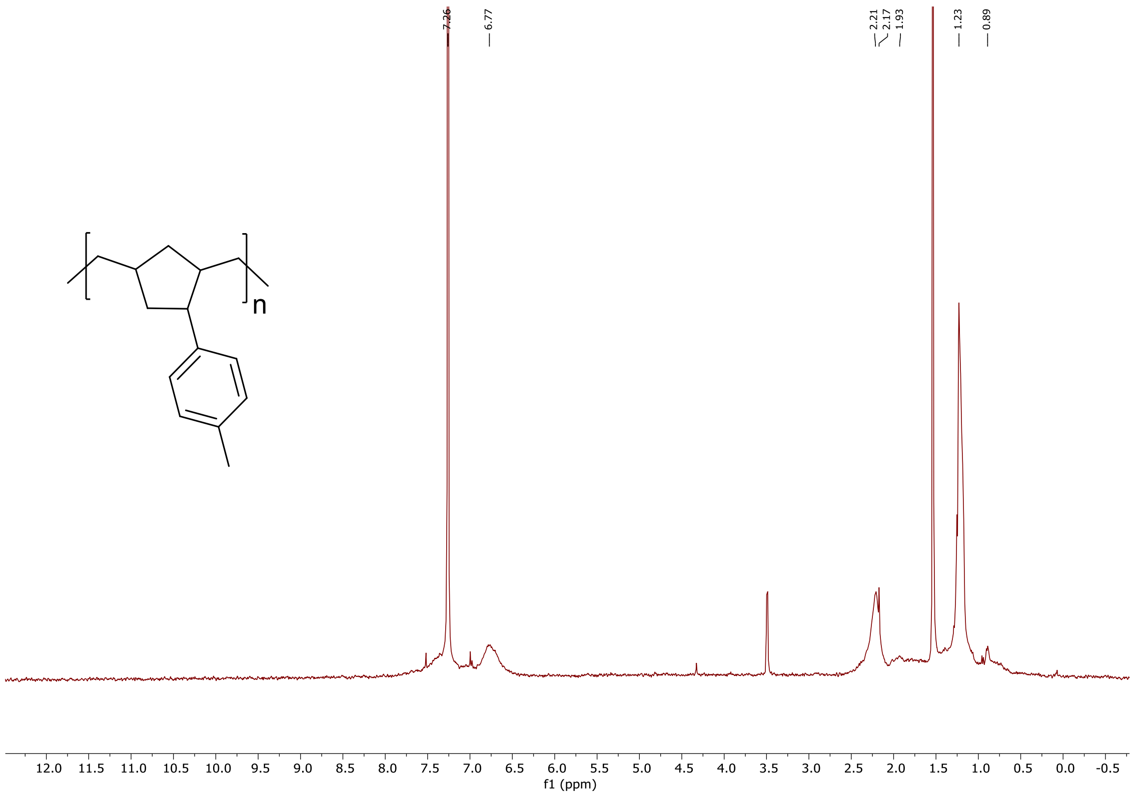

^{13}C NMR (126 MHz, $CDCl_3$) of compound (**poly-14-Ar-H**)

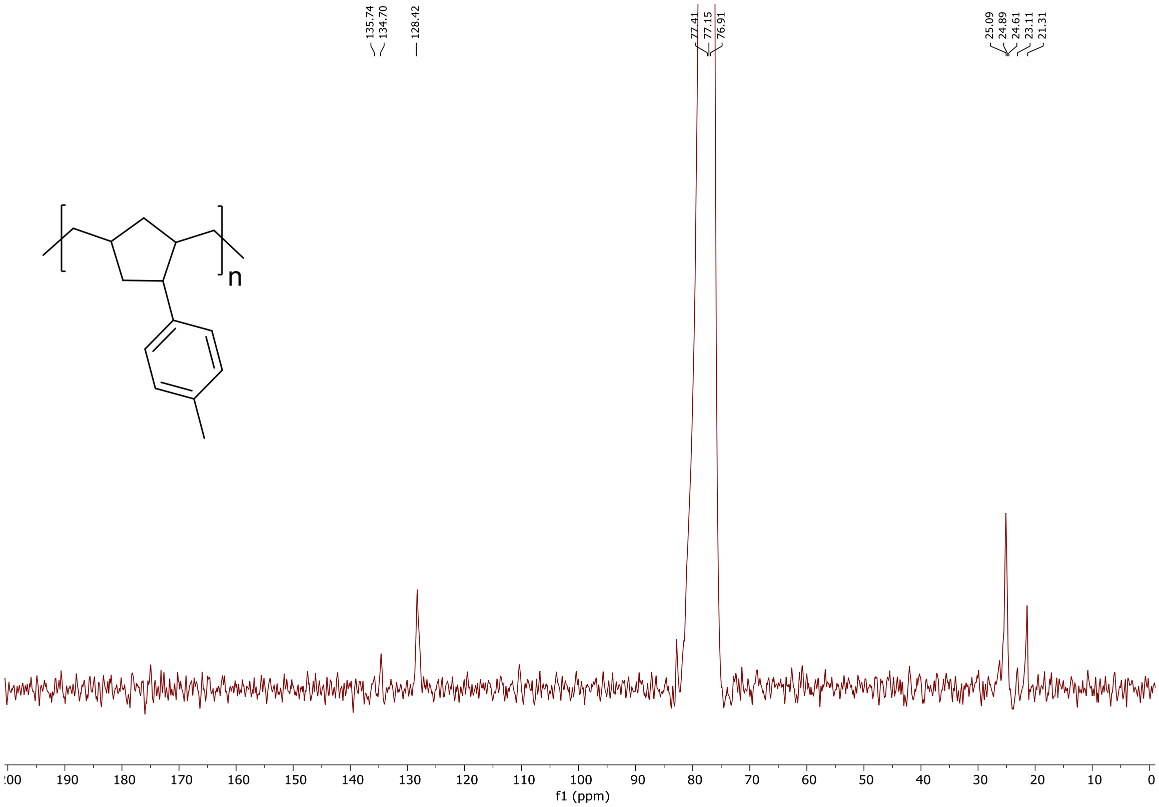

10. X-Ray Crystallography Data

Crystallographic Data for compound (4c)

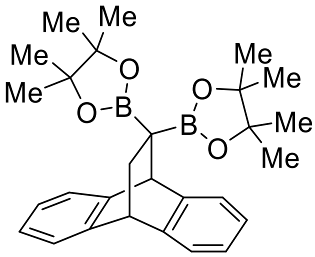

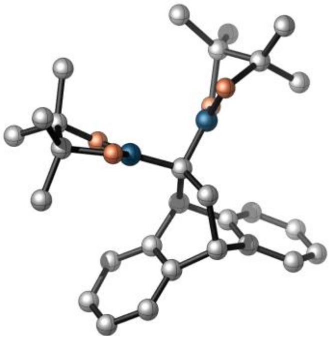

4c

Table 1 Crystal data and structure refinement for AhmadM5a.

Identification code	AhmadM5a
Empirical formula	C28H36B2O4
Formula weight	458.19
Temperature/K	295.2(4)
Crystal system	monoclinic
Space group	P21
a/Å	10.4619(3)
b/Å	11.8333(4)
c/Å	10.9918(3)
α/°	90
β/°	98.107(3)
γ/°	90
Volume/Å3	1347.17(7)
Z	2
ρ_{calc}g/cm3	1.130
μ/mm-1	0.072
F(000)	492.0
Crystal size/mm3	0.354 × 0.223 × 0.063
Radiation	Mo Kα (λ = 0.71073)
2Θ range for data collection/°	3.742 to 61.77
Index ranges	-14 ≤ h ≤ 14, -15 ≤ k ≤ 15, -14 ≤ l ≤ 15
Reflections collected	17790
Independent reflections	6352 [R_{int} = 0.0391, R_{sigma} = 0.0382]
Data/restraints/parameters	6352/1/351
Goodness-of-fit on F2	1.022
Final R indexes [I>=2σ (I)]	R_1 = 0.0476, wR_2 = 0.1263
Final R indexes [all data]	R_1 = 0.0627, wR_2 = 0.1339
Largest diff. peak/hole / e Å-3	0.20/-0.16
Flack parameter	-0.9(5)

Crystallographic Data for compound (4k)

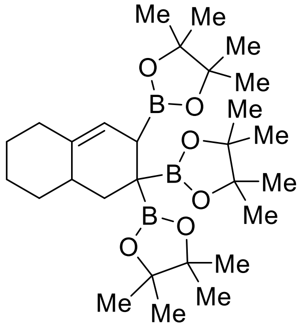

4k

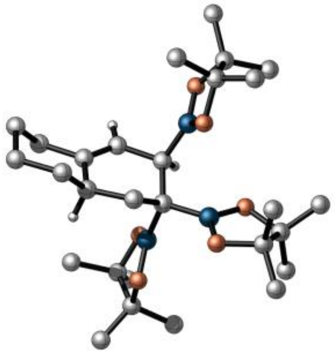

Table 1 Crystal data and structure refinement for AhmadM18LB_auto.

Identification code	AhmadM18LB_auto
Empirical formula	$C_{30}H_{51}B_3Cl_6O_6$
Formula weight	752.83
Temperature/K	150.00(10)
Crystal system	triclinic
Space group	P-1
a/Å	11.34290(10)
b/Å	11.36440(10)
c/Å	16.3938(2)
α/°	108.5670(10)
β/°	91.1740(10)
γ/°	102.8150(10)
Volume/Å3	1943.84(4)
Z	2
ρ_{calc}g/cm3	1.286
μ/mm-1	0.479
F(000)	792.0
Crystal size/mm3	0.57 × 0.31 × 0.31
Radiation	Mo Kα (λ = 0.71073)
2Θ range for data collection/°	3.914 to 62.142
Index ranges	-15 ≤ h ≤ 16, -16 ≤ k ≤ 16, -23 ≤ l ≤ 23
Reflections collected	208897
Independent reflections	11592 [R_{int} = 0.0363, R_{sigma} = 0.0137]
Data/restraints/parameters	11592/0/471
Goodness-of-fit on F^2	1.066
Final R indexes [I>=2σ (I)]	R_1 = 0.0494, wR_2 = 0.1381
Final R indexes [all data]	R_1 = 0.0592, wR_2 = 0.1466
Largest diff. peak/hole / e Å-3	0.75/-0.52

Crystallographic Data for compound (5a)

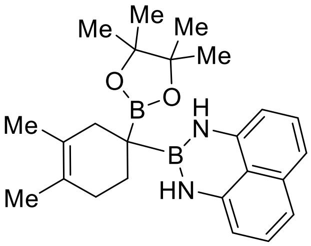

5a

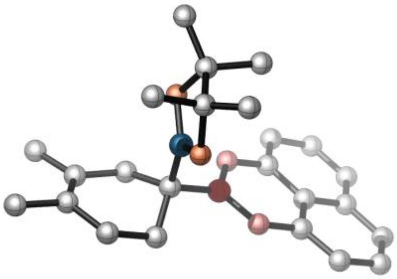

Table 1 Crystal data and structure refinement for AhmadM9.

Identification code	AhmadM9
Empirical formula	$C_{24}H_{32}B_2N_2O_2$
Formula weight	402.13
Temperature/K	293(3)
Crystal system	orthorhombic
Space group	$P2_12_12_1$
a/Å	11.5104(4)
b/Å	13.2932(4)
c/Å	14.6400(5)
α/°	90
β/°	90
γ/°	90
Volume/Å^3	2240.07(13)
Z	4
ρ_{calc}g/cm^3	1.192
μ/mm^{-1}	0.074
F(000)	864.0
Crystal size/mm^3	0.11 × 0.046 × 0.034
Radiation	Mo Kα (λ = 0.71073)
2Θ range for data collection/°	4.138 to 64.2
Index ranges	$-13 \leq h \leq 16$, $-18 \leq k \leq 17$, $-21 \leq l \leq 21$
Reflections collected	24973
Independent reflections	6614 [R_{int} = 0.0432, R_{sigma} = 0.0617]
Data/restraints/parameters	6614/0/277
Goodness-of-fit on F^2	1.074
Final R indexes [I>=2σ (I)]	R_1 = 0.0938, wR_2 = 0.1993
Final R indexes [all data]	R_1 = 0.1633, wR_2 = 0.2300
Largest diff. peak/hole / e Å^{-3}	0.40/-0.17
Flack parameter	0.2(6)

Crystallographic Data for compound (5c):

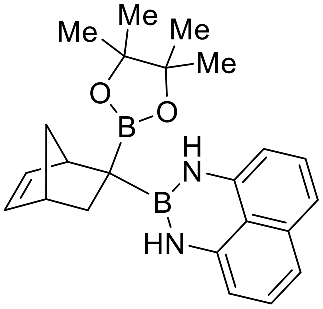

5c

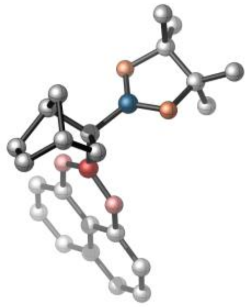

Table 1 Crystal data and structure refinement for AhmadM11.

Identification code	AhmadM11
Empirical formula	C23H28B2N2O2
Formula weight	386.09
Temperature/K	297(1)
Crystal system	orthorhombic
Space group	P212121
a/Å	6.5751(3)
b/Å	11.8098(6)
c/Å	27.4894(13)
α/°	90
β/°	90
γ/°	90
Volume/Å3	2134.57(17)
Z	4
ρ_{calc}g/cm3	1.201
μ/mm-1	0.075
F(000)	824.0
Crystal size/mm3	0.217 × 0.143 × 0.083
Radiation	Mo Kα (λ = 0.71073)
2Θ range for data collection/°	4.548 to 55.996
Index ranges	-8 ≤ h ≤ 8, -13 ≤ k ≤ 15, -31 ≤ l ≤ 36
Reflections collected	18800
Independent reflections	5172 [R_{int} = 0.0625, R_{sigma} = 0.0565]
Data/restraints/parameters	5172/4/302
Goodness-of-fit on F2	1.030
Final R indexes [I>=2σ (I)]	R_1 = 0.0819, wR_2 = 0.1707
Final R indexes [all data]	R_1 = 0.1255, wR_2 = 0.1893
Largest diff. peak/hole / e Å-3	0.31/-0.16
Flack parameter	0.3(10)

Crystallographic Data for compound (5k')

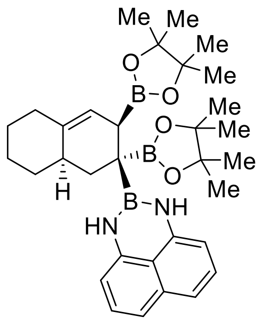

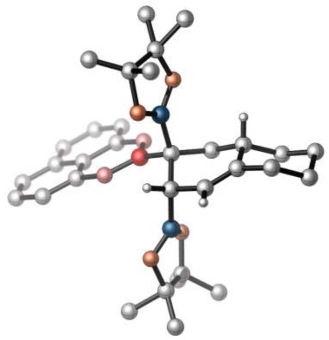

5k'

Table 1 Crystal data and structure refinement for AhmadM19.

Identification code	AhmadM19
Empirical formula	B3C37H57N2O4
Formula weight	626.27
Temperature/K	293(2)
Crystal system	triclinic
Space group	P-1
a/Å	9.9231(2)
b/Å	12.3374(2)
c/Å	16.7882(3)
α/°	71.631(2)
β/°	89.7970(10)
γ/°	75.524(2)
Volume/Å3	1882.31(6)
Z	2
ρ_{calc}g/cm$_3$	1.105
μ/mm-1	0.069
F(000)	680.0
Crystal size/mm3	0.3 × 0.25 × 0.06
Radiation	Mo Kα (λ = 0.71073)
2Θ range for data collection/°	4.254 to 64.726
Index ranges	-14 ≤ h ≤ 13, -18 ≤ k ≤ 18, -24 ≤ l ≤ 25
Reflections collected	63595
Independent reflections	11700 [R_{int} = 0.0313, R_{sigma} = 0.0302]
Data/restraints/parameters	11700/0/530
Goodness-of-fit on F$_2$	1.032
Final R indexes [I>=2σ (I)]	R_1 = 0.0695, wR_2 = 0.1898
Final R indexes [all data]	R_1 = 0.1172, wR_2 = 0.2137
Largest diff. peak/hole / e Å-3	0.25/-0.11

Note: 1. A solvent mask was calculated and 87 electrons were found in a volume of 383\%A^{3} in 1 void per unit cell. This is consistent with the presence of one solvent molecule (Ether / Pentane) per Unit Cell which account for 84 electrons per unit cell. 2. The boronic pinacol ester groups are disordered.

Crystallographic Data for compound (5f-*endo*).

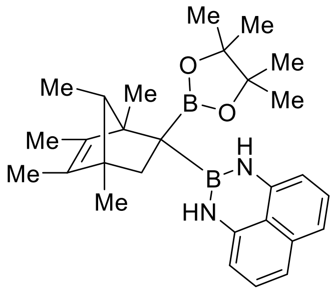

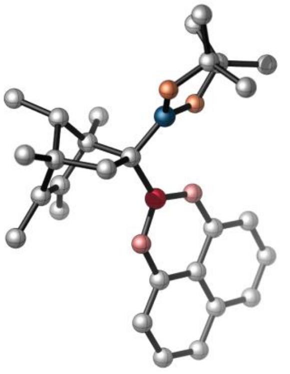

5f-*endo*

Table 1 Crystal data and structure refinement for AhmadM12.

Identification code	AhmadM12
Empirical formula	C28H38B2N2O2
Formula weight	456.22
Temperature/K	296.8(2)
Crystal system	monoclinic
Space group	P21/n
a/Å	10.7733(2)
b/Å	12.8250(2)
c/Å	19.4104(3)
α/°	90
β/°	92.2730(10)
γ/°	90
Volume/Å3	2679.78(8)
Z	4
ρ_{calc}g/cm^3	1.131
μ/mm-1	0.069
F(000)	984.0
Crystal size/mm3	0.418 × 0.326 × 0.192
Radiation	Mo Kα (λ = 0.71073)
2Θ range for data collection/°	3.808 to 64.36
Index ranges	-15 ≤ h ≤ 15, -18 ≤ k ≤ 19, -28 ≤ l ≤ 28
Reflections collected	113270
Independent reflections	8728 [R_{int} = 0.0287, R_{sigma} = 0.0147]
Data/restraints/parameters	8728/0/316
Goodness-of-fit on F^2	1.063
Final R indexes [I>=2σ (I)]	R_1 = 0.0623, wR_2 = 0.1983
Final R indexes [all data]	R_1 = 0.0818, wR_2 = 0.2159
Largest diff. peak/hole / e Å-3	0.54/-0.31

11. Diastereomeric and regioisomeric ratios determination by NMR.

Dr determination by H-NMR for compound (**4f**)

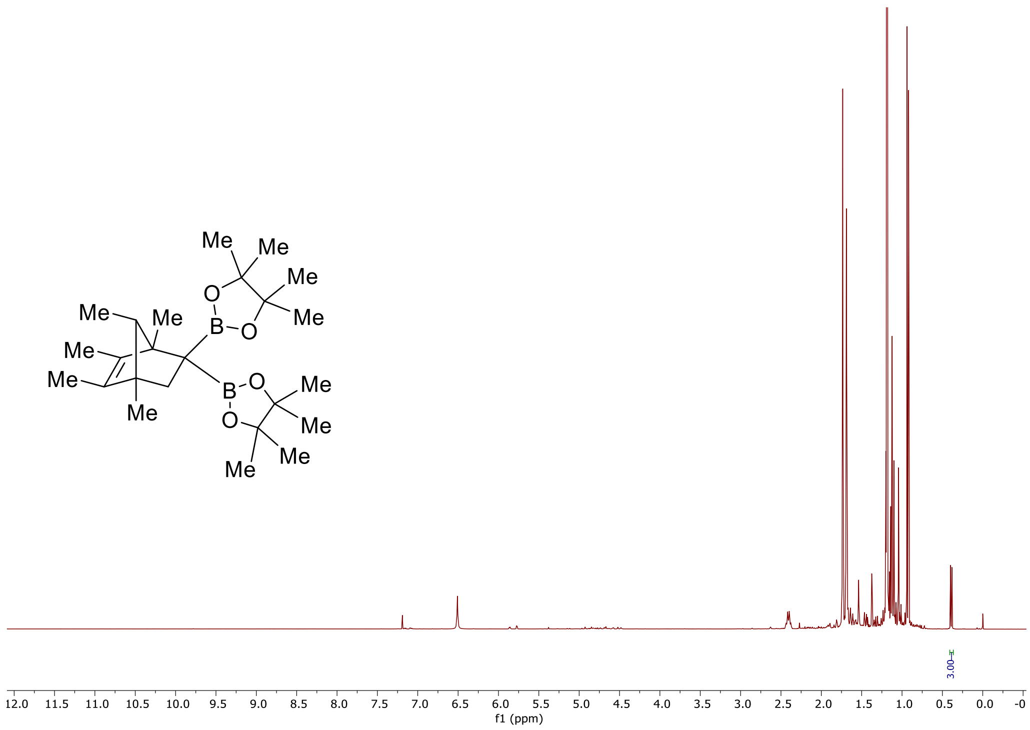

Dr determination by H-NMR for compound (**4h+4h’**)

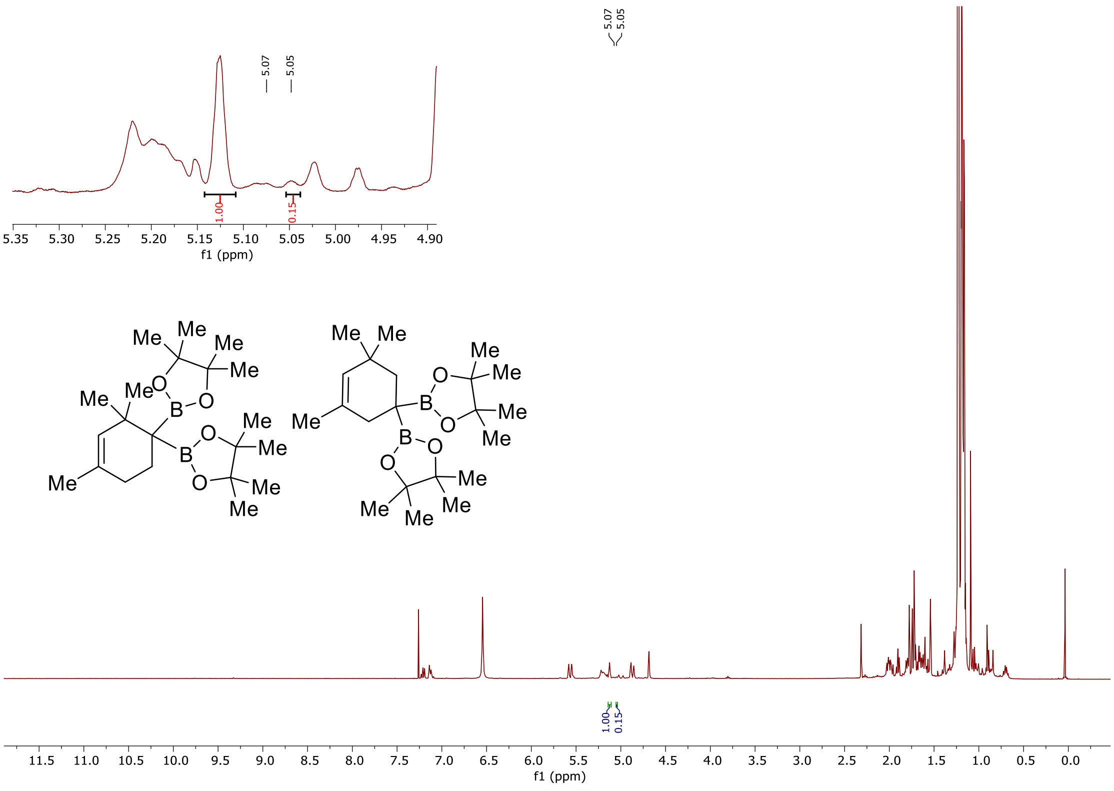

Dr determination by H-NMR for compound (**4j+4j’**)

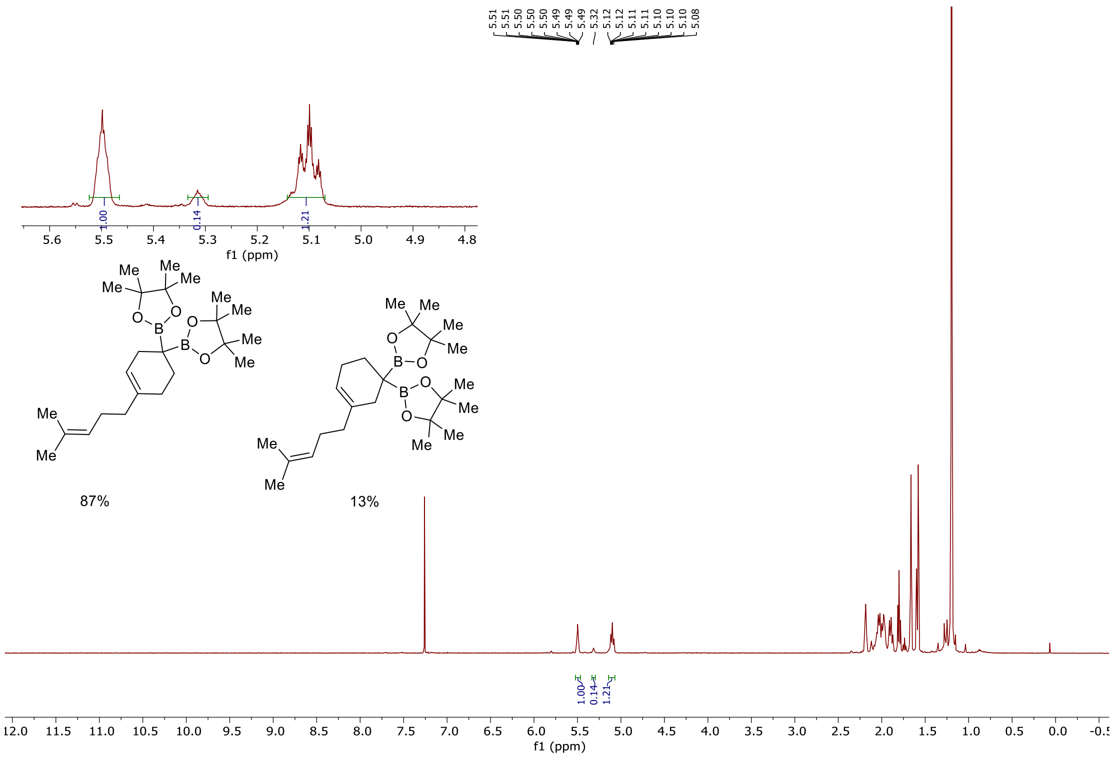

Dr determination by H-NMR for compound (**4i+4i’**)

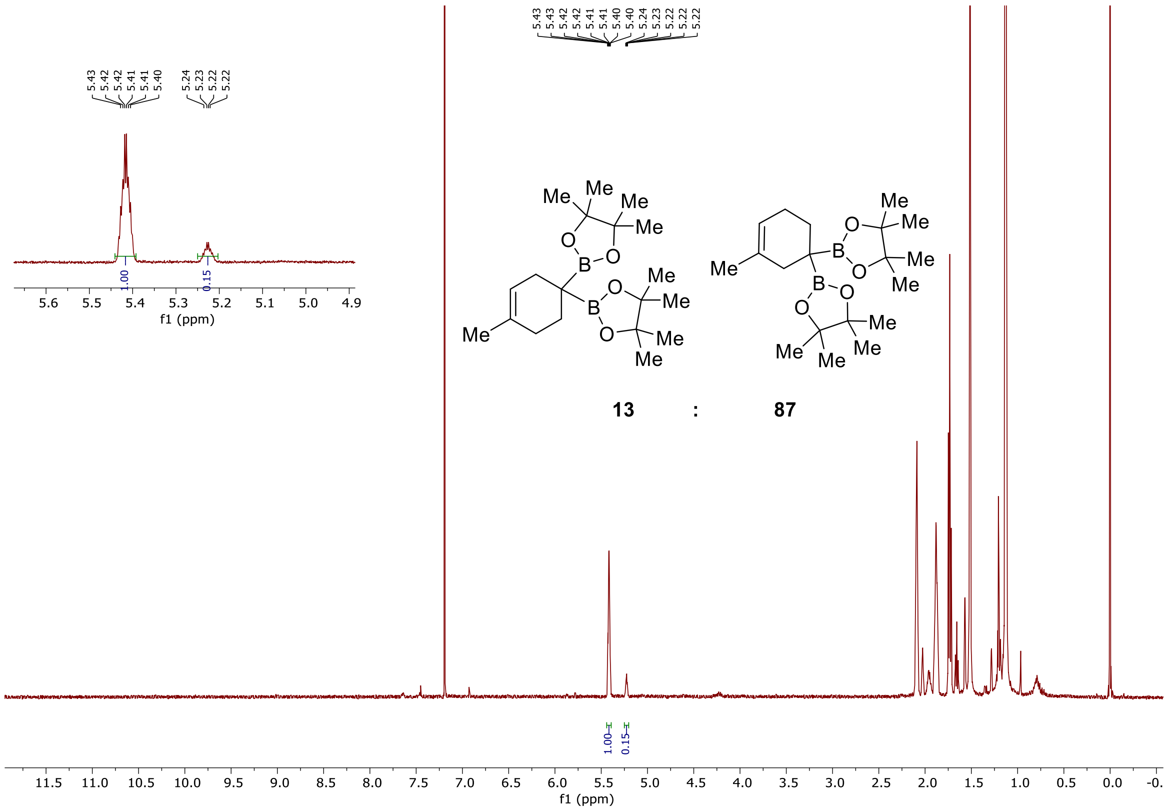

Dr determination by H-NMR for compound (**5c**)

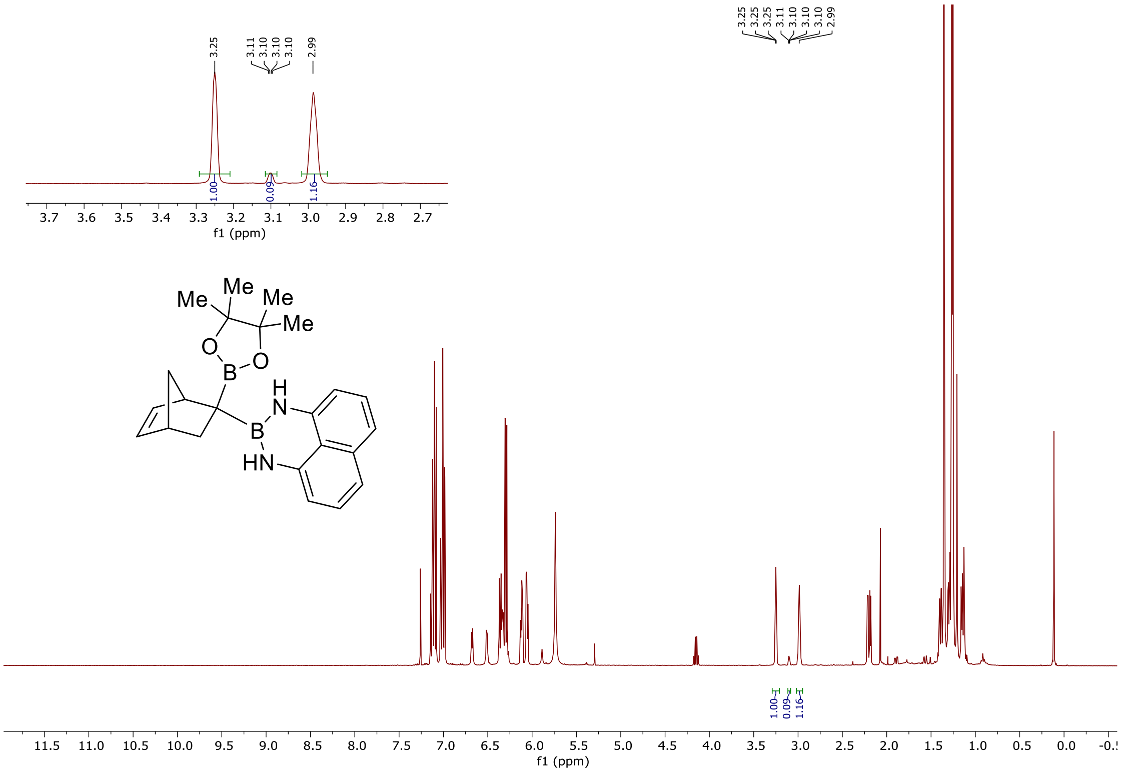

Dr determination by H-NMR for compound (**5f-*endo***)

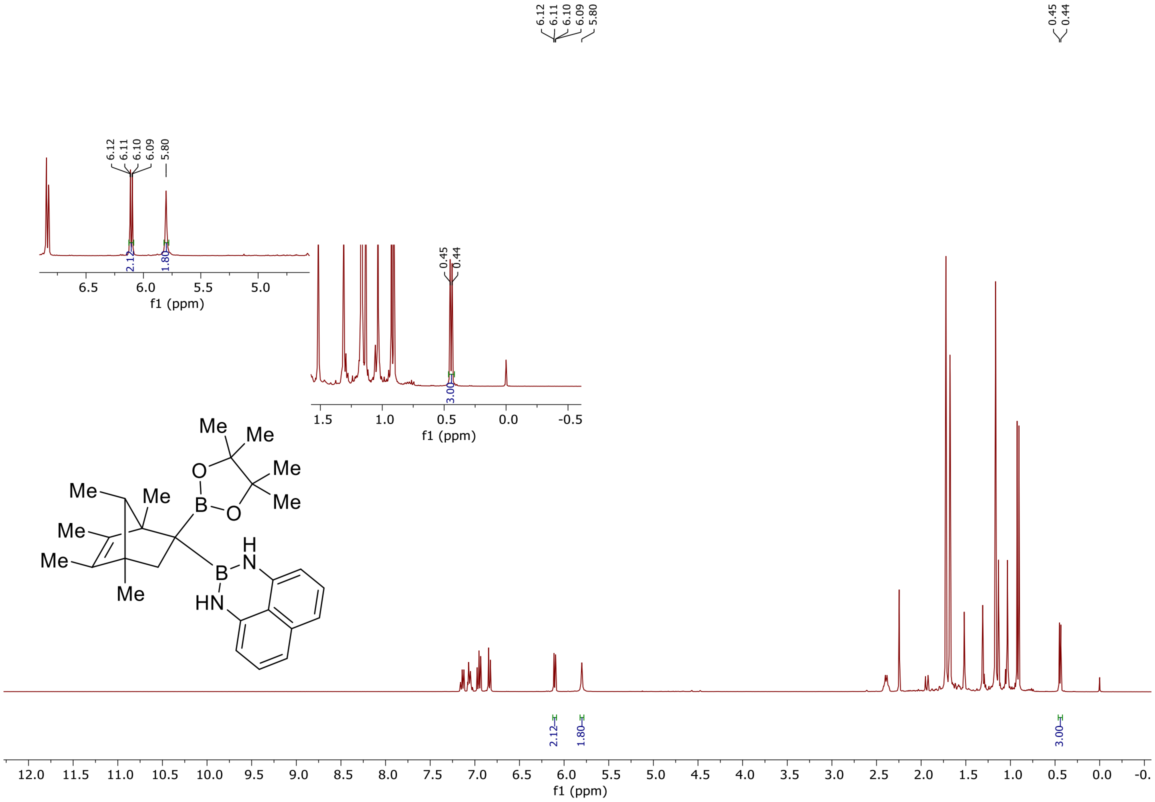

Dr determination by H-NMR for compound (**5k+5k'**)

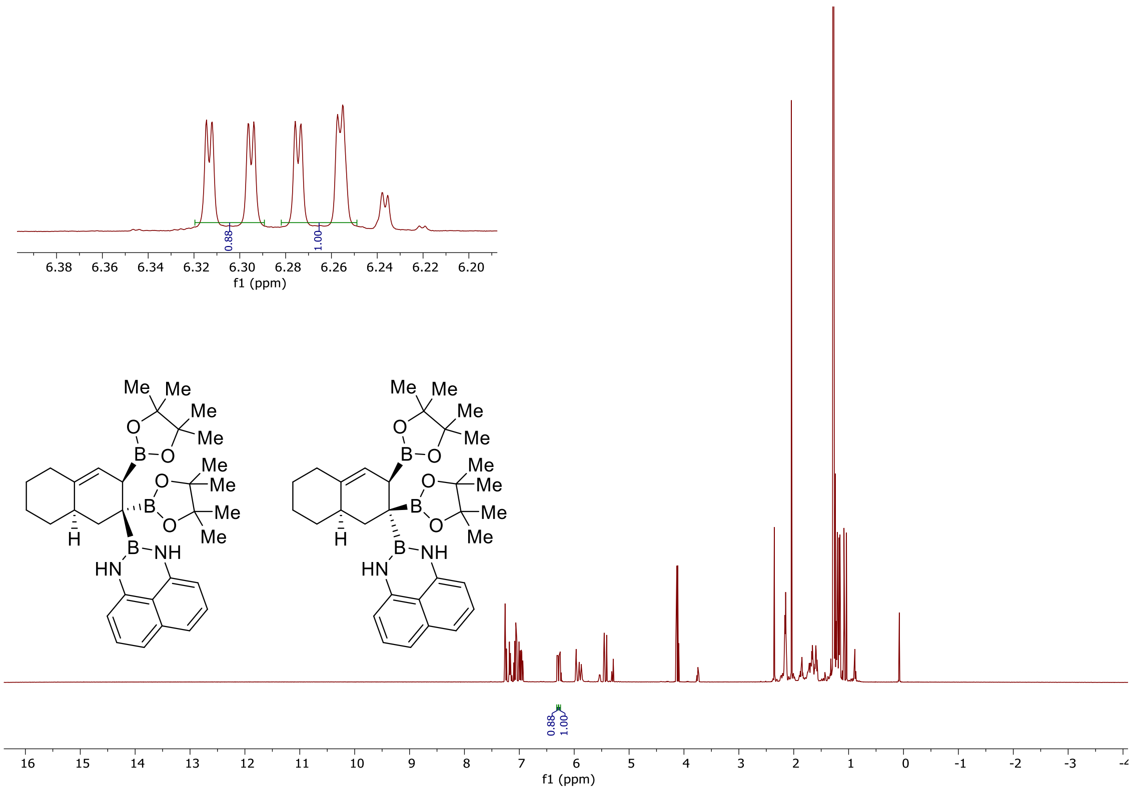

Dr determination by H-NMR for compound (**6b**)

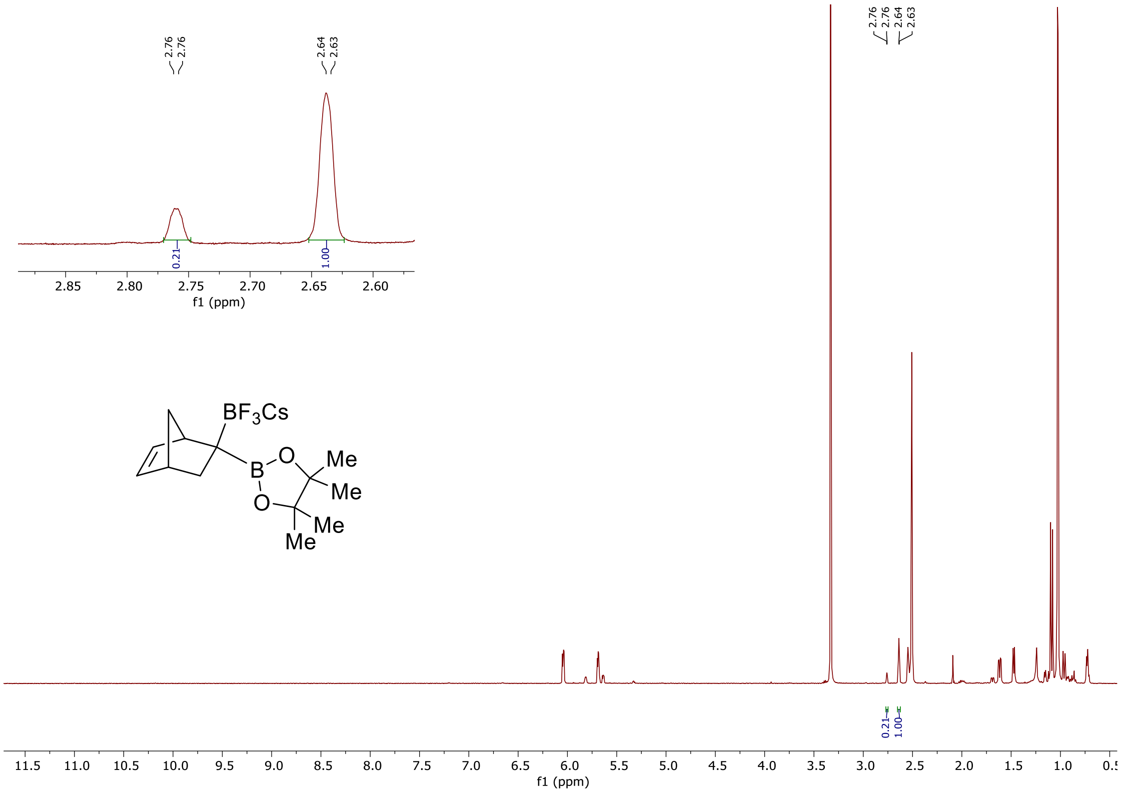

Dr determination by F-NMR for compound (**6b**)

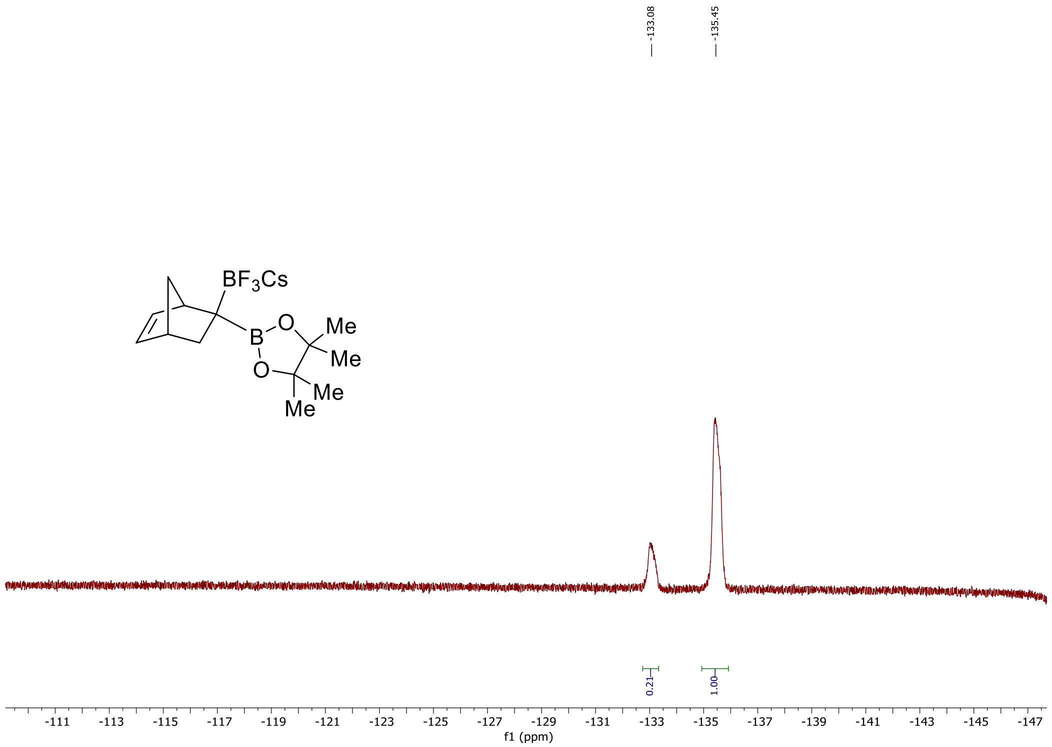

Dr determination by H-NMR for compound (**6d**)

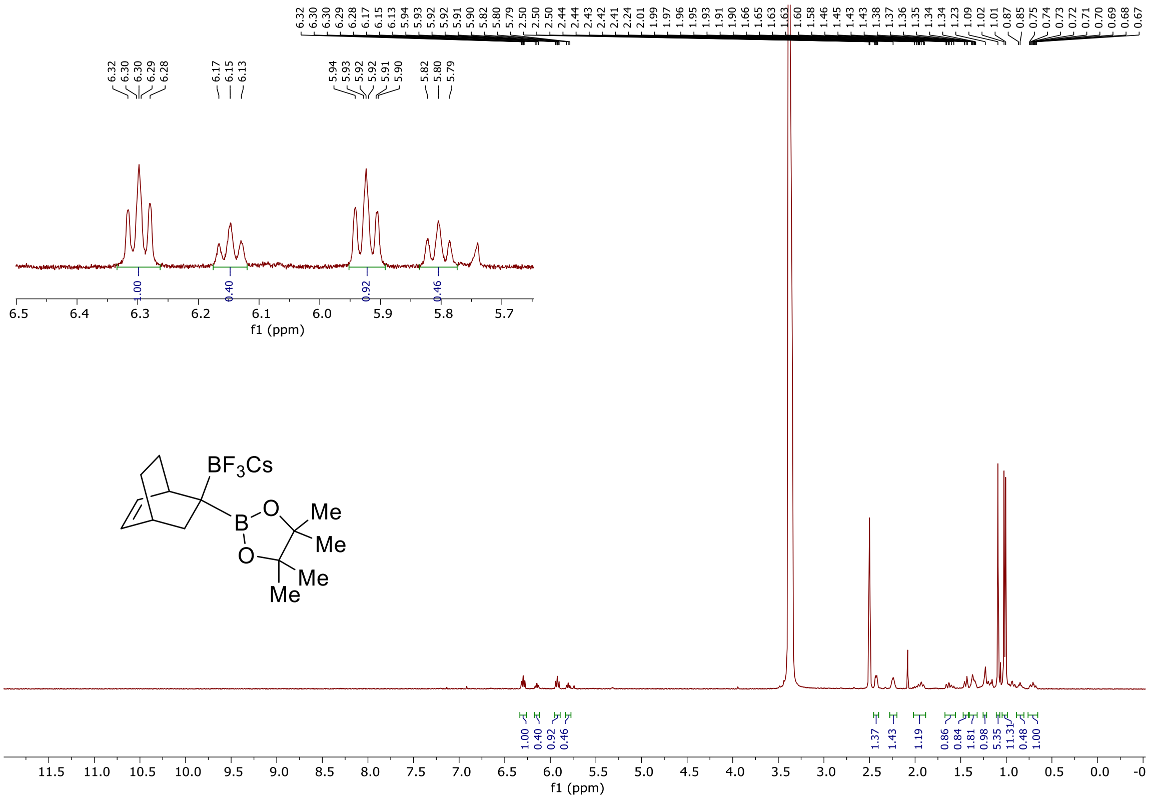

Dr determination by F-NMR for compound (**6f**)

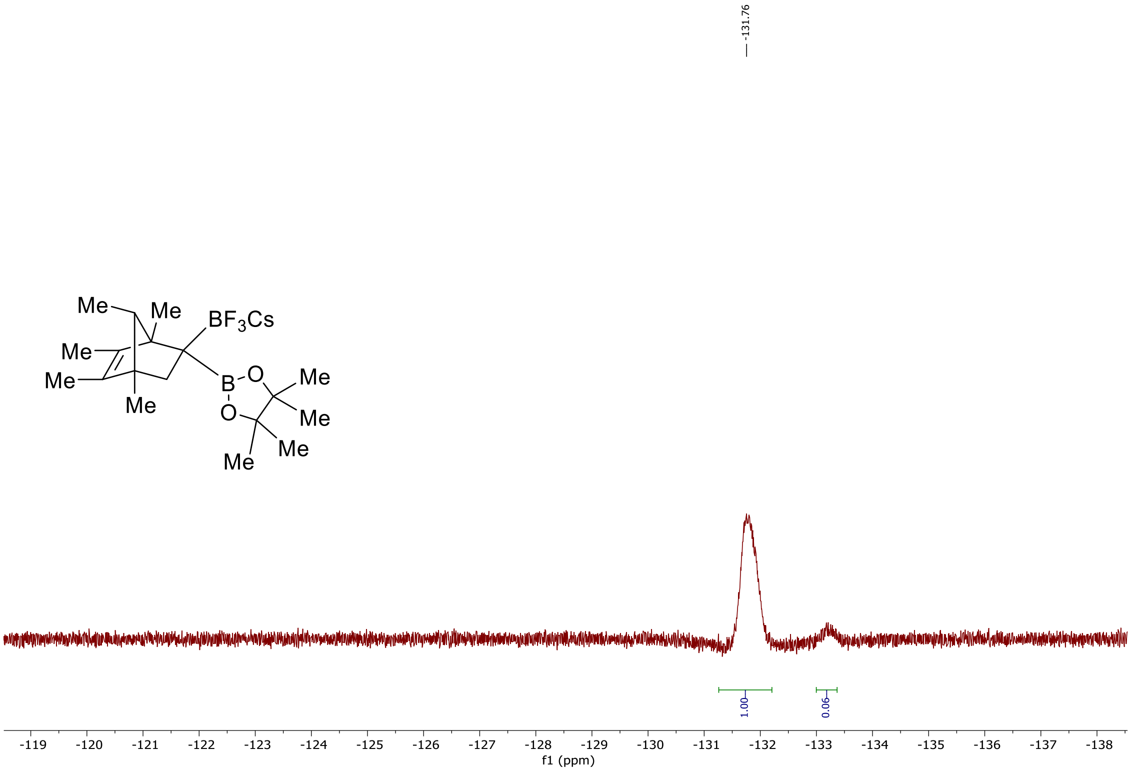

Dr determination by H-NMR for compound (**5f-exo**)

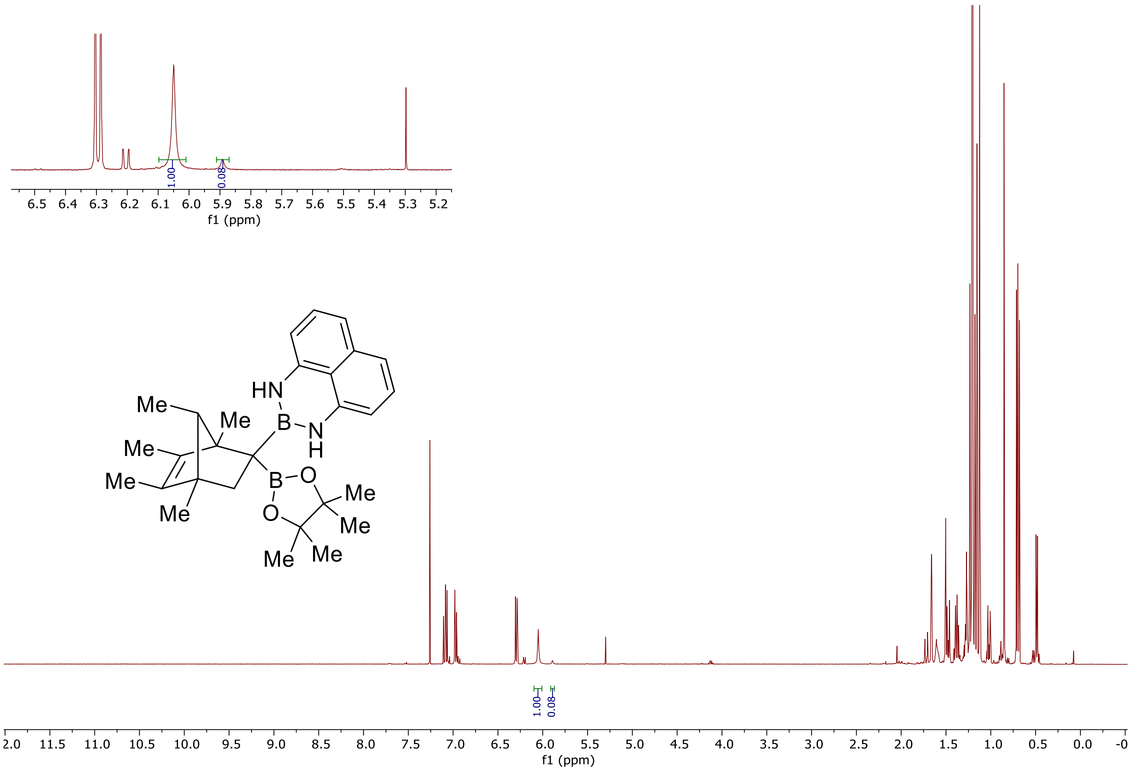

12. NOESY NMR analysis

Analysis for Compound **4f** in ($CDCl_3$):

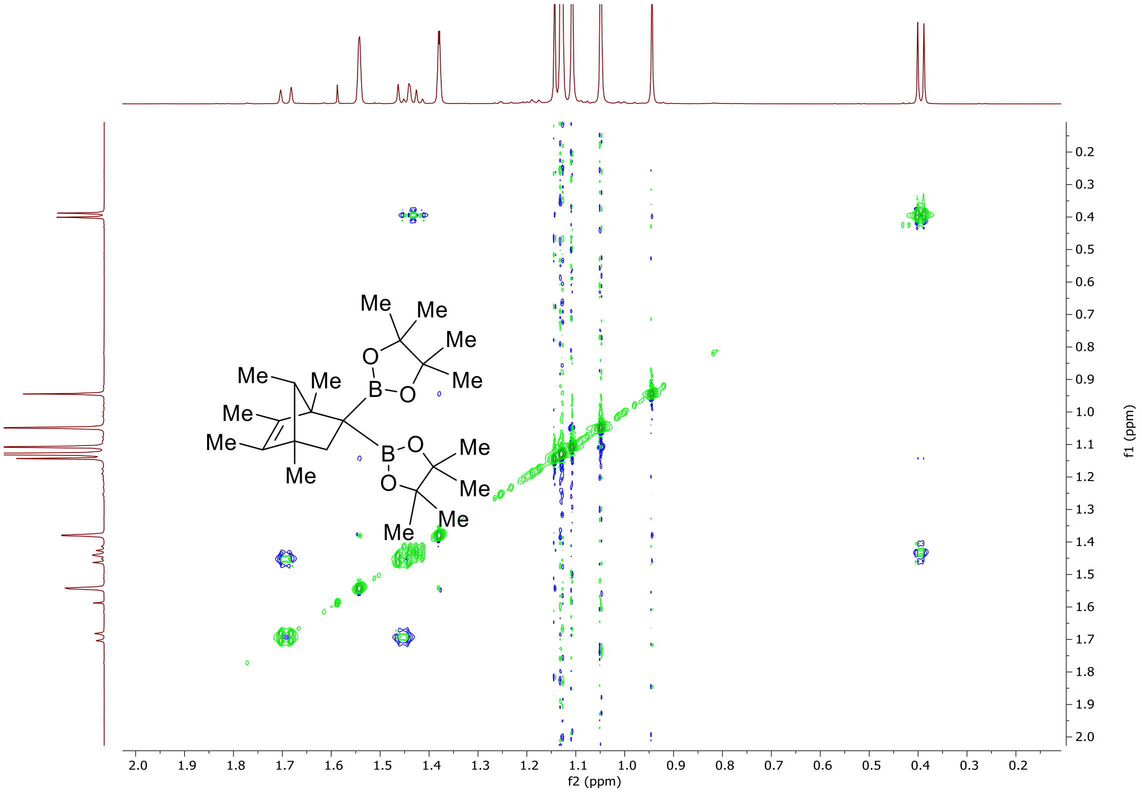

<u>Analysis for Compound **4g-Si** in ($CDCl_3$):</u>

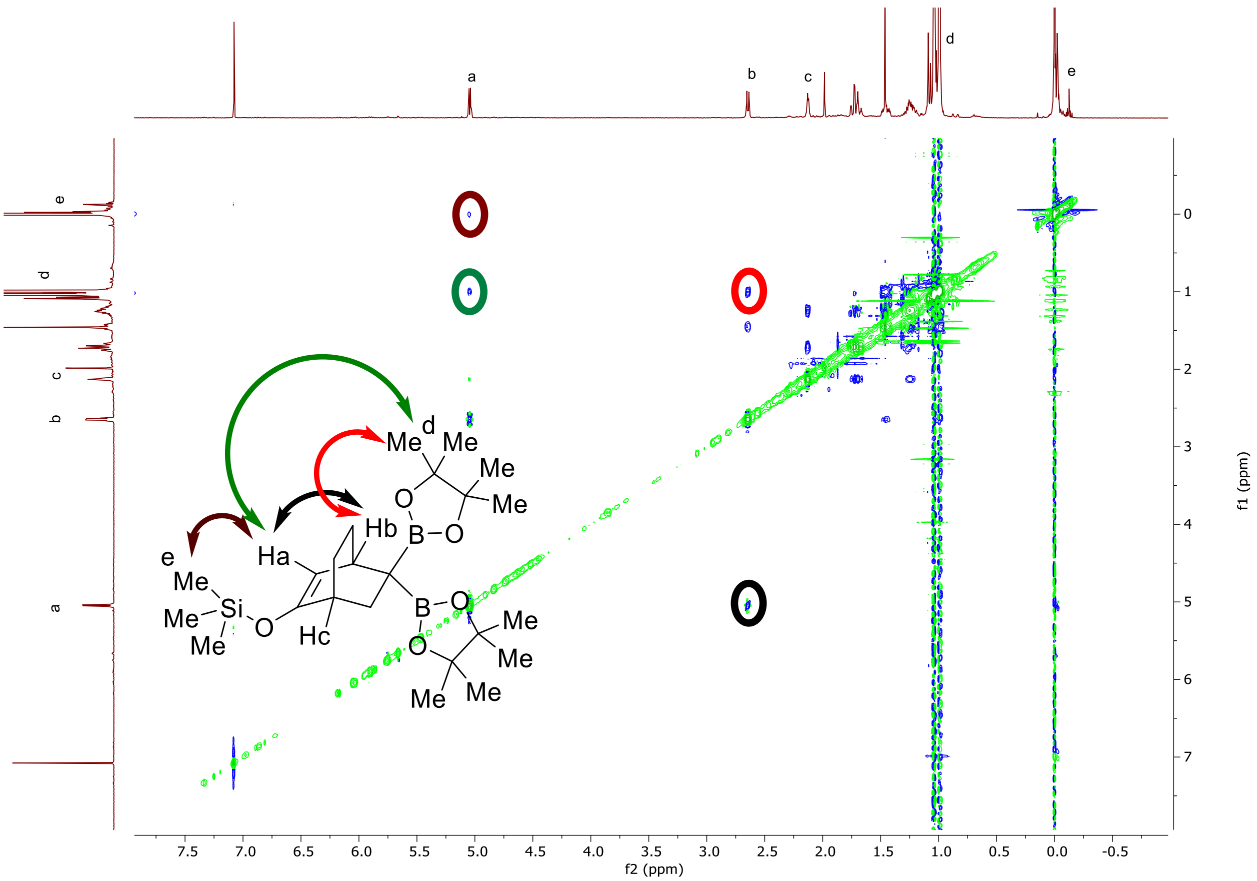

Analysis for Compound **4O-f** in ($CDCl_3$):

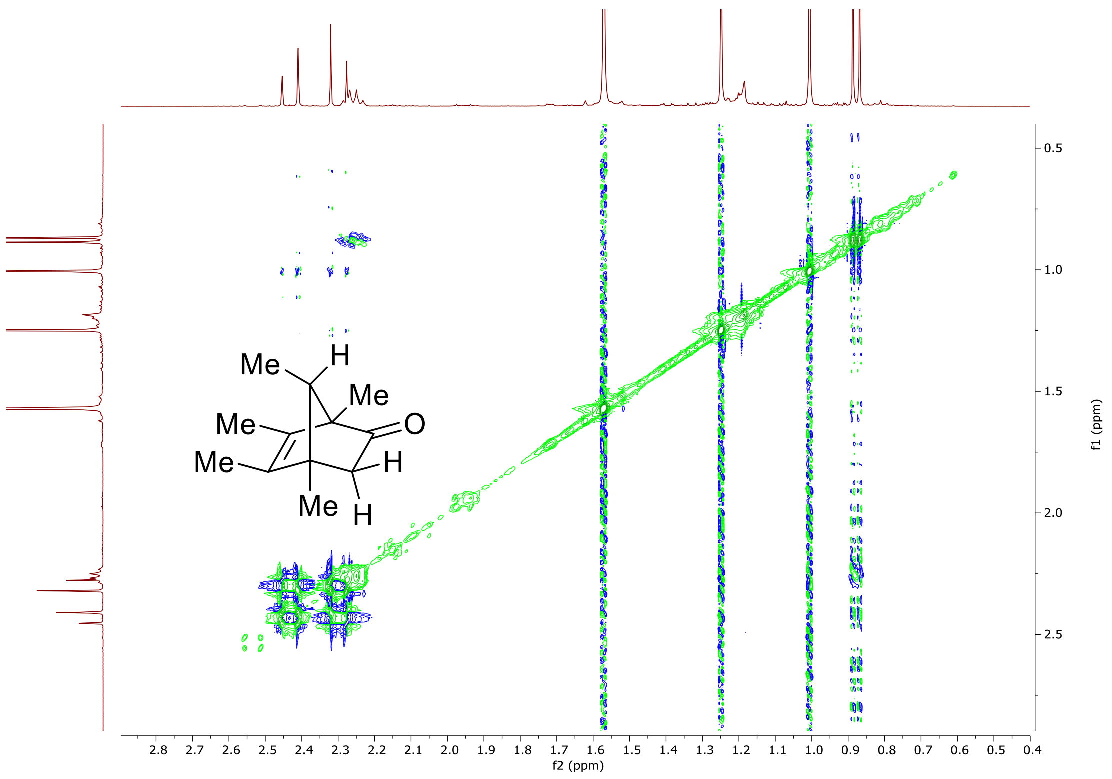

Analysis for Compound **6b** in ($CDCl_3$):

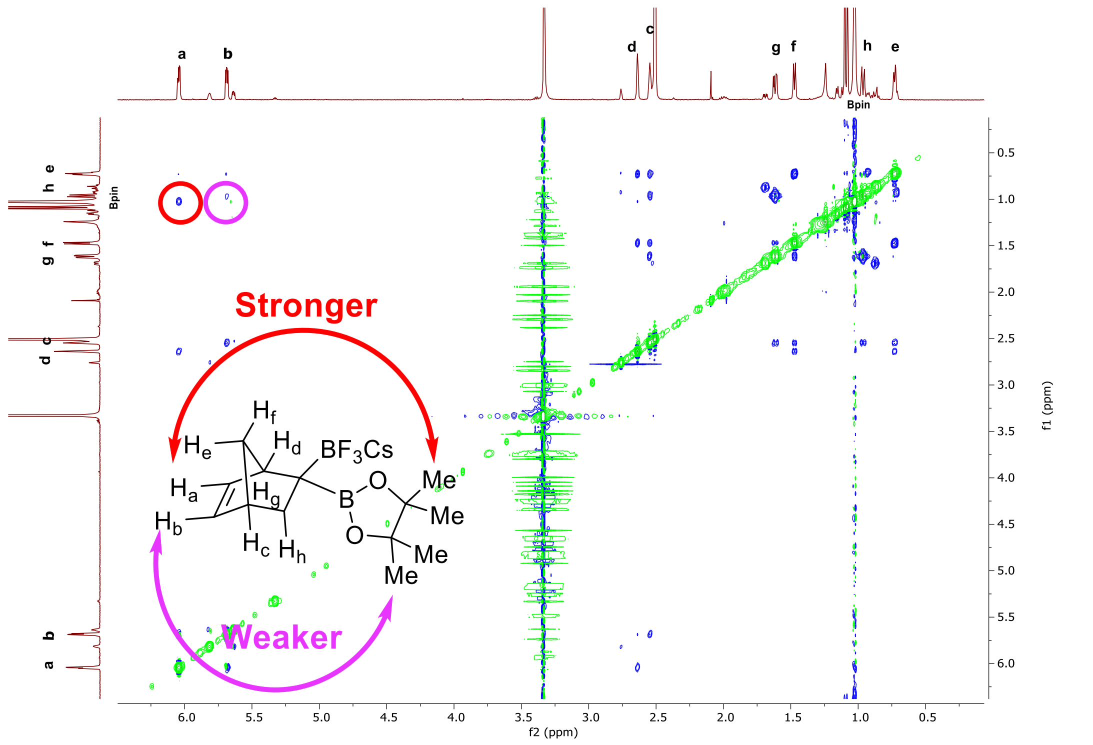

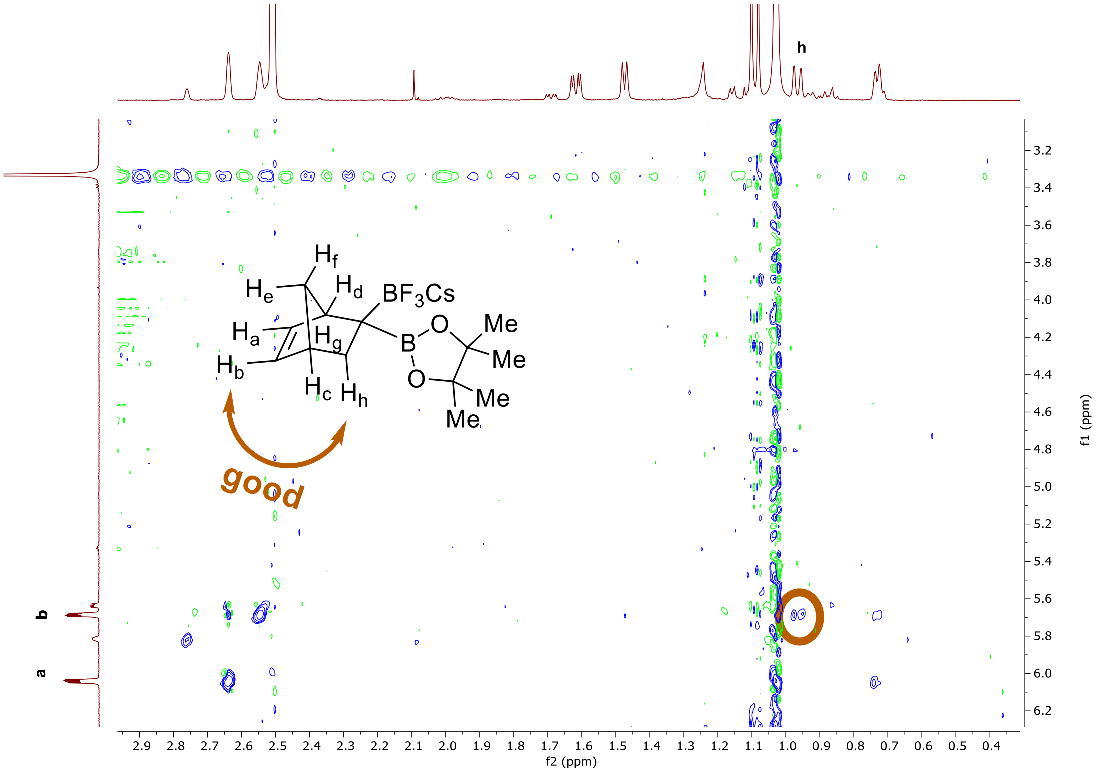
h
b
a
Hf
He
Hd
BF3Cs
Ha
Hg
B
O
Me
Hb
Hc
Hh
good
f1 (ppm)
f2 (ppm)

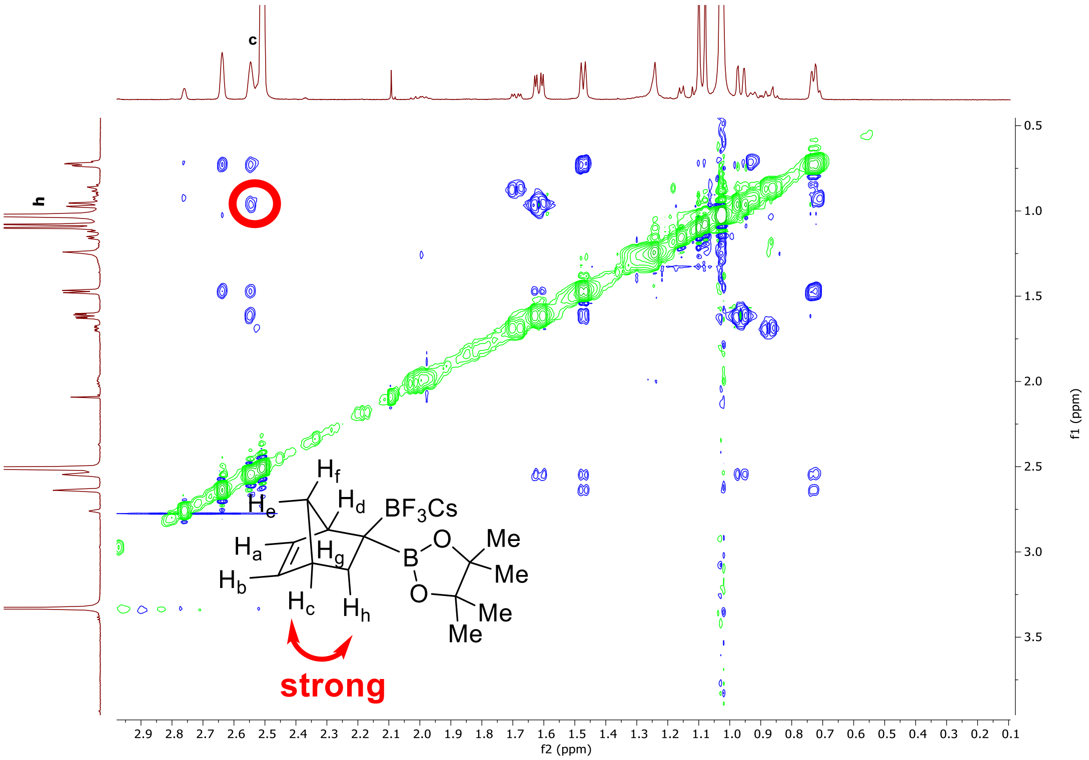
c
h
Hf
He
Hd
BF3Cs
Ha
Hg
Hb
Hc
Hh
Me
strong
f1 (ppm)
f2 (ppm)

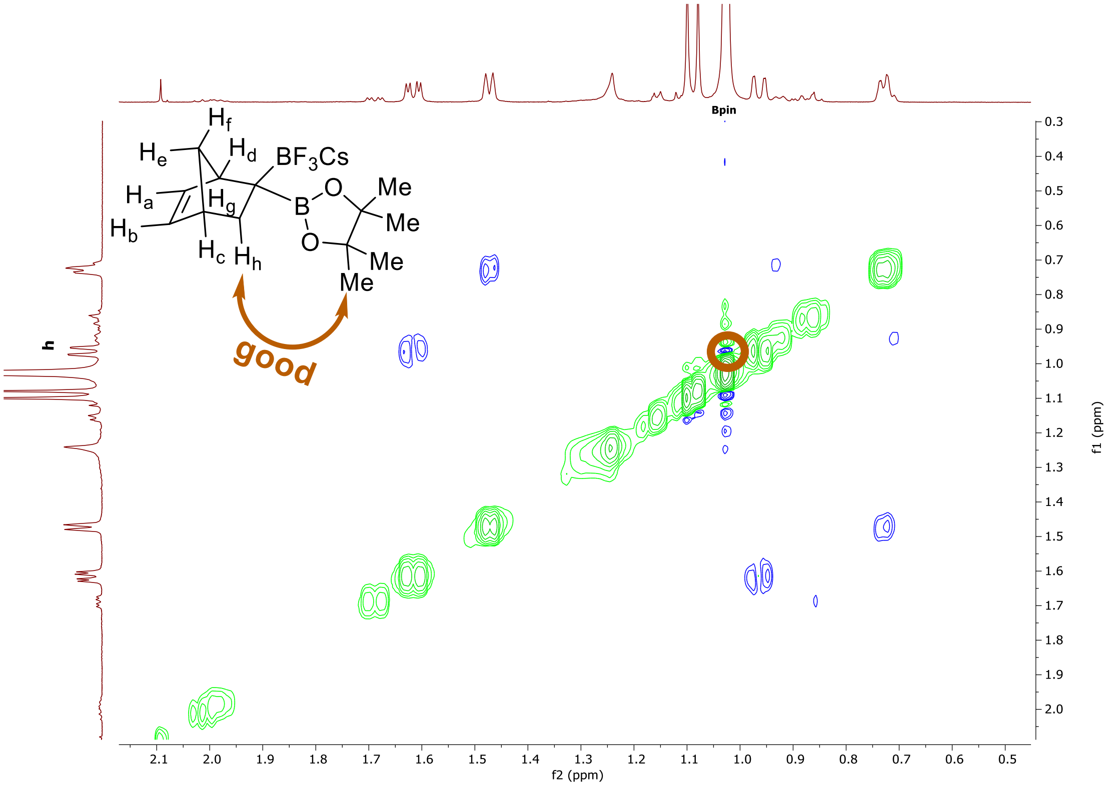

Analysis for Compound **4k** in ($CDCl_3$):

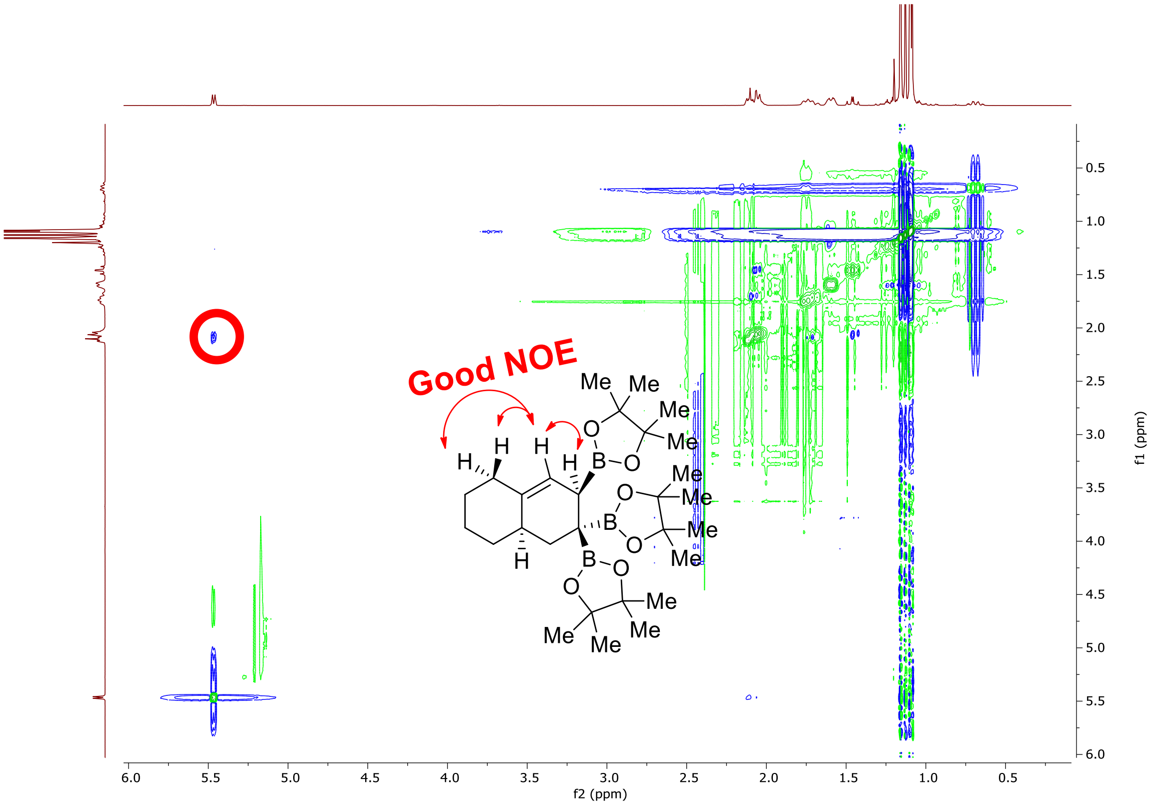

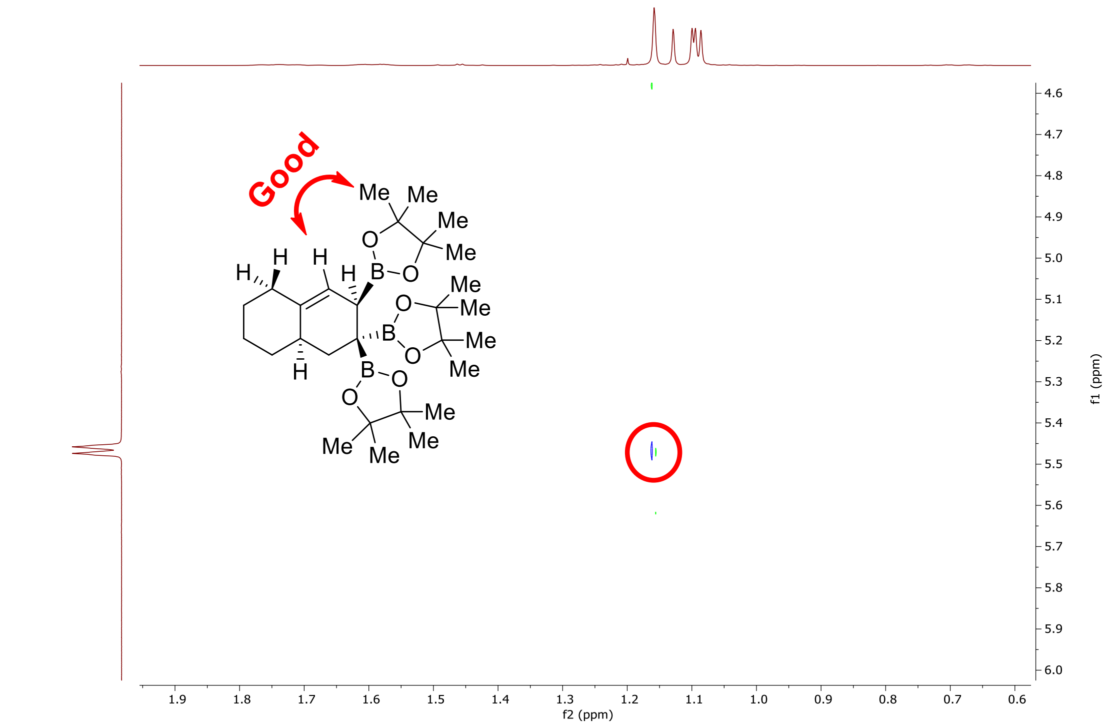

Analysis for Compound **5k'** in ($CDCl_3$):

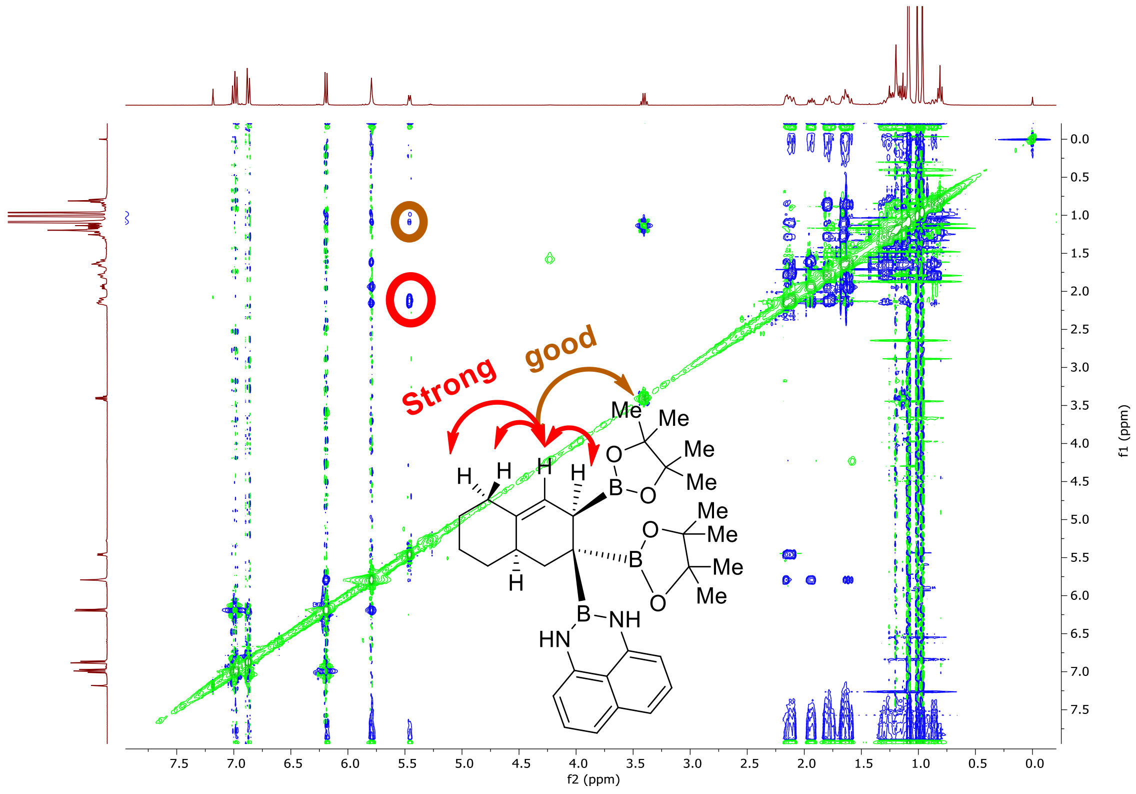

Analysis for Compound **5k** in ($CDCl_3$):

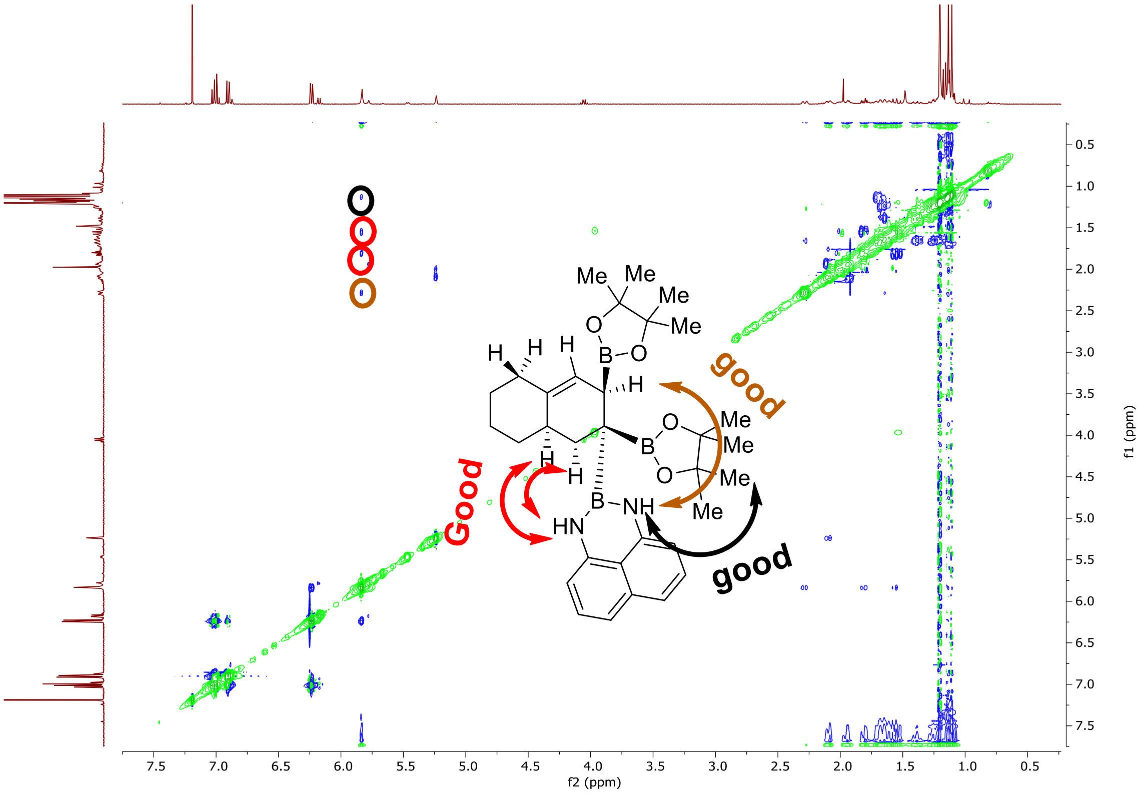

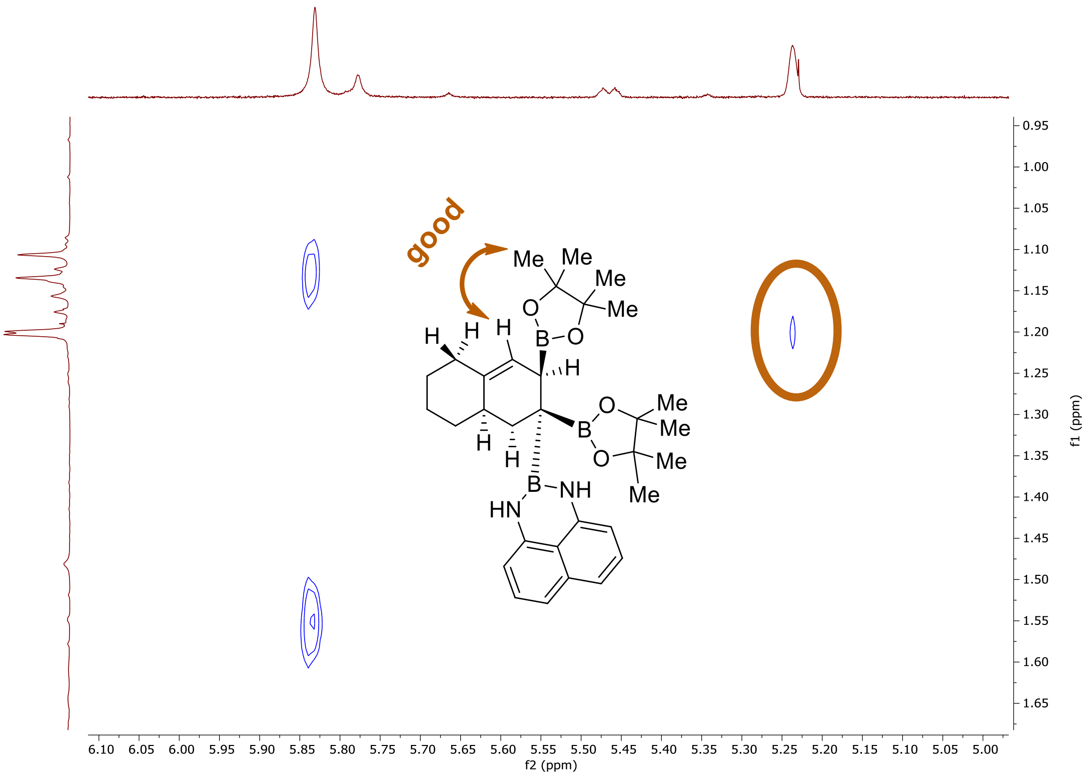

<u>Analysis for Compound **4O-d** in ($CDCl_3$):</u>

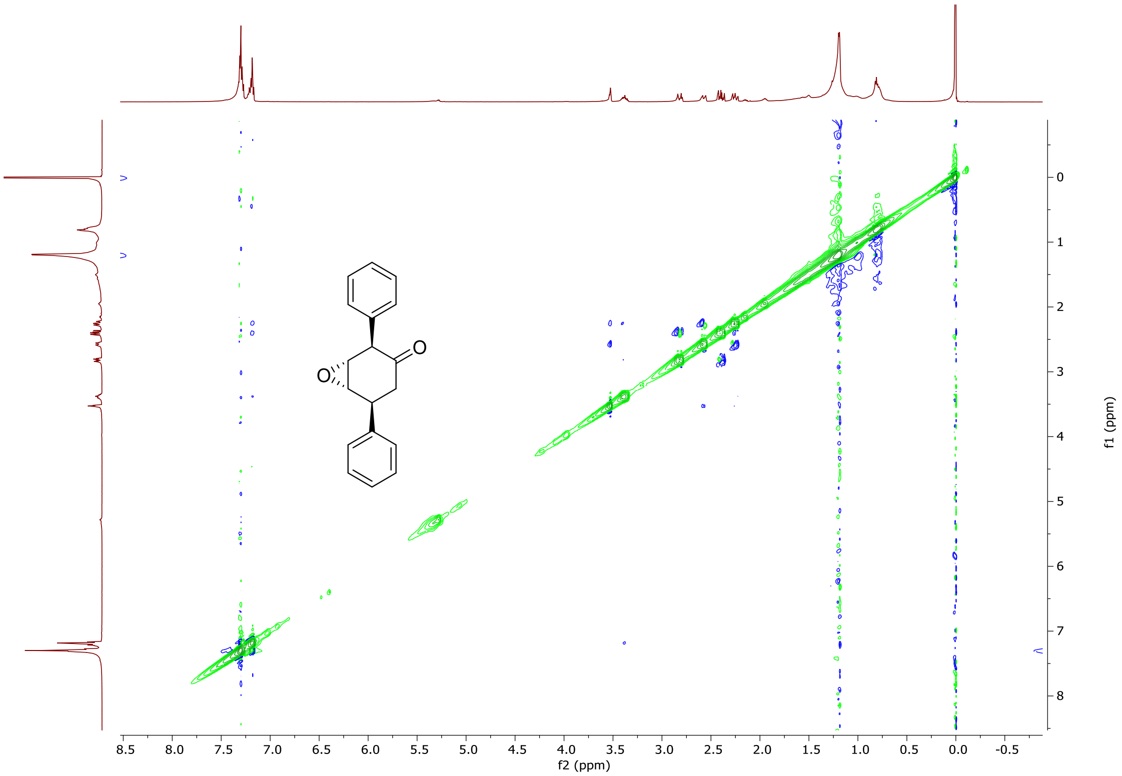

Supplement: Supplementary file 1 — ja1c01471_si_001.pdf [file ja1c01471_si_001.pdf]
